# Ciliary transition zone evolution and the root of the eukaryote tree: implications for opisthokont origin and classification of kingdoms Protozoa, Plantae, and Fungi

**DOI:** 10.1007/s00709-021-01665-7

**Published:** 2021-12-23

**Authors:** Thomas Cavalier-Smith

**Affiliations:** Department of Zoology, University of Oxford, South Parks Road, Oxford, OX1 3PS UK

**Keywords:** Glaucophyta, *Rhodelphis*, Picozoa, Transitional plate, Acorn-V filaments, Infrakingdom Rhodaria

## Abstract

I thoroughly discuss ciliary transition zone (TZ) evolution, highlighting many overlooked evolutionarily significant ultrastructural details. I establish fundamental principles of TZ ultrastructure and evolution throughout eukaryotes, inferring unrecognised ancestral TZ patterns for Fungi, opisthokonts, and Corticata (i.e., kingdoms Plantae and Chromista). Typical TZs have a dense transitional plate (TP), with a previously overlooked complex lattice as skeleton. I show most eukaryotes have centriole/TZ junction acorn-V filaments (whose ancestral function was arguably supporting central pair microtubule-nucleating sites; I discuss their role in centriole growth). Uniquely simple malawimonad TZs (without TP, simpler acorn) pinpoint the eukaryote tree's root between them and TP-bearers, highlighting novel superclades. I integrate TZ/ciliary evolution with the best multiprotein trees, naming newly recognised major eukaryote clades and revise megaclassification of basal kingdom Protozoa. Recent discovery of non-photosynthetic phagotrophic flagellates with genome-free plastids (*Rhodelphis*), the sister group to phylum Rhodophyta (red algae), illuminates plant and chromist early evolution. I show previously overlooked marked similarities in cell ultrastructure between *Rhodelphis* and *Picomonas*, formerly considered an early diverging chromist. In both a nonagonal tube lies between their TP and an annular septum surrounding their 9+2 ciliary axoneme. Mitochondrial dense condensations and mitochondrion-linked smooth endomembrane cytoplasmic partitioning cisternae further support grouping Picomonadea and Rhodelphea as new plant phylum Pararhoda. As Pararhoda/Rhodophyta form a robust clade on site-heterogeneous multiprotein trees, I group Pararhoda and Rhodophyta as new infrakingdom Rhodaria of Plantae within subkingdom Biliphyta, which also includes Glaucophyta with fundamentally similar TZ, uniquely in eukaryotes. I explain how biliphyte TZs generated viridiplant stellate-structures.

## Introduction: rationale of this review

At the eukaryotic cell surface, the poorly understood transition zone (TZ) between the motile ciliary shaft and centriole from which it grows is of great evolutionary and cell biological significance. I show here that the dense transitional plate (TP) forming its core structure has an overlooked underlying filamentous core skeleton conserved in a majority of eukaryotes—those I collectively name discaria, defined as all eukaryotes with a circular, discoid TP. By critical reinterpretation of TZ anatomy across phyla and kingdoms, I also show that all major groups of discaria have the radially asymmetric filament system (acorn-V complex) first discovered in *Chlamydomonas* (Geimer and Melkonian 2004) at TZ's extreme base where doublets change to triplet microtubules. For the first time, I explain that the TZ of malawimonad Protozoa radically differs from that of discaria (i.e., all other eukaryotes) by being ultrashort, without V-filaments and with simpler segment-shaped pre-acorn, which I conclude is the ancestral state for all eukaryotes. This provides extremely strong evidence that the root of the eukaryote tree is between phylum Malawimonada and clade discaria. I reevaluate evidence for the eukaryote root position based on outgroup rooting of sequence trees for 26 proteins of eubacterial origin on eubacterial outgroups (Derelle et al. 2015) and conclude that they support the very same conclusion, which had previously been overlooked.

The present synthesis has major implications for eukaryote classification and phylogeny, and for TZ and centriolar functions. It stemmed from realising that discoverers of *Rhodelphis* (Gawryluk et al. 2019), a remarkable heterotrophic flagellate with an unseen relict plastid unexpectedly related to red algae, had overlooked key aspects of its TZ structure that give compelling evidence for an evolutionary relationship with both glaucophyte algae and another heterotroph of uncertain affinity, *Picomonas* (Seenivasan et al. 2013). This led me to reconsider classification of plant subkingdom Biliphyta and the origin of the remarkable stellate structure of green plant TZs (a major reason for establishing subkingdom Viridiplantae: Cavalier-Smith 1981), during which I found overlooked tomographic evidence for a star-like substructure of the *Chlamydomonas* TP periphery (O'Toole et al. 2003) and was able to generalise star pattern elements to other corticate eukaryotes and find differences in their TZ structure from other discaria. This allowed me to explain the origin of green plant stellate structures by evolutionary hypertrophy of selected parts of the standard corticate TP and/or its more widespread accessory structures.

In so doing I discovered many other unrecognised similarities in ciliary TZ structure across phyla that can be interpreted in terms of more complex common ancestral states than previously recognised coupled with differential losses of certain subcomponents or hypertrophies of others. I concluded that TP structure is an overlooked aspect of eukaryotic cell biology, whose importance and basic unity has not been sufficiently appreciated. This review is a first attempt to develop the implications of these unifying principles by presenting detailed, well illustrated evidence for the major aspects of TZ evolution across all eukaryotes and using it in conjunction with critical reappraisal of multiprotein trees and centriole-related structures to improve understanding of eukaryote overall phylogeny, cell structural evolution, and higher taxonomy. Explaining my conclusions requires more detailed ultrastructural comparisons for the TZ than previously, so the next four sections provide an essential introductory background. I list 28 major conclusions at the end of the review. Reading them after these introductory sections might help orient the reader through the unusually diverse material considered.

## Introduction: expansion of kingdom Plantae

Four decades ago classical kingdom Plantae of Haeckel (1866) was refined by restricting it to those eukaryotes possessing plastids located in the cytosol and bounded by only two membranes and considered to have evolved by one common ancestral enslavement of a cyanobacterium (Cavalier-Smith 1981). Eukaryote algae with plastids located instead within the rough endoplasmic reticulum (ER) and with an intervening periplastid membrane (PPM) were placed instead in a new kingdom Chromista thought to have evolved by one secondary enslavement of a plant cell whose plasma membrane became the periplastid membrane; algae with plastids in the cytosol but with three bounding membranes were then placed in kingdom Protozoa, where euglenoid algae remain, though dinoflagellate algae are now in Chromista as their ancestor secondarily lost the PPM (Cavalier-Smith 2018). In parallel, Cavalier-Smith (1982a) argued that the outer membrane (OM) of plant plastid envelopes evolved from the cyanobacterial envelope OM and predicted that chloroplasts of all three groups then put in Plantae (Viridiplantae, Rhodophyta, Glaucophyta) would all share the same machinery for importing nuclear-coded proteins and all Chromista would share a different import machinery. This perspective, stemming from comparative ultrastructure plus critical thinking about protein-import molecular biology and evolutionary aspects of symbiogenesis—not from sequence trees, proved correct. All Plantae have plastid import machinery comprising an OM translocator Toc whose core channel is of cyanobacterial origin, notably the ß-barrel protein Omp85, plus inner membrane translocation proteins Tic; Toc and Tic are homologous throughout Plantae. Though Chromista retain parts of this protein translocation complex, they additionally evolved a separate shared import process across the novel PPM involving Derlin and other host proteins originally having ER function (Cavalier-Smith 2018).

Plantae as thus defined fall into two subgroups with very different chloroplasts: (1) subkingdom Biliphyta comprising phyla Glaucophyta and Rhodophyta (red algae), which both retained blue or red phycobilisomes and so also unstacked thylakoids as in ancestral cyanobacteria; (2) Viridiplantae (green plants) which ancestrally lost phycobilisomes and use chlorophyll b instead of phycobilins as secondary antenna pigment and whose thylakoids are stacked for greater photosynthetic efficiency (Cavalier-Smith 1981, 1998). Viridiplantae are also united by sharing a ciliary transition zone (TZ) basal cylinder surrounded by a characteristic stellate structure, this TZ structure being unique in eukaryotes, and a particularly important cell evolutionary character as Manton (1965) first emphasised. As red algae never have cilia, ciliary characters could not previously be used to test the classificatory grouping of Glaucophyta and Rhodophyta. This is radically changed by discovery of two non-photosynthetic phagotrophic flagellates (*Rhodelphis*, assigned to new protist class Rhodelphea) which 253-protein sequence trees convincingly show are sisters of Rhodophyta (Gawryluk et al. 2019). Though Gawryluk et al. (2019) cursorily compared *Rhodelphis* ultrastructure with a few protists including glaucophytes, they mistakenly asserted that its TZ structure is unique and 'does not allow the identification of any significant morphological traits common in glaucophyte and *Rhodelphis* cell organisation'. However, they cited no glaucophyte papers and seemed unaware that the long-standing idea that glaucophyte centriolar microtubular roots are cruciate (unlike *Rhodelphis*, but like many—not all—green plants) is erroneous, as independently shown by Heiss et al. (2017) and Cavalier-Smith (2018 in electronic appendix). I show that *Rhodelphis* centriolar roots were misinterpreted and are virtually identical to those of glaucophytes.

**Fig. 1.**
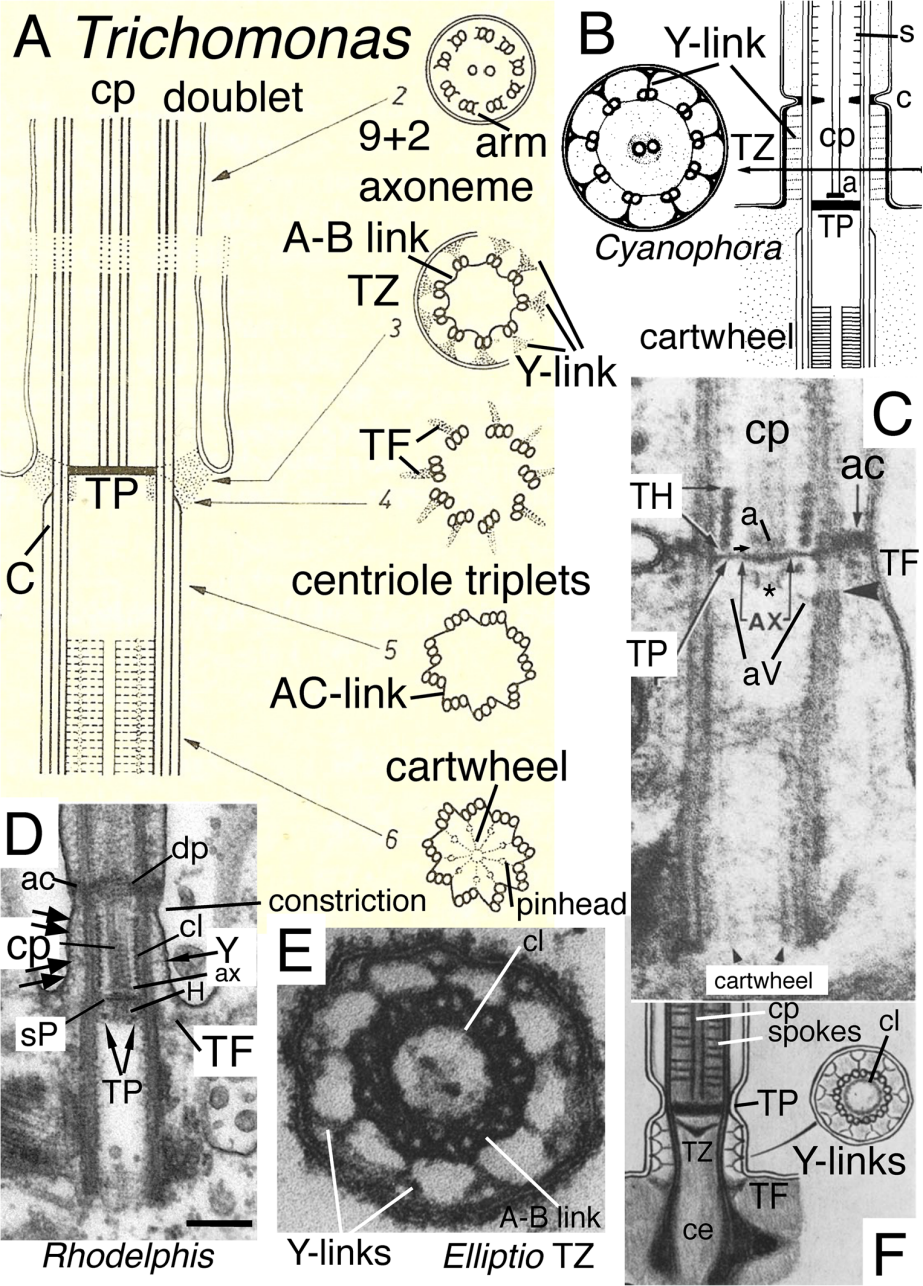
Ciliary transition zones (TZ): conserved and variable features. **A.** Simple type I TZ, exemplified by the metamonad flagellate *Trichomonas*, with single dense transitional plate (**TP**) between the outer doublets at which the central pair (**cp**) microtubules end. Cross sections on right show axoneme (2-4) and centriole (5-6) structure at levels indicated on the longitudinal section on left. 3 shows Y-links;4 shows transitional fibres (**TF**). **C** = triplet C fibre. (After Casper 1974 fig. 32 by permission). **B.** Glaucophyte *Cyanophora paradoxa*; Y-link zone extends above **TP** to the transitional constriction (**c**); it has Y-links but no doublet spokes (s) or dynein arms unlike the distal motile axoneme. A small dense axosome (**a**) terminates cp just above TP. (After Mignot et al. 1969 Fig. 2A, B by permission.)**C.** Chrysophyte alga *Uroglena* type I TZ, like most heterokonts has a dense transitional helix (**TH**) above **TP** just inside the doublets. Its small dense axosome (**a**) is attached to a central axosomal thickening (**AX**) of TP; small arrow = short hub linking **a** to TP. Its dense annular connector (**ac**) links doublets tightly to the ciliary membrane only slightly distal to TP. A central dense hub (asterisk) is present immediately below TP just distal to the point (arrowhead) where centriolar triplet C tubules end and TFs attach. aV = probable acorn-V filaments (From Hibberd 1979 Fig. 1, by permission). **D.***Rhodelphis limneticus* (Biliphyta class Rhodelphea) like glaucophytes has its constriction and associated **ac** plus a diaphragm or distal plate (**dp**) well distal to **TP**, and extended Y-link zone (Y, wide arrows); sP is probably a secondary plate attached just below cp's axosome, but it is possible that 'sP' and 'TP' are actually TP and aV instead (see text; from Gawryluk et al. 2019 Fig. 1r by permission). **H** = hub; **cl** = cylinder-like nonagonal tube, **E-F.** Type II TZ in gill cilia of bivalve mollusc *Elliptio*, with an extended Y-link zone because TP and the constriction have both moved together further from the plasma membrane than in **A** or **C**, making Y-links especially obvious in cross sections (**E**). **F** shows a secondary plate below TP and absence of spokes in TZ. **ce**= centriole. (E-F from Gilula and Satir (1972 fig. 14, 15) by permission.)

Also contrary to their assertion, I show below that *Rhodelphis* TZ structure is fundamentally similar to that of the glaucophytes *Cyanophora* (Mignot et al. 1969; Heiss et al. 2017), *Gloeochaete* (Kies 1976), *Glaucocystis* (Kies 1980), and *Cyanoptyche* (1989) in having (1) a distal diaphragm that traverses the central pair (cp) microtubules (mts) just distal to a TZ constriction; and (2) a transitional nonagonal 'cylinder' close to the outer doublets located immediately distal to the proximal transitional plate. Fig. 1B, D shows the fundamental similarity in TZ structure of the glaucophyte *Cyanophora* and *Rhodelphis* and their joint contrast with shorter distal TZs of typical Protozoa (Fig. 1A), heterokont Chromista (Fig. 1C), and animals (Fig. 1E, F). The only essential difference is that the distal diaphragm has a central aperture in glaucophytes, but not *Rhodelphis*. Both characters are extremely rare in eukaryotes and provide independent ultrastructural synapomophies that unite Biliphyta, if (as I formally enact here) we assign Rhodelphea to subkingdom Biliphyta of Plantae. Though Kies (1989) noted that ciliary ultrastructure was fundamentally similar in all glaucophytes, the evolutionary conservation and significance of their nearly unique TZ structure was not previously reviewed, rectified here.

**Fig. 2.**
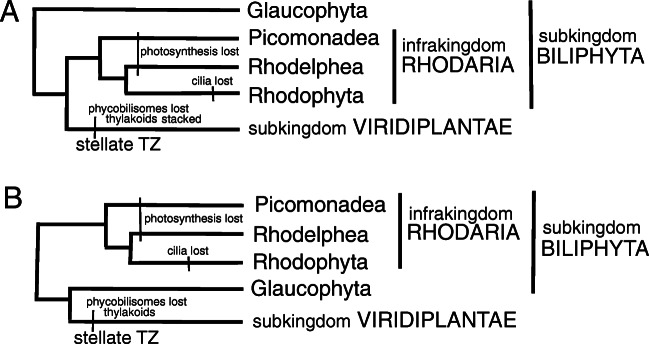
Alternative phylogenies for kingdom Plantae. **A**. This topology with green plants sister to Rhodaria is supported by both chloroplast- and nuclear-coded multiprotein trees and most likely correct. **B.** Some nuclear-coded multiprotein trees suggest instead that green plants are sisters of glaucophytes but no recent well sampled multiprotein chloroplast trees support this. There is essentially no credible multiprotein tree support for the third possibility that Biliphyta are a clade. Therefore biliphytes are almost certainly ancestral to green plants and green plant characters are evolutionarily derived from biliphyte ones

I also show that the only other eukaryote with essentially the same TZ ultrastructure as glaucophytes and *Rhodelphis* is the heterotrophic flagellate *Picomonas* (Seenivasan et al. 2013), originally called a 'picobiliphyte' (Not et al. 2007, who erroneously assumed it was related to cryptophytes), which was sister to glaucophytes on 258-protein trees (Burki et al. 2012) strongly supported by site-heterogeneous PhyloBayes (PB, 0.92 posterior probability support); but very weak ML support. Later sequence tree evidence discussed below plus the previously overlooked ciliary TZ similarity of *Picomonas* to *Rhodelphis* and glaucophytes now lead me to place all three together with Rhodophyta in an expanded subkingdom Biliphyta of kingdom Plantae. Their likely ancestral relationship to Viridiplantae is depicted in Fig. 2A.

The phylogenetic position of *Picomonas* was previously controversial. 187-protein trees (Cavalier-Smith et al. 2015) contradictorily grouped *Picomonas* as sister to Rhodophyta by PB (maximal support) but by ML insignificantly as sister to *Telonema* (a chromist flagellate that by PB grouped weakly with Haptista) and more distantly *Microheliella* (an axopodial nonflagellate that grouped by PB with cryptist chromists with maximal support). Lacking an obvious ultrastructural reason to associate *Picomonas* with red algae, we then doubted that grouping, a doubt stimulated as Leigh et al. (2008) and Deschamps and Moreira (2009) found that some chromist proteins may group with red algal homologues and suggested that these might have come from red algae during the symbiotic origin of chromists and could be misleading as to the relatives of the host component of the chromist chimaera. If that were true, the grouping of *Picomonas* could have been artefactual; as that of *Microheliella* with Cryptista seeemed more reliable, we used other evidence to classify *Picomonas* and *Telonema* also with it. Subsequent work shows that was mistaken; these genera probably branch in separate parts of the corticate tree: *Picomonas* with red algae (Lax et al. 2018; strong support by three methods using 351 proteins) or red algae plus *Rhodelphis* (Gawryluk et al. 2019; maximal support by two of three methods using 253 proteins) and *Telonema* with Harosa. A 248-protein tree (Strassert et al. 2019), including three genically well sampled *Telonema* species (earlier trees had only one, poorly sampled genically) but no *Picomonas*, shows beyond reasonable doubt that phylum Telonemia is sister to Harosa, not related to Cryptista as Cavalier-Smith et al. (2015) suggested. Thus the best available multiprotein tree evidence shows Rhodaria as defined here (Fig. 2A) as a clade. Ultrastructural characters that led us to group *Picomonas* and *Telonema* with *Microheliella* and Cryptista as suggested by ML trees (Cavalier-Smith et al. 2015) are less convincing than the TZ characters elucidated here.

I also draw attention to rare mitochondrial and endoplasmic reticulum (ER) characters that provide further support for a specific relationship between *Rhodelphis* and *Picomonas*. This paper therefore expands plant subkingdom Biliphyta by adding *Picomonas* and *Rhodelphis* and grouping both with red algae as new infrakingdom Rhodaria. It discusses TZ evolution in Biliphyta, contrasting it with that of green plants and Chromista. I explain for the first time how the seemingly unique stellate green plant TZ could have evolved from the simpler biliphyte TZ by multiplying elements of its TP in ways analogous to the TZ hypertrophy in biliphyte pseudocilia.

I begin by summarising the major features of TZ architecture and its delimitation from the centriole; then provide the first comprehensive treatment of the glaucophyte TZ before explaining how those of Rhodaria are related, and discussing origin of green plant stellate TZs from biliphytes. Understanding the origin of the green plant TZ is especially important for effectively using insights from the superb model system for ciliary biology provided by the viridiplant *Chlamydomonas reinhardtii* (Randall et al. 1967; Cavalier-Smith 1974; Geimer and Melkonian 2004, 2005; Pigino et al. 2009) in conjunction with those of the ciliate chromists *Tetrahymena* and *Paramecium*, which as corticates also differ in some crucial respects from those of animals, and trypanosomes which are evolutionarily deeply divergent from both plants and animals. I show that ciliates, Rhizaria, and *Trypanosoma* (and many other eukaryotes) have an overlooked asymmetric acorn-V structure at the apex of their centrioles first discovered in *Chlamydomonas* (Geimer and Melkonian 2004) and discuss evolution of the TZ/centriole junction in eukaryotes generally. In so doing I show that elements of the TZ hub-lattice and distal nonagonal fibre structures discovered in Rhizaria (Cavalier-Smith et al. 2008a, 2008b, 2009) exist also in Plantae, some other corticates, and a majority of eukaryote lineages and are thus of broader significance for eukaryote TZs than hitherto appreciated; correct some past interpretative errors of TZ and centriole comparative anatomy; adduce evidence for numerous overlooked ultrastructural homologies within and across phyla; argue that a filamentous skeleton of the dense TP is conserved to some degree across all eukaryotes; and provide a novel explanation of the ancestral role of the acorn-V filament complex.

## Introduction: ciliary transition zone function and evolution

Normal ciliary axonemes have a cylinder of nine outer doublet mts whose A tubules are furnished with tangential dynein arms (usually an outer and inner, the latter heterogeneous) plus radial spokes (of three different kinds) that interact with projections from the cp mts. The ciliary TZ was originally defined as the basal region of cilia lying between the upper end of the centriole (=basal body) where its triplet C tubules end and where axonemal cp mts begin (Gibbons and Grimstone 1960). This definition is adequate for simple cases like the metamonad flagellate *Trichomonas* shown in Fig. 1A, whose TZ is short and simple, which likely represents the ancestral condition. However, it breaks down in cases where the 9+2 axoneme does not have its canonical structure at its proximal end and is clearly transitional in substructure at its base. One example is in heterokont chromists where most lineages have a single or double dense transitional helix (TH) surrounding the base of the central pair (Hibberd 1979; Cavalier-Smith and Chao 2006)—Fig. 1C. Their TH has been discussed by taxonomists and evolutionists for decades, but it has not previously been explicitly pointed out that TH structure and mode of attachment to A tubules is incompatible with the presence of radial spokes (Barber et al. 2012) and inner arms, both of which must compete for bingeing to the same doublet regions as the TH attachment protein(s). Doublet regions with a TH also never have dynein arms so cannot generate mutual sliding forces. The original TZ definition is also unhelpful in cases where motile cilia altogether lack a cp, or where the centriole has doublets not triplets, both true of centric diatoms (Manton and von Stosch 1966), a major heterokont subgroup.

**Fig. 3.**
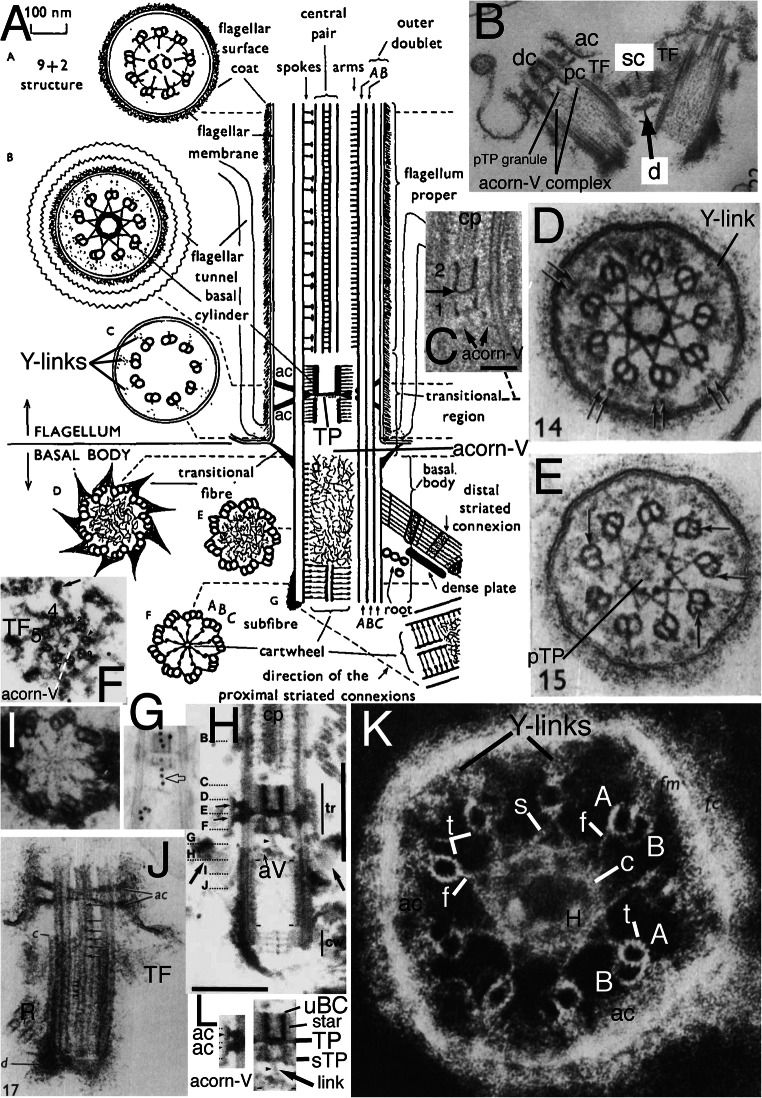
Ciliary and centriolar structure of Viridiplantae as shown by *Chlamydomonas reinhardtii*. **A.** Drawing shows LS of cilium and centriole (G) and six TSs at levels A-F**. B.** LS through isolated centriole/TZ complex shows physical connection of centrioles via striated connector (**sc**), of TZ to ciliary plasma membrane via two annular connexions (**ac**) and transitional fibres (**TF**), and of dense plate (**d**) to sc despite cell homogenisation. **C.** Tomographic slice of freeze-substituted wild-type TZ showing that the 'H cross piece' separating distal and proximal basal cylinders is composite: the base of the longer distal cylinder is denser than the distal septum of the shorter proximal cylinder. Note that the proximal septum (not included in diagram **A**) has a central granule (visible also in **B**) connected by an oblique linker to the acorn-V, which is more clearly distinct from the centriole in **H** after detergent extraction that removes centriolar matrix but retains acorn-V. One cp mt apparently is lodged within the lumen of the distal of basal cylinder (or attached to a distal septum or matrix within it); diagram misleadingly shows empty lumen. **D**, **E**. TSs of distal (**D**) and proximal (**E**) TZ stellate structures in cell homogenates without detergent treatment showing ciliary coats and that the distal basal cylinder has more dense material around its inner-facing obtuse star points. **D.** Arrows mark paired granules characteristic of Y-links. **E.** Arrows mark projections into lumen of B tubules. The central granule implies that this section includes the proximal transverse plate (**pTP**) and part of its linker to the underlying acorn-V (in LS in **H**). **F**. TS through TFs and acorn-V filament system in detergent-extracted isolated ciliary apparatus. V filaments are attached to doublets 4, 5; doublets 3-6 show distal parts of C tubules but the five linked to peripheral acorn filaments do not. **G.** Anticentrin gold-label extends through link between acorn-V and TP. **H**. Medial LS of detergent extracted cilium showing detergent resistant-membrane remnant linked by acs to doublets and proximal septum of proximal basal cylinder joined to radially asymmetric acorn-V (aV) by a slanting centrin link (arrowhead) shown separately in **L** to allow labelling and to demonstrate that both acs are horizontal, not slanting towards TP. **tr** = double stellate region of TZ. **cw**= centriole cartwheel zone. Radial asymmetry of acorn-V and centriolar regular A/B tubule inner projections throughout centriole above cartwheel are obvious as matrix is dissolved. **I.** TS of cartwheel in isolated centriole (no detergent extraction). **J.** Tangential LS of isolated TZ/centriole (no detergent) showing end of C tubules (***c***), two ***ac***s, 2-mt root (**R**), **T**F; arrows mark end-on doublet outer projections, arrowheads A-C connections; ***d*** = dense fibres at centriole proximal end. **K.** Negatively contrasted transverse view of isolated TZ (no detergent) resolves doublet mt protofilaments (**t**) and protein subunits of star filaments (**s**), of A-tubule feet (**f**), of Y-links, and of filaments of the basal cylinder (**c**); surface coat (***fc***) is outside the ciliary membrane (***fm***). **ac** material partially obscures Y-links. **H** = dense material thickening basal cylinder filaments to resemble a dense hub. (A, B, D, E, I-K from Cavalier-Smith (1974 figs 5, 13, 14, 15, 17, 18, 22); C from O'Toole et al. (2003 Fig. 3F); F, H from Geimer and Melkonian (2004 fig. 1G,K), and G from Geimer and Melkonian (2005 fig. 1.8), all by permission.)

In marked contrast to the highly conserved motile 9+2 axoneme, TZ is immensely variable amongst different eukaryote lineages, though rather constant within most major lineages, so was early recognised as an outstandingly valuable character for use as a marker of true evolutionary affinity (Manton 1963; Casper 1974, who depicted 32 major variants). Shared TZ variants often revealed affinities before sequence trees confirmed them, as in the heterokont TH or the completely different stellate structure and basal cylinder of Viridiplantae (Manton 1964, 1965; Cavalier-Smith 1974, 1981) depicted in Fig. 3. Pitelka (1974) pointed out that TZs can be classified into two broad types: type I (short proximal TZ) represented by Fig. 1A-D, and found in basal Fungi as well as many Protozoa and harosan Chromista, in which TP is very close to the upper end of the centriole but usually significantly separated from it; and type II (longer proximal TZ) with much greater separation of TP and centriole, represented by animals (Fig. 1F), their choanoflagellate relatives, most hacrobian chromists, green plants and several derived groups of Protozoa. More recent reviews of TZ diversity are Grain et al. (1988), Karpov and Fokin (1995), and Fisch and Dupuis-Williams (2011). Type I and II have several major variants, characteristic of specific lineages, three shown in Fig.1 for type I (B/C and D having a longer TZ distal to TP with contrasting extra structures absent primitively in A). I shall argue that an even simpler variant of type I than Fig. 1A is probably ancestral for all eukaryotes and that type II variants evolved independently in several derived lineages of Protozoa, Fungi, Chromista, and Plantae.

Despite such dramatic variations in ultrastructure, all TZ doublets lack both dynein arms and radial inward pointing spokes, but instead have outward-pointing radial projections, originally called 'doublet outer projections' (Cavalier Smith 1967; Cavalier-Smith 1974), which link doublets firmly to the ciliary membrane (Fig. 1B, E). Gilula and Satir (1972) showed that these linkers adjoin intramembrane protein particles arranged within the ciliary membrane as a ciliary necklace and poetically called them 'champagne glass extensions', but they are now widely called Y-links, because of their appearance in cross section (Fig. 1F). It is now usual to define the TZ as the proximal part of the ciliary axoneme immediately distal to the centriolar C tubule ending, and which bears Y-links instead of dynein arms and radial spokes (Reiter et al. 2012; Garcia-Gonzalo and Reiter 2017). Four Y-link proteins have now been mapped three dimensionally in great detail by supermicroscopy (Shi et al. 2017). Almost all TZs also have a conspicuous dense transverse plate, which in conjunction with Y-links and their associated proteins is a passive barrier to diffusion of larger molecules or vesicles into the cilium, thereby maintaining it as a separate compartment with different chemical composition from the cytoplasm. That is the universal function of the TZ irrespective of its marked evolutionary variation in other ultrastructural aspects and of whether cilia are motile or not or have a cp or lack it as do immotile sensory 9+0 cilia of animals. Some TZ proteins are conserved across all eukaryotes from trypanosomes to animals and some are lineage specific (Hodges et al. 2010; Dean et al. 2016).

It is clear from comparative anatomy of protist TZs, yet insufficiently recognised, that the transverse plate consists of two developmentally and evolutionarily distinct structures: a central dense plate attached inside the doublet ring, here called the *transitional plate* (**TP**), and a peripheral annular connexion (**ac**; Cavalier-Smith 1974) that links doublets to the ciliary membrane and is typically associated with a ciliary membrane constriction—and likely the mechanical cause of that constriction. In animals, but only a few protists, e.g., *Telonema* (Yabuki et al. 2013a, 2013b), TP and ac are at the same level and have been lumped as a single structure (Fig. 1F), but in most protists, e.g., typical heterokont chromists (Fig. 1C), ac is offset slightly distally from TP (Fig. 1C). Ac may also be called the dense collar (Cavalier-Smith 2014, who argued that ac and TP both date back to the ancestral cilium). As first shown for the glaucophyte *Cyanophora paradoxa* (Mignot et al. 1969), distal offset of ac (which they called 'un septum annulaire' or annular septum, being unaware of the earlier name 'annular connection', as originally spelt: Cavalier Smith 1967) can be greater even than the diameter of a cilium (Fig. 1B). I show below that this great offset is true of all glaucophytes, *Rhodelphis* (Fig. 1A), and *Picomonas*, and thus of all Biliphyta, and only of Biliphyta; and that much of the diversity of protist TZs is best interpreted as similar positional changes in a basic conserved set of structures and in their being hypertrophied, reduced, or obscured by secondary material differentially across lineages.

Another widely overlooked feature of TZs is that (instead of dynein arms) robust linkers between A and B tubules of adjacent doublets, here called A-B links, are *invariably* present at some level, often throughout the major part of the TZ. I discuss these in relation to the evolutionary origin of TPs and the primary split of discaria into two huge clades: *dorsates*, which include animals, fungi, and revised protozoan subkingdom Sarcomastigota and *natates* comprising plants, chromists and new protozoan subkingdom Natozoa. I shall argue that natates ancestrally swam by ciliary undulation, whereas dorsates instead ancestrally glided on surfaces by ciliary surface motility of their posterior cilia, and this difference may have been associated with the origin of a second set of A-B links in ancestral natates (hower the dorsate *Sulcomonas* also has two sets, which complicates interpretation).

When TZ proteins mutate some cause serious diseases (ciliopathies) in animals, ranging from blindness to severe kidney disease. Evolutionarily and developmentally, immotile 9+0 animal sensory cilia are essentially hypertrophied highly elongated TZs that never switched over to 9+2 morphogenesis at their distal end. Comparative studies of animal 9+0 sensory cilia and 9+2 motile ones of genetically amenable model protist *Chlamydomonas reinhardtii*, *Tetrahymena/Paramecium*, and *Trypanosoma brucei* are revealing TZ functions and principles underlying TZ architecture (Dean et al. 2016; Kilburn et al. 2007). Y-links and associated protein complexes provide the diffusion barrier and sites for docking machinery involved in early ciliary development and opening gates to allow entry and exit of correct molecules to the ciliary compartment. The ciliary necklace and likely associated septin proteins make a similar barrier within the base of the ciliary membrane that allows it to have a different lipid and protein composition from the plasma membrane. Transitional fibres (TF) that attach centrioles distally to the plasma membrane to allow ciliary growth are also docking sites for intraciliary transport particles (IFTs) which recognise cargo such as axoneme precursors that needs to be carried actively into, along, or out of the ciliary compartment during development (Wingfield and Lechtreck 2018). Separate kinesin-driven anterograde IFTs travel up doublet B tubules and retrograde dynein-driven IFTs down A tubules (Stepanek and Pigino 2016). Though some macromolecular complexes associated with Y-links, notably BBsome and MKS complexes, are near universal in eukaryotes and are becoming much better defined, proteins responsible for the fundamental structure of the necklace and Y-links are less well identified. The TZ membrane differs in lipids and proteins from the main ciliary shaft and the plasma membrane; as cilia of many protists autotomize at the TZ site, which depends on Ca^*++*^, transmembrane ion channels are likely general in TZ.

A few protists apparently lack Y-links and/or MKS complexes (functionally associated), notably *Giardia* and Sporozoa (*Plasmodium*, *Toxoplasma* with cilia only briefly present in gametes) (Barker et al. 2014). Absence in *Giardia* is an unsurprising secondary loss as unlike other metamonads the basal part of axonemes are free in the cytosol not bounded by membrane (including the short TZ with standard TP; the speculation by Barker et al. that TZ may be absent is incorrect), so there is no longer a need for Y-links to a membrane, and IFTs can access the axoneme laterally directly from the cytosol. *Plasmodium* may add its ciliary proteins by an IFT-independent mechanism as genomes lack identifiable IFT homologues–lost, or drastically mutated beyond recognition. Thus A-B links are more fundamental than Y-links for defining TZs.

In kingdom Plantae, Viridiplantae have an exceptionally complex TZ. In addition to Y-links throughout the TZ, a central TP connects the doublets; in *Chlamydomonas* and relatives there are two distinct annular connexions, not just one as in most other eukaryotes. Below and above TP are two basal cylinders (distal and proximal) each connected to A tubules by a characteristic stellate structure (Fig. 3). Though varying somewhat in different lineages (Melkonian 1984), the ancestral green plant is inferred to have had a TZ essentially like that of *Chlamydomonas*. Bryophytes and pteridophytes, plants with cilia only on sperm, lost MKS (and supposedly also Y-links) and BBsomes (and various other ancestral ciliary proteins: Hodges et al. 2012; Barker et al. 2014), so ciliary protein targeting during spermatogenesis must have been modified analogously to and independently of sporozoan ciliogenesis.

The nature of the boundary between TZ and centriole was clarified by discovery in *Chlamydomonas reinhardtii* detergent-extracted cytoskeletons of a rotationally asymmetric structure at the distal apex of the centriole (Geimer and Melkonian 2004): the acorn-V filament system. This was proposed to provide information for the non-rotationally symmetric attachment of ancillary structures, notably centriolar roots, in all eukaryotes. That is supported by protein VFL1, located asymmetrically at the outer acorn filament, pleiotropically disturbing the number and position of cilia when mutated (Silflow et al. 2001). Acorn-V filaments are present at the proximal end of the TZ immediately distal to where procentriole triplets start, so are fundamentally TZ structures, not centriolar, but are present in 'procentrioles'. Geimer and Melkonian (2004) found literature evidence for related structures also in a chytrid fungus and the metamonad flagellate protozoan *Pseudotrichonympha*, so suggested all eukaryotes may have them. I have found extensive overlooked published evidence that acorn filaments are indeed present extremely widely in eukaryotes and give further details below. Geimer and Melkonian (2004) showed that the V-filament appears as a Y at one level and V at another and later found that V-filament components and the central filament linking them to the *Chlamydomonas* TP contain centrin (Geimer and Melkonian 2005) and that the centrin filaments are tilted, not strictly transverse. Whilst the centriole/TZ boundary is often assumed to lie in a single transverse plane, McNitt's (1974) serial sectioning showed the zoospore centriole of the fungus *Phlyctochytrium irregulare* (which Geimer and Melkonian considered to have an acorn-like structure) to be chamfered distally so doublets extend further upwards on one side than on the other. After examining micrographs of scores of species across eukaryotes during this work I conclude that this unequal distal extension of C tubules may be true of many, perhaps most, eukaryotes, but probably not all.

I show below acorn-V presence also in developing centrioles of ciliates and in the short barren centrioles of fungal zoospores and argue that this has overlooked implications for the mechanism of centriole growth. Simultaneously clarifying the comparative anatomy of the TZ and centriole-abutting acorn-V is important because of many past confusions between centriolar and TZ transverse plates, calling different structures by the same name and the same structure by different names, which has impeded understanding their development and evolution. I correct some of these.

I also show for the first time that in malawimonad protozoa the acorn system is exceptionally simple and TP is absent, making these flagellates the best candidate for the most divergent eukaryote lineage of all, thus pinpointing the root of the eukaryote tree more confidently than before, in a way closely similar but not identical to the rooted sequence trees of Derelle et al. (2015) using 37 or 39 genes of eubacterial origin, but virtually identical to their site-heterogeneous trees after excluding the 10 most divergent proteins. This illuminates the TZ's evolutionary origin and early diversification.

## Introduction: the TZ hub-lattice and nonagonal fibre

**Fig. 4.**
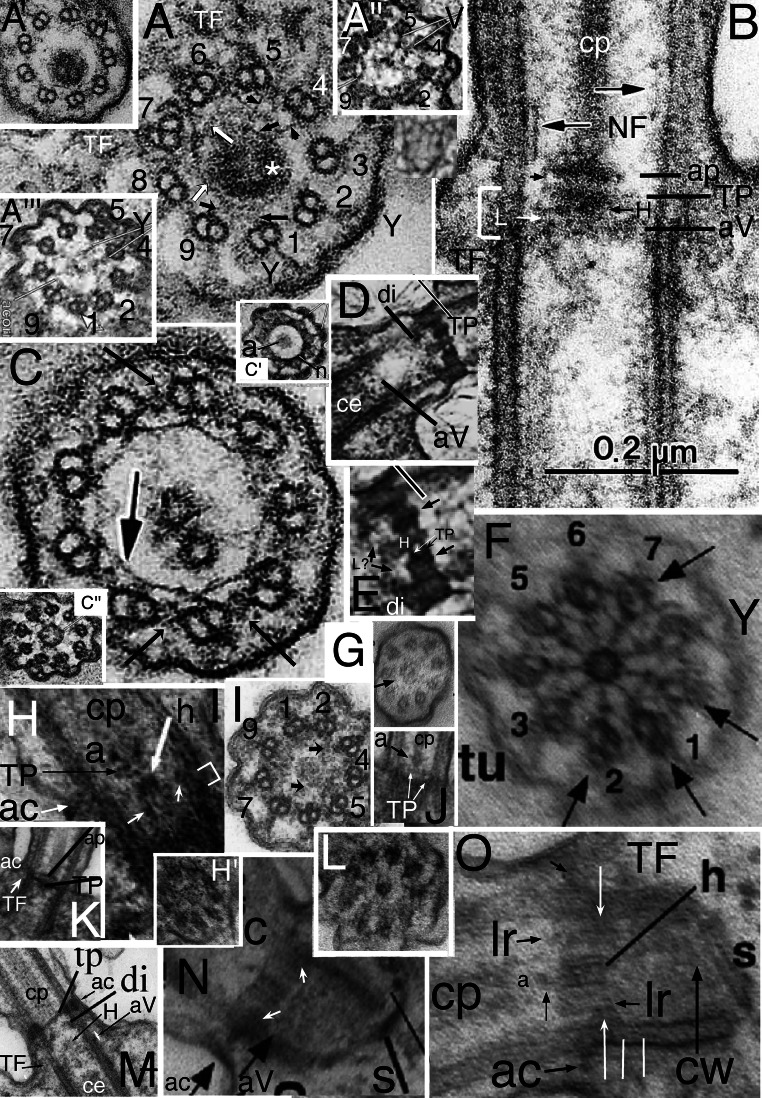
Cercozoan TZ hub-lattice and nonagonal fibres. **A.** Transverse slice through TZ/centriole junction of *Bigelowiella natans* showing superimposed hub-lattice structure and acorn-V filament system; doublets numbered following Geimer and Melkonian (2004) assuming that the slender filament just outside the more prominent circumferential filament (white arrows) surrounding the central density (likely the dense TP centre labelled in **B**) is the acorn filament (black arrows). Asterisk marks the hub densities. The more obvious peripheral lattice is best seen between doublets 4-8. **Y** = Y-links. **TF** = transitional fibres. **A'**. transverse section (TS) (serial section immediately distal to **A** and proximal to **C)** includes the axosomal plate and cp base and grazes some peripheral filaments. **A'', A'''.***Viridiraptor invadens* from Hess and Melkonian (2014 Figs 6C,D) by permission. **A''** TS of acorn-V complex, **A'''** slightly more distal thus including acorn-V plus parts of the proximal lattice between doublets 4-8 like that in **A**. **A**^**iv**^ TS of proximal hub (= central ring). **B.** Longitudinal section (LS) through *Bigelowiella natans* TZ. The bracket embraces the transition plate (TP) and acorn-V system (located at the mid level of the transitional fibres, TF) that are both *partially* included in **A**; the proximal hub (H) and lattice (L, exceedingly thin) are tightly sandwiched between them. The axosomal plate (**ap**) terminates the central pair (**cp**) microtubules. The axosomal plate (**ap**) has a bounding filament (smallest arrow) that begins at the base of the nonagonal filament (NF) that begins just below the end of **cp**. **ap** and the major thickening of **TP** are eccentric and linked by less dense material. **C.** TS of *Bigelowiella natans* distal TZ through the nonagonal fibre (large arrow). A-B links (small arrows) are double, each part oppositely kinked to give a diamond profile. A', A-C, from Moestrup and Sengco (2001 Figs 6B-D, I) by permission.) **C'**, **C''** *Viridiraptor invadens* from Hess and Melkonian (2014 Figs 5C_2_, E) by permission; **C'** TZ at level of axosomal plate (**a**) and nonagonal tube (n); **C''** at level of proximal hub (='central ring') and its spokes. **D.** TZ of *Metromonas simplex* long posterior cilium showing thick **TP** and proximal diaphragm (**di**) well separated from the acorn-V (**aV**); enlargement (**E**) shows filaments (**L**) linking **di** to broad end of hub (**H**); arrows mark the protruding lateral rods. **(**D/E from Mylnikova and Mylinkov (2011) by permission.) **F.** *Sainouron acronematica* TS of TZ hub-lattice of posterior ciliary TZ. **tu** = upper TF. Arrows indicate extra crescentic structures on certain doublets. **G**. Section immediately distal to the dense hub-spoke in **F**, showing faint central granule, surrounding starfish-like structure (like the axosome in **C'**, so represents the axosome best seen in LS in Fig. 5 upper insert of Cavalier-Smith et al. 2008) and barely visible nonagonal fibre (arrow). **H.** *Helkesimastix marina* LS through posterior centriole and TZ; arrow indicates the short hub of the hub-lattice structure, proximal to the cp axosome (**a**); bracket and small arrows show likely position of acorn-V complex. **H'** TS of *H. marina* short cilium (lacking cp) shows TP lattice more clearly than in **L**. **I.** *Katabia gromovi* TS of most proximal TZ possibly grazing centriolar acorn lumenal filament (small arrows) numbered after Geimer and Melkonian (2004) (From Karpov et al. (2003a, b Fig. 49) by permission)**. J, K.** *Massisteria voersi* TZs with axosome (**a**) and axosomal plate (**ap**) above TP*.*(J, K from Mylnikov et al. 2015. Figs 11, 9 by permission.) **L.** *Helkesimastix marina* long cilium TS straddling cp (only 1 mt at this level; see text) and TP junction. **M.** *Katabia gromovi* TZ LS from Karpov et al. (2003a Fig. 27) by permission. **cp** is directly attached to **tp**. **H**=hub. **aV**= putative acorn-V. **di**=TZ diaphragm, **not** the same as the centriole structure that Karpov et al. (2003a) also labelled diaphragm in their Fig. 49. **N.** *Sainouron acronematica* LS of posterior ciliary TZ and chamfered centriole also showing putative acorn-V filaments immediately proximal to the thick hub-spoke structure; ac is in line with the latter's mid point. **s** = spiral fibre; arrows mark TP. **O.** *Sainouron acronematica* LS of posterior ciliary TZ and centriole. **h**=distal hub of hub-lattice. **TF**= lower transitional fibre **ac** marks the position of the annular connector in typical eukaryotes with longer TZ, which in *Sainouron* in **F** was called **tu** as it is a discrete fibre not obscured by dense matrix as usual. Small arrow shows central connector between fainter **cp** axosome (**a**); **lr**= projecting lateral rods. White arrows indicate triangular section peripheral thickening of TP. White lines mark likely thickness of acorn-V complex. (F, G, N, O from Cavalier-Smith et al. (2008a Figs 4e, f, b; 3f); H, H' L from Cavalier-Smith et al. (2009 Figs 4C, D, 5E) by permission.)

Cercozoan helkesid flagellate's TZs are shorter than in any other eukaryotes except malawimonads and radically simplified compared with standard ancestral short TZs with a dense TP, e.g., Fig. 1A, C. Helkesid centrioles also are extremely short and chamfered at the base (Fig. 4N, O), the anterior one bearing only a ciliary stub and long posterior cilium is used for gliding. Instead of a dense seemingly amorphous TP as in most eukaryotes, helkesid flagellates (*Sainouron*, *Helkesimastix*, *Cholamonas*) have rotationally symmetric transverse hub-spoke/lattice structures (Fig. 4F) almost immediately above the end of the centriolar C tubule and almost immediately below the base of the cp (Cavalier-Smith et al. 2008a, 2008b, 2009).

The *Sainouron* hub-spoke is thicker than in most other Cercozoa (about 2.5 times thicker than a mt), so most easily seen. Its dense central hub of nine subunits is connected by prominent spokes to the dense granule that terminates the A-tubule inner projections (Fig. 4F). Two slenderer types of filaments contribute also to the peripheral lattice: nine direct links between adjacent granules forming a regular nonagon; and nine slanting connectors between the granules and the sides of adjacent A tubules. There are also separate A-B links; and at least some filaments slanting from the opposite side of the spoke granule that meet the more obvious slanting filament at about 45° thus forming a triangular star point out of phase with the doublets, i.e., pointing to the mid-point of the A-B linkers—clearest between doublets 3 and 4 in Fig. 4F. The hub is slightly wider than a mt; its distal face is linked by a dense granule to a plate-like axosome that terminates the equal-length cp mts (Fig. 4O). The centriole lumen of *Sainouron* is so dense that it is hard to see how its distal end connects with the hub-spokes/lattice, though some micrographs faintly show a just discernible acorn complex in the posterior cilium immediately below the hub-spokes (Cavalier-Smith et al. 2008a Fig. 4b, indicated by the short arrow inadvertently not originally explained in the legend, and 4e lower TS).

In *Helkesimastix* the hub-spoke plate (Fig. 4H) is only about half as thick as in *Sainouron*; being scarcely thicker than a mt makes it harder to see in TS as it occupies only about a third of the thickness of thin sections, so more distal and/or proximal structures necessarily overlay it, yielding hard to interpret superimposed pictures. I now regard the section in Fig. 4L as embracing the TP plus base of the single cp mt attached to it, thus just distal to the hub—not through it as Cavalier-Smith et al. (2009) suggested. The diameter of the central density is too low for the hub shown in LS (Fig. 4H) and the faint lattice surrounding it resembles the TP lattice in the anterior cilium that lacks a cp so more clearly shows the inner slightly denser axosome-like part of its lattice (Fig. 4H'). A little below the *Helkesimastix* hub Fig. 4H appears to show an asymmetric acorn filament system that Cavalier-Smith et al. (2009) saw hints of in their Figs 5D (upper TS) and E (lower TS) but hesitated to mention. A hub-lattice seemed to be present in other Rhizaria wherever TP was not so dense as to make its detection impossible, but had not been observed in any other eukaryotes so was proposed as a shared character unique to Rhizaria.

**Fig. 5.**
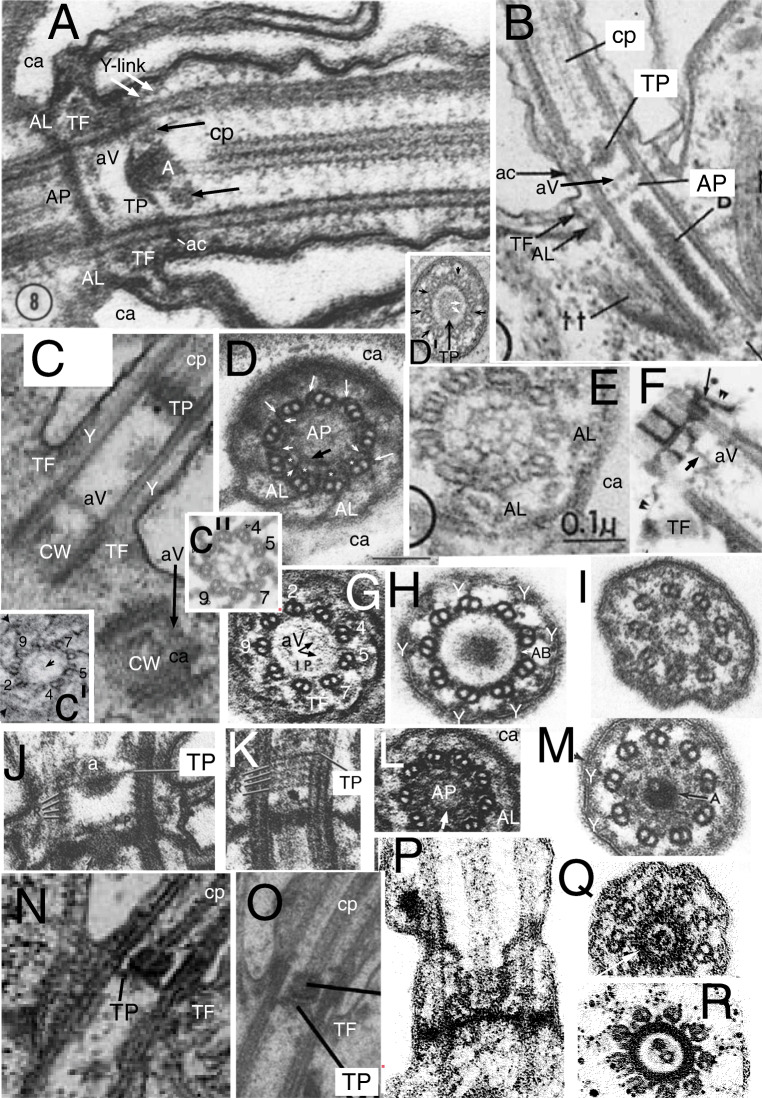
Ciliary TZ and centriolar ultrastructure comparisons in ciliates, relatives, and other model systems. **A.** *Paramecium tetraurelia* TZ in LS; **A** axosome; **ac** annular connection; **AL** alveolar linker; **AP** alveolar plate; **aV**acorn-V system; **ca** cortical alveolus; **cp** centre pair mts; **TF** transition fibre; **TP** transition plate. Arrows mark 'loose ring' distal to TP. **B.** *Tetrahymena pyriformis* TZ in LS. Note that **TP** and **AP** are radially symmetric but differ in substructure, whereas **aY** is radially asymmetric. **C.** *Trypanosoma brucei* (Euglenozoa) type II TZ in LS with long Y-link zone (**Y**) below TP, which is not connected to **aV** (level with **TF**s). Procentriole also capped by an **aV** but still lacks TFs. **CW** cartwheel. **c'***T. bruceii* TS of acorn-V (arrow). **c''** *Chlamydomonas reinhardtii* TS of isolated TZ showing two nested lumenal acorn filaments (more proximal than Fig. 3F). **D.** *Paramecium tetraurelia* centriole TS at level of **AP** and **AL**s grazing circumferential fibre (thick arrow); **ca** cortical alveoli; C tubules (long arrows) incomplete; asterisks mark A-tubule inner projections. short white arrows show radial linkers to A-B links. **E.** *Tetrahymena pyriformis* TS of AP lattice whose spokes point between centriolar triplets. **F.** *Chlamydomonas reinhardtii* LS of isolated TZ showing detergent resistant membrane fragment (double arrowheads) adhering to ac (long arrow) and **TF**s; and asymmetric linker (short arrow) from proximal basal cylinder proximal septum to **aV**. **G.** *Paramecium tetraurelia* TS of TZ grazing top of **aV**. **H.** *Paramecium tetraurelia* TS of TZ grazing bottom of TP, showing Y-links (**Y**) and A-B links (**AB**). **I.** *Paramecium tetraurelia* TS of TZ including the loose ring and/or lateral part of TP lattice and the extended cp mt. **J, K.** *Paramecium tetraurelia* median and tangential LSs of TZ showing four gyres of spiral fibre proximal to TP; **a** axosome. **L.** *Paramecium tetraurelia* TS of AP lattice; circumferential fibre (arrow) more complete than in D. **M.** *Paramecium tetraurelia* TS of peripheral TP surrounding central axosome (**A**) and extreme base of single cp mt. (A, D, G-M from Dute and Kung (1978 Figs 8, 12, 15, 11, 13, 20. 21, 7); B, E from Allen 1969; C from Lacomble et al. (2010 Fig. 2A); c' from Lacomble et al. (2009 Fig. 4A); c'' from Geimer and Melkonian (2005 fig. 4.38); F from Geimer and Melkonian (2004 Fig. 3C) by permission.) **N.** Miozoan alveolate *Colponema* aff. *loxodes* from Mylnikova and Mylnikov (2010 Fig 2b) by permission. **O.** *Colponema vietnamica.* Tikhonenkov et al. (2014 Fig. 4e) by permission. **P, Q** Thraustochytrid heterokont *Schizochytrium aggregatum* from Kazama (1980 Figs 7A,D) by permission. **R.** Heterokont oomycete pseudofungus *Phytophthora parasitica*. Barr and Allan (1985 Fig. 4) by permission.

Interpretation of the cercozoan TZ is revised here in the light of the discovery that *Metromonas*, representing class Metromonadea branching one node below helkesids and one node above chlorarachnids (Cavalier-Smith et al. 2018), has a unique funnel-shaped hub that penetrates through TP (Mylnikova and Mylnikov 2011) and thus has a narrower distal part and wider proximal part of its central hub (Fig. 4D, E). One can now see that non-metromonad cercozoa mostly have only a broader proximal hub-lattice structure below TP *or* a narrower distal hub-*spoke* structure above TP, *not both* as in *Metromonas*. Thus *Sainouron* has a distal hub-spoke complex, whereas Chlorarachnida like *Bigelowiella* have a proximal hub-lattice structure and *Helkesimastix* a proximal hub (possibly with a thin underlying lattice). The spokes and lattice are apparently not homologous, and occur at slightly different levels, whereas the distal and proximal hubs may be indirectly as shown by their continuity in *Metromonas*.

The first unambiguous evidence for an acorn-V in Rhizaria was in the glissomonad cercozoan *Viridiraptor invadens* (Hess and Melkonian 2014 fig 6C, D), as shown in Figs 4A'' (uncomplicated by superimposition) and A''' (complicated by partial superimposition of immediately distal hub-lattice structures overlooked by Hess and Melkonian who thought the lattice was absent in *Viridiraptor*). Strong evidence for Cercozoa having an acorn-V also comes from Fig. 4A of *Bigelowiella*, an early branching cercozoan (Cavalier-Smith et al. 2018) one of 10 excellent serial sections (Moestrup and Sengco 2001) through the ciliary base whose TZ structures are more diverse and slightly more spread out than in helkesids (Fig. 4B). Nonetheless, the Fig. 4A section includes parts of the TP, hub-lattice, and acorn-V structures superimposed. TZ doublets are overlaid on the centriole triplets whose C tubules show faintly for doublets 1, 8, 7. 6, 5, and 4 only (numbered as in Geimer and Melkonian assuming that the broad end of the acorn is attached to extra large granules associated with the A-tubule projections of 1 and 2). The dense central disc diameter of TP is slightly less than that of the acorn, enabling acorn filaments (small black arrows) and thicker circumferential middle filament of the hub lattice (white arrows) to be seen: the extremely slender peripheral filament attaches to triplets 7-9, 1-2 as in *Chlamydomonas* and the lumenal acorn filament (upper arrow) is further away from triplets 3-7 enabling the peripheral lattice to be seen. The inferred acorn-V of *Bigelowiella* appears isomorphic with those of *Viridiraptor* and *Chlamydomonas* showing homology throughout corticates.

Hess and Melkonian (2014) doubted that hubs of *Viridiraptor* (Fig. 4C″) and other cercozoa are all related; but it so closely resembles *Bigelowiella*'s in diameter, structure, and position (immediately distal to an isomorphic slender lattice that is proximal to TP and just distal to the acorn system) that homology of both can scarcely be questioned. But it is not directly homologous with that of *Sainouron*, though an indirect relationship is now evident via metromonads. In particular its angular outline and characteristic inner dense projections into its lumen are distinctive; this plus its greater diameter make it rather distinct from the narrow hub of *Sainouron*, here recognised as distal not proximal; previously I had not identified its TP level so was unable to make this distinction accurately (Cavalier-Smith et al. 2008a, 2008b). Its greater axial length is unsurprising as *Viridiraptor* TZ is highly stretched axially compared with helkesids and chlorarachnids. The deepest branching Cercozoa with known TZ structure are Granofilosea, e.g., *Massisteria* with extremely short type I TZ (Fig. 4J, K) and chlorarachnids. Therefore helkesids have retained the ancestral short compact TZ whilst metromonads and glissomonads independently greatly stretched it by moving TP greatly distal to the acorn-V. In *Viridiraptor* the hub became exclusively proximal and retained its spokes, but in another glissomonad group (*Bodomorpha*, my own unpublished observations) the hub is narrow, distal and has spokes similar to those of *Sainouron*.

This makes it likely that an ancestor of glissomonads (stem sarcomonad) had a tapering hub like that of *Metromonas* with both distal and proximal spokes (a proximal spoke is visible in Fig. 4E). I postulate that the very fine filaments connecting the diaphragm and base of the proximal hub in *Metromonas* are a radially symmetric lattice isomorphic with that of *Bigelowiella* and *Viridiraptor* and that this also was present in the ancestral sarcomonad, being retained by *Viridiraptor* (lacking a distal hub) and lost by *Bodomorpha* (which seems to lack a proximal hub). High resolution study of metromonads, Granofilosea and *Tremula* (the earliest cercozoan branch with no EM) would do much to improve understanding of their early TZ evolution. *Massisteria* appears to have a broad proximal hub (Fig. 4K); Fig. 4J implies that its TP is double and furnished with a tapering embedded hub and protruding lateral rods like that of *Metromonas*, which is therefore likely the ancestral cercozoan pattern, but micrographs are too sparse and fuzzy to show more detail (except that *Massisteria*'s axosome resembles an arrowhead). Nonetheless, I now infer that this ancestral pattern could have generated the *Sainouron* pattern by losing the proximal part of the funnel and the at first sight very different *Viridiraptor* pattern by breaking the broad base of the funnel and its spokes and lattice away from TP and losing the distal part of the funnel, and the *Bodomorpha* arrangement by losing the broad part of the funnel and the lattice and retaining the narrow distal part and its radial spokes.

I also found evidence for a broad proximal hub and closely underlying acorn-V in *Katabia* (Fig. 4I, M) which may be related to glissomonads as its TZ resembles them in being relatively well spread out below a rather clear dense TP (Fig. 4M), i.e., type II like *Metromonas* (Fig. 4D, E), not the type I of early diverging Cercozoa like Chlorarachnea (Fig. 4B) and *Massisteria* (Fig. 4J, K) as well as cercomonads. Though slightly less clear than in *Bigelowiella*, *Katabia* Fig. 4I section appears to include both a faint central hub (proximal to TP; Fig. 4M), hints of a peripheral lattice and an asymmetrically placed lumenal acorn filament (arrows) and densities between it and putative doublets 4, 5 that may represent the V-shaped connectors or other material attached to them. As in *Viridiraptor* the putative extremely slender lattice is sandwiched closely between the hub and acorn-V; though well separated from TP (unlike chlorarachnids, helkesids and Granofilosea) separation is much less than in *Viridiraptor*.

*Bigelowiella* also best shows the nonagonal fibre (NF) attached to A-tubule projections distal to the TP (Fig. 4B, C) in the TZ of both phyla and most classes of Rhizaria. *Viridiraptor* is exceptional in Cercozoa in the long axial extension of its nonagonal fibre (also called inner cylinder), which resembles the nonagonal tube (NT) of Biliphyta discussed below. Axial reduplication of NF so it is below as well as above TP is largely responsible for the extra length of its TZ and from the separation of the proximal hub from TP. As a glider rather than swimmer, its long posterior cilium continually adheres to and is moved along the substratum presumably by membrane-associated kinesin motors, but the basal ciliary zone is not in contact with the substratum, so it is desirable to exclude kinesin gliding motors from it and also to exclude dynein arms and spokes that might disruptively cause active bending. As *Viridiraptor* is a much larger cell (10–20 μm) than other glissomonads it has to be held further from the substratum when gliding, thus needs a longer TZ. The primary function of the extended NT compared with other Cercozoa is therefore probably to exclude arms and spokes (it must directly block spoke binding), but as this cilium has to support the whole bulkier cell off the substratum a longer NT will also usefully increase stiffness and strength of this ciliary zone. The dense outer cylinder that tightly links doublets to the membrane throughout the NT zone would likely prevent normal surface motility in this zone by excluding kinesin, and can be regarded as an axially multiplied dense ac. I argue that suppression of active bending in the basal region of cilia is likely the most basic function of the polyphyletic evolution of longer TZs in most lineages, and shall give other examples. Length control of NT assembly would provide a simple way of defining the extent of the distal TZ.

Hitherto NFs were thought to be restricted to Rhizaria, but I show below that NFs or NTs immediately distal to the TP are characteristic also of Biliphyta in Plantae and even a few Fungi, so may therefore have been present (associated with TP) in the ancestral corticate and discarian. I discuss which elements of the hub/lattice/spoke complex are truly restricted to Cercozoa after considering the other groups.

## Ciliate TZs and acorn-Vs

Before turning to Plantae in detail, I must show that ciliates (superphylum Alveolata), another chromist outgroup to Plantae, also resemble them in having a centriolar acorn-V structure, which has been confused with TPs. Karpov and Fokin (1995) in reviewing TZ diversity noted that in ciliates transverse plates vary from one to four in different subgroups, but are typically three; but did not satisfactorily discriminate between them or establish their homologies with basal ciliary structures in flagellates. Here I focus solely on the models *Paramecium* and *Tetrahymena*, as it is most urgent to put them in sound comparative context.

*Paramecium tetraurelia* has three transverse dense plates. The most distal, called axosomal plate by Dute and Kung (1978), is the only homologue of TP of other eukaryotes, so I call it the transitional plate also. It extends between doublets in the necklace (Y-link) region at the ciliary constriction about 20 nm proximal to the annular connexion just as in Heterokonta (Fig. 1C). Its upper surface bears a dense strongly curved cup that I call the axosomal cup as the dense axosome that surrounds the base of one cp mt only is embedded in it; thus the TP axosome complex is tripartite: TP at the base, the central cup, and distal axosome (Fig. 5A). The second 'intermediate' plate is less dense and unlike the TP clearly lacks radial symmetry in LS and is located immediately above the centriolar C-tubule ending. It is therefore undoubtedly an acorn-V filament system, not a duplicated TP. This identity is confirmed by the presence in TS of an asymmetric lumenal filament (Fig. 5G) with densities between it and doublets 4/5 representing the V). The third plate is thicker and uniformly dense and clearly in the triplet zone, thus centriolar not transitional (Fig. 5A). Though usually called a 'terminal plate', I rename it the alveolar plate (AP) for three reasons: it is level with the dense fibres that connect ciliate centrioles to the edges of the immediately adjacent cortical alveoli (Fig. 5A); its substructure appears unique to ciliates; and nonhomologous structures (some of them acorn-Vs) have also confusingly been called terminal plates in other protists. Its linkers (resembling and originally confused with TFs that connect centrioles to the plasma membrane) are structurally and positionally distinct, so I call them alveolar links (AL). I propose that AP functions specifically to stabilise and further strengthen the link between centrioles and surrounding alveoli and thus make the ciliate cortex more rigid. This function explains why AP and AL are both absent in the sister alveolate phylum Miozoa that typically has only two cilia not associated with cortical alveoli since miozoan alveoli are differently arranged in the absence of kineties (regular rows of cilia). At the level of AP the *Paramecium* C tubules are incomplete at their inner ends, resembling projecting hooks (Fig. 5D, L), whereas *Tetrahymena* has complete triplets. Chinese hamster ovary centriole distal C tubules are incomplete (Greenan et al. 2018) in the same way as the *Paramecium* centriole, but such incompleteness is probably rare in protists.

This third plate in *Tetrahymena pyriformis* (Allen 1969) appears thinner and less dense than in *Paramecium* enabling its ultrastructure to be seen in TS as a novel hub-spoke structure (Fig. 5E). Unlike the cercozoan TZ hub this centriolar hub does not consist of solid granules but of nine open cells with solid walls but empty lumen; each spoke is not opposite A tubules as in cercozoan hub-spokes but in line with the A-C linkers between triplets; it branches as it approaches the linker and joins a peripheral lattice on the inner face of the triplet cylinder. In *Paramecium tetraurelia* the AP peripheral lattice appears to have essentially the same structure despite being more obscured by the denser amorphous matrix, but the central part of the hub is so homogeneously dense that one cannot clearly see its cellular substructure, though there are hints of it being fundamentally the same as in *Tetrahymena* (Fig. 5L). As *Paramecium* and *Tetrahymena* belong respectively to subclasses Peniculia and Hymenostomatia of class Oligohymenophorea, APs with homologous filamentary substructure likely occur throughout the class. I suggest that they arose in the last common ancestor of all ciliates when regular rows of cilia alternating with cortical alveoli first evolved and will thus be found in all other classes of phylum Ciliophora but in no other eukaryotes. I am unaware of any organisms with a similar structure. I also expect APs to be homologous throughout Ciliophora; given their marked difference from the lattice structure of TPs inferred below they cannot simply be duplicated TPs.

*Tetrahymena*'s two 'terminal plates' should not both have been called terminal plates by Allen, as the upper one is a homologue of the acorn-V complex, thus an asymmetric eukaryote-wide structure, but the lower one is the ciliate-specific alveolar plate with a fundamentally different symmetric lattice; they are not homologous. When *Tetrahymena* centrioles first develop they have only the capping acorn-V, formed even before they grow to full length and dock onto the cell surface (Allen 1969 Fig. 7); AP is added later after the development of ALs, which apparently mediate docking onto the cell surface (my new interpretations of Allen's micrographs). The *Tetrahymena* axosomal complex is similar to *Paramecium* and also connects to just one cp mt, as in ciliates generally—I shall show that this cp asymmetry was ancestrally present in Miozoa (sister phylum to ciliates) and heterokonts the sisters of Alveolata, thus is an ancestral character for their joint clade. Thus TZ and centriole structure are fundamentally similar in *Paramecium* and *Tetrahymena*, but share only the TP and acorn-V 'plate' with other eukaryotes.

The axosome cup appears to consist of a roughly circular filament less dense than the central axosome itself, which must really be a sheet of filaments. The TP is not homogeneous but appears to consist of a fine mesh of filaments (Fig. 5H). This supports my thesis of a filamentous core skeleton for TPs, to which I shall return after showing similar figures for Plantae and other groups. For now the key point it that TP filamentary substructure is different both from the radially symmetric AP and asymmetric acorn-V, so all three ciliate plates fundamentally differ and are not homologous duplicates of the same structure, not previously established.

*Paramecium* TZ has two other less general structures. A ring of dense granules immediately above TP (Fig. 5A, I) appear to be connected to A-tubule projections similarly to the gyres of the heterokont TH. Dute and Kung (1978) called them a 'loose ring'; Karpov and Fokin (1995) noted the presence of this distal ring in eight *Paramecium* species and a few other ciliates in two subclasses of Oligohymenophorea, but they seem absent in *Tetrahymena* and most ciliate classes. Proximally to TP *Paramecium tetraurelia* TZ has a set of thin rings just inside the doublets, which Karpov and Fokin (1995) interpret as a single helix, analogously to TH (but being proximal to TP it is not homologous with heterokont TH), thus call it a spiral fibre. It lacks obvious homologies with other ciliates or eukaryotes generally, but has some similarity to parts of a sub-TP ring structure in the haptophyte *Pleurochrysis* (Inouye and Pienaar 1985) and a few fungi. I shall show that there are still more striking previously unrecognised fundamental similarities amongst the TZ of ciliates, other alveolates, and heterokonts that add ultrastructural support to alveolates and heterokonts being sister groups and rightly classified together as chromist infrakingdom Halvaria (Cavalier-Smith 2010, 2013, 2018). I discuss these similarities after treating the TZ of Plantae and other protists.

**Fig. 6.**
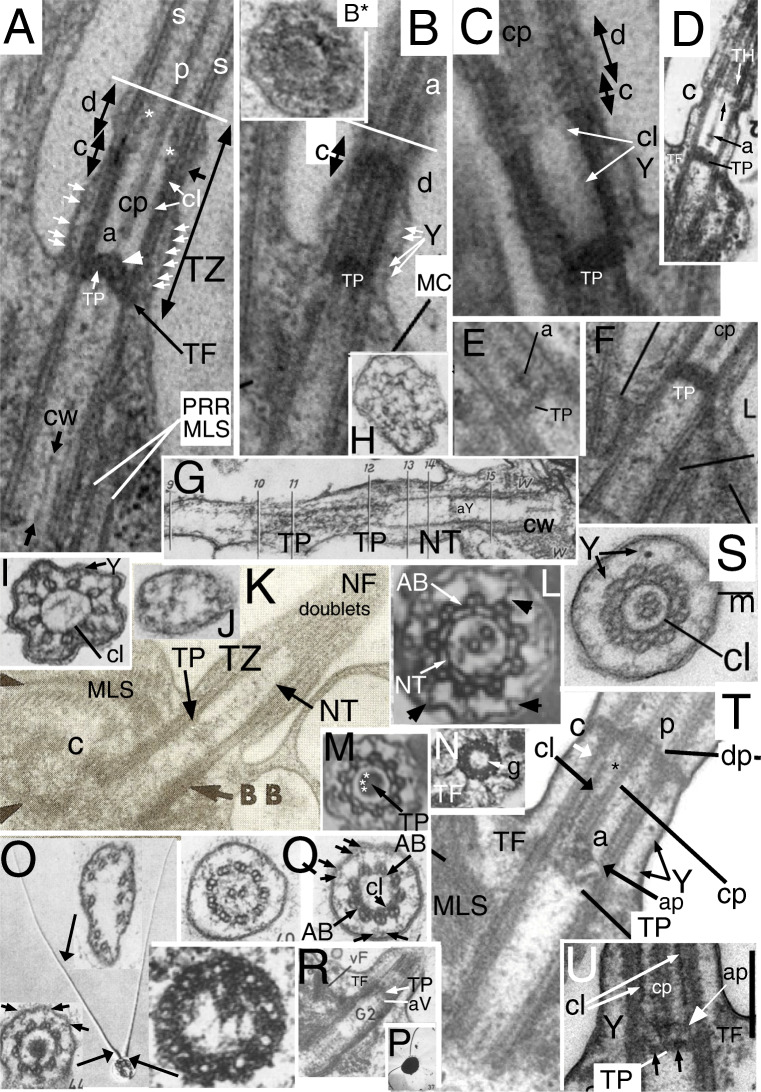
Ciliary transition zones (TZ) of glaucophytes (A-D, F-O) compared with *Rhodelphis* (E, S, T)**. A-C** serial longitudinal sections (LS) through *Cyanophora cuspidata* cilia (from Heiss et al. 2017 Fig. 3A-C by permission). **A** median, **B** lateral section of anterior cilium; its centriole is joined by the multilayered connective (**MC**) to the microtubular base of the multilayer structure (**MLS**) serving as the right root (**PRR**) of the posterior centriole. **A.** The thick transitional plate (**TP**) is concave upwards but in some cells was flat, implying that the slender fibre connecting it to the axosome (**a**) at the base of the central pair (**cp**) can transmit force from **cp** to distort the flexible **TP**. The TZ has a broader constriction (**c**) than drawn for *C. paradoxa* (Fig. 1B but similar to the actual *C. paradoxa* constriction: **D**); TZ extends from transitional fibres (**TF**) to the transverse white line. Y-links (small white arrows) below **c** join doublets to the ciliary membrane. A slender dense basal cylinder (**cl**) with periodic substructure is on the inner face of the doublets between TP and the short black arrow. In the distal TZ (region **d**) a cylinder of larger granules (asterisks) lies further from the doublets (similarly to **TH** in Fig. 1C). The basal cylinder and **d** region cylinder subunits both differ from the spokes (**s**) in the standard 9+2 region distal to TZ where cp exhibits its standard prominent projections (**p**). **CW** = centriolar cartwheel hub. **B** of the same cilium shows standard dynein arms (a) distal to the white line and Y-links (**Y**) in TZ. B* TS of *C. cuspidata* anterior cilium TP (From Heiss et al. 2017 Fig. 7A by permission). **C.** Median LS of *C. cuspidata* posterior cilium shows the same regional differentiation of TZ substructure (**cl** and **d** region) as the anterior cilium (**A,B**), cylinder (**cl**) substructure more apparent. **D.** *Cyanophora paradoxa* LS of posterior cilium. Axosome (**a**) has more distinct proximal extension than in **A**. Note transition helix (**TH**, overlooked by Mignot et al.) distal to constriction (**c**) and that distal septum (arrow) extends to central pair (**cp**). From Mignot et al. (1969 Fig. 8) by permission. **E.** *Rhodelphis limneticus* TP and double axosome. From Gawryluk et al. (2019 Fig. 1i) by permission. **F.** *Cyanophora cuspidata* TZ LS showing less deformed TP; from Heiss et al. (2017 Fig. 9C) by permission. **G.** *Glaucocystis geitleri* 9+0 pseudocilium in LS showing normal centriole with CW but no cp distal to TP. **H-J** are transverse sections (TSs) at levels 11, 14, 9; **H** through mid-region 11 shows reduplicated TP lattice. **I** through the basal cylinder (**cl**) showing it is a nonagonal tube (NT) attached to A tubules. **J** through distal region (level 9) of *G. nostochinearum* pseudocilia shows 9 outer singlets and probably a rudimentary cp. (G-J from Schnepf 1966 Figs 8, 9, 11, 14, by permission.) **K.** LS of *G. nostochinearum* pseudocilium shows a long TZ with disorganised material only in cp region, but a clear periodic basal nonagonal tube (**NT**); both centrioles (=**BB**) have a proximal cartwheel (**c**). (From Kies 1980 Fig. 14 by permission.) **L-N.** *Cyanoptyche gloeocystis* TZs of motile cilia (from Kies 1989 figs 12, 13, 15, by permission). **L.** Distal TZ around cp: doublets joined by single A-B links are attached by thick radial linkers to cylindrical nonagonal tube (**NT**) and to the membrane by Y-links (arrowheads). **M.** Median TZ section through transitional plate (**TP**), **NT** and Y-links; asterisks mark 9 peripheral densities. **N.** Basal TZ section through TFs close to acorn level showing dense peripheral granules (**g**). **O-R.** *Gloeochaete wittrockiana* from Kies 1976 Figs 12, 13, 15, 51 by permission. **O.** Interference contrast light micrograph of a separated vegetative cell showing long non-motile pseudocilia without cps plus electron microscope transverse sections at three levels: bottom left, through doublets within the thick base where central dense material replaces cp; upper, median of the long, thin acroneme where B tubules are absent and A tubules remain linked to the membrane; bottom right, basal section though transition from centriolar triplets (left) to TZ doublets (right) that includes the (?partially disorganised) radially asymmetric acorn-V structure of Geimer and Melkonian (2004). **P.** Chromium-shadowed electron micrograph of zoospore with motile cilia**. Q.** TS through motile 9+2 axoneme with cp (left) and TZ basal cylinder (**cl**) and Y links (right). Small arrows mark the dense granules at the membrane ends of the Y-link arms seen also in *Chlamydomonas* (Fig. 2). **R.** LS showing **TP** and putative acorn-V (**aV**); **vF** = striated connection between centrioles. **S-U.** *Rhodelphis limneticus* TZs from Gawryluk et al. (2019 Figs 1q,r and extended data Fig. 1e) by permission: **S.** TS showing Y-links from doublet partitions to membrane (**m**); the basal cylinder (**cl**) is a NT linked to A tubules. **T.** *Rhodelphis* ciliary LS showing standard cp projections (**p**) where **cp** penetrates the distal plate (**dp**, just above the constriction **c**). The white arrow marks where TZ structure changes from proximal dense cylinder (**cl**) near the doublets to a slenderer structure (asterisk) nearer cp. The axosomal plate (**ap)** central knob is distinct both from the axosome (**a**) and **TP**. TP and ap both deformed, unlike in **E, U**. **Y**= Y-links. The thick centriolar root is likely a **MLS**. **U.** TP region of posterior cilium; unlike in **P** TP and cp are undistorted; ap (white arrow) is more septum-like than in **E** making plates seem double. A central hub (right of the asterisk) connects the cp base to TP

Before doing so I must point out that terminology of TZ and centriolar plates in the model system *Trypanosoma* is also confusing. Molecular specialists (e.g., Dean et al. 2016) tend to use a nomenclature similar to but contradictory with ciliatologists, calling the upper dense TZ plate the basal plate (rather confusing as it is basal only to the cp, but neither part of the basal body (=centriole) nor basal to the TZ; they call the distal centriolar plate the terminal plate (abbreviated TP) despite it not being homologous with the 'terminal plate' of *Paramecium* and ciliates generally as used by leading ciliatologists (e.g., Lynn 1981; Lynn and Small 2002), which is actually AP, which is clearly not found in *Trypanosoma* (Fig. 5C). Exactly contrariwise, many morphologists (e.g., Frolov and Karpov 1995, reviewing kinetoplastid ultrastructure generally) use 'terminal plate' (also abbreviated tp) for the subaxosomal plate here called 'transitional plate' (TP) and 'basal plate' for what Dean et al. call the 'terminal plate', which is positionally equivalent to the acorn-V structure. Some studying bodonid kinetoplastids call the distal plate the 'base plate' (bp; e.g., Nikolaev et al. 2003); others use the general term 'transverse plate' (tp) for it (Tikhonenkov et al. 2016, despite sharing one author with the former), or both transverse and transversal in the same paper (Frolov and Karpov 1995) or transitional plate (Andersen et al. 1991, who list other proposed synonyms, some also used contradictorily for different structures). Curiously, Sleigh (1995) despite being coauthor of Andersen et al. (1991) who recommended all to use 'transitional plate' used 'basal plate! As Fig. 5C indicates, the distal plate of the *Trypanosoma* centriole (=basal body) is not only positionally equivalent to the acorn-V, but like it (Fig. 6F of *Chlamydomonas*) is radially asymmetric, and the characteristic acorn-V pattern is visible in low contrast in TS in Fig. 5c'. I therefore argue it actually is an acorn-V filament system and should be called such in *Trypanosoma* as well as in ciliates and *Chlamydomonas*, in order to harmonise terminology amongst the different model systems ultrastructurally and evolutionarily correctly.

Therefore this paper completely avoids the previous, confused terms terminal plate and basal plate. Identifying the trypanosomatid centriole-abutting distal 'plate' as an acorn-V structure means that if the root of the eukaryote tree lies within Eozoa (whether between Euglenozoa and the rest as I once proposed or between Discicristata and the rest, as I favoured more recently (Cavalier-Smith and Chao 2020), the acorn-V structure must be an ancestral structure for all eukaryotes, as Geimer and Melkonian (2004) first suggested. Lynn and Small (2002) called the ciliate acorn-V homologue (=*Paramecium* 'intermediate plate') SP, presumably 'secondary plate', not a useful term as too vague and not relating it to nonciliate homologues. However, my discovery here that the TP/acorn complex is so much simpler in Malawimonada than in any Eozoa or other eukaryotes, now leads me to reject the idea that the root is within Eozoa (Cavalier-Smith 2010) and argue that it is between Malawimonada and all other eukaryotes, now collectively called discaria.

## The glaucophyte TZ in cilia and pseudocilia

TZs are superficially much simpler in glaucophytes than in Viridiplantae, without obvious stellate structures: *Cyanophora* (Mignot et al. 1969; Heiss et al. 2017) and *Cyanoptyche* (Kies 1989) are motile biciliate unicells whose TZ is long (~0.5 μm) and largely distal to TP, which is very close to the TFs (Figs 1B; 6A-C). Heiss et al. (2017) expressed surprise that ciliary ultrastructure of such an evolutionary important group as glaucophytes was so poorly characterised. They studied ciliary roots in detail, correcting previous errors, but though their micrographs provide much evidence concerning the TZ, this is barely mentioned; both they and Mignot et al. (1969) overlooked a fundamental principle of glaucophyte TZ organisation. This is that its long distal zone is divided at the constriction figured in Fig. 1B into two contrasting regions characterised by different structures associated with the outer doublet inner faces. Both groups failed to notice that distal to the constriction *Cyanophora* has a TH similar to that of Heterokonta on the one hand and the primitive viridiplant *Pyramimonas* on the other. This TZ subdivision is evolutionarily important as it exists also in *Rhodelphis* but was similarly entirely overlooked by Gawryluk et al. (2019); I will demonstrate that it was ancestral to all Plantae and explain how it was modified during the origin of Viridiplantae. The constriction where the ciliary membrane is tightly linked to doublets is unusually broad in *Cyanophora*, and has a septum just above its centre through which the cp passes; Mignot et al. (1969) called it the annular septum; their diagram depicts a large central hole, but Fig. 6E shows it extends (albeit faintly stained) centrally right up to cp. Only two other non-glaucophyte eukaryote genera have a similar distal septum surrounding cp: *Rhodelphis* and *Picomonas*. Y-links are present throughout the glaucophyte TZ between TP and septum and in *Cyanopohora* probably also in the distal TH-bearing zone.

Another TZ structure in *Cyanophora cuspidata* is a dense but slender 'basal cylinder' lying just inside the A tubules throughout the zone between the TP and distal septum only, which exhibits a periodic substructure in longitudinal section (LS; Fig. 6A-C, E) and is indistinguishable from the identically positioned 'cylinder' of *Rhodelphis* (Fig. 6P, Q). In transverse section (TS Fig. 6B*) it appears as a beaded ring. It was not evident in *Cyanophora paradoxa*, perhaps owing to fixation only with osmium tetroxide (Mignot et al. 1969), not initially by glutaraldehye as in later studies. No LS was published for *Cyanoptyche* so we cannot say if its distal TZ has an annular septum and different peripheral structures proximally and distally. But in TS the observed structures extending from the TP to past the beginning of the cp apears not as a beaded circular fibre as in *Cyanophora* but as a nonagonal fibre with thicker short links to the A tubules (Fig. 6I, J). In *C. cuspidata* it extends only 80% of the distance to the distal annular connexion.

zhe more distal zone d in Fig. 6A, B, E is occupied by a cylinder of larger periodic granules closer to the axonemal axis. Somewhat tangential sections of both *C. cuspidata* and *C. paradoxa* (Fig. 6D, E) imply that these are not discrete granules but a discontinuous dense coiled fibre or TH. Without LSs for *Cyanoptyche* we do not know if it is distal TZ is similar, though comparison with outgroups below predicts that it is. Below TP and level with the TFs *Cyanoptyche* has a ring of dense granules just inside the doublets (Fig. 6K), probably absent in *Cyanophora*; there is no evidence for a nonagonal substructure or homology with the nonagonal supra-TP 'basal cylinder'. Kies (1989), who first noted these TZ structures above and below TP called both simply electron dense rings. The sub-TP structure (Fig. 6K) can be regarded as a simple ring of granules (? about two projecting inwards from each doublet), but the supra-TP 'basal cylinder' is not a ring but is elongated longitudinally like a cylinder. But as it has flat faces it is not strictly a cylinder (which has circular not polygonal ends), so I shall call it a nonagonal tube (NT; it can also be considered an open-ended nonagonal prism). I argue here for the first time that all glaucophytes have a basal NT of stacked essentially straight slender filaments, whose fundamental structure is obscured in *Cyanophora* by extra matrix and granules that make it thicker and more circular in TS. I further suggest that all glaucophyte motile cilia also have a distal septum and distal TH. I shall argue that the NT, distal septum and TH evolved even earlier and were all present in the last common ancestor of Plantae.

Glaucophyte genera *Glaucocystis* (Schnepf 1966; Kies 1980) and *Gloeochaete* (Kies 1976) have vegetative cells with non-motile pseudocilia that are essentially 9+0, similarly to animal sensory cilia except that they retain the doublets as a ring distally (there is no intrusion of doublets into the centre as in animal and *Leishmania* 9+0 cilia: Gluenz et al. 2010) and these become singlets distally. Glaucophyte pseudocilia are essentially highly modified extended TZs that retain Y-links, TP and NT, but have largely or entirely lost cps. *Glaucocystis* have entire cellulosic walls so pseudocilia trapped within are reduced to short stubs that retain typical TPs and the nonagonal basal tube which retains its structure (Fig. 6F; length in Fig. 6D similar to *Cyanophora*) despite absence of cps in that zone. Distal to unmodified NT in *Glaucocytsis geitleri* is a zone of ~1 μm where the region inside the doublets is filled with filaments and granules having a roughly nine-fold symmetry (Fig. 6D, E) but without any cp. In TS (Fig. 6E) the structure within the doublet ring resembles that of the TS of *Cyanophora* TP (Fig. 6B*) except that the central zone is less dense, so I interpret these as longitudinally manyfold multiplied TP core filaments; because of TP's linear hypertrophy and absence of cp neither dp nor TH is visible, more distal structures than TP generally being suppressed. I return to these structures later after discussing homologues in other groups as their interpretation is critically important for TP evolution. *G. geitleri* apparently lacks a cp altogether; its doublets probably become singlets distally to the hypertrophied zone. *Glaucocystis nostochinearum* retains doublets basally, has no clear cp (Fig. 6H), but has singlets distally (Fig. 6E), which suggests it may sometimes have a vestigial cp.

Uniquely, *Gloeochaete* is vegetatively multicellular with immotile cells embedded in noncellulosic jelly (Fig. 6L) through which immensely long rigid 9+0 pseudocilia protrude. However, its motile zoospores have typical (shorter) 9+2 axonemes with dynein arms and spokes (Fig. 6N) and typical glaucophyte TZ with basal NT and Y-links (Kies 1976). Its pseudocilia have no trace of cps; doublets lack dynein arms or spokes but have Y-links and a TP, which appears single not double thickness as in *Cyanophora*; they appear to have a discrete acorn-V complex (Fig. 6L right inset, 6M), and dense material in the TH zone suggests relics of TH and/or constriction-associated dense matrix. In their mid-zone vegetative cell doublets lose B tubules and nexin links (no longer linked as a rigid hollow cylinder) but largely retain Y-links to the ciliary membrane.

Glaucophyte immotile cilia somewhat resemble 9+0 cilia of 'amastigotes' of the kinetoplastid euglenozoan *Leishmania mexicana* (Gluenz et al. 2010), though *Leishmania* 'amastigotes' differ from *Gloeoechate* but convergently resemble animal 9+0 cilia in that its doublets retain B tubules throughout but about two of them lose Y-links so move into the axoneme core. Therefore the animal/*Leishmania* pattern is not the only way eukaryotes evolve 9+0 immotile cilia. Presumably *L. major* retains doublets for greater rigidity as its rigid cilium serves to attach it to the parasitophorous membrane of its macrophage host (unlike some other *Leishmania* species 'amastigotes' that do not have an extended cilium or use it for attachment: Castro et al. 2006). I suggest that this attachment may be the major function of the *L. mexicana* 'amastigote' cilium, not sensory as suggested by Gluenz et al. (2006), so functional analogy with animal sensors may be misleading. By contrast *Gloeochaete* vegetative cilia project into the water and do not serve as attachments to the substratum, making a sensory function more likely. Their distal singlets imply that doublets are not inherently necessary for sensory function in 9+0 cilia, but may be retained simply in the mechanical construction of animal sensors. It would be more economical to dispense with B tubules if not mechanically necessary, as in *Gloeochaete*. Of course, the *Gloeochaete*, *Leishmania mexicana* and animal rigid cilia all lack dynein arms or spokes, whereas *motile* 9+0 diatom sperm (Manton and von Stosch 1966) have arms but lack spokes (see later) and are thus a third evolutionary variant.

## Rhodelphid and picozoan TZs

**Fig. 7.**
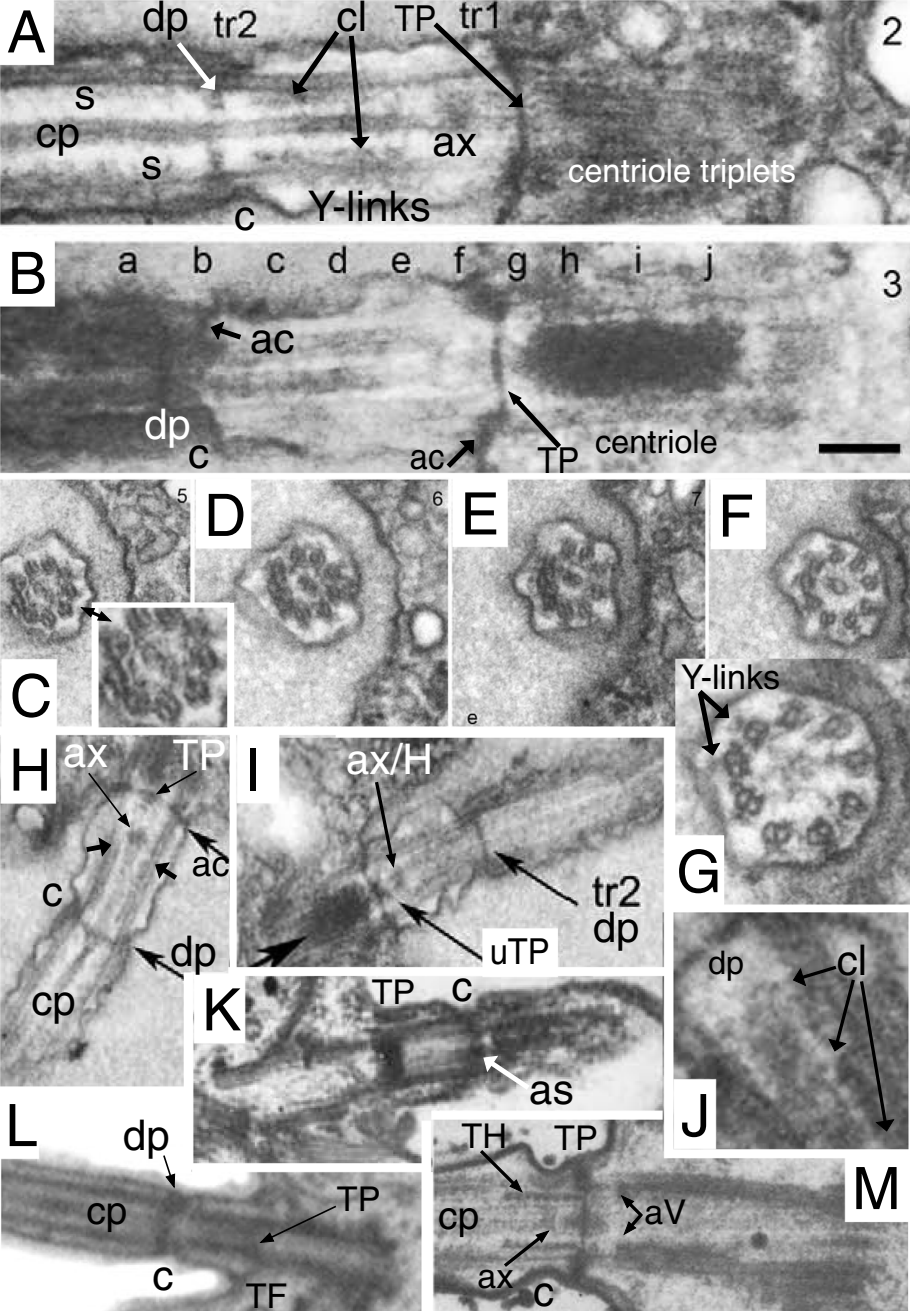
Ciliary transition zone of picozoan/picobiliphyte *Picomonas judraskeda* (A-J) compared with the glaucophyte *Cyanophora* (K), *Rhodelphis* (L) and heterokont *Synura* (M). **A-B.** Slightly and peripheral for centriole, **B** peripheral for axoneme and near median for centriole; both show **TP** (original label **tr1**). A basal cylinder (**cl**) of periodic substructure extends from the central pair (**cp**) axosome (**ax**) region to just below the constriction (**c**). The distal plate (**dp**, originally called **tr2**) extends across the entire axoneme but stains very faintly close to cp as in *Cyanophora* Fig. 6D. Spokes (**s**) are evident above dp. **Y-links** are present between **TP** and **c**. **C-G**. Consecutive TSs through TZ at approximate positions c-g in **B**. **C-E** show nonagonal substructure of the basal cylinder surrounding cp, enlarged in **C** inset. Cylinder and cp are absent in **F**, replaced by a dense central hub and faint peripheral lattice or web of fine fibres connecting it to the doublets; the hub probably represents a structure labelled **ax** in **A**, **H**, **I**. **G** shows transversely sectioned ciliary membrane, so is probably not below **TP** as the g label in **B** implies but just above it; thus it passes through the dense dish-shaped structure marked by the **uTP** (upper TP) arrow in **I**, implying that this central disc has a 9-fold star structure. **H.** *Picomonas* anterior cilium in LS. Distal plate (**dp**) is just distal to constriction **c.** The cp axosome is more substantially distal to and distinct from the **TP**'s central thickening. The basal cylinder extends from just below **c** to just above **ax** (ending at the short arrows). **I.** *Picomonas* posterior cilium in LS. The TP central thickening is bipartite, its upper part (**uTP**) is dish-shaped. The axosome (**ax**) resembles a short dense central hub (**H**). **J.** *Picomonas* anterior cilium in LS; **cl** (note periodic substructure) ends distinctly below **dp**. **A-J** from Seenivasan et al. (2013**A-I** from Fig. 5; **J** from Fig. 2D) by permission. **K.** Glaucophyte *Cyanophora paradoxa* posterior cilium; the anterior septum (as) is marginally distal to the constriction (**c**). From Mignot et al. (1969 Fig. 6), by permission**. L.** *Rhodelphis* cilium; dp is just distal to c. From Gawryluk et al. (2019 Fig. 1F). **M.** *Synura uvella* (chrysomonad heterokont) cilium with transition helix (**TH**) with about 10 gyres immediately distal to TP. The cp-terminating axosome is distinct from the upper and lower central dense TP projections. The constriction (**c**) is opposite the axosome not distal to it as in Biliphyta

Returning to plant TZs, those of *Rhodelphis* (Fig. 1D, 6S-T) and *Picomonas* (Fig. 7A-J) are virtually identical to glaucophytes yet differ from all other protists. Like glaucophyte motile cilia they have a dense septum (ds) or diaphragm traversing the cp well distal to the axosome, which is continuous right up to the cp and its projections, thus probably holds both cp and doublets in a rigid lattice. Throughout this TZ zone all three have Y-links but no dynein arms or radial spokes on the A tubule. Furthermore all three have either a slender nonagonal basal tube (NT) or thicker more granular basal cylinder attached directly to the inner face of the A tubules by short linkers throughout the TZ below ds and above TP (i.e., not via an intermediate stellate structure as is the narrower basal cylinder in Viridiplantae) and by longer slender linkers to the cp. Moreover unlike *Chlamydomonas* and related Viridiplantae which have two separate annular connexions (ac Fig. 3) between doublets and ciliary membrane *Picomonas* at least has only a single ac just distal to TP (Fig. 7A, B, H, I); which may also be true for both *Rhodelphis* (weakly hinted by Gawryluk Fig. 1i and extended data Fig. 2g). I therefore argue that a long distal TZ with single ac, Y-links occupying the whole TZ between axosome and distal septum perforated by the cp, plus a slender NT was present in the last common ancestor of *Rhodelphis* and *Picomonas*, which given the phylogeny of Gawryluk et al. (2019) was also the last common ancestor of these genera plus Rhodophyta. Therefore all three form a clade ancestrally sharing all these characters; which in a later section I formally classify as an infrakingdom named Rhodaria. Even in fine details TZs of rhodaria and glaucophytes are the same. Thus in *Rhodelphis* and *Picomonas* the distal plate is slightly above the centre of the constriction and NT ends a little below the constriction but extends at least down to the axosome but is absent below TP, all exactly as in glaucophytes. Though the 'basal cylinder' was explicitly noted by Gawryluk et al. (2019), it was not recognised as nonagonal rather than truly cylindrical, and its presence also in *Picomonas* was overlooked by them and by Seenivasan et al. (2013) but is shown in Fig. 7A, C, E, H. On this interpretation Rhodophyta lost cilia after diverging from *Rhodelphis*, and *Picomonas* and *Rhodelphis* lost photosynthesis and plastid genomes, but *Rhodelphis* at least retained a plastid as shown genetically but not ultrastructurally by Gawryluk et al. (2019).

No eukaryotes other than glaucophytes, *Rhodelphis*, and *Picomonas* have this type I TZ variant with a distal dense TZ plate surrounding cp and associated with a constriction. This unique TZ character almost certainly evolved in their last common ancestor. If glaucophytes are sisters of Viridiplantae as strongly in 253-protein trees (Gawryluk et al. 2019 Fig. 2a, b) or with 351 proteins (Brown et al. 2018), both using ML or PhyloBayes or ML trees using 187-protein (Cavalier-Smith et al. 2015 Fig. 1), 201 proteins (Janouškovec et al. 2017), or 351-proteins (Lax et al. 2018), then dp evolved in the last common ancestor of Plantae and must have been lost by Viridiplantae when the stellate structures evolved. That conclusion is also true if glaucophytes are instead the deepest branching of the three major plant groups as in the PhyloBayes trees using 258 proteins (Burki et al. 2012) or 187-proteins (Cavalier-Smith et al. 2015 Figs. 2 and 6) or 248 proteins (Strassert et al. 2019), which is arguably more likely considering chloroplast evolution. A comprehensive tree for 42 chloroplast-encoded proteins strongly supports glaucophytes as deepest branching Plantae and strongly excludes their being sisters of Viridiplantae (Figueroa-Martinez et al. 2019), making it likely that Fig. 2A (not 2B) correctly represents the history of Plantae. No recent well sampled multiprotein trees support the third possibility that Biliphyta are a clade, and sister of Viridiplantae, which alone would avoid the necessity for evolving green plant TZ from that demonstrated here as ancestral for Biliphyta. Therefore I conclude that the TZ characters shared by glaucophytes and Rhodaria are most likely ancestral for Plantae and those of Viridiplantae are derived. In that case the ancestral characters shared by Biliphyta (dp and NT) must have been lost and unique viridiplant characters (stellate structures, double basal cylinder and double ac) gained during, before or after the origin of Viridiplantae from Biliphyta.

*Rhodelphis* resembles glaucophytes also in having distinctive TZ structures near the annular septum: the glaucophyte TH has about five thick dense gyres and is closer to the ciliary axis than the basal cylinder/NT. In the same region *Rhodelphis* has a cylindrical structure midway between the doublets and cp that appears zigzag in profile (Fig. 6P, Q). I suggest it is homologous with the glaucophyte TH core skeleton; though in both groups the TH begins immediately distal to the NT and is associated with the constriction (Fig. 6A, T) there are slight differences in the relative axial positions of the TH and dp. In *Cyanophora paradoxa* the TH appears entirely distal to dp, whereas in *C. cuspidata* the precise position of dp is unclear as it may overlap slightly with the TH—if dp is in the same position relative to the broad constriction in both *Cyanophora*, then dp may be represented by the very thin septum in Fig. 6A at the level between the lower asterisk and small arrow in Fig. 6A; if so, then it is at the very base of TH not proximal to it. By contrast in *Rhodelphis limneticus* the denser dp and ac are level with the distal half of the TH (Fig. 6T), thus not proximal to it. Even though somewhat different and not associated with a NT, the heterokont algal TH can also appear in profile as a row of dense blob-like granules as in *Uroglena* or as a thin zigzag as in *Botrydiopsi*s (Hibberd 1979). Therefore the *Rhodelphis* distal cylinder that straddles dp and the largely distal *Cyanophora* equivalent are probably both also THs, which must therefore have been present in the ancestor of Biliphyta and Plantae, but was lost by *Picomonas* with normal spokes distal to dp (Fig. 7A).

## Viridiplant stellate structures and acorn-V

TZ substructure is best resolved in negatively stained partially cell fractionated TZs of *Chlamydomonas*, where 13 individual A mt protofilaments and the globular macromolecular substructure of the star filaments are evident (Fig. 3K). The structures here labelled 'A-tubule feet' consist of about three subunits and the outer nonagonal sheets forming the cylinder wall appear to have two parallel rows of subunits—additional large globules project into the lumen of the basal cylinder. 'A-tubule feet' was originally proposed for the inner projections from centriolar triplets in the region distal to the cartwheel (Cavalier-Smith 1974). However, using isolated detergent-extracted centrioles pretreated with tannic acid, Geimer and Melkonian (2004) showed that these projections are not restricted to the A tubule but extend across the A-B junction. Cryotomography of the homologous distal centriolar projections of mammals revealed their structure in great detail, confirming that they are fixed to *both* sides of the A-B junction (to protofilaments A1, 2 and B10: Greenan et al. 2018). Therefore I rename these centriolar distal inner projections, which appear to be fundamentally similar in *Chlamydomonas* and mammals, A-B feet. By contrast the pinhead-shaped inner projections in the cartwheel region that link A tubules to cartwheel spokes project solely from the protofilament A3/4 junction in both mammals and *Chlamydomonas* (Li et al. 2019), and are now called 'pinheads' (Kitagawa et al. 2011).

**Fig. 8:**
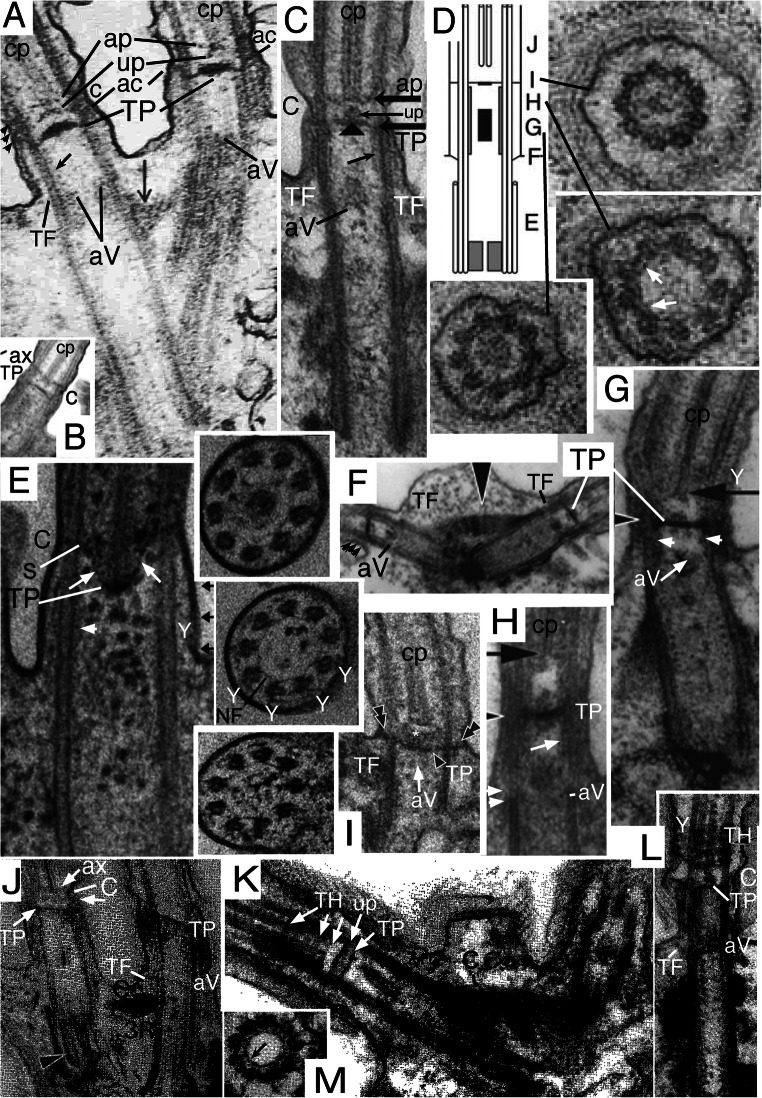
Ciliary transition zones of Cryptista and *Telonema*. A. *Cryptomonas reticulata* From Lucas (1970 Fig. 17B). by permission. Large arrow marks centriolar connection, small arrow A-tubule feet between TP and acorn-V complex (**aV**). Arrowheads show three distinct acs. Note distinct axosomal (**ap**) and upper plates (**up**)**. B.** Cryptophyte *Hemiselmis amylosa* from Clay and Kugrens (1999 fig. 12) by permission. **C.** *Goniomonas avonlea* (**Cryptomonada: Goniomonadea**) From Kim and Archibald (2013 fig. 10A) by permission: arrow marks nonagonal tube. **D.** *Hatena arenicola* (**Cryptista: Leucocryptea**). Diagram of LS plus TSs at levels G, H, I; from Okamoto and Inouye (2006 Fig. 7D, G, H) by permission (arows mark sub-TP nonagonal tube), I inludes TP. **E.** Aberrant goniomonad relative *Hemiarma marina* from Shiratori and Ishida (2016 Fig. 6A, C-E) by permission; LS plus 3 TSs on right at positions shown by black arrows; **s** = TP near its junction with doublets; white arrows show recurved part of TP between **s** and its thicker central disc; arrowhead marks nonagonal fibre seen on middle TS; **C** constriction; **Y** Y-links. **F-H.** *Palpitomonas bilix* (**Cryptista: subphylum Palpitia**) from Yabuki et al. (2010 Figs 4C, 7) by permission. **G.** arrow marks cp base; white arrowheads A-tubule feet (and possibly sub-TP nonagonal tube). **H.** White arrow marks likely sub-TP nonagonal tube. **I.** *Telonema subtilis* (harosan phylum Telonemia) from Yabuki et al. (2013a Fig. 2K) by permission. **J.** *Kathablepharis ovalis* (**Cryptista: Cryptomonada, Leucocryptea**) **C** constriction; arrow level of upper plate. **K--M '***Kathablepharis*' G-2 (likely really a new undescribed genus: see text; J-M from Lee et al. (1992 Figs 26, 27, 29, 30) by permission). **K.** shows exceptional orthogonality of centrioles connected by massive striated connector as in *Palpitomonas.* **L.** LS shows **TH** above constriction (**c**). **M.** TS shows 18-gonal tube (arrow). **ac** annular connexion, **ap** axosomal plate, **aV** putative acorn-V complex, **ax** axosome, **c** constriction, **cp** central pair mts, **TF** transition fibre, **TH** transition helix, **TP** transition plate, **up** upper plate, **Y** Y-links

**Fig. 9.**
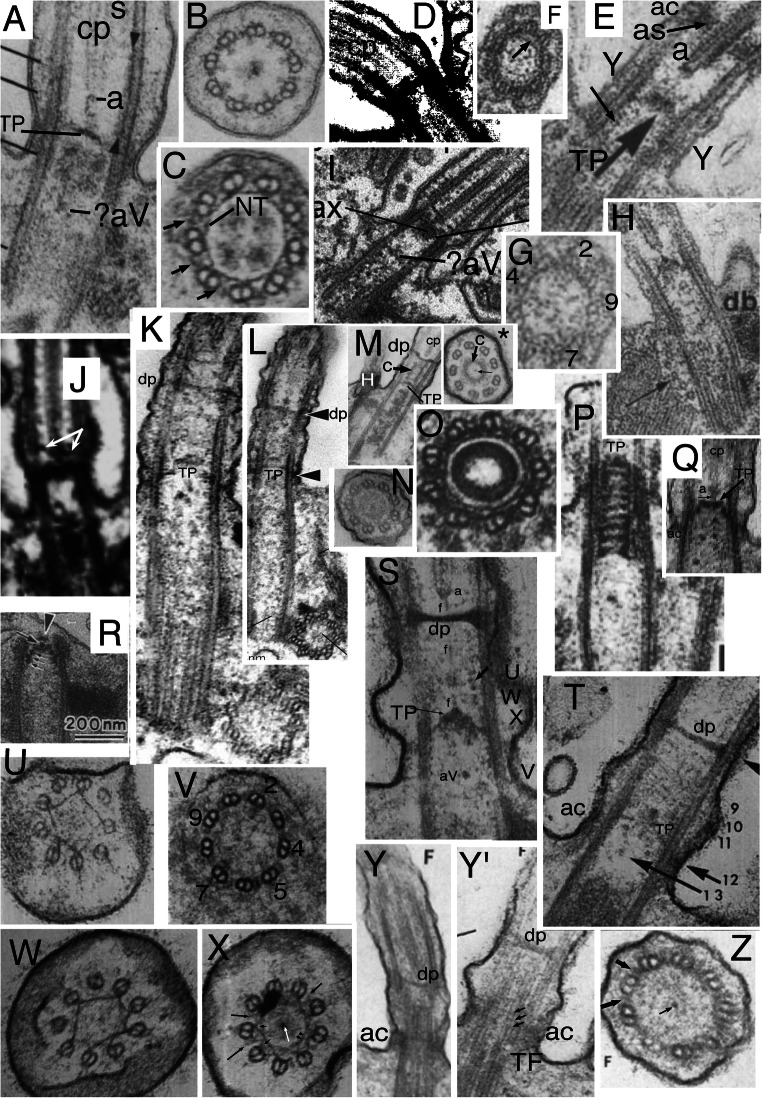
Ciliary transition zones of Haptista. A-D. Pavlovophyceae: **A-C** *Diacronema vlkianum* from Green and Hibberd (1977 Fig. VIF,G,J) by permission. **A.** Bipartite central filament connects axosome (**a**) to TP; distal to TP between arrowheads A tubules have short dense inner projections (likely A-tubule feet *without* extra attached structures) distinct from longer spokes, **s. ?aV** putative aV; cp central pair mts. **B.** TS through central filament showing Y-shaped A-tubule feet. **C.** TS proximal to TP through nonagonal tube (**NT**) and double diamond-shaped A-B links (arrows). **D.** *Pavlova granifera* from Green (1973 Fig. 14) by permission. **E-H.** *Isochrysis galbana* (**Prymnesiophyceae**: Isochrysidales) from Hori and Green (1991 Figs 3B, 8A-C). **E.** LS showing cylindrical axosome (**a**) linked by central filament to central disc of hat-like TP (large arrow), Y-links (**Y**) and tripartite **ac** beside the tripartite annular septum (**as**) perforated by **cp**; small arrow marks A-tubule feet as in **F**. **F.** TS of proximal TZ with A-tubule feet and grazing a NT (arrow). **G.** Section serial to **F** at TZ/centriole junction including the asymmetric acorn-V filaments. **H.** shows central filament more clearly. **I, J. Alveidia: I.** *Ancoracysta twista* from Janouškovec et al. (2017 Fig. S1P) by permission. **J.** *Ancoracysta marisrubri* from Mylnikov (2009 Fig. 3A) as '*Colponema*' by permission; arrows mark cryptomonad-like axosomal plate. **K-Z Prymnesiophyceae: K, L. Prymnesiales:** *Hyalolithus neolepis* from Yoshida et al. (2006 Fig. 8A,B) showing 'top-hat'-like TP and flat distal partition (dp). **M, N. Phaeocystales:** *Phaeocystis poucheti* Parke et al. (1971 Figs 26, 27, 35). **M.** LS showing basal cylinder between TP and distal partition (**dp**). inset * shows basal cylinder in TS linked to doublets by faint A-tubule feet. **N.** TP with irregular lattice central filament and 18 radial links (A-tubule feet and links to A-B links). **O. P. Coccolithales:** *Pleurochrysis* sp. from Inouye and Pienaar (1985 Figs 11, 12). **O.** TZ TS showing spiral fibre attached to A-tubule feet and A-B links with thin radial connectors to inner dense ring. **P.** TZ dense rings in LS. **Q. Coccolithales:** *Calyptrosphaera radiata* from Sym and Kawachi (2000 Fig. 26) by permission; top-hat like **TP** connected by central filament (arrow) to axosome. **R. Coccolithales:** *Cruciplacolithus neohelis* from Kawachi and Inouye (1994 Fig. 5) by permission. **S-Z Prymnesiales: S-X.** *Prymnesium parvum* from Manton (1964 figs 17 18; 9. 10. 13, 14) by permission. **S.** Central filament (**f**) connects axosome (**a**) to TP centre; arrow marks polygonal filaments; **T. U-X** mark approximate positions of TSs **U-X**. **U.** peripheral filaments join alternate A-tubule feet. **V.** TS near base of TZ at level of TFs and putative acorn-V. **W.** peripheral filaments joining alternate A-tubule feet overlap to make pseudo star points. **X.** TS embraces top-hat shaped TP and base of central filament (white arrow); black arrows mark interdoublet star points. **Y, Y', Z.***Chrysochromulina chiton* Manton (1968 Figs 5, 10, 30) by permission. Oblique LSs (Y, Y') show **dp** and tripartite **ac. Z.** TZ TS with central filament (thin arrow) and A-B links (thick arrows)

I have noticed that the great majority of TZ doublet inner projections (possibly all in all groups) are A-only projections, like pinheads; henceforth I shall call such TZ projections A-tubule feet; examples in Cryptista support nonagonal tubes (Fig. 8A) as they do in *Rhodelphis* (Fig. 6S); in Haptista also they support NTs proximally to TP (Fig. 9C) but distally end in simple pinheads only (Fig. 9A, B) without more extensive distal attachments. In principle TZ A-tubule feet might have evolved from the pinhead base; 'feet' connecting doublets to star points in Fig. 3K appear to project from between protofilaments A3 and A4, like the pinhead base. By contrast, axonemal spoke stalks attach to protofilaments A1-3 (Barber et al. 2012), thus are fixed very differently from the TZ A-tubule feet and centriolar pinheads despite their binding sites overlapping and being mutually exclusive. The longitudinal periodicity differs for all three projections, greatest for spokes, least for pinheads. Centriolar A-B feet have a complex distal mass or 'head' that fills much space on the inner surface of triplets connecting one A-B junction to the next (Greenan et al. 2018); they are distinct from TZ A-B links which apparently do not extend to the A-B partition.

**Fig. 10:**
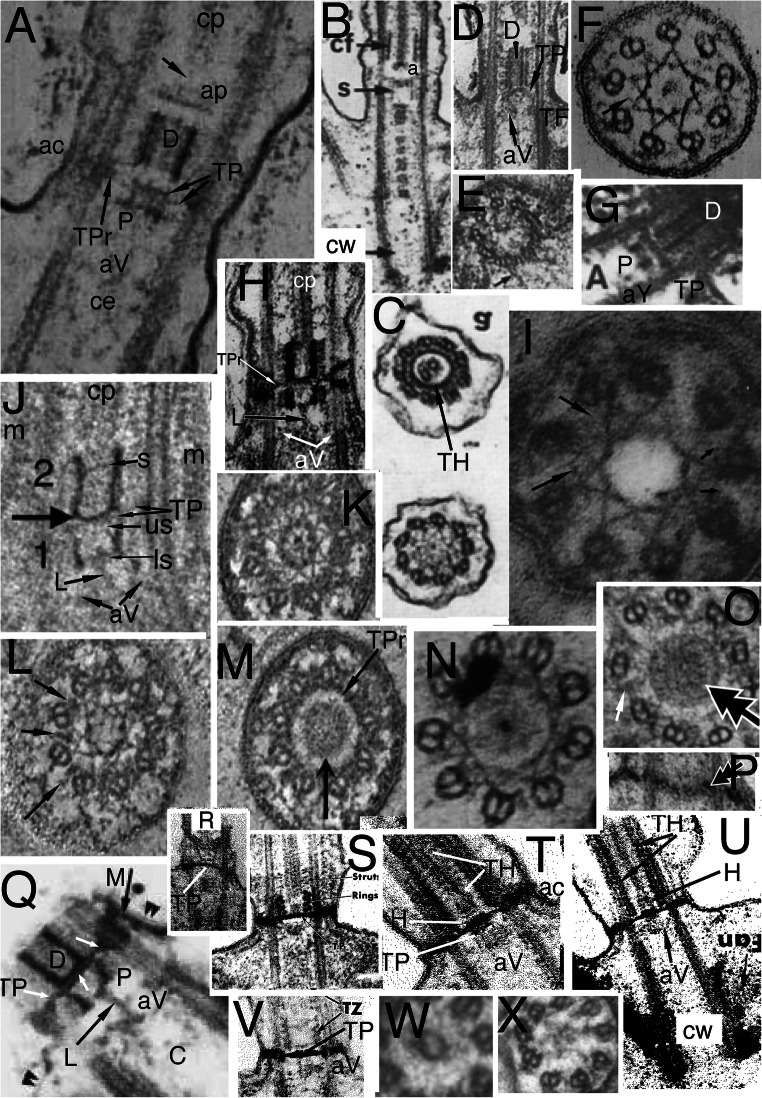
Green plant (A-M) and chromist TZs compared. A. *Nephroselmis* (=*Heteromastix*) *rotunda* (Prasinophytina**:** Nephrophyceae): **cp** is linked to the axosomal plate (**ap**) by central filament (arrow); TP is associated with proximal (P) not distal (D) basal cylinder. **B.** LS, **C.** TS**s** of *Pyramimonas orientalis* (Prasinophytina: Pyramimonadophyceae) **cf** coiled fibre (=TH); **s** stellate structure. **D. E.***Mesostigma viride* (**Charophyta**: Mesostigmatophyceae). **F.***Nephroselmis* (=*Heteromastix*) *rotunda* proximal stellate structure**. G.***Coleochaete pulvinata*(Charophyta) zoospore TZ LS. **H.***Chlamydomonas reinhardtii* (Chlorophyceae: Chlamydomonadales) standard glutaraldehyde plus osmium fixation; **cp** base lodged within distal basal cylinder; **L** linker between proximal basal cylinder lower septum and acorn-V (aV); TP centrally of medium density granules stretched within peripheral ring (TPr) is sandwiched between more amorphous, denser base of distal cylinder and lighter, more alveolate distal septum of proximal cylinder. **I.***Polytoma obtusum* (**Chlorophyceae**: Chlamydomonadales) osmium fixation; TS of proximal stellate structure, long arrows marks intermediate star-point, short ones simpler A-B links. **J-M.***Chlamydomonas reinhardtii* tomographic slices of freeze-substituted wild-type TZ. **J.** LS showing that the 'H cross piece' separating distal and proximal basal cylinders is a trilaminar composite: the base of the longer distal cylinder (large arrow) is denser than the underlying thin **TP**, and the distal septum of the shorter proximal cylinder is alveolate thus lighter still. Note that the proximal lower septum (**ls**, not included in Fig. 3**A** diagram) has a central granule connected by an oblique linker (**L**) to the acorn-V, which is more clearly distinct from the centriole (**c**) in **Q** after detergent extraction that removes centriolar matrix but retains acorn-V. **K.** TS though the upper part of the distal stellate structure; the lattice within the basal cylinder (finer than in **L**, coarser than in **M**) probably represents its distal septum (**s** in **J). L.** upper septum of proximal stellate structure with coarse lattice within the basal cylinder; arrows mark radial interdoublet supports. **M.** Tomogram at level of amorphous/finely latticed TP; TPr TP ring. **N.***Prymnesium parvum*(Haptista) TP showing central filament and peripheral star-points. **O TP** TS, **P TP** LS of *Platysulcus tardus* (Heterokonta, Bigyra). **Q.***Chlamydomonas reinhardtii* LS of isolated TZ showing detergent resistant membrane fragment (M, double arrowheads) adhering to ac (long arrow) and TFs; and asymmetric linker (**L**) from proximal basal cylinder proximal septum to aV. **R-X Heterokonta: R.***Thraustochytrium aureum* TZ (Bigyra: Labyrinthulea). **S-V Pseudofungi**: **S.***Rhizidiomyces apophysatus* (Hyphochytrea). **T-V Oomycetes: T, U.***Phytophthora parasitica*. **V.***Saprolegnia diclina*. **W, X***Picomonas juradskaya* (**Plantae**, Biliphyta). **W.** TZ distal hub, enlargement of Fig. 7F. **X.** TZ upper TP, enlargement of Fig. 7G. A, F, N from Manton (1964 Fig. 11, 20, 21); B, C from Moestrup and Thomsen (1974 Fig. 40, 41); D, E from Melkonian (1989 Figs 11, 23); G from Sluiman (1983 Fig. 26); H from Ringo (1967 Fig. 32); I from Lang 1963 Fig. 2J-M. from O'Toole et al. (2003 Fig. 3C-F), O, P from Shiratori et al. (2015 Fig. 2F, J); Q from Geimer and Melkonian (2004 Fig. 3C); R-U from Barr and Allan (1985 Fig. 3C, 10, 15, 18B, 35); V,W from Seenivasan et al. (2013 Figs 5Bf,g) by permission

Note that early diagrams of the double basal cylinder of *Chlamydomonas reinhardtii* by me (Cavalier Smith 1967; Cavalier-Smith 1974) and Ringo (1967) depicted it as a dense capital H (Fig. 3), with cross-piece of the H corresponding to the TP of typical TZs without a stellate structure, and the proximal basal cylinder being open at the base. In those reconstructions, images like that of Fig. 21 of Ringo (1967) where the proximal basal cylinder also is closed basally by a dense plate equal in thickness and density of its walls were incorrectly ignored. Since discovery of the asymmetric acorn-V filament system at the distal end of the centriole (Geimer and Melkonian 2004) it has been evident (though not previously explicitly stated) that in *Chlamydomonas* the shorter proximal basal cylinder must be closed by a septum at its base. That is because of the presence of a slender filament that links a granule at the centre of this lower septum to the granule beside the vertex of the V-filament (Fig. 3C, H, L; Fig. 5F); there must be a complete or partial lower septum for attachment of this central filament. Thus the basal cylinder complex of Viridiplantae is not really H-like in LS but consists of two cylinders each with a TP-like septum sealing its base. I call the lower usually more indistinct transverse plate the proximal transition plate pTP. The pTP-V-filament connector is not an artefact of detergent extraction used by Geimer and Melkonian (2004) as it is visible (but originally overlooked) in isolated TZ/centriole complexes made purely mechanically, and even more clearly in a few especially well contrasted and fortuitously sectioned intact directly fixed cells, e.g., Fig 32 of Ringo (1967) reproduced in Fig. 10H, and by tomography after freeze substitution (Fig. 3C). Usually however it is more faintly stained than the distal TP. From Fig. 5F/10Q and 10H the distal TP (dTP) is clearly really a complex of two distinct substructures: (a) a lower septum that extends completely across the zone between opposite outer doublets and appears as a single row of dense but discrete granules; (b) an upper central plate that shows no granular substructure but appears as a thin but very dense-staining continuous plate restricted to the zone within the dense walls, which does not exhibit a granular substructure. This composite structure was already recognised and depicted in Fig. 1 of Cavalier-Smith (1974). Fig. 10H shows that pTP is structurally equivalent only to part (a) of dTP as it consists of a row of discrete particles only, which I interpret to run also the entire distance between the doublets. The additional presence of the upper dense continuous layer only in the dTP makes it so much more obvious that uTP was long recognised but pTP widely overlooked. If this interpretation is correct the green plant TZ consists essentially of two separate cylinders, each with a closed base extending laterally as a peripheral flange like a measuring cylinder from a chemistry laboratory. However, this may be an oversimplification as comparative studies show that the upper and lower cylinders and the TP can all evolve and vary independently (see below).

As evident at the outset for the colourless volvocalean *Polytoma uvella* (Lang 1963 Figs. 1, 2, latter reproduced in Fig. 10I) the core structure of each cylinder is not a cylinder but a radially symmetric star with 18 flat sides, each with a central dense (approximately triangular) projection inwards into its lumen. In other words, it is an inner 9-pointed star whose points are less acute than and out of phase with the outer star whose points attach to the A-tubule feet. In *Chlamydomonas* that structure is simplest in the proximal cylinder (Fig. 3E); the distal cylinder has extra diffuse dense material making it and its attached star points thicker and more obvious. The 18 filaments making the outer star points consist largely or partly of centrin, whose Ca^++^-driven contraction causes ciliary autotomy in most green algae (Sanders and Salisbury 1994). I argue that use for autotomy of the 9+2 axoneme when cilia are trapped by a predator or damaged by environmental misfortunes is the functional reason why basal cylinders and stellate structures evolved in the ancestral viridiplant. In *Chlamydomonas*, and no doubt other Volvocales with ciliary tunnels in each cell cycle after ciliary axoneme disassembly (Cavalier-Smith 1974), autotomy also occurs to disconnect the protoplast from the cell wall, a necessary prerequisite of the characteristic 90° protoplast rotation within the cell wall prior to cell division (Cavalier-Smith 1974) by multiple fission (Craigie and Cavalier-Smith 1982; Cross and Umen 2015), which leaves the TZ trapped inside the ciliary tunnels, thus allowing it to be purified and its protein composition established (Diener et al. 2015). A-tubule feet remain unaltered when centrin star filaments collapse into the basal cylinder during Ca^++^-induced contraction (Sanders and Salisbury 1994 Fig. 2), so the feet chemically differ from centrin. I suggest that A-tubule feet linked to the star tips are homologous with and evolved from A-tubule feet linked to the nonagonal tube vertices of glaucophytes and Rhodaria. I further suggest that the short inner projections from the A tubules to which the peripheral acorn and V-filaments are attached are also homologous A-tubule feet (in the new sense proposed here) not necessarily identical to those of the star, and that the acorn-V is fundamentally a TZ structure (the most proximal), not centriolar. A-tubule feet serve as doublet linkers for chromist, protozoan, and fungal NTs and for THs throughout discaria, and in haptophytes also exist as simple pinheads (Fig. 9B). A-tubule feet unambiguously distinguish TZ from spoke-bearing motile axonemes as reliably as do Y-links; both also distinguish TZs from centrioles. A-B feet and pinheads distinguish distal and proximal zones of the centriole.

## Origin of the TZ of Plantae

I have shown that a type I TZ with nearly basal TP and long supra-TP TZ with a distal septum traversing the CP was present in the last common ancestor of Rhodaria and Glaucophyta, and that this type I variant is not found in any other eukaryotes. If as argued above Viridiplantae are sisters of Rhodaria and derived from Biliphyta this TZ pattern must be ancestral for all Plantae. To understand how and from what it may have evolved we need to consider the immediate outgroups of Plantae, currently classified as Chromista. It is universally accepted that chromist subkingdom Harosa (previously comprising Alveolata, Heterokonta, Rhizaria) is a clade and by most specialists that Alveolata and Heterokonta are sisters (collectively infrakingdom Halvaria, a subclade that is sister to infrakingdom Rhizaria; Cavalier-Smith et al. 2018). The other chromist subkingdom Hacrobia (Cryptista, Haptista) is more controversial not because of their ultrastructural characters but because on some multiprotein trees they are a clade (which may branch either as sister to Harosa or as sister to Plantae) and on others Cryptista branch as sisters of Plantae (sometimes weakly even within Plantae, e.g., Strassert et al. 2019, but almost nobody thinks that is correct), in which case Haptista usually are sisters of Harosa instead of Cryptista, making chromists appear paraphyletic. (For discussion of these contradictory trees see Cavalier-Smith et al. 2015, 2018.)

Harosa ancestrally had a type I TZ as this is found in all Heterokonta (with the addition in most of a TH (Fig. 1C), clearly ancestral for Alveolata (characteristic of Ciliophora, Protalveolata, and Apicomplexa; Dinozoa only secondarily have type II; all without TH) and for Rhizaria (found in Phytomyxea within phylum Retaria, and the deepest branching Cercozoa, Chlorarachnea, Granofilosea (Mylnikov et al. 2015), Helkesea, as do Cercomonadida, Paracercomonadida and Marimonadida; some derived lineages have type II, e.g., Metromonadea, Glissomonadida, Eothecia, Thaumatomonadida, Spongomonadida). By contrast Hacrobia all have type II TZs. As this is a derived condition for corticates, if they are indeed a clade as strongly suggested by their shared lateral gene transfer into chloroplasts, then evolution of type II TZ may be a second synapomorphy for Hacrobia, though as it also evolved independently in Dinozoa and more than once in Cercozoa the possibility exists that Cryptista and Haptista did so independently. However the fact that *Telonema* has a type I TZ (Fig. 8I) very similar to (but even shorter than) that of ciliates (Yabuki et al. 2013a, 2013b) is a strong reason for now excluding it from both Cryptista and Hacrobia, as there is no known case in eukaryotes of a type I TZ being secondarily derived from a type II by shortening, but there are numerous phylogenetically diverse examples of the reverse. Therefore in the taxonomic section below I formally make phylum Telonemia a third infrakingdom of Harosa, which is in conformity with their being maximally supported as sister to infrakingdoms Halvaria plus Rhizaria.

When Sleigh (1995) reviewed ciliary ultrastructure he noted that cryptomonads and haptophytes both had two transverse dense plates in the TZ. In principle this might have been an ultrastructural synapomorphy giving evidence that Hacrobia are indeed a clade. However, subsequent work on deeper branching members of both phyla show that this is not universally true of either Cryptista or Haptista. As hacrobian TZ structure has not been reviewed comprehensively, Fig. 8 shows diversity within Cryptista and Fig. 9. samples that of Haptista, and I shall explain my conclusion that each ancestrally had only TP and subclades of both independently evolved a second plate, which are not homologues across Hacrobia. In both, the lower plate is the TP and upper one secondary. I show for the first time that Cryptista apparently fundamentally have a tripartite upper TZ structure (Fig. 8) and reveal a novel nonagonal structure in their lower TZ. I also show that comparisons of TZ structure in Haptista and Viridiplantae illuminate both the origin of Plantae and TP evolution generally. Manton (1964) suggested that the stellate structure she discovered may be homologous with that of Viridiplantae. For decades I and other protistologists disagreed, considering them purely convergent. My present unusually detailed analysis of TZ structure reveals some remarkable and unexpected similarities between haptophyte and plant TZs—perhaps not surprising as Haptista diverged from plants so close to the divergence time of glaucophytes, Rhodaria, and Viridiplantae.

## The type II TZ of Cryptista

In Cryptista, only Cryptophyceae and *Goniomonas* (together comprising derived superclass Cryptomonada) have two plates. Deeper branching Leucocrypta, e.g., *Kathablepharis* (Lee et al. 1992; Clay and Kugrens 1999), *Hatena* (Fig. 8D; Okamoto and Inoue 2006), and subphylum Palpitia (Fig. 8F-H; Yabuki et al. 2010) are often thought to have a standard single TP, which is thus the ancestral cryptist condition. *Palpitomonas* the most divergent definite cryptist has a TP of fairly even thickness, but with projecting lower hub and a slightly narrower distal hub (Fig. 8F-H). In other cryptists (i.e., subphylum Rollomonadia) a lower hub is not obvious and an upper hub (with dense lumen) obvious only in *Goniomonas* (Fig. 8C); as in many heterokonts the central disc of rollomonad TPs is thicker than its periphery (about five times so in *Cryptomonas* (Fig. 8A)). As contrasting upper and lower hubs are often obvious in heterokont TPs (e.g., Fig. 1C), having both may be the ancestral condition for Chromista, modified by losing one or both hubs in some lineages. In *Cryptomonas* (Fig. 8A) the annular connexion (ac) is offset distally from TP by the same amount as in heterokonts (Fig. 1C), probably also true in other cryptists but ac is often less distinct; in *Goniomonas* (Fig. 8B) the constriction probably marks its position. In *Cryptomonas reticulata* contrast is particularly good (Lucas 1970), revealing three distinct acs (Fig. 8A) and three distinct transverse plates, not just two, the middle one being at the site of the constriction and thus likely mechanically responsible for it. The uppermost plate is very slender and in line with the uppermost ac and also the end of the cp mts, so I call it the axosomal plate (ap). The middle plate is thicker, centrally curved and opposite and likely connected to the middle ac; as it is positionally related to the upper hubs of *Palpitomonas* and *Goniomonas*, in which it does not take the form of a plate, I call it the upper plate (up). The lowest plate is thickest with a conspicuous central disc and offset proximally from the lowermost ac to the same degree as in heterokonts, ciliates, and most Protozoa and thus the canonical TP, found in all Cryptista.

A homologous ap is present in all Rollomonadia, but not clearly visible in *Palpitomonas*. Nonetheless, despite its absence the *Palpitomonas* cp starts at exactly the same distance above TP as in Rollomonadia, revealing an underlying shared geometric pattern (which must have a hidden shared molecular basis) throughout Cryptista that can be expressed in different structures in different branches of the phylum. The middle plate (up) is absent in the blue green cryptomonad *Hemiselmis amylosa* (Fig. 8B), almost maximally divergent from *Cryptomonas* on rDNA trees (Clay and Kugrens 1999; Hoef-Emden 2008), but is probably represented by the hub-like structure attached below ap (and perhaps also contacting TP). In *Goniomonas* this mid position is occupied by a solid hub attached to TP (Fig. 8C).

A reasonable candidate for an acorn-V structure is present in most cryptist LSs (Fig. 8A, C, F, G, J, L) but I found no TSs that unambiguously demonstrate it. The proximal TZ between TP and putative aV is several times greater in Rollomonadia than *Palpitomonas.* A nonagonal fibre is apparently present in all cryptists in that zone (obviously nonagonal in *Hatena* (Fig. 9D) and *Hemiarma* (Fig. 9E), only visible in LS in most others), though in '*Kathablepharis*' marine clone G-2 it has 9 extra radial connectors alternating with the doublets and thus is really 18-gonal (Fig. 8M). The generality of proximal nonagonal fibres (really open prisms) in cryptists was previously overlooked—noted only for *Hemiarma* (Shiratori and Ishida 2016).

*Hemiarma*, strongly sister to *Goniomonas* on two-gene rDNA trees, was stated to have a single transverse plate (Shiratori and Ishida 2016). However I argue that *Hemiarma* actually has homologues of both TP and ap but this was not obvious as the thin outer part of TP is strongly curved upwards and contacts the doublets immediately below ap, so their distinctness in less obvious. That means the common ancestor of Cryptophyceae, Goniomonadida, and *Hemiarma* had two plates, ap and TP; I placed *Hemiarma* in superclass Cryptomonada (Cavalier-Smith 2017a, 2017b, 2017c), and regard two TZ plates as a synapomophy for cryptomonads—but not for Cryptista. However *Hemiarma* appears to have lost all trace of up, its cp passing through ap, unlike all other Cryptista. *Hemiarma* was placed in separate order, Hemiarmida included with Goniomonadida within class Goniomonadea (Cavalier-Smith 2017a, 2017b, 2017c, Cavalier-Smith 2018 supplementary material Table S1), as its TP is also unusual in being concave, its periplast plates are irregular polygons not squares as in Goniomonadida and are absent from the left half of the cell, and it lacks a furrow/gullet complex, and is extremely genetically divergent from *Goniomonas*. However, its basic body plan with large ejectisomes arranged transversely in the cell anterior, not longitudinally as in Cryptophyceae and Leucocrypta indicates that this transverseness evolved in the common ancestor of *Hemiarma* and *Goniomonas*, if they are sisters as the two-gene tree shows. If so the nakedness of half the *Hemiarma* cell and its concave TP and loss of up are secondary modifications, not ancestral for Cryptomonada. The weak branching on 18S trees just below Cryptophyceae plus Goniomonadida is probably a tree reconstruction error caused by insufficient conserved information compared with that for the whole rDNA operon.

Though *Kathablepharis* is stated to have only one transition plate (Lee et al. 1992; Clay and Kugrens 1999) Fig. 8J shows that in *Kathablepharis ovalis* in addition to a prominent dense TP, there are two fainter plates above it in the precise positions of up and ap in relation to the constriction. This shows that the TP/constriction zone of Rollomonadia was ancestrally fundamentally tripartite.

*Kathablepharis* clone-G2 is unusual amongst Rollomonadia in having nearly orthogonal centrioles (about 80° in Fig. 8K, not nearly parallel as wrongly depicted in Fig. 37 of Lee et al. 1992), whereas all other studied rollomonads have them parallel or nearly so (e.g., Fig. 8A,I). It is also exceptional in having a transition helix (TH) of about four gyres (Fig. 8K, L) located distal to TP. It appears to be located just within the outer doublets as in heterokonts (Fig. 1C), and was thus depicted in Fig. 37 of Lee et al. (1992), but their text contradictorily says it is between doublets and membrane. They may have confused it with the probable Y-links in that position on the left of TH in Fig 8L. The main difference from the heterokont TH is the greater gap between the TH base and TP in '*Kathablepharis*'. But this arguably stems from the presence of both up and ap which would force TH to begin just above ap, not TP. Interpolating ap/up between TP and TH seems not sufficiently important a difference to be incompatible with TH being homologous structures that may have evolved in the ancestral chromist and have been lost by other lineages that apparently lack a TH. But establishing the molecular basis of both would be required to test that. Its plausibility is somewhat increased by the fact that even some ciliates (even more closely related to heterokonts) have one or two gyres that might be a TH just above TP (e.g., *Paramecium* Fig. 5A). The fact that clone G-2 has orthogonal centrioles and a putative TH unlike *Kathablepharis ovalis* (Fig. 8K, L) makes it very unlikely that it is really the same genus. More likely it is yet another example of Mylnikov's brilliance in discovering and cloning novel flagellates but misinterpreting them as known genera (as for *Ancoracysta marisrubri* originally misidentified as *Colponema*; see Cavalier-Smith et al. 2018 and several other cases). Unfortunately we have no molecular data for it. If any is found I predict it will branch somewhat lower in the rollomonad tree before the ancestral orthogonality of centrioles otherwise seen only in *Palpitomonas* was lost, as they became parallel before *Kathablepharis ovalis* diverged from Cryptomonada but after *Palpitomonas* diverged.

A TH might have been ancestrally present in all early diverging cryptists when they still had orthogonal centrioles. Even *Palpitomonas* has three or four peripheral dense granules inside the distal TZ doublets just above TP (Fig. 8G) that might be a loose version of a TH, though not previously remarked on. Exclusion here of both *Telonema* and *Picomonas* from Cryptista, where they were formerly classified, together with the aciliate pseudoheliozoan *Microheliella* (Cavalier-Smith et al. 2015), makes Cryptista homogeneous with respect to TZ type. I now think it incorrect to group *Heliomorpha* with *Microheliella* (Yabuki et al. 2012) as its kinetocyst extrusomes, long centrioles, and flat centrosomes support its original classification instead in the same order as *Tetradimorpha radiata* that lacks such channels, i.e., Heliomonadida Cavalier-Smith (1993a), if we accept that *Heliomorpha* and *Microheliella* evolved transnuclear cytoplasmic channels for their axopodial axonemes independently, as I now do; this is done in my revised classification of Cercozoa (Cavalier-Smith et al. 2020). *Heliomorpha elegans* has a complex type I TZ with hub-like axosome and TH (studied as *Dimorpha mutans*: Brugerolle and Mignot 1997 Fig. 10), consistent with but not diagnostic for it being a cercozoan.

The main reason for my changed view is that '*Tetradimorpha*' *pterbica* (Mikrjukov 2000) now seems a better candidate than *Heliomorpha* for a ciliated relative of *Microheliella*. That is because as well as sharing transnuclear axopodial channels both have globular centrosomes with distinctive shell and core, unlike any other axopodial eukaryotes, and both have simple flattened extrusomes, not complex kinetocysts, though these are more irregular in shape and at least twice the size in *T. pterbica*. I had previously overlooked these three major ultrastructural similarities with *Microheliella*, partly because Mikrjukov (2000) included only a summary diagram not actual electron micrographs of *T. pterbica*. He also found that unlike in *T. radiata* (Brugerolle and Mignot 1984) centrioles within each of the two pairs were parallel not divergent and also were only about 2.5 times longer than wide, and thus not exceptionally long as in heliomonads (*Heliomorpha* and *T. radiata*). These four differences from *T. radiata* are at least as evolutionarily significant as the differences from *Heliomorpha*, so I do not accept that *T. pterbica* should be in the same genus or even order as *T. radiata*. Therefore I establish below a new genus *Tetrahelia* for it and restrict order Axomonadida established by Yabuki et al. (2012) for *Tetradimorpha* generally to *T. pterbica* only, plus any other flagellates with the same centrosomal and centriolar pattern that may be discovered, and place thus refined Axomonadida in existing class Endohelea together with *Microheliella*, which it most resembles ultrastructurally; they differ essentially only in the presence or absence of cilia and pseudopellicle. As Telonemia and Picozoa are now excluded from Cryptista, I abandon superclass Corbistoma that erroneously grouped them together, as well as the similarly polyphyletic subphylum Corbihelia which also included Endohelea (Cavalier-Smith et al. 2015). Instead I now raise former superclass Endohelia in rank to become the third subphylum of Cryptista, comprising only class Endohelea (*Microheliella*, *Tetrahelia*).

Although we lack direct sequence evidence for the phylogenetic position of *Tetrahelia*, for *Microheliella* being sister of other Cryptista is currently the best estimate from multiprotein trees. If both endohelians are really the most divergent cryptists, and if Mikrjukov's diagram is accurate, then *Tetrahelia* has type I TZ, implying that was the ancestral state for cryptists. However, that is doubtful as a cursory drawing of *Palpitomonas* TZ might also put TP level with the plasma membrane as it appears in the right cilium (but not the left) in Fig. 8F and overlook the proximal part of the TZ. Therefore without original micrographs we cannot exclude the possibility that the *Tetrahelia* TZ is essentially identical to that of *Palpitomonas* (with which *Microheliella* strongly grouped on the 2-gene rDNA tree: Yabuki et al. 2012) with a short proximal TZ. It is likely that his figure would have shown a more complex TZ (as it did for *Heliomorpha*) if more structures than a simple TP were present. The parallel nature of each *Tetrahelia* centriole pair is characteristic of all cryptists except *Palpitomonas*, thus not a reason for excluding it from Cryptista. Endohelea, now comprising only *Tetrahelia* and *Microheliella*, have tubular cristae like Leucocrypta, not flat ones as in Cryptomonada and *Palpitomonas*; so keeping them in Cryptista does not make it more heterogeneous in crista form than it is otherwise. Two changes from tubular to flat need to have occurred irrespective of whether Endohelia are included or not. *Tetrahelia* was from a low salinity mangrove swamp and *Microheliella* from a low salinity estuary, so Nucleohelea generally may be brackish specialists.

By contrast, *Rhodelphis* and *Picomonas* both having type I TZ with a distal plate substantially above TP as in Glaucophyta strongly supports their all being Biliphyta, and excludes them from Cryptista. The fact that *Telonema* has a type I TZ without a distal plate, the ancestral condition for Harosa, fits Telonemia being sister to Harosa not Cryptista, as now shown by Strassert et al. (2019). When TZ structure (with a great deal of complex information when carefully interpreted) agrees with multiprotein trees we can be rather confident that we have the correct phylogeny. Thus the contrasting TZs of Picozoa, Endohelia, and Telonemia, formerly all grouped as Corbihelia perfectly fit their assignment here respectively to Biliphyta, Cryptista, and Harosa, stimulated by my discovery that *Rhodelphis* also has a fundamentally biliphyte TZ organisation.

## Type II TZs of Haptista

Currently Haptista are divided into three subphyla (Haptophytina, Heliozoa, Alveidia: Cavalier-Smith 2018; Cavalier-Smith et al. 2018). Heliozoa include only Centroheliozoa that lack cilia, so I can discuss only haptophyte classes Pavlovophyceae and Prymnesiophyceae, and the alveid heterotroph *Ancoracysta*. TZs of all are invariably type II, relatively short proximal TZ in Pavlovophyceae but rather long in *Ancoracysta* and Prymnesiophyceae, the latter being quite variable in TP-associated structures.

Simplest are Pavlovophyceae (Fig. 9A-D), where there is only one prominent TP in *Diacronema* (Green and Hibberd 1977) and in *Pavlova pinguis* (Green 1980), which represent two of the three main subclades (Bendif et al. 2011) and in *P. granifera* (Green 1973). *Diacronema* TP is connected to its cp by a long central filament subdivided into distal and proximal parts that stain differentially (Fig. 9A, B). A nonagonal tube is proximal to TP similarly to cryptists (Fig. 9C). *Ancoracysta twista* has a deeply curved bowl-shaped TP with large sub-axosomal mass lodged in it connected by a short filament to a small axosome at cp's base; no proximal nonagonal structure is evident, but a likely acorn-V is present in LS (Fig. 9I). *Ancoracysta* and Pavlovophyceae TP is level with the upper part of a marked broad constriction with dense annular connexion. *Prymnesium* has an even longer central filament in this position; as cryptists lack such a filament, it is likely a synapomorphy for Haptista, uniting haptophytes and alveids, adding to strong support for the haptophyte, heliozoan, alveid clade by 201-protein ML trees (Janouškovek 2017 Fig. 2A), though PhyloBayes put *Ancoracysta twista* a node lower. The proximal TZ nonagonal fibre found in all Cryptista may be another synapomorphy uniting Hacrobia, as it is in *Diacronema* also; and probably also in *Isochrysis*; presence in *Ancoracysta* cannot be excluded. *Ancoracysta marisrubri* (misidentified as *Colponema*: Mylnikov 2009) has an extra, thin, curved plate at cp's base similarly to ap of rollomonad cryptists (Fig. 9J), but *A. twista* has only a hint of such a structure (Fig. 9I). If overstained *Diacronema* shows a lightly stained secondary plate in the same position (Bendif et al. 2011 Fig. 9D).

Prymnesiophytes have substantially different TZ patterns in each of the three major clades, but in all TP has a distinctive top-hat-like structure with raised central crown (ancestrally attached to the central filament) (Fig. 9E-H; K-Z). Clades A (Phaeocystales Fig. 9M) and B (Prymnesiales Fig. 9K, L, S, T) of Edvardsen (2000) independently greatly lengthened their TZ distally by inserting extra structures including a distal plate below the central filament, whereas the largest clade C (orders Coccolithales and Isochrysidales) retained a moderately long TZ with single TP—the composite annular septum of *Isochrysis* (perforated by cp; Fig. 9E-H) is apparently unrelated to distal plates of Prymnesiales (Fig. 8K, L, S, T) and *Phaeocystis* (Fig. 8M), which are below cp. As TZ are somewhat longer in clade C than in Pavlovophyceae, but similar in length to the outgroup *Ancoracysta*, this length and presence of only a single TP is the ancestral state; thus Pavlovophyceae did shorten their TZ and centrioles secondarily as Moestrup (1982) postulated. *Isochrysis* has a putative acorn-V (Fig. 9G).

However all three prymnesiophyte clades are derived with respect to the form of TP, which in LS resembles a hat with a raised crown and broad brim (Kawachi and Inouye 1994; Sym and Kawachi 2000). The detailed structure of the hat varies; its top is usually flat, sometimes with a depression that may or may not contain a granule and its sides consist of dense granules, often three, that usually flare outwards; its brim is usually flat, but in *Prymnesium* is sloping making its hat-like profile less obvious (Manton 1964 called it a domed septum). In some organisms the crown edges are continued upwards making its profile more H-like, thus superficially resembling the TP/stellate complex of Viridiplantae. Often a dense disc is present just above the top of the hat. In some the brim is continued inwards giving the impression that the hat is basally closed. Another variant in *Pleurochrysis* sp. (Fig. 9O, P) only is the presence of eight extra dense rings below the hat, which I suggest may have arisen by repeatedly duplicating the lowermost tier of hat lateral granules to make a more robust base to the TZ. Figure 9 illustrates most of these variants. *Pleurochrysis carterae* has the simple hat (but with some extra central density) and instead of the thick rings has slenderer subTP peripheral rings more like a nonagonal fibre (Beech and Wetherbee 1988).

In clade C the cp usually remains attached to the centre of the top of the hat. In *P. carterae* when cilia disassemble before division, the hat and lower half of the TZ remain attached to centrioles at the spindle poles (Beech et al. 1988). Moreover in coccolithophorids vegetative cells are often non-ciliate; such cells keep the hat and lower TZ, but the cp is absent (e.g., *Cruciplacolithus*: Kawachi and Inouye 1994). *P. carterae* and many other prymnesiophytes autotomize their cilia under stress at the same position immediately distal to the hat (Beech et al. 1988), just as Chlorophyceae do immediately distal to the H-profile basal cylinder. Thus the central filament is a weak point and some filaments just distal to the TP hat are likely to include centrin as in green algae. The hat structure itself might have evolved in the ancestral prymnesiophyte to stabilise the TZ stump during autotomy allowing ready healing and to enable controlled 9+2 depolymerisation before division and prevent wasteful lower TZ disassembly in the longer TZ of prymnesiophytes. However, the next section gives reasons for thinking that hat structure may predate Haptista and its apparent absence in Pavlovophyceae may result from secondary compression.

In clade A (Phaeocystales the deepest diverging of ultrastructurally characterised prymnesiophyte clades), *Phaeocystis* vegetatively has large multicellular gelatinous aggregates with nonciliate cells, only zoospores or gametes developing cilia. That may be why their zooids uniquely in Haptista appear to have lost the central filament connection to the cp and added a novel long dense basal cylinder overlain by a dished dense distal partition (dp in Fig. 9M); cp appears to be attached directly to the depressed central zone of dp. However, a central filament remains in the lumen of the basal cylinder implying it is attached to the centre of dp distally and TP proximally Fig. 9M*. Possibly as short-lived reproductive stages, this TZ lengthening and extra stability was more important than retaining autotomy ability.

In contrast clade B (Prymnesiales) retained the central filament but hugely lengthened the separation between TP and cp. They increased stability despite this expansion by adding a distal partition (dp), not immediately below cp, but I suggest at the central point marked by the transverse density in *Diacronema* central filament (Fig. 9A). The new dp is neither hat-like nor dished in profile but flat; in *Prymnesium parvum* it appears homogeneous, the much elongated central filament passing through it and retaining its central attachment to the top of the hat (Fig. 9S). But in *Hyalolithus* the central filament appears to terminate at a central differentiation of dp, and not to continue below dp—connection to the hat seemingly lost (Fig. 9K, L); in this silicified species the filament above dp bears a dense hub/granule and below cp a fainter one (Fig. 9L). By contrast in *P. parvum* it appears to be the longer part of the central filament below dp that retains the dense hub at least (Fig. 9S). Thus though both have similar longitudinal differentiation of the central filament it is differently spaced relative to dp in the two genera. *Imantonia*(Green and Hori 1986), the most divergent genus, is more like *Prymnesium* so it seems that *Hyalolithus* underwent secondary shortening of its central filament and reattachment to a novel differentiation of dp.

Immediately distal to the TP hat *P. parvum* has a system of peripheral filaments just inside and attached to the A-tubule feet of the doublets, which Manton (1964) called a 'stellate structure', believing it to be homologous with that of green plants. As this is the site of autotomy it is likely that part of this structure contains centrin. But that does not make it homologous. Many different centrin-containing structures are not positionally or ultrastructurally homologous. The review by Karpov and Fokin (1995) called it a pseudostellate structure but their diagram misrepresents its structure in several ways and is useless for interpreting its homologies as they did not notice that its structure differs at different levels, omitted important details, and misplaced the stellate structure axially. I think Manton herself misinterpreted the exact positions and homologies of these structures, so present a new interpretation; Fig. 9T indicates the levels where I think the TSs in Figs 9U-X came and Fig. 9T shows where Manton thought they were. I therefore reexamine it in detail here; when comparing it critically in the next section with Viridiplantae I shall show that parts are homologous and parts not.

Distally this structure is not really star-like but comprises straight filaments that connect A-tubule feet of alternate doublets, which as Manton points out implies they are in spiral form and cannot be simple rings—nor are they a regular polygon like a nonagonal fibre, nor do they seem star-like! More proximally several extra shorter filaments are added to this spiral system which make it look more star-like. But there are not just nine star points as in Viridiplantae (Fig. 3), but apparently 18, half attached to A-tubule feet and half to structures in between the doublets. In the centre of Fig. 9X is a central granule and a starfish-like structure with nine points, which though having some substructure is not so obviously made of discrete structures as the peripheral 18-fold lattice; it closely resembles the innermost star shape in the TP of *Cyanophora cuspidata* (Fig. 6B*). I think Fig. 9X represents a section in whose thickness lies the whole of the top-hat structure of prymnesiophyte TPs. I suggest the central granule is the central granule at the top of the hat to which the central filament connecting to cp is linked, that the central dense and rather diffuse 9-fold 'starfish' represents the flat top of the hat and that the 18-fold (approximately) peripheral lattice represents the brim of the hat. This new interpretation has wide implications for eukaryotes generally as the next section explains by detailed comparisons with outgroups. One advantage of Manton's pictures is their fixation only in osmium tetroxide, which preserves the basic framework well but not all the matrix proteins around the filaments. Therefore lattices like that of Fig. 9X are more clearly shown than in most preparations using glutaraldehyde prefixation which often increases density overall and reduces contrast because it preserves more 'background' protein, making disentangling TP structure like looking for a black cat in an unlit coal cellar.

First I argue that the flat top of the prymnesiophyte TZ hat-like TP is homologous with the dense central disc of the flat TP of Pavlovophyceae, which is attached to the central filament connecting it to the TP (Fig. 9A). In other words the central disc even in Pavlovophyceae will have the relatively amorphous 9-fold starfish structure. Second the brim of the hat is homologous with the less densely stained outer part of the pavlovophyte TP that connects to the doublets; thus I predict that pavlovophytes also will have the 18-fold star-lattice structure at the thinner less dense periphery of their TPs. Thirdly the change in shape to form the hat came about (in ancestral prymnesiophytes, unless secondarily lost by Pavlovophyceae) by inserting at least two rings of granules between the central 9-fold starfish and peripheral 18-fold lattice. This implies that 'starfish' and peripheral lattice are developmentally and chemically distinct structures, yet spatially associated, both homologous with corresponding parts of other TPs for which the next section gives strong evidence.

Clearly therefore I disagree with Green and Hibberd (1977) suggestion (before Prymnesiophyceae with only one TP were known) that the single TP of *Diacronema* corresponds with the upper dp of *Prymnesium* and *Chrysochromulina*; they did not explain why they thought that. The fact that in all prymnesiophytes only the lowermost partition closest to the centriole has the unique hat substructure means that the lower partitions are homologous throughout prymnesiophytes irrespective of whether a dp is present also. Furthermore the fact that dp has a different substructure in the two orders that evolved a second plate and different also from the lower plate means that distal plates are not even homologous across all prymnesiophytes and also did not evolve simply by duplicating the ancestral hat-like TP. Comparison of *Diacronem*a (Fig. 9A) with Hibberd's heterokont *Uroglena* (Fig. 1C) makes it evident that its TP has moved only slightly distally relative to ac, compared with heterokonts, and that both have a very similar thicker central disc and thinner periphery. Figure 8A shows that in cryptists too that lack a dp, TP likewise has a thicker central TP disc equivalent in profile to the hat-top/starfish-body part of haptophyte TPs. Thus all three major chromist subgroups (Cryptista, Haptista, Harosa) probably had essentially the same TP substructure ancestrally; I shall argue that this conclusion applies not only to all chromists, but to all corticates, whereas all other eukaryotes have a similar, probably somewhat simpler, TP lattice structure.

## Generality of corticate TP substructure

I now reconsider Manton's idea of TZ homology between Viridiplantae and haptophytes by arguing that the TP basic structure of Plantae and Haptista is homologous, but many features distal and proximal to it have diverged radically. One clue is extra star points in between the nine canonical star points attached to A-tubule feet in Viridiplantae. Previously overlooked in the colourless green alga *Polytoma* (Lang 1963) are Y-shaped filaments where the arm tips of each Y join the points of two adjacent canonical star-points and the stem of the Y points outwards radially between the doublets (Fig. 10I). Though in Fig. 10I these star points can only be clearly seen between three adjacent doublets, this is likely because the section just grazed one side of them, slightly obliquely. The reason such structures have been generally overlooked in Viridiplantae must be because they are spatially restricted to a very thin slice of the TZ—a much narrower zone than the easily seen nine canonical star points.

They appear to be almost identical to the extra star points between at least eight of the A-tubule-feet -contacting stars in the haptophyte *Prymnesium* (Fig. 9X, magnified Fig. 10N) discovered by Manton (1964) which I interpreted above as part of the peripheral lattice forming the brim of the prymnesiophyte hat-like TP. From Manton's LS, I calculate the brim of the hat to be only ~8 nm thick or less, thus little more than a tenth of the thickness of many thin sections, thus easily overlooked or concealed by superimposition on other structures. Both *Prymnesium* and *Polytoma* extra star points appear to have additional short filaments connecting on one side to the B tubule or crossing the star point, so the peripheral lattice they form is actually more complicated than just an 18-fold star. In addition, on the opposite side from the extra star tip in *Polytoma* Fig. 10I one can see extra beaded filaments linking the ends of the A-tubule feet as in a nonagonal fibre. This suggests there may be a very short nonagonal fibre either just above or just below the extra star points, so that if the TS is slightly oblique one may see one or the other on opposite sides of the doublet ring.

EM tomographic slices can be as thin as 2.3 nm. Tomography of a single TZ fixed by fast freezing and freeze substitution by glutaraldehyde and osmium reveals the same inter-doublet Y-shaped star-point structures (Fig. 10L) in *Chlamydomonas reinhardtii* (O'Toole et al. 2003). The authors apparently overlooked them and their evolutionary significance, commenting only on the conspicuous central dense disc in the next, more distal tomogram, shown here in Fig. 10M; this amorphous disc represents the dense base of the distal basal cylinder (large arrow in the LS tomogram in Fig. 10J), as Fig. 10M must lie between the distal and proximal stellate structures as it does not include star points of either; interestingly this tomogram shows a beaded ring just inside the A tubules, not visible in adjacent tomogram 10L, which may correspond with the beaded filaments of *Polytoma*. Fig. 10L tomogram is immediately proximal to Fig 10M tomogram and must represent the upper (most distal) part of the proximal basal cylinder which should include its faintly staining distal septum discussed above and also shown in a standard EM preparation (Fig. 10H). Figure 10L shows that the distal septum is real and has a lattice-like substructure. The marked diffence in appearance of the central septa confirms my longstanding interpretation that the crosspiece of the H in *Chlamydomonas* is a composite structure of two adhering plates. I argue that the Fig. 10M lattice is the previously overlooked skeleton of the *Chlamydomonas* TP and that this septum is homologous with the disc that forms the top of the haptophyte hat-like TP. As also argued above the distal basal cylinder also has a subapical distal septum, to which the cp appear attached in Figs 10H and J, which is confirmed by the distalmost tomogram, Fig. 10K, which shows that the upper cylinder's distal septum also has a lattice substructure, but the lattice pattern differs and has a central hub/granule. Not only is the peripheral lattice of the green algal and haptophyte TP virtually identical, but the central zone is rather similar, with a lattice substructure essentially indistinguishable in *Chlamydomonas* from that in *Prymnesium* (Fig. 9X), which in turn is also indistinguishable from that of *Cyanophora* (Fig. 6B*), except that it has a large almost central granule (or narrow hub) not a smaller eccentric one), not seen in *Chlamydomonas*. The other important difference is that the canonical star points are distinctly larger in Viridiplantae, making the diameter within the basal cylinder substantially smaller than in Viridiplantae. But since filaments can get longer or shorter in evolution size alone is not a sound reason for denying the likely homology of the basic filamentous skeleton of the TPs of haptophytes and Viridiplantae. Dimensions of the *Cyanophora* star are as for haptophytes, thus representing the ancestral condition making Viridiplantae the odd one out, implying that their proportions altered when the longer stellate structures were added.

Detergent-extracted isolated centriole/TZ complexes also clearly show the composite nature of the H crosspiece: in Fig. 10Q the medium-density lower half of the composite is clearly in line with and distinct from the much denser bottom of the distal basal cylinder. Therefore this lower continuous mid-density layer is part of the TP, whereas the dense base of the distal cylinder is an optional extra. This is proven by evolutionary experiments. In the scaly prasinophyte green alga *Nephroselmis* (=*Heteromastix*) *rotunda* the distal cylinder lacks both the proximal dense base and the distal septum, being open at both ends (though with central hub and spokes throughout: Fig. 10A). Instead the cp is apparently attached by a filament to an axosomal plate distal to the basal cylinder. In contrast the proximal cylinder has a prominent distal and fainter proximal septum, making it hat-shaped in profile. The crown of the hat is attached to the most prominent doublet inner projections (opposite the ac), which probably represents the TP ring (TPr) seen in the tomogram of *Chlamydomonas* TP (Fig. 10M) by very slender filaments which I postulate to have the same lattice structure as the peripheral TP lattice in *Prymnesium* and *Chlamydomonas*. The prasinophyte genus *Pyramimonas* (a particularly early diverging lineage) unlike most Viridiplantae has a TZ coiled fibre very like the heterokont transition helix (TH); Fig. 10B, R-T compares these structures. *Pyramimonas* generally has only one stellate structure and basal cylinder; in *P. orientalis* this stellate structure is hat-like as in *Nephroselmis* and is probably the proximal basal cylinder*—*its basal septum may be connected to the putative acorn-V by a longer and more tenuous filament than in *Chlamydomonas*. The non-hat-like basal cylinder of *Pyramimonas obovata* (which also has a TH) appears to be a short version of the distal stellate structure as it is just distal to a typical thin TP with central thickening that itself is very close to the cell surface (Melkonian 1982 Fig. 17); thus this species has a type I TZ of an apparently rather primitive type compared with other Viridiplantae. In *Pyramimonas orientalis* the central septum is thicker than in *Nephroselmis* and has a hub-like substructure in LS (Fig. 10B) and may be a modified TP. The deepest branching scaly streptophyte biciliate, *Mesostigma*, also has only one proximal basal cylinder but no TH; though no obvious transverse plate was seen (Melkonian 1989) four LSs (his Figs 24-6, 54) suggest a plate is present at its base and a clear amorphous septum is evident in his Fig. 8 TS. I conclude that *Mesostigma* lost the proximal stellate structure but retains a weakly stained TP similar to those of *Chlamydomonas* and *P. obovata* at or immediately below the base of its distal stellate structure, which sometimes appears hat-like rather than flat (Fig. 10D); the distal stellate structure is also rather long as in multicellular streptophytes like *Coleochaete* that have retained a short proximal cylinder and a thicker TP between the stellate structures (but not attached to either: Fig. 10G). These important variants must be considered when discussing the origin of the viridiplant TZ from a biliphyte ancestor; one must not assume that the ancestor was like the rather derived and specialised *Chlamydomonas* type.

These striking similarities in TP skeleton between haptophytes (kingdom Chromista) and Viridiplantae raise the question whether the TP of Biliphyta also resembles that of these two taxa, and whether the same is true of more distant chromists, notably Harosa. An alert reader will have noticed that I already provided the answer for Harosa, when discussing the hub-lattice structure of Cercozoa. Look back at Fig. 4A, B of the early branching cercozoan *Bigelowiella*, and you will see that at the top of Fig. 4A the peripheral lattice includes some 'extra' open star points that point between the doublets, especially clear on either side of doublet 7; however, there also appear to be small star points like those of *Prymnesium* TP pointing directly to some of the A-tubule feet, notably on doublets 1, 5, 7, 9 in the position of the canonical star points of Viridiplantae; between 5 and 6 there appear to be three overlapping star points two attached to the A-tubule feet and a third out of phase in between. There are radial spokes from a central hub connecting to the A-tubule feet. Viridiplantae, haptophytes, and the outer part of the cercozoan hub lattice/TP share the same peripheral filament pattern of nine Y-like points offset from the doublets, so pointing between them, in addition to nine open star points in phase with and linked to the A-tubule feet.

**Fig. 11.**
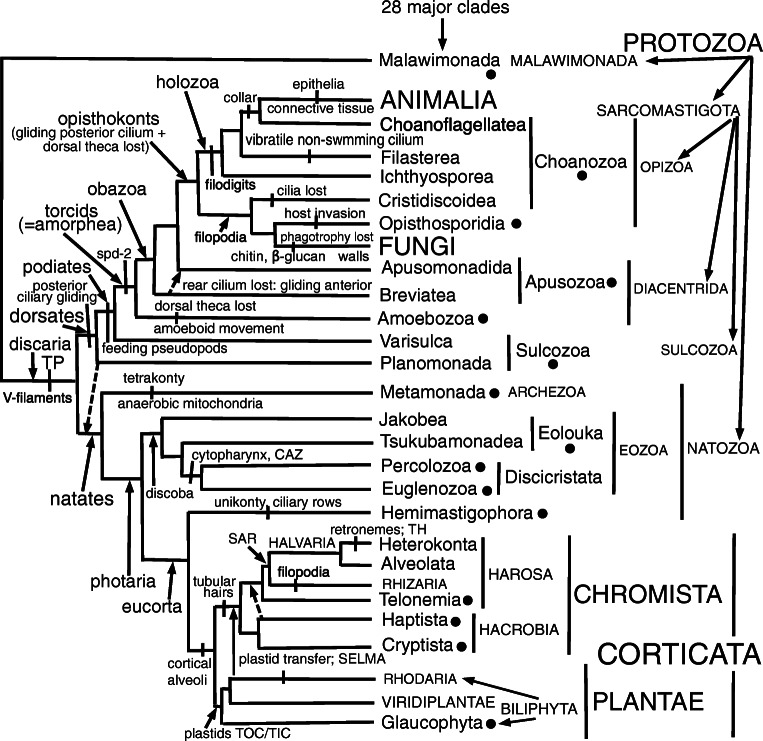
Relationships between all major eukaryote clades based on multiprotein sequence trees. Clades classified as kingdoms, subkingdoms and infrakingdoms are in capitals of correspondingly decreasing size; the others are mostly phyla (marked by blobs) or super or subphyla; classes end in -ea, orders in -ida. Clades not treated as taxa are in lower case. The tree is rooted between Malawimonada and discaria as the text explains. Site-heterogeneous trees are contradictory about whether Apusozoa is paraphyletic, as suggested by a 159-protein, 68-taxa tree (Brown et al. 2013) or a 351-protein, 61-taxa tree (Brown et al. 2018) and shown here, or holophyletic as on a 253-protein, 151 taxon tree (Gawryluk et al. 2019); both contradictory positions of breviates and apusomonads had maximal statistical 'support'! The position of Haptista is controversial; only some multiprotein trees group them with Cryptista as shown, which a shared lateral transfer makes most likely (Cavalier-Smith 2018), others grouping them as sister to Harosa or (probably erroneously) put cryptists with Plantae. If Plantae, Chromista, and Discicristata were shown as single clades only 21 would be needed to represent the entire diversity of eukaryotes or 20 if Apusozoa are holophyletic (consistent with ultrastructure)

I therefore argue that the ancestral corticate TP had a thin peripheral lattice of two offset sets of nine open star points and associated shorter filaments connecting to the B tubule plus an inner more amorphous denser disc with the same underlying lattice structure as the *Chlamydomonas* TP. These features therefore evolved more deeply in the Fig. 11 eukaryote tree than previously recognised.

## Rhizarian TZ hub-lattice features shared with Plantae

Originally Cavalier-Smith et al. (2008a, 2008b) interpreted the major part of the innermost dense central ring in Fig. 4A of *Bigelowiella* as equivalent to the central hub of the *Sainouron* TZ (Fig. 4F). But unlike *Sainouron* TZ LSs (Fig. 4N, O) where the dominant central structure is indeed hublike, in *Bigelowiella* LS (Fig. 4B) the dominant structure at this level is the central dense disc of the TP, the only slightly hublike structure being a very thin layer proximal to this disc connecting it to the putative acorn-V structure below. In view of the clear homology shown above of the *Bigelowiella* TZ peripheral lattice with that of the central dense disc of the TP of *Chlamydomonas* and *Prymnesium*, I now interpret the peripheral lattice of the rhizarian hub-lattice structure as homologous with the skeletal lattice of the TP periphery in corticates generally. This new interpretation of the rhizarian TZ hub-lattice recognises the peripheral lattice and hub-spoke system as evolutionarily and spatially separable structures, which can be easily superimposed within one EM section and thus easily conflated. The lattice is clearly conserved across corticates. I suggest some TZ hub structures are also, as suggested by the Fig. 7F*Picomonas* hub overlooked by Seenivasan et al. (2013), which is also surrounded by a faint lattice which includes a complex system of radiating star points similar to but less clear than that of *Chlamydomonas* TP; peripheral star points appear to include some pointing between doublets and some pointing at A-tubule feet. The *Picomonas* hub is probably distal, not proximal, as one serial section proximal to it (Fig. 7G) appears to be a hub-less moderately dense TP including star points (large acute outer points in phase with A tubules and inner more obtuse smaller star points out of phase with them). The central density in *Bigelowiella* also shows innermost filled obtuse star points between doublets similar to those of *Picomonas* and of glaucophytes as explained below, consistently with it being TP's central disc not a hub, which are more peripheral in heterokonts at least (Fig. 1C). The apparent absence of interdoublet star points in Fig. 7G but presence in F suggests they may be restricted spatially to the distal end of this *Picomonas* stellate structure. That mirrors the situation in the *Chlamydomonas* proximal stellate structure where A-phase star points occur throughout its length but interdoublet ones are restricted to the distal septum. That suggests that pattern was inherent in the common ancestor of Rhodaria and Viridiplantae before the latter's more obvious thicker stellate structures evolved.

Reinterpreting the relationship between rhizarian hubs and the TP lattice is helped by the remarkable TP of the early branching monadofilosan cercozoan *Metromonas* (Mylnikova and Mylnikov 2011), unknown when I first described and reviewed hub-lattices (Cavalier-Smith et al. 2008a, 2008b, 2009). Unlike *Bigelowiella*, *Metromonas* and its ultrastructurally similar distant relative *Metopion* (Mylnikov et al. 1999) have a long type II TZ with exceptionally thick TP associated with a particularly broad constriction and ac and well separated from the acorn-V (Fig. 4D). At its centre is a so-far unique tapered funnel-like hub that is narrow distally (possibly solid there and projecting only slightly) but wide and probably hollow proximally, projecting more than the thickness of TP (enlarged in Fig. 4E). Peripherally *Metromonas* TP has longitudinal dense lateral rods midway between doublets and this central funnel. Similar lateral rods are present in the unusually thick hub of *Sainouro*n TZ (Figs 4N, O); in TS they appear as dense granules at the junction of the radial spokes and A-tubule feet (Fig. 4F). The funnel's narrow end passes through two distinct transverse plates, each the thickness of a typical TP, and protrudes slightly from the upper one. I suggest this uppermost transverse plate represents the original TP before metromonads evolved their unique massive tapering hub. *Metromonas* feeds by attaching its long cilium to the substratum and swinging its cell to and fro like a metronome to catch small flagellates (Larsen and Patterson 1990). The unique double structure of metromonad TPs probably evolved so that the extra lower transverse plate could better support the exceptionally long, uniquely tapering hub; by providing extra strength to the region connecting the basally flexing cilium to the cell body it may have been a useful preadaptation for evolving its unique rapidly nodding flexing of the base of its long cilium when attached. A similarly thick TP in *Metopion* suggests it has a funnel-like hub also (Mylnikov et al. 1999), but no good median LSs were published. Protrusion of the hub narrow end is similar to the central thickening seen in many normal slender TPs, e.g., in the imprecisely classified monadofilosan cercozoan *Katabia* (Fig. 4M). This remarkable hub of Metromonadea (overlooked by its authors) is the most prominent in Rhizaria and adds to the list of cercozoan classes in Table 1 of Cavalier-Smith et al. (2008a, 2008b) possessing TZ hubs.

**Table 1.** Classification of kingdom Protozoa* Owen 1858 em. Cavalier-Smith 2010 and its 11 phyla, 18 subphyla and 42 classes

**Subkingdom 1. Malawimonada** subking. n. **Diagnosis:** ciliary transition zone with atypically simple acorn filament system (no V-filament) at same axial level as axosomal 'half-plate'; lacking rotationally symmetric dense transition plate; axosomal half-plate connects by a dozen radial links to acorn peripheral filament, itself linked by A-tubule feet and A-B feet to five doublets; single microtubule (mt) of central pair embedded in half-plate. Biciliate non-amoeboid aerobes with centrioles askew, in different orthogonal planes separated by about 0.25 μm distally and laterally linked by a long twisted unstriated fibre. A striated band links the other side of the anterior centriole distally either to proximal end of right root (*Malawimonas*) or to posterior centriole base (*Gefionella*). Posterior cilium with one or two lateral vanes draws bacterial prey into ventral groove supported mainly by its split right microtubular root (R2; outer branch with I and B fibres) and singlet mt; left posterior root with C fibre supports left rim of groove; anterior root R3 supports anterior microtubular fan. Aerobes, mitochondrial cristae discoid. **Phylum Malawimonada** phyl. n. **Diagnosis:** as for subkingdom, plus at least some transition zone doublets have partial C tubules: no gliding or dorsal proteinaceous pellicle. **Sole class** Malawimonadea (*Malawimonas*, *Gefionella*). Subkingdom 2. Natozoa* subkingdom. n. **Diagnosis:** ancestrally swimming bikonts, mostly with single major distal striated connexion directly between two centrioles roughly in single plane, more in tetrakonts; ciliary transition zone with radially symmetric transition plate above asymmetric acorn-V filament complex; includes also secondarily surface-associated ciliary gliding phagotrophic euglenoids that retain pellicle from swimming ancestors. Cristae discoid or absent, less often tubular. **Etymol**: L. *nato* I swim; Gk *zoa* life. **5 phyla and 22 classes:** **Infrakingdom 1. Archezoa**Cavalier-Smith^1983^ stat. n. em. **Diagnosis:** anaerobic tetrakonts; TZ type I with TP attached directly above acorn-V filaments, cp mts directly attached directly above it; mitochondria hydrogenosomes or mitosomes without cristae, rarely absent. Split right centriolar root with I fibre and associated singlet root. With R3 anterior microtubular root. **Phylum Metamonada** Grassé, 1952 em. stat. n. Cavalier-Smith 2003 **5 classes** Subphylum 1. Anaeromonada Cavalier-Smith 1997 as infraphylum (=unranked clade Preaxostyla Simpson (2003), a junior synonym). Diagnosis as for **sole class** Anaeromonadea (TP with embedded cp) with 3 orders: Trimastigida Cavalier-Smith 2003 em. (*Trimastix*); Paratrimastigida ord. n. **Diagnosis** as for *Paratrimastix*(Zhang et al. 2015 p. 487); Oxymonadida Grassé 1952 (e.g., *Oxymonas*, *Pyrsonympha*, *Streblomastix*, *Saccinobaculus*) Subphylum 2. Trichozoa Cavalier-Smith 1997 (sequences evolve extra rapidly, especially for the parasitic non-phagotrophic majority) Infraphylum 1. Parabasalia, **2 classes**, Trichomonadea (e.g., *Tritrichomonas*, *Histomonas*), Trichonymphea (e.g., *Barbulanympha*, *Pseudotrichonympha*) Infraphylum 2. Fornicata infraphyl. n. **Diagnosis** as for the infraclasss (Cavalier-Smith 2013 p. 8; originally an unranked clade name: Simpson 2003); **2 classes**: Carpomonadea, e.g., *Carpediemonas*, *Chilomastix*, *Dysnectes*; Eopharyngea (Diplomonadida, e,g. *Giardia*; Retortomonadida *Retortamonas*) **Infrakingdom 2. Eozoa** (Cavalier-Smith 1997 (as subkingdom) stat. n., em. Cavalier-Smith et al. 2015) (=clade Discoba). **Diagnosis:** biciliates with ventral groove and I fibre plus singlet root associated with split right root but no anterior R3 root (i.e., Eolouka); and bikonts or double bikonts (i.e., Discicristata) without singlet root or I fibre but with microtubulecytopharynx supported by multiplied singlet roots**.** Phylum 1. Eolouka*Cavalier-Smith^2013^ stat. n. **Diagnosis:** Biciliates with ventral feeding groove; no homologue to malawimonad/metamonad/corticate anterior centriolar root R3. Cristae ancestrally tubular or vesicular, rarely flat; absent in Stygiellidae hydrogenosomes (Leger et al. 2016; Pánek et al. 2015). **2 classes** with non-homologous groove support: Jakobea (groove support mainly by root R1; posterior ciliary vane) *Histiona*, *Jakoba*, *Reclinomonas*; *Andalucia, Stygiella*, *Velundella*; Tsukubamonadea (R1 and vane absent; groove is between R2 branches (*Tsukubamonas*) **Superphylum 2. Discicristata**Cavalier-Smith 1993 (discoid cristae, paraxonemal rods, mt pellicle divides at ciliary attachment zone; cytopharynx supported by complex mt arrays distinct from centriolar roots) **Phylum 1. Euglenozoa**Cavalier-Smith^1981^ em. 1998 (latticed ciliary paraxonemal rods, biciliates with pellicle mts antiparallel to centriolar roots, 2 parallel centrioles with 3 roots; cytopharynx, no groove, cemented feeding apparatus, long extrusomes; discoid mitochondrial cristae, Golgi stacks; ciliary transition zone type II) **8 classes** (Cavalier-Smith 2016) Subphylum 1. Euglenoida Bütschli 1884 stat. n. Cavalier-Smith 1993 **5 classes** Subphylum 2. Postgaardia Cavalier-Smith^2016^ (*Bihospites*, *Calkinsia*, *Postgaardi*) Subphylum 3. Glycomonada Cavalier-Smith^2016^ 2 **classes**: Kinetoplastea (e.g., *Bodo*, ) Diplonemea (e.g., *Diplonema, Rhynchopus*) **Phylum 2. Percolozoa**Cavalier-Smith 1991 (amoeboflagellates, flagellates with 1, 2 or 4 cilia or aciliate amoebae; 2 centriole pairs; unstacked Golgi; varied ventral grooves; ciliary transition zone type I; anterior ciliary root R3 absent) **6 classes** Subphylum 1. Orthozoa **subphyl. n. Diagnosis:** centrioles in single kinetid comprising one or two orthogonal pairs; left posterior microtubular R1 root(s) well developed, *unlike* Heterolobosea and Lyromonadida; microtubular bundle connects R2 laterally to posterior centriole *unlike* Heterolobosea. **Etymol:***ortho* Gk straight, right, referring to centriole orthogonality; *-zoa* Gk life, typically animal, commonly used for high-ranked zoological taxa, e.g., phyla and subphyla. Class 1. Pharyngomonadea Cavalier-Smith in Cavalier-Smith & Nikolaev 2008 (4 cilia: 2 side-by-side bikont kinetids each with R1, R2 and striated rhizoplast; amoeboid stages only sometimes eruptive; dorsal fan microtubules absent) (*Pharyngomonas*). Class 2. **Lunosea cl. n. Diagnosis:**non-amoeboid biciliates, centrioles orthogonal, having microtubular roots R1 and R2 and recurrent cilium, but no ventral groove or cytopharynx; plus eruptive amoebae cladistically more closely related to *Dactylomonas* than to *Neovahlkampfia damariscottae* Page 1974. **Etymol:***Luna* L. moon refers to the often crescentic form of *Dactylomonas* and moon-crater-like cyst pores of *Selenaion*. Suffix -osea used as for class names in Amoebozoa. Sole order Selenaionida Hanousková et al. 2019 (*Selenaion*, *Dactylomonas*) Subphylum 2. Tetramitia Cavalier-Smith 1993 em. 2008 em. (centrioles parallel; root R1 absent except in uniciliate amoeba *Creneis* and non-amoeboid*Percolomonas*) **4 classes:** Class 1. Neovahlkampfea** cl. n. **Diagnosis:** only aerobic amoeboid stage known; phylogenetically defined as for Neovahlkampfiidae Hanousková et al. 2019 p. 136. Order Neovahlkampfida ord n. **Diagnosis:** as for Neovahlkampfiidae. **0 cilia** Family Neovahlkampfiidae Hanousková et al. 2019 (*Neovahlkampfia*) **Infraphylum Eutetramitia** Hanousková et al. ex Cavalier-Smith 2020. **Diagnosis:** centrioles parallel, ancestrally in pairs or groups of four, rarely single. Ancestrally with both amoeba and flagellate phases, either can be secondarily lost. **3 classes:** **Class 1. Heterolobosea**+ Page and Blanton 1985 (R1 root absent; without feeding groove, unlike Psalteriomonadidae or Pharyngomonadea; lacks numerous kinetids; invariably with limax amoeboid phase with eruptive lobose pseudopods; no microfibrillar bundle connecting posterior basal body to root R2) **2 orders:** Acrasida (aciliate amoebae: *Acrasis*, *Allovahlkampfia*, *Solomitrus*); Schizophyenida with 3 families: Vahlkampfiidae (*Tetramitus*, *Vahlkampfia*), Tulamoebidae (*Pleurostomum*, *Tulamoeba*), **Naegleriidae fam. n. Diagnosis:** amoeboflagellates or amoebae; cysts with multiple pores, not one as in Tulamoebidae; 0, 2 or 4 cilia. Type genus *Naegleria* Aléxéieff 1912 (*Naegleria*, *Willaertia*, *Marinamoeba*). **Class 2. Lyromonadea**Cavalier-Smith 1993 em. [Order Lyromonadida Cavalier-Smith, 1993 with 2 or 4 cilia but no root R1 (e.g., *Psalteriomonas*, *Sawyeria*, *Harpagon*, *Lyromonas*, *Oramoeba*, *Heteramoeba*); plus new order **Creneida. Diagnosis:** anteriorly uniciliate amoeba with broad anterior non-eruptive lamellipodium; seemingly uniquely retains R2 and R1 without retaining posterior cilium; rarely with a multiciliate phase with up to 14 cilia; kinetids with single centriole; acristate anaerobe (sole family Creneidae Pánek et al. 2014: *Creneis*). **Class 3. Percolatea**Cavalier-Smith in Cavalier-Smith & Nikolaev 2008 (no amoeboid phase: either 4 cilia with ventral feeding groove with single R1 and R2 and microfibrillar bundle connecting posterior basal body to R2 (*Percolomonas*) or with rows of many cilia in individual pockets surrounded by singlet microtubules, neither R1 nor R2 distinguishable (*Stephanopogon*). **Infrakingdom 3. Hemimastigophora** infraking. n. **Diagnosis:** two grooves with separate ciliary rows of monokinetid cilia, and cortical plates; extremely short centrioles; ciliary transition zone type I with distal nonagonal tube and distal hub/filament). **Phylum Hemimastigophora** Foissner, Blatterer and Foissner, 1998 (**sole class** Hemimastigea: *Hemimastix*, *Spironema, Stereonema***).** **Subkingdom 3. Sarcomastigota**Cavalier-Smith 1993 em. **Revised diagnosis:** Ancestrally and predominantly substrate-attached, benthic gliding bikonts with ventral groove and dorsal proteinaceous submembrane skeletal layer, plus derived creeping amoebae, sessile unikonts or vegatively amoeboid wall-free parasites; rarely secondarily planktonic swimmers that retain either a ventral groove (order Diphylleida), or single swimming cilium (Acanthoecida only). Ancestrally aerobes with flat or tubular cristae. Dense ciliary transition plate with 9-fold symmetry attached to A-tubule feet and A-B links. **5 phyla and 19 classes:** **Infrakingdom 1. Sulcozoa** infrak. n. **Diagnosis:** heterotrophic phagotrophs with ventral groove and single layered dorsal pellicle; with two or four cilia and/or narrow pseudopodia; ancestral ciliary gliding lost by diphylleids, both cilia lost by rigifilids; centrioles ancestrally orthogonal with two or one lateral fibrous connectors; distal TZ basal cylinder. Typically locomote on surfaces by posterior ciliary gliding or amoeboid motion; rarely (3 genera of Diphylleida only) swim with two or four cilia. **3 phyla:** Phylum 1. Sulcozoa*Cavalier-Smith 2013 em. by excluding Apusozoa and Discocelia. **4 classes:** Subphylum 1. Planomonada subphyl. n. **Diagnosis:** as for order Planomonadida (Cavalier-Smith et al. 2008 p.548) **sole class** Planomonadea cl. n. diagnosis as subphylum Planomonada (*Planomonas*, *Fabomonas, Ancyromonas*, *Nutomonas*; flat mitochondrial cristae; single short dorsolateral centriolar connector with faint longitudinal striations; non-pseudopodial) Subphylum 2. Varisulca Cavalier-Smith 2013 em. pseudopodial; left cross striated and right amorphous centriolar connectors [**3 classes:** Diphyllatea Cavalier-Smith, 2003 em. tubular cristae (Diphylleida: *Collodictyon*, *Diphylleia*, *Sulcomonas*); Glissodiscea Cavalier-Smith 2013 em. here by excluding Planomonadida and the cercozoan *Discocelia* (Mantamonadida: *Mantamonas*); Hilomonadea Cavalier-Smith 2008 em. 2012 flat cristae (Rigifilida: *Rigifila*, *Micronuclearia*)] Infrakingdom 2. Diacentrida* infraking. n. **Diagnosis:** ancestrally biciliates with centrioles almost antiparallel, anterior centriole forward pointing surrounded by cytoplasmic collar (often lost), posterior centriole pointing backwards at about 150° to it; mitochondrial cristae tubular or absent in anaerobes (excludes opisthokonts). Etymol: Gk *dia* apart + E. *centri-* truncation of centriole. **2 phyla, 9 classes:** Phylum 1. Apusozoa*Cavalier-Smith 1997 em. 2013 (two or three connectors between centrioles; TZ basal cylinder absent) **2 classes:** Thecomonadea, sole order Apusomonadida, e.g *Apusomonas, Manchomonas, Multimonas*: Breviatea (Family Breviatidae Cavalier-Smith 2013, *Breviata*, *Subulatomonas*; new family Pygsuidae **Diagnosis:** biciliate breviates gliding on posterior cilium; type genus *Pygsuia* Brown et al. 2013). **Phylum 2. Amoebozoa** Lühe 1913 stat. n. Corliss 1984 em. Cavalier-Smith 1998 (locomote by usually broad pseudopodia; cristae tubular, often branched) **7 classes:** Subphylum 1. **Tevosa subphyl. n.** Kang et al. 2017 ex Cavalier-Smith 2020 **Diagnosis:** the putative clade comprising Lobosa and Conosa (in the revised sense here, including Cutosea). **6 classes:** Infraphylum 1. Lobosa Carpenter em. Cavalier-Smith in Cavalier-Smith et al. 2004 stat. n. (non-ciliate sole class Tubulinea, e.g., *Amoeba*, *Hartmannella*) Infraphylum 2. Conosa Cavalier-Smith 1998 em. stat. n. [5 classes: unikont Archamoebea, e.g., *Entamoeba*, *Mastigamoeba*; superclass Mycetozoa, e.g., *Dictyostelium*, *Physarum*; Variosea (biciliate, uniciliate, multiciliate, non-ciliate, e.g., *Ceratiomyxella*, *Phalansterium*, *Multicilia*; aciliate Cutosea (*Armaparvus*, *Squamamoeba*)] Subphylum 2. **Discosa subphyl. n**. **Diagnosis** as for **sole class** Discosea Cavalier-Smith in Cavalier-Smith et al. 2004 p. 43 (no cilia: Flabellinia, e.g., *Vannella*; Centramoebia, e.g., *Acanthamoeba*) **Infrakingdom 2. Opizoa** infrak. n. **Diagnosis:** unicellular or oligocellular opisthokonts with single cilium **(developmentally younger; older reduced to procentriole)** used for swimming (when points posteriorly) or generating water currents for feeding, not for gliding (sometimes lost); ancestrally flat mitochondrial cristae; mostly phagotrophs without vegetative cell walls. **Etymol:***opi* truncation for euphony of *opistho* Gk posterior + Gk *zoa* life, to signify they are the unicellular opisthokonts other than fungi and Myxozoa. Phylum 1. Choanozoa*Cavalier-Smith^1981^ em. 1983 (ancestrally free living, swimming or sessile, not gliding, uniciliates (or secondarily aciliate), and mostly filose pseudopods; cristae mostly flat; if parasites no specialised host-invasion machinery) **4 classes** Subphylum 1. Choanofila* Cavalier-Smith 2013 (**3 classes**: Choanoflagellatea with feeding collar of filodigits: Filasterea with radiating filodigits; Ichthyosporea now expanded to include Corallochytrida (*Corallochytrium*, *Syssomonas*)** as well as Ichthyophonida and Dermocystida, often walled, sometimes filose) Subphylum 2. Paramycia Cavalier-Smith 2013 em. (filose amoebae: sole class Cristidiscoidea: orders Nucleariida Cavalier-Smith, 1993 e.g., *Nuclearia*, *Vampyrellidium*, *Pompholyxophrys*, *Lithocolla*; Fonticulida Cavalier-Smith, 1993 e.g., *Fonticula, Parvularia*) **Phylum 2. Opisthosporidia phyl. n.** Karpov et al. (2014) ex Cavalier-Smith, 2020 (**Diagnosis:** opisthokont parasites with chitinous spores with specialised extrusive host-invasion apparatus; vegetative cells naked, sometimes phagotrophic) **4 classes** Subphylum 1. Rozellidia subphyl. n. **Diagnosis:** posteriorly uniciliate naked cells that bore though cell walls of fungi or pseudofungi, enter their cytoplasm and phagocytose it from within; unlike in *Aphelidium* intracellular phase not surrounded by host cell membrane. Aerobes with irregular mitochondrial cristae (class Rozellidea Cavalier-Smith 2013 em.) Subphylum 2. Aphelidia subphyl. n. **Diagnosis** as for its sole order Aphelida Cavalier-Smith 2013 p. 155 (class Aphelidea Gromov 2000) (uniciliate aerobes with flat/tubular cristae) Subphylum 3. Microsporidia Balbiani, 1892 stat. n. (classes Microsporea, Metchnikovellea) (aciliate anaerobes with mitochondria reduced to tiny acristate mitosomes).	

*Probably paraphyletic groups

**Cavalier-Smith et al. (2016) accepted four orders of infrakingdom Archamoebae within a single class Archamoebea. Bayesian 325-gene trees put *Rhizomastix* as sister to Pelomyxidae (*Mastigella*/*Pelomyxa*) (Kang et al. 2017) and this clade as sister to Mastigamoebidae, and there is no good morphological reason not to include them all in one order. As the deepest archamoeban divergence is between mastigamoebae and Entamoebida (see also Pánek et al. 2016 using only seven proteins but more archamoebae), their simplest classification would have just two orders: Entamoebida Cavalier-Smith, 1993 and another for all mastigamoebae including *Pelomyxa* and *Rhizomastix*. However there has been no agreement as to the most suitable name for this mastigamoebid order, as four different ones have been used: Rhizo-flagelleta Saville Kent, 1880; Rhizomastigida Bütschli, 1884 (then spelled Rhizomastigina); Mastigamoebida Frenzel, 1887 (then spelled Mastigamoebaea); Pelobiontida Page 1976, which originally did not include any classical mastigamoebae, and was restricted to *Pelomyxa*, thus most distant from modern concepts. Hardly anyone has used Saville Kent's name since, so it is inadvisable to revive it. Rhizomastigida has the longest continuous use as an order; Doflein used order Rhizomastigina (attributed to Bütschli) comprising mastigamoebae and *Cercobodo*. Minchin (1922) ranked Rhizomastigina as a suborder to embrace the genera *Mastigella*, *Mastigamoeba*, and *Mastigina* with single centrioles, thus excluding biciliates like *Cercobodo*, establishing a well defined monophyletic assemblage of mastigamoebae, and considered them related to Mycotozoa and Sarcodina and grouped them in the suborder Holomastigina (*Multicilia*) in an order Pantostomina (based on Saville Kent). Precisely the same three genera were accepted as order Rhizomastigina or Rhizomastigida (both spellings use) by Hyman (1940). It was demoted to family Mastigamoebidae by Chatton (1953) within order Amoebaea but reverted to order Rhizomastigida in Honigberg et al. (1964), but muddled by implicitly adding unrelated flagellates, ignored altogether by Levine et al. (1980) but retained as an order by Lee et al. (1985) who illustrated only the genera included by Minchin and did not realise that mastigamoebae were related to *Entamoeba* and both related to *Pelomyxa*; Lee et al. (1985) mistakenly called Rhizomastigida 'arteficial', and Bovee (1985) still had only *Pelomyxa* in Pelobiontida (within class Lobosea, then polyphyletic through including Schizopyrenida also) but kept *Entamoeba* in order Amoebida. Ptáčková et al. (2013) were incorrect to assert that Rhizomastigina 'was standardised to Rhizomastigidae by Calkins (1901)'; his much broader invalid family lumped with four others (unrelated) as order Monadida merely confused things. Cavalier-Smith(1983) first grouped all then known anaerobic amoebae (including explicitly *Entamoeba* and *Pelomyxa*) as phylum Archamoebae, only later making separate classes Mastigamoebea and Pelobiontea (Cavalier-Smith 1987a), and then two explicit orders (Cavalier-Smith 1991c). By contrast Griffin (1988), apparently unaware of this, moved mastigamoebids into order Pelobiontida, unassigned to class or phylum. My early papers used Mastigamoebida Frenzel as the primary order for mastigamoebae (Cavalier-Smith 1991c, 1993c) or treated Mastigamoebida and Pelobiontida as separate orders of class Archamoebea (Cavalier-Smith et al. 2004), as *Rhizomastix* Alexieiff, 1911 was not then suspected to be an archamoeba. *Rhizomastix* was ignored by Lee et al. (1985, 2002), first considered a possible archezoan by Cepicka (2010); Rhizomastigida (then containing *Rhizomastix* only) was later added to Archamoebea (Cavalier-Smith 2013), confirmed by sequence trees Ptáčhová et al. (2013). TZ and centriolar root ultrastructure now makes it clear that *Rhizomastix* has fundamentally the same body plan as classical mastigamoebae (Zadrobilková et al. 2016) so they all should be one order. Kang et al. (2017) used Mastigamoebaea Frenzel, 1982 to include both classical mastigamoebae and *Rhizomastix*, which would be appropriate if ranked as class but suffix–ida is needed to denote an order. Several authors (e.g., Brugerolle 1991a, 1991b; Mylnikov 1991) have followed Griffin's wider interpretation of Pelobiontida than Page's as a substitute for both Rhizomastigida and Mastigamoebida. That broadening of Pelobiontida and choosing the youngest of four available ordinal names ignored priority principles preferring older more widely used names; it was also poor taxonomic practise to adopt a name originally referring solely to the most atypical genus a group rather than a morphologically more typical one such as *Mastigamoeba* or *Rhizomastix*. Neither reason given by Ptáčková et al. (2013) for rejecting classical Rhizomastigina in favour of compositionally unstable Pelobiontida is sound. First, the fact that Rhizomastigidae Calkins 1901 (which actually is historically older than that: see Cavalier-Smith and Scoble 2013) is not a valid *family* name (because it was not based on and did not include *Rhizomastix*), though true, does not invalidate *order* Rhizomastigida (or suborder Rhizomastigina) as a descriptive name under the International Code of Zoological Nomenclature (ICZN). Second, saying that composition of Rhizomastigida has 'always been confused' was exaggerated given Minchin's clarity, now confirmed by ultrastructure and sequences. 'Pelobiontida' repeatedly changed in concept and circumscription, so may be regarded as more radically confused. To correct the invalidity of the 19th century family, I established a new family Rhizomastigidae with *Rhizomastix* as type (Cavalier-Smith and Scoble 2013 p. 336; electronically published 4 December 2012). Ptáčková et al. (2013) independently erected replacement family Rhizomastixidae Ptáčková et al. 2013, but being electronically published over a month later (9 January 2013) it is an invalid junior synonym. Therefore the valid name of a family restricted to *Rhizomastix* is Rhizomastigidae Cavalier-Smith 2012 in Cavalier-Smith and Scoble 2013, not Rhizomaxidae. Contrary to Ptáčhová et al., 2013, ICZN Recommendation 29A is inapplicable to this case as there was no previously available valid name based on any genus, so there is no established homonym to risk causing confusion, so it was not necessary to use the whole name rather than the root in forming the family name. I here formally include now valid Rhizomastigidae in order Rhizomastigida Bütschli 1884 stat. n. Doflein 1916 em. Cavalier-Smith 2020, together with families Pelomyxidae (*Pelomyxa*, *Mastigella*), Tricholimacidae (*Tricholimax*: sometimes included in Pelomyxidae, perhaps correct), Mastigamoebidae (*Mastigamoeba, Mastigina*) and Endolimacidae (*Endolimax*, *Iodamoeba*, *Endamoeba*); there is no sound reason to suppress monophyletic family Endolimacidae merely because they evolved from Mastigamoebidae by ciliary loss or at present to split Mastigamoebidae into two families. Ptáčhová et al. (2013) wrote that by the principle of priority if entamoebae prove to be 'sisters to the pelobionts, then the correct name for the clade would be Archamoebida Cavalier-Smith 1983', but that is incorrect as it ignores suprafamilial Linnean ranks. I have never used that precise name nor ranked archamoebae as an order as its suffix implies, always treating them at least as a class for which the spelling Archamoebea is best

***There was no need for a new clade name Pluriformea for *Corallochytrium* plus *Syssomonas* (Hehenberger et al. 2017). *Syssomonas* should just be put in existing order Corallochytrida Cavalier-Smith, 1995 but in a separate **new family Syssomonadidae** [**Diagnosis:** differs from Corallochytriidae Cavalier-Smith in Cavalier-Smith and Allsopp (1996) by having naked phagotrophic phases with a single latero-posterior cilium and/or filopodia as well as walled cysts; can form cell clumps. Type genus *Syssomonas* Tikhonenkov, Hehenberger, Mylnikov, and Keeling in Hehenberger et al. 2017]. This illustrates how monotypic orders can become non-monotypic by discoveries of new genera that can be readily slotted into an existing Linnean hierarchy without disturbing it, often needing no name changes even to suffixes. Aversion to monotypic taxa is irrational, dogmatism against them harmful. However, a separate class for Corallochytrida is unnecessary now, given its robust relationship with filopodial flagellate *Syssomonas*, and the robust grouping of *Corallochytrium* as sister to the two ichthyosporean orders (Grau-Bové et al. 2017). It is sounder to transfer thus revised Corallochytrida to Ichthyosporea as a third ichthyosporean order, given the robust grouping of all three (Grau-Bové et al. 2017) and the rather continuous distribution of characters across the here revised class, and to abandon class Corallochytrea which new species discovery and robust phylogeny has made unnecessary. Recognising only these three choanofilan classes also makes the new supraclass name Teretosporea (Grau-Bové et al. 2017) redundant.

+ Expanding circumscription of this class to embrace all other Percolozoa under the same unranked name (e.g., Adl et al. 2019) has been seriously confusing; I have therefore shown key characters for each class to illustrate the superiority of having *several classes* within Percolozoa

The flared base of the *Metromonas* funnel is held in place by fine filaments linking it to a peripheral dense diaphragm proximal to TP. *Katabia* also with type II TZ (Fig. 4M) has a similar diaphragm, but this does not have an obvious hub linked to its very slender TP; instead it has hub with similar lateral filaments to *Metromonas* substantially proximal to the diaphragm. Section Fig. 4I from a series of *Katabia* TSs (Karpov et al. 2003a) includes both the very base of the TZ with the central hub and the immediately underlying acorn-V filaments, and in conjunction with the LS of Fig 4M gave the first (previously overlooked) good evidence for the acorn-V in Rhizaria. It is as convincing as the evidence from the type I TZ of *Pseudotrichonympha* (Fig. 10R, with the TP lattice superimposed on the acorn-V) cited by Geimer and Melkonian (2004) and accepted as evidence for acorn-V in Protozoa and more convincing that the evidence they cited for a chytrid fungus. Comparison of Figs 4B and M shows that which structures are superimposed on the acorn-V vary with the structure of the TZ, and the evolutionary separability of the hub and lattice in Cercozoa. *Chlamydomonas* was ideal for the first discovery of the acorn-V as it is so distant from the major TZ structures: evolution of the proximal stellate structure and the long linker connecting it from acorn-V moved TP far away.

*Metromonas* proves that a single hub structure can be both proximal and distal to the TP; comparison with *Katabia* suggests that its broad proximal hub region can indeed be proximal to TP in some Cercozoa, but does not prove that was true for *Sainouron* and *Helkesimastix*. Though *Sainouron* was ideal for first discovering the cercozoan hub and spoke structure because its hub is thicker than in *Helkesimastix* (Fig. 4H), *Katabia* or any other Cercozoa, its extremely compressed type I TZ and ultrashort centriole make it bad for determining the axial arrangement of TZ structures as all are telescoped into just one or two sections and superimposition makes it hard to distinguish basically distinct structures or even their relative position axially. The peripheral lattice structure of *Bigelowiella* and the fainter lattice of the same substructure represents a TP skeletal peripheral lattice conserved across corticates, which is generally only ~8 nm thick, whereas the hub-spoke system of *Sainouron* is about 45 nm thick; we can now see why the peripheral lattice was so much fainter in Fig. 4F. It is five or six times as thin as the hub-spoke complex, so the lattice should be at least five times fainter than the spokes, which is what we see. Therefore I conclude that the lattice is simply at a different level, either proximal or distal to the hub/spokes.

I originally assumed that *Sainouron*'s prominent hub-spoke structure (Fig. 4F) was proximal as it appeared to be in direct contact with the centriole, but now consider it proximal to the TP identified here for the first time by careful reexamination of *Sainouron* TZ in LS (Fig. 4N, O). This reveals just inside the doublets, in line with the base of the hub, two triangular densities linking the doublets to the lateral rods. I now suggest these previously overlooked dense triangles are the flared lateral edges of the TP, as clearly shown in most heterokont TPs illustrated by Hibberd (1979), such as e.g., *Uroglena* (Fig. 1C), and which also occur in Cercozoa, e.g., *Katabia *(Fig. 4M). Thus *Sainouron*'s TP is actually level with the thin septum at the base of the hub in Fig. 4O, which I suggest represents the TP itself. In other words *Sainouron*'s hub is not proximal to the TP but immediately distal to it. Therefore the peripheral lattice (as part of TP) is also proximal to the hub, not at the same level as originally assumed. If hub and spokes are actually distal to the much thinner TP and in direct contact with its upper surface, the helkesid TP is not missing as we previously supposed. I have now detected TP homologues in all discarian lineages on Fig. 11, implying that TPs were never lost. Fig. 4O also shows that the lateral rods of the hub-spoke complex extend proximally further than does the hub and thus must pass right through the TP as do the lateral rods in *Metromonas*, which project both from its lower and upper surface (Fig. 4D, E). Further corroboration of this new interpretation is that it means that the ac linkers (called upper transitional fibres in Cavalier-Smith et al. 2008a, 2008b) are offset distally from the TP by exactly the same amount as in heterokonts and other Cercozoa such as *Massisteria* (Fig. 4K), which offset pattern is clearly the ancestral condition for corticates, and also for all discarian eukaryotes as it occurs in many protozoa including the euglenozoan *Bodo borokensis* (Tikhonenkov et al. 2016).

If the *Sainouron* hub is distal not proximal, it can justifiably be considered homologous with the distal hub of *Picomonas* (Fig. 7F, 10W) and *Rhodelphis* (Fig.1 D). Therefore distal hubs are not restricted to Rhizaria. Furthermore within Rhizaria comparison of *Katabia* and *Sainouron* implies that rhizarian hubs may be distal or proximal to TP. It may be necessary to use EM tomography to sort out which of the taxa listed in Table 1 of Cavalier-Smith et al. (2018) have distal and which proximal hubs. The TZ in Cercomonadida is so compressed that they are hard to interpret, but LSs of *Cercomonas dacytloptera* and *Eocercomonas ramosa* (Karpov et al. 2006 Figs 10H and 13 K) suggest distal hubs; *Eocercomonas* (their Fig. 13D) TS may show a TP lattice superimposed on the acorn-V. *Paracercomonas* has hubs discovered since that table (Cavalier-Smith and Karpov 2012, Fig. 32 is TS of a *P. metabolica* hub-spoke and part of TP lattice; for *P. virgaria* their Fig. 17 LS suggests that the hub is distal as for *Sainouron*). For the glissomonad *Bodomorpha* with a more spread out type II TZ the hub-spoke appears to be distal (our unpublished micrographs).

As explained above, the fractionally oblique section including the TP lattice (about half) of the rhizarian *Bigelowiella* (Fig. 4A) also includes about half of the underlying acorn-V as well as the slight hublike proximal connector to it (perhaps also part of the distal connector to the overlying axonemal plate). The TP of the ciliate *Paramecium* (chromist superphylum Alveolata) has more obscuring dense matrix than the four just mentioned, largely hiding its underlying lattice (Fig. 5M), but one can still see within it densities representing peripheral star points both in phase and out of phase with A tubules and the circumferential filament at their bases, but the central lattice structure is totally hidden by the extremely dense distal cup and massive axosome lodged within it and also shows the very base of the longer cp mt. However comparisons with Fig. 5H and other TSs discussed later lead me to conclude that these star-like features are not integral to the more irregular lattice of the TP but marginally distal to it. In *Sainouron* by contrast, the zone just above the hub-spoke structure that connects it to cp is always so lightly stained (Fig. 4O) that in TS one can only just detect the narrower hub that connects the broad hub to cp (Fig. 4G). The upper TP TS of *Picomonas* TP (subkingdom Rhodaria) shows both the prominent distal hub and the faint outer part of the TP lattice (Fig. 10W), whereas the lower section (Fig. 10X) includes primarily the inner part of the lattice but may also include part of the connector to the presumably underlying acorn-V. The TS of the biliphyte *Cyanophora cuspidata* is not confused by overlying dense structures as the cp connector is so thin and faintly stained, only by the TP being strongly domed so different levels are seen centrally and peripherally (Fig. 6B*). The TP TS of the cryptist *Hatena*, though less clear (Fig. 8D level I), can also be interpreted as showing a similar lattice with the same three rings of star points (and central zone denser) superimposed on a fine irregular mesh-like background as discussed for all the other corticate groups. Therefore I have now found evidence for the same three-fold TP star-lattice structure in all four major lineages of Chromista and all three major lineages of Plantae. Prior to the present paper most authors did not even recognise that the TP had any substructure, considering it just a simple uniformly dense plate - and often muddled up different non-homologous plates as explained for Hacrobia and ciliates.

## TP lattice ultrastructure conserved across Corticata

If Viridiplantae evolved from Biliphyta, as argued above, what is their TP substructure? Many of the few micrographs are low resolution, but *Picomonas* has a simple thin TP with an upper broad cup into which the double dense axosome of the cp fits via an ill-defined less dense central element—overall somewhat like that of ciliates but with much slenderer axosome. *Rhodelphis* and glaucophytes both appear to have two plates, not easy to interpret. Gawryluk et al. (2019) labelled the *Rhodelphis limneticus* upper plate tp in Fig. 1D (their Fig. 1r) but contradictorily called the lower plate tp in Fig. 6T (their Figs 1q, where both plates are distorted, and also their extended Fig. 1e). Which is right? One possibility is that the upper plate, which in Fig. 1D appears trilaminar, the thin middle layer being denser, is TP and the lower plate is the acorn-V radially asymmetric plate. These two are linked by a clear axial hub in Fig. 1D, by a narrow hub and two lateral links in Fig. 7L; a thinner broad more distal hub joints the upper plate to cp. The two plates are even closer in *Cyanophora* and in *C. cuspidata* are clearly joined by multiple linkers and behave as unit when distorted, presumably by pulling upwards centrally by the cp, which is joined to the upper plate by a thin tube (Fig. 6A) that superficially resembles the central filament of haptophytes. I prefer the second possibility that the lower dense plate is actually TP and the upper one a secondary plate that may be termed the (sub)axosomal plate as it is immediately below cp, and that in most biliphytes the acorn-V is too tenuous to be seen easily, especially if it is stuck directly to the base of TP, except in *Cyanoptyche* (Fig. 6M) and *Glaucocystis geitleri* (Fig. 6G) where it seems distinct. One reason for this choice is that it puts TP level with the TFs not above them. More importantly it makes the most prominent hub distal, as it is in *Picomonas* and glaucophytes not proximal, so all biliphytes would then have a concordant axial pattern for their most obvious hub, which would have simplified their evolutionary diversification. A third more indirect point is that on this interpretation the connector between TP and cp is bipartite as shown above for the long one of Haptista, a rather close outgroup of Plantae.

Figure 6B* of the *Cyanophora cuspidata* TP is the only TS that shows lattice substructure of glaucophyte motile cilia, but contrast is low because much almost as dense matrix fills in the lattice spaces. Fig. 6H is a TS through the reduplicated putative TPs of the *Glaucocystis geitleri* pseudocilium, which lacks background dense matrix, so shows essentially the same lattice structure in great contrast, albeit somewhat disorganised. Oversimplifying somewhat, one can interpret this as three concentric rings of star points, the two outer ones acute and strongly stained and the innermost ones more weakly stained and more obtusely pointed, roughly nine in each ring. Compare this with a tomogram of the *Chlamydomonas* TZ (Fig. 10M) which also primarily consists of three rings, each of 9 outward-pointing star points, the innermost being the 18-sided proximal basal cylinder, the middle the canonical A-tubule-linked star points and the outermost the offset interdoublet star points. This identity of pattern cannot possibly be a coincidence. It proves for the first time that the common ancestor of Viridiplantae and Glaucophyta had a TZ skeletal lattice of three concentric star point sets with these very properties. Intriguingly the innermost star of *Glaucocystis* has a single eccentric dense granule just as does the innermost star of the *Chlamydomonas* tomogram located at one of its inner lattice junctions; even the *Cyanophora* TP (Fig. 6B*) has one eccentric granule denser/larger than the others. That implies that the innermost obtuse star of *Glaucocystis* also has a similar inner lattice even though one can see only about three of its filaments.

That I suggest is the fundamental principle of corticate TP organisation. An evolutionary consequence of this discovery is that the fundamental pattern of the immensely complex, seemingly unique green plant ciliary stellate pattern and basal cylinders evolved much earlier in ciliated eukaryote evolution and was latent (as the next section shows) in almost all corticate eukaryotes, making its apparently unique origin a much more comprehensible evolutionary event. Manton (1964) was right to imagine that the complex green plant stellate pattern that fascinated me ever since starting research that year (Cavalier Smith 1967) may have lessons for all cilia.

## Heterokont TZs less distinct than once thought

In heterokont algae reviewed by Hibberd (1979) the simplest TPs lack additional structures and show only a slight radially symmetric hub-like swelling (*Pseudopedinella* that unusually for heterokonts lacks a TH; his Fig. 17). Most heterokonts not only have a conspicuous TH distal to TP but also seldom discussed or even mentioned distal and/or proximal hubs attached directly to (or extensions of the TP: Fig. 1C, 7M, 10R-V). Usually the upper and lower TP-associated heterokont hubs differ in dimensions (often the proximal is narrower and shorter) and staining, as exemplified by the proximal and distal hubs of heterokont Pseudofungi which not only differ from each other but also between oomycetes *Phytophthora, Saprolegnia* (Fig. 10T-V) and hyphochytrean *Rhizidiomyces* (Fig. 10S) representing the two classes. Their function has never been discussed, but I suggest that both hubs effect attachment to the radially symmetric TP of asymmetric adjacent structures: the upper hub mediates linkage to the cp and lower hub to the acorn-V-system, recognised here for the first time in heterokonts (Figs 1C, 7M in ochrophytes; Fig. 10T, U in oomycetes, which have particularly conspicuous TP-lined hubs, distal larger; Fig. 41 of Barr and Allan (1985) is probably a TS of *Rhizidiomyces* acorn). Heterokonts as one of the two major outgroups of Rhizaria, are particularly germane to the question whether hub-lattice structures first identified in helkesid Rhizaria may actually be more general in Harosa than previously assumed.

If TP-linked hubs are generally present in heterokonts, as are hubs previously assumed to be mostly proximal (but now seemingly mainly distal) in Rhizaria, both types of hub may also have been present in the ancestral harosan, which I have now shown must have had the star-containing peripheral lattice. We can unambiguously deduce that a lattice similar to that of *Bigelowiella* was present in the ancestral harosan. Fig. 10O is a TS through the TP of *Platysulcus*, the most deeply branching heterokont (Shiratori et al. 2015; Thakur et al. 2019), which lacks a TH and also noticeable hubs. Its TP is not amorphous but composed of a lattice of peripheral, open, acute, star-point filaments, both in phase and out of phase with the A tubules, plus a central starfish like structure very similar to that of the haptophyte *Prymnesium* (Fig. 10N); this central starfish zone also has a lattice that is more obscured by dense matrix than that of *Chlamydomonas* (Fig. 10L). Comparison of TZ lattices of the viridiplant *Chlamydomonas* (kingdom Plantae) and both subkingdoms of Chromista (haptophyte *Prymnesium* and heterokont harosan *Platysulcus*) clearly shows that they are fundamentally the same (Fig. 10L, N, O respectively), proving that the TP filamentary lattice is homologous across Chromista and across superkingdom Corticata. The precise appearance of the lattice at the TP centre inevitably differs because except in *Chlamydomonas*, relying on a very narrow tomographic slice, normal EM 'thin' sections are so thick that they will all include additional superimposed structures involved in TP attachment to the distal cp and/or proximal acorn-V. Thus *Prymnesium* Fig. 10N shows also the central granule that connects the filament linking TP and cp. *Platysulcus* (Fig. 10O) whose mode of attachment to cp is not obvious from LS shows a slenderer apparently hollow tubule at its centre, likely to be cp connector. Only the *Chlamydomonas* tomogram is completely clean showing only the inherent TP lattice with no more distal or proximal structures.

Multiprotein trees show that the immediate outgroup to Corticata is probably Hemimastigophora (Lax et al. 2018). *Hemimastix* (Foissner et al. 1988) has extremely short, chamfered centrioles but rather complex TZ over twice as long that I discuss below when briefly considering corticate outgroups. I noted earlier that *Hemimastix* has slender concentric fibres/basal cylinder (which I argue later may be related both to the cercozoan nonagonal fibre and heterokont TH), and might also have a hub-spoke structure resembling that of *Sainouron* (Cavalier-Smith et al. 2008a); combining that with our belief at the time that TZ nonagonal fibres and hub-lattice was restricted to Cercozoa were reasons for tentatively including Hemimastigida in Cercozoa, which sequence trees disproved. However, as I have now shown that similar hubs are present in *Picomonas*, Retaria, and apparently some Heterokonta, and that nonagonal fibres are even more widespread, both structures likely preceded the origin of Corticata and are not Cercozoa-specific. *Hemimastix* also has a TP lattice quite similar to that of corticates and I argue that all eukaryote TPs must have a lattice substructure, which is related to but simpler than that of corticates plus Hemimastigophora.

To test my novel interpretation further we need EM tomographic studies of selected ciliates, heterokonts and cercozoa in comparable detail to those for *Chlamydomonas* (O'Toole et al. 2003). I predict that they will reveal a common peripheral lattice structure throughout corticates and will also reveal an underlying common skeletal structure for the central TP disc, similar to *Chlamydomonas*. However testing the idea of homology across all corticates with require molecular and genetic dissection of the TP skeleton and associated hubs and lattice structure in several representatives of each group. In principle I expect core components of the TP to be conserved across all corticates, and indeed all eukaryotes except malawimonads, but that more peripheral components (above and below TP) would undergo more radical change and/or replacement by different macromolecular systems as the TZ lengthens or shortens in diverging lineages with different mechanical arrangements or geometries of the cell (e.g., having a wall with tunnels in *Chlamydomonas* relatives or not; or with cilia projecting from the cell apex or else being deeply embedded in a reservoir as in euglenoids; coadaptation will necessarily change TZ dimensions and mechanics in evolution).

## Bell-shaped Labyrinthulea transition rings: clues to halvarian TZ evolution

Most algal heterokonts (ochrophytes) have a TH (a broad cylinder surrounding both cp mts, typically attached to the TP: Hibberd 1975), whereas Pseudofungi have a positionally equivalent set of double stacked rings (Barr and Allan 1985) resembling a concertina in LS often called a double TH (Karpov and Fokin 1985; Cavalier-Smith and Chao 2006) and also found in some phagotrophs in Opalozoa and Bigyromonada. In Labyrinthulea (saprotrophic thraustochytrids plus labyrinthulids) these cylindrical structures are replaced by an inverted bell shape and only one cp fits inside its dome (Kazama 1980; Barr and Allan 1985). All three structures are often assumed to be evolutionarily related and derived from a common ancestral heterokont TH, but it has not explicitly been shown how that could have happened (Karpov and Fokin 1995; Cavalier-Smith and Chao 2006; Cavalier-Smith 2018). Figure 5P,Q of the thraustochytrid *Schizochytrium aggregatum* stresses the single cp is fixed within its cup-shaped bell dome in a fundamentally similar way to that of *Paramecium* (Fig. 5A) where a single cp mt also nests within a dense cup attached to TP's centre. I therefore argue that a single mt/axosomal cup complex with TP was ancestral for all halvaria and modified in different lineages as explained below.

Overlooked features of the thraustochytrid bell are first that its structure is axially complex and divided into three structurally distinct zones and that the bell's rim is fixed to the doublets opposite the annular connection (ac) and ciliary constriction. Other heterokonts with TH or cylindrical transitional rings have ac immediately distal to TP, whereas in Labyrinthulea ac is substantially distal to TP, separated by the height of the bell. The mid region of the *Schizochytrium* bell is a short cylinder with double wall and zigzag pattern indistinguishable from that of Pseudofungi, and I suggest ancestral to the pseudofungal zig-zag double transitional rings. Distal to this is a short cylinder with a single wall, like that of the ochrophyte single TH, and I suggest ancestral to them and to the single basal cylinder of some Opalozoa. The bell's inverted dome plus the zigzag region that embraces the single mt are essentially similar to the axosomal cup of *Paramecium* and I suggest ancestral to it. Thus an ancestral halvarian with the tripartite bell-shaped structure of *Schizochytrium* could have generated the ciliate pattern and the single and double TH patterns of other heterokonts by differential losses of different regions. All these lineages would have lost the bell rim and its connection to ac, which by default would be assembled in the ancestral position immediately distal to TP. The non-labyrinthulean heterokonts would have also lost the bell's dome and thus retained only the central cylindrical region, either in its ancestral double-walled form or retaining only the outer component attached to the A-tubule feet, thus losing the bell shape.

**Fig. 12.**
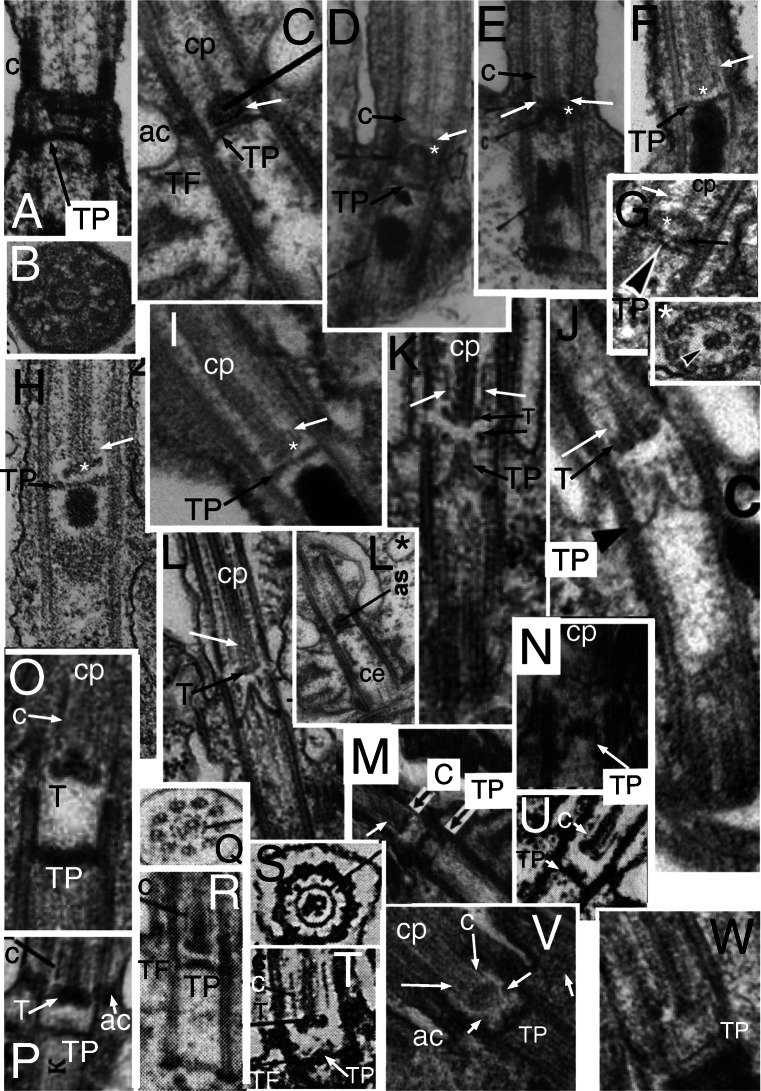
Miozoan TZ diversity (C-T) compared with *Thraustochytrium* (A, B). **A, B.***Thraustochytrium* sp. from Kazama (1972 figs 4, 18) by permission. **A.** LS showing bell-shape is shorter than in *Schizochytrium* (Fig. 5P) and the distal constriction (**c**) wider. **B.** TS showing only a single cp mt within the basal cylinder. **C.***Colponema vietnamica* (Protalveolata: Colponemea) showing single cp mt penetrating the basal cylinder, which (as in *Thraustochytrium*: **A**) has a definite dense base (white arrow) distinct from **TP**—thus is not muff-like (i.e., open at both ends; close-up from L*). From Tikhonenkov et al. (2014 fig. 5A) by permission. **D-I Perkinsozoa:** white arrows mark transverse disc to which both cp mts attach; asterisk marks upper septum of basal cylinder distinct from **TP; c** is thin-walled distal cylinder. **D, E.***Perkinsus* sp. from Coss et al. (2001 Figs 23, 24) by permission. **F-I***Parvilucifera*. **F.***P. rostrata***G** and G*.*P. prorocentri* from Leander and Hoppenrath (2008 figs 40, 48) by permission. **H.***P. infectans* type strain posterior cilium from Norén et al. (1999 Fig. 29) by permission. **I.***P. infectans* RCC2816. F, I. from Lepelletier et al. (2014 figs 5D,G) by permission. **J-N Dinoflagellata** white arrows mark peri-cp cylinder. **J-L. Myzodinea J.***Psammosa pacifica* Okamoto et al. (2012 fig. 4B) by permission. **K, L.***Colpovora* (=*Colpodella*) *unquis* from Mylnikov (2009 fig. 1d) by permission: **K** anterior, **L** posterior cilium. L*.*Colponema vietnamica* centriole (ce) and TZ). **M, N. Peridinea:***Woloszynskia micra* from Leadbeater and Dodge (1967 fig 13, 17). **O-W Apicomplexa, O-V Apicomonadea: O.***Chromera velia* from Oborník et al. (2011 fig. 43) by permission. **P**. *Colpodella pseudoedax* from Mylnikov and Mylnikov (2007 fig. 3.4) by permission. **Q, R.***Colpodella edax* clone BE (type) from Mylnikov et al. (1998 fig. 2B,D) by permission. **S, T.***Voromonas pontica* (as *Colpodella* sp. G-3) From Mylnikov et al. (2000 fig. 2B,D) by permission. **U.***Voromonas* (=*Colpodella*) *pontica* clone G-3(type) from Mylnikov (2000 fig. 1d) by permission. S-U fixed in 2% OsO_4_ + 0.6% glutaraldehyde mixture **V.***Voromonas pontica* from Cavalier-Smith and Chao (2004 fig. 3B) by permission. Fixation in 1% OsO_4_ + 2% glutaraldehyde mixture. **W. Coccidea***Eimeria acervulina* Fernando (1973 fig. 5) by permission

Assertions that the pseudofungal outer ring does not touch doublets (Barr and Allan 1985) and the ochrophyte TH also is not connected (Karpov and Fokin 1995) are incorrect. Linking A-tubule feet are evident in Fig. 5R of the oomycete *Phytophthora parasitica* (from Barr and Allan) and many other published figures. A-tubule feet are necessarily longer in Labyrinthulea as the cylindrical zone is narrower because of the bell shape. But the fundamental similarity is clear by comparing Fig. 5Q (*Schizochytrium*) and 5R (*Phytophthora*): in both the double cylinder connects to A tubules and also has nine thicker knob-like projections alternating with doublets. These must have been present in the common ancestor of oomycetes and Labyrinthulea and confirm that the zigzag zone of Labyrinthulea is homologous with the entire double ring structure of oomycetes. Similar knobs are present in the hyphochytrid *Rhizidiomyces*, where they have an additional narrower distal extension between the doublets (Barr and Allan 1985 Fig. 40) likely a specialised feature of this class. Previously I argued that the common ancestor of all heterokonts had a double TH and all lineages with single TH evolved from them by losing the inner ring/helix. I assumed that bell shaped and cylindrical TZ structures were related but did not understand how. Now, by stressing homology with the sister group Alveolata, I argue that the bell-shaped configuration was ancestral to all Halvaria (Heterokonta plus Alveolata, which are sister groups: Cavalier-Smith et al. 2018) and differential losses of different parts could generate all heterokont and alveolate structures. The bell base (see also the *Thraustochytrium* variants: Figs. 10R, 12A) may be related to the ciliate axosomal cup. The radial interdoublet knobs in Fig. 5R should also be compared with the similar dense radial interdoublet structures in *Chlamydomonas* TZ tomograms (Figs. 10L, M) which might be related.

This unifying interpretation explains for the first time the unusual so-called 'muff-like axosomes' of Colponemea, the most divergent members of Miozoa the sister phylum to Ciliophora (ciliates) (Mignot and Brugerolle 1975; Mylnikova and Mylnikov, 2010; Tikhonenkov et al. 2014). In *Colponema* aff. *loxodes *(Fig. 5N) the dense cylinder at the base of cp is not an 'axosome' but a dense sleeve or cylinder surrounding a central microtubule and attached basally to TP. Distally the rim of this sleeve is extended by a previously overlooked thin, curved, annular lamina linked at its other margin to the doublets immediately opposite the annular constriction and its associated ac. Thus cylinder plus annular lamina are together topologically identical to the labyrinthulean inverted bell in their connections proximally to the TP central zone and distally to the doublets beside a more distal ac. This strongly supports my thesis that ancestral Halvaria had an inverted bell linking proximal TP and more distal ac. Sequenced *Colponema vietnamica* is essentially similar (Figs. 5O, 12C), though the slender lamina/rim of the bell is scarcely visible; calling it 'muff-shaped' (i.e., open at both ends) was misleading (Tikhonenkov et al. 2014) as it is closed at the base by a denser plate much as is the labyrinthulean bell (compare Figs. 5O, 12C with 5P; both have numerous short fine linkers between bell-homologue and TP). I suggest the cup-shaped lamina linking the base of the ciliate axosomal cup to the loose ring (Fig. 5A) is homologous with the curved lamina of *Colponema*, but the ring has lost its ancestral connection to the doublets so ac has moved proximally compared with ancestral halvaria to be opposite TP. Thus the colponemean 'muff' is homologous with the central cylinder of the labyrinthulean bell. I predict that higher resolution micrographs, if less overstained, will reveal an internal double zigzag structure and TSs would show only one microtubule inside it exactly as in ciliates and Labyrinthulea.

Within Miozoa, the basal cylinder split away from TP in subphylum Myzozoa (Apicomplexa and Dinozoa) and widened so that *both* cp mts fit inside it. Apicomonadea (Apicomplexa) have a rather long thin-walled basal cylinder with a bulbous dense torus at its base that surrounds both cp mts, one of which is typically narrower than standard (Fig. 12O-V); this torus is also obvious in Myzodinea (Dinoflagellata) and therefore is an ancestral character for free-living Myzozoa, earlier misleadingly called a 'double axosome' (Cavalier-Smith 2018 supplementary appendix SD4). It is especially distant from TP in the alga *Chromera* (Fig. 12O) but closer in heterotrophic colpodellid-like apicomonads (Fig. 12P, R, T, U, V). Apicomplexa TP is flat and apicomonads have a shallow distal cup or central thickening; in *Voromonas pontica* fixing in stronger glutaraldehyde fixative expands the torus proximally so it no longer resembles a torus and also preserves a pointed structure at the centre of TP cup (Fig. 12V) not visible with stronger OsO_4_ and weaker glutaraldehyde (Fig. 12T, U). In dinoflagellates TP is centrally stretched distalward to form a hat-like structure in Peridinea (Fig. 12N, as in Prymnesiophyceae) or a pointed inverted curved cone in Myzodinea (Fig. 12J-L). Dinoflagellate basal cylinders are long and thin-walled as in apicomonads, but in Perkinsozoa, which have flat TP, they remain squat and thick-walled (*Perkinsus*: Fig. 12D, E) or are variously simplified in *Parvilucifera*, and do not enclose the cp bases, which instead are attached to a septum at the top of the cylinder (which might correspond with the uppermost perforated septum of *Thraustochytrium*: Fig. 12A). In *Parvilucifera rostrata* and *prorocentri* an apparently double cylindrical wall remains for the squat basal cylinder, but appears to be lost in *P. infectans*, whose cp-base-associated structures are bipartite—the more obvious dense disc (Fig. 12H, I asterisk) is likely a relic of the base of the bell-shaped structure, whereas the striated structure (Fig. 12H, I white arrow) at the very base of cp probably corresponds with the cp torus of other Myzozoa, which in the myzodinean *Psammosa* is striated (Fig. 12J). Comparing the TZ of *Perkinsus* with Myzodinea (Fig 12J, L), apicomonads, and *Schizochytrium* suggests the relationship is not simple: *Perkinsus* has *both* a dense, likely double-walled, cylinder proximal to the cp base *and* a single thin-walled cylinder distal to it (c in Fig. 12D, E). The thin-walled upper cylinders of *Perkinsus*, Myzodinea (Fig. 12J-L), and apicomonads (Fig. 12O-V) appear to be directly homologous structurally and positionally, but quite distinct from the *Perkinsus* proximal dense cylinder. The latter is probably homologous with the proximal double zone only of *Schizochytrium* (Fig. 5P) whereas the upper thin-walled cylinders are likely homologous with the thin-walled uppermost part of the thraustochytrid bell only that bends round to join the A tubule in Fig. 5O. Parasitic apicomplexa (Sporozoa) lack cilia vegetatively but some like the coccidian *Eimeria* have ciliated microgametes, which have a squat basal cylinder like that of *Parvilucifera prorocentri* but apparently no distal cp-ensheathing thin-walled cylinder (Fig. 12W). Thus different myzozoan lineages have evolved very different TZs by losing different parts of the more complex ones present in more distant halvarian outgroups. Polyphyletic simplification by loss is evolutionarily more likely than multiple independent innovations.

In the anterior cilium of myzodinean *Colpovora unguis* (Fig. 12K) its cylinder's thin wall also bends round to the doublets giving an overall bell-shape; being the younger first formed cilium its assembly process may better 'remember' the ancestral condition, whereas the older posterior cilium presumably modified it by making the cylinder much longer, so less bell-shaped (Fig. 12L). A functional reason for this difference may be that the posterior ciliary groove is much longer than of the transverse one, probably making it desirable to suppress basal bending/undulation of the cilium over a greater distance by replacing dynein arms and spokes by TZ structures preventing active bending. In eukaryotes generally, the function of evolutionary TZ lengthening is likely to be basal bend suppression, so the convergent changes in TZ length in many lineages may be associated with modified ciliary beating patterns, which are extremely diverse across lineages.

Note also that in Dinozoa *Parvilucifera prorocentri* (Fig. 12G*), *P. infectans* (Norén et al. 1999), *Oxyrrhis marina* (Dodge and Crawford 1971), and *Psammosa pacifica* (Okamoto et al. 2012) one of the cp mts has a thinner mt with fewer protofilaments than the others, just like Apicomonadea (Fig. 12Q, S). The thinner mt lumen is usually filled with dense material. Presence of this exceptionally rare character in both branches of Myzozoa but its absence in major sublineages of both implies that ancestral Myzozoa had one thinner central mt and lineages without it reverted to the ancestral eukaryotic state with 13 protofilaments (reversion was not hard as normal mts were never lost; only the nucleating centre at the base of cp needed changing). I suggest that during the transition from an ancestral bell-shaped TZ with narrow cylinder able to accommodate only one mt to the derived condition with wider basal cylinder able to enclose two normal sized mts (as in Peridinea: Leadbeater and Dodge 1967), Myzozoa went through a transitional stage with intermediate diameter that could accomodate only one normal and one thin microtubule. This change could have occurred by evolving the lumenal plug material at the base of one cp which by acting as a former could have constrained assembly to a smaller tubule; losing this former would automatically cause reversion, independently in Peridinea and Sporozoa. Dimorphic cp is found in *Perkinsus* and *Rastrimonas* also, so is general for Perkinsea, but is restricted to Myzodinea (probably all, but micrographs are ambiguous for *Colpovora unguis*) in dinoflagellates and apicomonads in Apicomplexa, but apparently not found in any other eukaryotes (references in Leander and Hoppenrath 2008). Coevolution with the changing diameter of the miozoan basal cylinder offers a simple explanation of its presence only in Myzozoa but not in all.

## The diatom test case

**Fig. 13.**
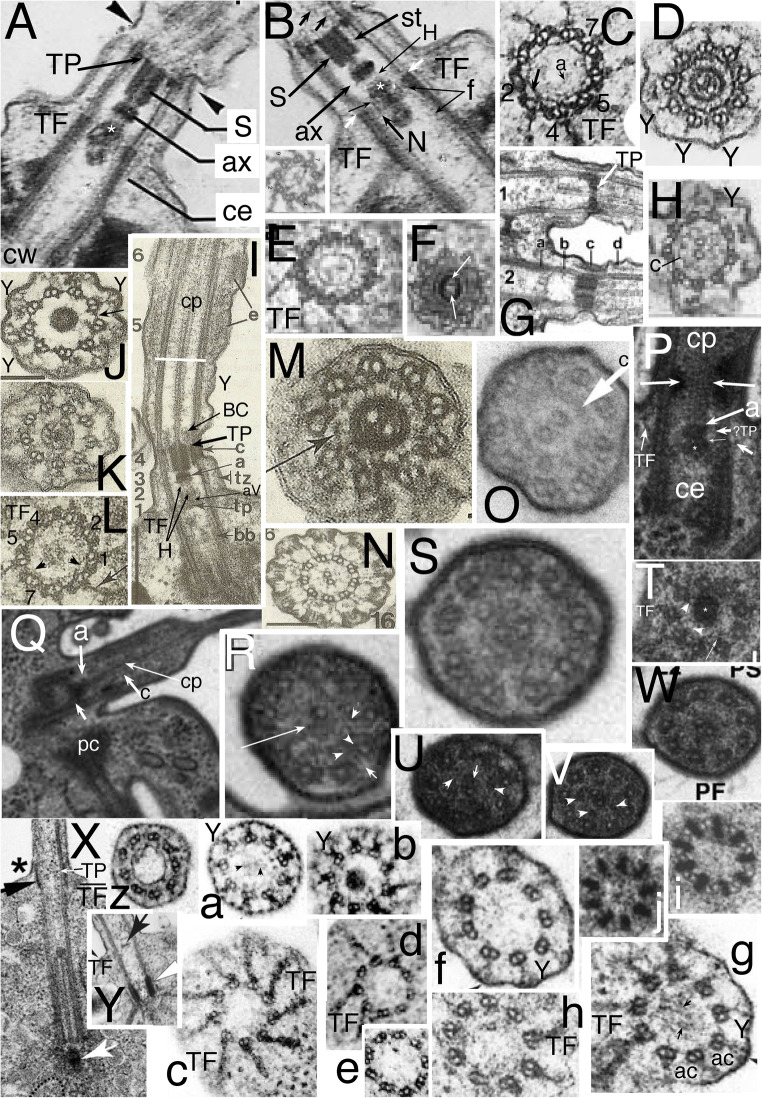
TZ diversity in Sulcozoa (A-N Diphylleida, L-W Planomonadida) and diatoms (X-g). **A-D.***Collodictyon triciliatum* from Brugerolle et al. (2002 Fig. 1d, e, g) by permission. **A, B.** Cilium 2 (**A**) and cilium 1 (**B**) LSs of same cell broken at constriction (arrowheads in **A**). **S** sleeve around cp; **ax** axosome; asterisk second discoid at TZ base linked to **ax** by hub (**H**); **st** peripheral star around sleeve. white arrows mark C tubule end, thin arrow putative thin acorn lattice; **f** centriolar A-B feet; **N** nonagonal tube in prominent centriolar distal plate (clearer in **B**; disrupted in **A** by TZ structures being pushed into centriole lumen). **B inset:***Chlamydomonas* proximal acorn-V lattice from Geimer and Melkonian (2005) for comparison with **C. C.***Collodictyon* TS at TF level includes centriolar nonagonal tube and grazes the putative acorn structure (doublets numbered as in *Chlamydomonas* (**B** inset). **D.***Collodictyon* TS of cp within sleeve and surrounding stellate structure and Y links (Y). **E-H.***Sulcomonas lacustris* from Brugerolle (2006 figs 5+ a, c, d). **E.** TFs at centriole/TZ junction (level a in **G**); likely represents faint asymmetric acorn filaments superimposed on underlying rotationally symmetric centriolar distal ring with radial linkers. **F.** TS of level **c** in **G** showing cp (arrows) tightly enclosed by dense sleeve. **G**. LS (1 tangential, 2 median) of ciliary bases showing absence of complex TZ structures between acorn (just distal to **a**) and TP/constriction(c) levels. **H.** TS through fluted (star-like in section) basal cylinder (**c**) at level **d** in **G** where spokes and arms are absent from doublets, replaced by inner (straight) and outer (V-shaped)A-B links. **I-N***Diphylleia rotans* from Brugerolle and Patterson (1990 as *Aulacomomonas submarina* figs 10-14, 16) by permission*.***J.** LS of TZ; in Y-link zone (Y) below white line doublets lack spokes and arms; **TP** separates distal basal cylinder (BC) and four distinct proximal TZ zone. **c** constriction level with sleeve, **a** axosome, **H** narrow and wide hubs; tp centriolar distal plate. **J-M.** TSs of *Diphylleia* TZ. **J** at level 3 through axosome showing Y-links (**Y**) and single set of A-B links (arrow)**. K** at level 2 embracing narrow (on left) and wide hub (right) junction. **L** at level 1 at TFs and triplet doublet junction (note filled C tubules as in **C**); section includes acorn homologue and base of overlying hub), possibly also grazing underlying centriolar plate hub, superimposed; arrowheads mark putative peripheral acorn filament. **M**. level 4 through sleeve and cp; radial links as **D** but surrounding basal cylinder comprises discrete densities opposite A-B links (arrow) not star-like as in *Collodictyon* (**C**). **O.***Planomonas micra* anterior cilium with basal cylinder (c arrow) around central pair mts (within ciliary pocket) from Cavalier-Smith et al. 2008b fig. 6C by permission. **P-W***Ancyromonas sigmoides* from Heiss et al. (2011 figs 4A, 2D, 3B, C, 5C, D, F, G) by permission. **P.** LS showing central mts (**cp**) surrounded by short dense basal cylinder (long arrows) terminating at an axosome (**a**) connected by asymmetric short links (? sandwiched acorn-homologue?) to dense material (asterisk) in the distal centre of the centriole (**ce**); small arrow marks end of C tubule; less dense thin material (**?TP**) between **a** and doublets may be the peripheral zone of **TP**. **Q.** LS of anterior cilium with central pair mts (**cp**) projecting into acroneme; **a** axosome; **pc** posterior centriole; arrow distal transverse centriolar plate**. R.** posterior cilium TS grazing the axosome (long arrow) to which one cp mt clearly abuts; arrowheads mark circumferential filaments (? zig-zag or starlike) associated with A-tubule feet. **S.** Anterior cilium within ciliary pocket showing upper zone of basal cylinder, i.e., distal to **R** fig. 3C. **T-W.**non-consecutive serial sections of another posterior cilium (B tubule lumens filled; **T-U** within posterior ciliary pocket**)**. **T.** at distal end of centriole; arrowheads mark filaments of its irregular lattice, partially obscured by central dense matrix (*); thin arrow marks A-tubule tubule foot from which lumenal filaments diverge; **TF** transition fibre. **U.** most proximal TZ with only one cp mt contacting axosome (arrow) surrounded by irregular lattice that may represent TP; denser granules (arrowheads) may be parts of underlying acorn or overlying basal cylinder. **V.** at level of basal cylinder of 9 discontinuous granules (arrowheads opposite doublets); 2 cp mts. **W.** in ventral groove after exiting ciliary pocket; some dynein arms appear, cylinder and spokes largely absent; cp mts surrounded by denser zone**. X.** Centric diatom *Melosira monoiliformis* var. *octogona* long centriole with prominent cartwheel in LS. Asterisk marks annular connection, and black arrow the dense material linking Y-link stems axially. **Y.** Centric diatom *Thalassiosira lacustris* LS showing short centriole with very short cartwheel. Black arrow marks dense central zone of TP seen in TS in **g**; white arrow marks interdoublet centriolar dnesities seen in TS in **i,j**. **Z-e.***Melosira* Consecutive TSs through transition zone (**Z-d**) to upper centriole (**e**). **Z** straddles axoneme base and uppermost TZ showing dynein arms, nonagonal fibre, and A-tubule feet. **a**, **b** show Y-link (**Y**) dense bases . **a.** embraces extremely thin TP showing a central pair of densities (arrowheads) where cp would originally have been attached and peripheral links to doublets. **b**. acorn-like structure with axosome-like aymmetric density at base of Y-link zone and double A-B links. **c**. **TF** zone with hints of acorn filaments. **d**. TZ/centriole transition. **e**. upper centriole doublets with single A-B links. **f-j.***Thalassiosira* TSs. **f-h.** Consecutive TSs though lower TZ. **f**. Y-link zone. **g**. section embracing TP lattice with central densities (arrowheads) and transition between Y-links/ac and **TF**s. putative acorn. **i,j.** Consecutive TSs through lower part of doublet centriole showing interdoublet dense rods. Figs X-g from Idei et al. (2013 figs 4b, 5b, c, d, h, i, 7d, 8b,c, d, e, f, g) by permission

Diatoms are the only major eukaryote group to have lost central pair (cp) mts whilst retaining motility (a few animal sperm have also done so). How does this affect the TZ, especially the TP whose primary purpose may have been to anchor the cp? These heterokont chromists lost cilia in vegetative cells; the ancestral centric diatom subgroup retains the anterior tinsel cilium only in sperm, which evolved a radially symmetric cone of mts connecting the centriole base to the nucleus, which is ultrastructurally much simpler than markedly asymmetric ancestral ciliary roots of flagellates. Their closest relatives are the biciliate Bolidophyceae with normal 9+2 cilia and type I TZ with simple TP just above the cell surface and a subsidiary plate just below, with no TH (Guillou et al. 1999). Amongst diatoms *Lithodesmium* (Manton and von Stosch 1966), *Coscinodiscus* and *Chaeotoceros *(Jensen et al. 2003) apparently retain both plates despite having lost the cp ancestrally. Plates, supposedly absent in *Melosira* and *Thalassiosira *(Idei et al. 2013), were overlooked, TP being less dense than usual (Fig. 13a, g). Centrioles have doublets not triplets (Fig. 13e, i, j), so the classical demarcation between TZ and centriole, like that of TZ and motile axoneme cannot be applied. Normal TFs mark the base of the TZ (Fig. 13X, c; faint putative acorns are visible in TS through them (Fig. 13b, c, h). In *Melosira* the changeover between the Y-link region and doublet dynein arms defines the upper limit of the TZ (Fig. 13Z); at this level is a short nonagonal fibre linked to A-tubule feet, which might be a TH relic; proximal to this the Y-links' bases are unusually dense (Fig 13a, b), appearing as a thicker zone in LS (Fig. 13X black arrow). Idei et al. (2013) thought arms to be absent in *Thalassiosira lacustris*, which makes no sense mechanistically and contradicts genetic evidence for *T. pseudonana* ciliary dynein; as none of their TSs showed any region distal to Y-links where arms should begin, I suggest that *T. lacustris* arms are probably only distal to an unusually extended arm-free Y-link TZ zone, perhaps for the basal 5 μm of the cilium which in their fig. 1k phase micrograph is straight below the first bend.

Deep-branching *Melosira moniliformis* has a very long centriole with long cartwheel zone (Fig. 13X), but very short TZ like bolidophytes (Guillou et al. 1999). Its putative TP lattice is very faintly stained but appears to have a central irregular meshwork and about 18 radial links to doublets like many other TPs (Fig.13Y). Surprisingly, as the cp was probably lost before the last common ancestor of diatoms in the early Cretaceous, at the centre of TP are two densities suggestive of sites of cp attachment (Fig. 13a arrowheads). Possibly therefore even after loss of cp mts a paired structure that originally bound the γ-TuRC nucleation machinery persists on the axosomal thickening of the TP lattice. In the TF zone proximal to the TP are asymmetric densities (Fig. 13b, c) similar to the acorn region of metamonads. The TF zone has both inner and outer A-B links (Fig. 13b-d) as in corticates generally.

*Thalassiosira lacustris* has a much shorter centriole with unusual very dense intercalary fibres linking the doublets above its base (Fig. 13i, j). Though stated to lack a cartwheel a very short one appears to exist at the very base partially obscured by dense material (Fig. 13Y, j). The putative TP in the Y-link zone (Fig. 13g) is more obvious with denser central zone with irregular lattice and two structures somewhat smaller than mts that may be relict mt attachment sites (arrowheads), and radial linkers to doublets and A-B links. A putative acorn complex is present proximally to TP in its usual position near the TF bases. Both genera have traces of the annular connection (Fig. 13X asterisk; Fig. 13g,a,c).

Thus despite losing the cp 110 My ago or more the TZ is a standard type I with TP, Y-links, A-B links and acorn complex fundamentally similar to other heterokonts that have lost the TH. Idei et al. (2013) asked what is the selective advantage of losing the cp, considering its evolutionary causes a mystery. The mystery is easily solved by accepting that it was not an advantage but probably harmful, but not so harmful as to outweigh the advantages of being a diatom, which prevented their extinction despite the loss. If sexual reproduction is temporally rare in a population and cilia are lost and unnecessary for vegetative growth, cells can undergo many vegetative generations so non-lethal mutations affecting only sexual stages can spread easily throughout the population either by neutral drift or by genetic hitchhiking on the back of other strongly selected mutations (from which they cannot be separated in the absence of sexual recombination). Therefore so long as the mutation causing cp loss either did not paralyse the cilium (as such mutations normally do in *Chlamydomonas*) or was accompanied or closely followed by a separate suppressor mutation that restored motility without regenerating the cp, the loss mutation would spread during vegetative growth. The situation is analogous to the spread during vegetative growth in inactive ciliate macronuclei of mutations harmfully jumbling gene order and being corrected phenotypically by evolution of unjumbling mechanisms, after which ciliate genomes could get more and more jumbled and descendants were stuck with the situation for ever (Cavalier-Smith 1993b).

Such major changes can occur thus without any selective advantage of the overall change if a harmful mutation spreads by drift or intragenomic processes like duplicative transposition in a sexual population and is mitigated by a positively selected suppressor(s) advantageous only in the presence of the preceding harmful change (s); another example was the origin of spliceosomal splicing to correct phenotypically the most harmful effect of the transpositional spread of introns (Cavalier-Smith 1991a). An analogy is using a false limb to 'correct' amputation: one would be better off without losing the limb and replacing it by a false one. A false limb is not better than a real one, just better than no limb. Thus vegetative loss of cilia by the ancestral diatom allowed (but did not require) cp loss close to diatom ancestry and later also allowed the total loss of cilia in pennate diatoms as they were not essential even for sex; these arguably negative changes were not sufficiently harmful to counteract the huge selective advantage of the frustule of the ancestral diatom and make it extinct. Likewise the ancestral pennate's other advantages enabled it to survive despite losing sperm cilia which did not prevent conjugation. The most successful/speciose pennates are the raphid clade with vegetative raphe-based gliding also used to bring gametes together instead of cilia. Ancestral raphid pennates are immotile and gametes probably mostly meet by chance, but one genus (*Pseudostaurosira*) evolved a retractable non-undulatory mt-containing filament enabling sperm to move actively towards the egg in large steps (Sato et al. 2011). Irrespective of whether its filaments evolved from a highly modified cilium or from cortical mts (more likely), this novel motility like raphid motility can be regarded as phenotypic corrections of the non-lethal harm done when pennates lost ciliary motility. We need not invent any benefit for their losing cilia. Weaker selection for retaining cilia just for a transient life phase is probably why cilia have been lost so often in eukaryotes that vegetatively lost cilia and keep them only for dispersal (many times in fungi and algae; in almost every major amoeboid group, in most seed plants).

## Non-corticate TZ evolution

Corticata (with many phototrophs) plus Hemimastigophora are a clade (Lax et al. 2018), which I call eucorta (Fig. 11). It has been controversial what is the next closest outgroup because of uncertainty where the eukaryote tree's root lies. Traditional views influenced by early prokaryote-rooted rDNA trees tended to place phylum Metamonada and/or infrakingdom Eozoa (=Discoba) (or some of them) at the base of the tree, with which rooted ribosomal multiprotein trees are consistent, but are highly suspect in that specific respect because of likely long-branch attraction towards the inflated stem of all ribosomal trees (Cavalier-Smith and Chao 2020). Rooted trees for eubacterial proteins that entered eukaryotes during mitochondrial symbiogenesis should be more reliable than ribosome-related ones as these sequences differ less markedly from bacterial ones, yet has yielded contradictory results. Some suggest a root between Eozoa/Discoba and all other eukaryotes (He et al. 2014); others suggest one between corticates plus Discoba and all other aerobic eukaryotes (Derelle and Lang 2012) or else have been interpreted as supporting the latter (Derelle et al. 2015) but are in fact contradictory for different protein samples, and as I show below the technically better ones imply a root between phylum Malawimonada and all other eukaryotes as shown in Fig. 11. I also show that TZ structure strongly supports a root between malawimonad protozoa and all other eukaryotes. To demonstrate this I defer consideration of Eozoa and obazoa (opisthokonts and Apusozoa) to focus on Malawimonadida and the three deep-branching eukaryote lineages most often branching closest to them, e.g., on the site-heterogeneous tree of Brown et al. (2018) based on 351 protein sequences, essentially congruent with Cavalier-Smith et al. (2014) using 187 proteins, i.e., the sulcozoan orders Diphylleida and Planomonadida, plus Metamonada.

Diphylleida have a highly derived long type II TZ whose unique substructures were previously wrongly interpreted, but being more spread out are evolutionarily especially informative. Planomonad and *Malawimonas* centrioles are radically shorter and TZs especially compact and not previously satisfactorily interpreted, and are here designated types III and IV respectively. Metamonad TZs are uniformly compact type I. Despite these major differences I show that all except malawimonads probably have a latticed TP as do corticates considered above. I start with members of protozoan phylum Sulcozoa which most trees show are more closely related to opisthokonts and Amoebozoa than are malawimonads and metamonads (Fig. 11).

## TZs of the most divergent dorsates: Sulcozoa

Diphylleida are freshwater swimming flagellates whose exceptionally deep feeding groove divides the cell into two halves and is supported by an unusually complex skeleton that retains many excavate features (Cavalier-Smith 2013) but unlike excavates the groove can emit pseudopodia; together with aciliate (filose) rigifilids they form the deepest branching clade in the ancestrally pseudopodial podiate clade (Fig. 11).

*Diphylleia* (two cilia) and *Collodictyon* (four cilia) constituting family Diphylleidae Cavalier-Smith (1993a) have a particularly complex TZ—note that family 'Collodictyonidae' is a nomenclaturally invalid junior synonym; Brugerolle et al. (2002) overlooked the priority of Diphylleidae. By comparing them with the simpler TZ of *Sulcomonas* (family Sulcomonadidae) I correct earlier interpretations where TP was incorrectly identified.

In *Sulcomonas* and both Diphylleidae the cp base is intimately surrounded by a dense cylindrical sleeve that penetrates all the way through the centre of the TP together with its tightly enclosed cp (Fig. 13A, B, D, F, G). This type of TZ is unique in eukaryotes so I describe it fully for both families which differ in major details. The dense sleeve is a proximal extension of part of TP corresponding to the edge of its usual central disc; the more proximal structure originally labelled transitional plate (tp) in *Diphylleia* and *Collodictyon* (Brugerolle and Patterson 1990; Brugerolle et al. 2002) is actually at the top of the centriolar triplet zone, i.e., positionally between the alveolar plate and acorn-V complex of ciliates, thus not a TZ structure. It is markedly thicker in *Collodictyon* than the other genera. Not only is the sleeve longer in Diphylleidae than *Sulcomonas* but the space between the putative acorn level and TP is filled with extra structures absent in *Sulcomonas* (Fig. 13G). *Sulcomonas* cp barely protrudes from the bottom of the sleeve/cp complex (Fig. 13G cilium 2), thus presumably needs no separate axosome as is present below the sleeve in both Diphylleidae, and which is linked to the centriolar plate by a short narrow hub and a more proximal longer wider one (Fig. 13A, B, I). Furthermore only *Collodictyon* has a dense centriolar body attached proximally to its thickened centriolar distal plate (Fig. 13A, B).

Distal to the sleeve/TP, all Diphylleida have a short TH-like basal cylinder (BC) that appears as a fluted 'cylinder' in *Sulcomonas* TS (Fig. 13H). However as some star points are opposite A-tubule feet and some point between doublets the Fig. 13H section must include two distinct superimposed star-like structures; the LS in Fig. 13G also shows differences in cylinder distal and proximal structures. If my interpretation is correct, then the proximal and distal BC stars of *Sulcomonas* are out of phase by 360°/18, i.e., 20°. In LS *Diphylleia* BC resembles the basal part only of the *Sulcomonas* BC complex (Fig. 13I) and is a zig-zag ring structure with about 3-4 tiers only, similar to the loose ring(s) of *Paramecium* (Fig. 5A, I) and the more densely stained hyphochytrid double ring cylinder (Fig. 10T). *Collodictyon* may have distinct denser basal and more tenuous distal BC regions (Fig. 13B black arrows) like *Sulcomonas*; its cilia seem to break easily between them, so they might be a centrin-based autotomy mechanism like *Chlamydomonas* stellate structures. Distal to TP *Sulcomonas* TZ has two sets of A-B links the outer ones being V-shaped in the same direction as in green plants (Fig. 13H); similar V-shaped links are present proximally to *Collodictyon* TP (Fig. 13D); between doublets and dense sleeve is a dense outer ring of filaments arranged as a 9-fold star with obtuse points that point *between* doublets not at them. It is joined to the inner sleeve by numerous (?27) radial links. This 9-fold star is equivalent to one of the two outer stars of corticates, but has obtuse rather than acute points. No outer star in phase with A tubules is evident in *Collodictyon*, but no TS was shown of TP itself, or for the BC region.

Extra structures proximal to TP of both Diphylleidae are almost the same. *Collodictyon* TP is of standard thinness except for the usual thicker flared edges that contact the doublets, whilst the sleeve (total thickness ~117 nm) projects ~95 nm below it (Fig. 13A, B); its cp protrudes slightly further proximally and is terminated by a dense discoidal axosome. The axosome is proximally linked by a faint hollow hub to a second dense discoid that directly contacts what is likely an acorn-homolgue at the doublet triplet junction (Fig. 13B thin arrow; just visible in TS in Fig. 13C superimposed on centriolar nonagonal fibres). Immediately below this putative acorn is a prominent radially symmetric partition (with central greater density) just below the top of the centriolar triplets and level with the TFs. Below the centriolar distal partition and connected to it by another hub is a dense short hollow hub with a central partition. Oddly Brugerolle et al. (2002) placed their 'transitional plate' label opposite this clearly centriolar hub structure which is neither plate like nor transitional; I assume they intended to label the distal centriolar partition as did Brugerolle and Patterson (1990) for *Diphylleia*.

In *Diphylleia* (originally described as *Aulacomonas*: Brugerolle and Patterson 1990) the discoid immediately above the distal centriolar partition is less dense, so resolved in LS into a wide proximal TZ hub with narrower inner zone. A TS (Fig. 13K) embracing two different levels shows two contrasting structures: the left half shows the TFs and in glancing section associated cytoplasmic bulges, and five C-tubule ends, so must be proximal to the right side which shows Y-links to the ciliary membrane and no C tubules so is marginally more distal. The wider part of the hub on the right side connects to each A-tubule foot by a very slender radial filament. In contrast the narrow hub is connected by radial acute star points (opposite A-B links between the doublets) associated with dense material between linker and membrane. Thus the narrower hub with interdoublet narrow star points must be proximal to the wider hub with simple filaments linking it to A tubules. Therefore diphylleid proximal hubs contains a short region within which are interdoublet acute star points indistinguishable from those of the upper septum of the proximal stellate structure of *Chlamydomonas* in addition to the obtuse star patterns associated with the TP-attached sleeve. Thus Diphylleidae evolved two axially separate star sets independently of Viridiplantae. Note that if one were to compress both the proximal and distal stars into a single plane they would appear to form a structure with 18 star points separated by 20° and directed alternately towards and between the A tubules, which was my interpretation of the *Chlamydomonas* Fig. 10L tomogram. The densities between sleeve and doublet are less star-like in the *Diphylleia* TS (Fig. 13M) than in *Collodictyon* (Fig. 13D); this may not be a species difference but might reflect different levels of section along the sleeve (13D is below the constriction; M on the right overlaps it).

Planomonads are notably harder to interpret than Diphylleida because the TZ is more compresssed making superimposition a greater problem, and because in Heiss et al. (2011) with the best serial sections overstaining often obscures important details. Planomonads have a basal cylinder that surrounds cp in the upper TZ (more distinct in the anterior cilium: Fig. 13O) and has a similar diameter to heterokont TH and viridiplant basal cylinder, i.e., greater than the diphylleid tight sleeve (Fig. 13O-W). However LSs show that its TZ is neither type I nor type II as the putative TP is neither level with nor distal to the plasma membrane, but somewhat below it (Fig. 13P, Q). I designate this rare pattern (I am aware of two other examples in unrelated groups) type III. I suspect that it is a primitive character associated with an extremely short TZ in which cp starts only a few nm above the end of the centriolar C tubule (Fig. 13P). Both cp mts terminate on a prominent axosome, which appears to be just the central disc of an otherwise very thin TP (Fig. 13P), but serial sections indicate that one must extend further into it (Fig. 13R, U). If C tubules terminate as shown by the small thick arrow in Fig. 13P, the dense disc (asterisk) just below the axosome must be part of a centriolar distal plate (Fig. 13Q) not a TZ structure; the very narrow space between the axosomal thickening of TP and this plate has apparently radially asymmetric material that I suggest is an acorn-homologue, but there appear to be no published TS of that precise region, so its exact structure is unknown. The basal cylinder appears partially (Fig. 13O) or completely subdivided into distinct fibres one opposite each doublet rather than being made of continuous rings like the diphylleid sleeve. It appears to begin just distal to TP (as in Viridiplantae or heterokont TH) but is less dense basally; it has radial linkers to cp and may be associated with a relict ac (which is not accompanied by the usual constriction; though the whole of the ciliary membrane base is very close to the doublets, Y-links not being obvious, unlike Diphylleida). In TS the putative TP (Fig. 13T, plus superimposed on one cp mt in 13R, U) appears to have an irregular lattice and to have little similarity to the peripherally star-like TPs of corticates (except for their irregular lattice in basal cylinder septa); though there is too much dense matrix to be sure, there is no reason to think it differs radically from the TP of Diphylleida. The central dense axosomal zone of TP (Fig. 13R,T) has a star-like outline and central lattice rather similar to that of *Prymnesium* and *Platysulcus* (Fig. 10N, O).

## TZs of the earliest branching natates: metamonada

For metamonads I focus primarily on the genetically divergent anaeromonad metamonads *Trimastix* and *Paratrimastix* (order Trimastigida), free-living phagotrophs likely less radically altered from the ancestral metamonad condition than the more numerous, often much modified, non-phagotrophic parasites. Trimastigida are tetraciliates ancestral to oxymonad parasites; their strongly vaned posterior cilium associates with a prominent feeding groove with typical excavate cytoskeleton*. Paratrimastix* are freshwater sisters of oxymonads, whereas marine *Trimastix* is more distantly related to both (Zhang et al. 2015).

**Fig. 14.**
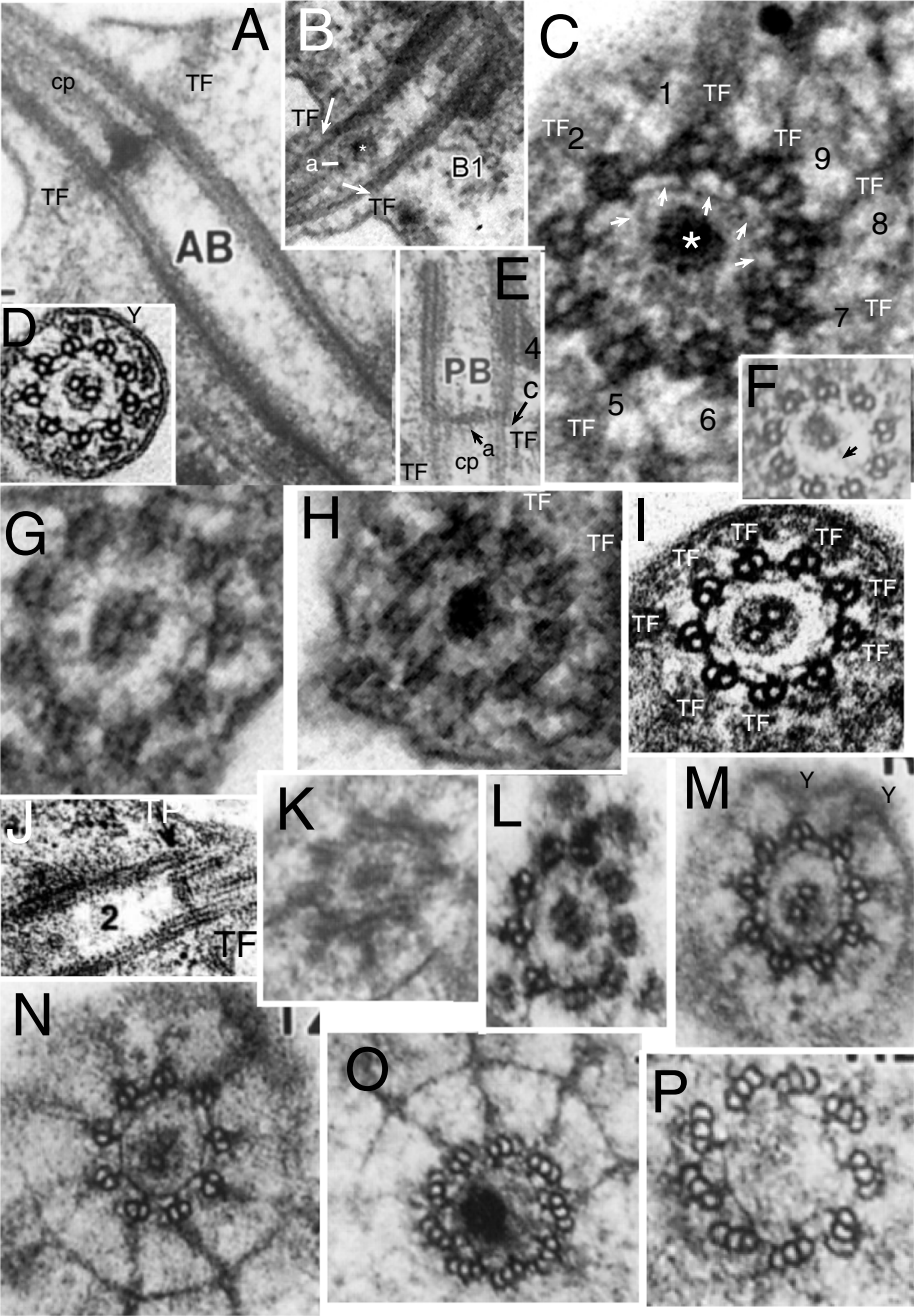
Metamonad TZ diversity: Trimastigida (A-D, F-O), Fornicata (E). **A.***Paratrimastix eleionoma* axosome/TZ structure bell-shaped in LS; **AB** anterior centriole. **B.***Trimastix marina* posterior cilium LS; axosome (**a**) terminating cp is separated from centriolar distal central density (asterisk) by less dense asymmetric material (acorn-complex in **C**); arrows mark ends of C tubules. **C.***Trimastix marina* anterior centriole/TZ junction with six triplets and three doublets (numbered as in Geimer and Melkonian 2004); arrows mark peripheral and part of lumenal acorn filaments, overlying centriolar density (asterisk). **D.***Paratrimastix pyriformis* distal TZ with Y-links(Y) and fluted basal cylinder surrounding cp. **E, F. Fornicata:***Carpediemonas membranifera* posterior cilium TZ LS and anterior cilium TS of upper TZ; **c** end of C tubule; **a** axosome; arrow, possible lumenal acorn filament. **G, H.***Trimastix marina* anterior cilium adjacent serial sections; **G** upper TZ including axosomal zone around cp, **H** lower TZ/centriole junction. **I.***Paratrimastix pyriformis* TZ showing outer starlike lattice with tenuous connectors to denser axosome. **J.***Paratrimastix pyriformis* tangential LS. **K***Paratrimastix eleionoma* anterior cilium TZ. **L.***Paratrimastix eleionoma* posterior cilium upper TZ. **M.***P. eleionoma* right cilium TZ with nonagonal fibre and axosome. **N-P.***Paratrimastix eleionoma* consecutive serial TSs of right cilium base (**N**), TZ/centriole junction (**O**), and distal centriole (**P**). A, K M-P from Simpson et al. (2000 Fig. 2h, j-m, 3b); B, C, G, H— from Zhang et al. (2015 figs 7E,F, 8C,D); D, J from O'Kelly et al. (1999 figs 24, 7);. E, F from Simpson and Patterson (1999 fig. 2i, j) by permission

Both lineages have long centrioles but an exceptionally short TZ, the simplest of any discaria, so short that TSs necessarily also include parts of the central pair or the centriolar apex. Above I called it type I but, as O'Kelly et al. (1999) pointed out, their TP is very slightly recessed below the level of the plasma membrane (Fig. 14A, J) so they have a slight resemblance to the type III TZ of planomonads and type IV of malawimonads. *Trimastix marina* (Zhang et al. 2015) has particularly clear TZ acorn filaments (Fig. 14C) immediately underlying the TP, whose skeletal lattice is star-like peripherally; V-filaments could be present but cannot be seen against underlying centriolar densities. *Paratrimastix eleionoma* (originally wrongly equated with *Trimastix marina*: Simpson et al. 2000) has more dense matrix pervading the TZ and adjacent axonemal and centriolar regions making their boundaries indistinct (Fig. 14A) but serial sections separate substructures (Fig. 14N-P); the transition from central pair mts to the first two centriolar tiplets occurs within one section (Fig. 14N) as does the transition from TZ nonagonal fibre to normal doublet spokes and arms (Fig. 14L). *Paratrimastix pyriformis* has the least TP-associated dense matrix (O'Kelly et al. 1999) so its lattice substructure is exceptionally clear (Fig. 14D, I). In all three species cp mts terminate at the same level at the TP.

The distal part of TP of *P.* (=*Trimastix*) *pyriformis* where it directly contacts cp has a very clear outer star whose denser (obtuse) points join the A-tubules inner projections; its denser central 'axosomal' lattice is mostly obscured by cp but is bounded by a distinct but very thin peripheral nonagonal filament (Fig. 14D). At least some radial filaments link the vertices of the nonagon to the axils between the star points—more are visible in Fig.14D than I, so it is reasonable to suppose there are actually nine but their extreme thinness makes them hard to preserve or stain; O'Kelly et al. (1999) called the central disc of TP an axosome, but in my view it is simply a very slightly thickened central part of TP, *not* a distinct structure like the classical axosome of ciliates (Fig. 5H) or those of Diphylleida. In *Trimastix marina* one can see parts of the same 9-fold star point structure despite less good preservation and at least one radial filament pointing to a star-point axil (Fig. 14G). Therefore this outer star plus central elongated nonagon structure is arguably ancestral for Anaeromonadea. In both genera A-B links are much thicker than the star and nonagonal filaments at the TP level.

However, the TP lattice is somewhat different and more complex in *Paratrimastix eleionoma.* The filaments connecting the A-tubule feet are thicker and straighter than the V-shaped star filaments of the other species making the structure more nearly resemble a nonagonal fibre (or tube as the same structure extends over at least four adjacent serial sections: Fig. 14N-P) so is both distal and proximal to both TP and acorn. Instead of radial filaments between the central nonagon and these filaments are thicker and shorter linkers between the centre of each nonagonal fibre and A-B links centres. Three such filaments are actually visible in *P. pyriformis* Fig. 14D and two or three in 14I. Because matrix is denser in *P*. *eleionoma* than *pyriformis* we cannot exclude the possibility that star-axil radial filaments are present in *P. eleionoma* but longer and hidden by matrix*.* The simplest explanation is that in all three species these radial filaments extend all the way from the central nonagon to the centre of the A-B links but are elastic or contractile and that differences in relative tension on either side of the star axil can alter the shape of the star or potential star. Contracting the inner part would make an 18-sided star shape, contracting the outer part would change it to a nonagon with each side subdivided by the radial filaments*.* It is well known that *Chlamydomonas* TZs contain the contractile protein centrin. If radial filaments contain centrin and can be differentially controlled in the radial direction (either physiologically or developmentally during TP assembly) the alternative morphologies of the two *Paratrimastix* could be interconverted. Alternatively they might be developmentally fixed and interconvertible only evolutionarily by modifying their morphogenesis.

**Fig. 15.**
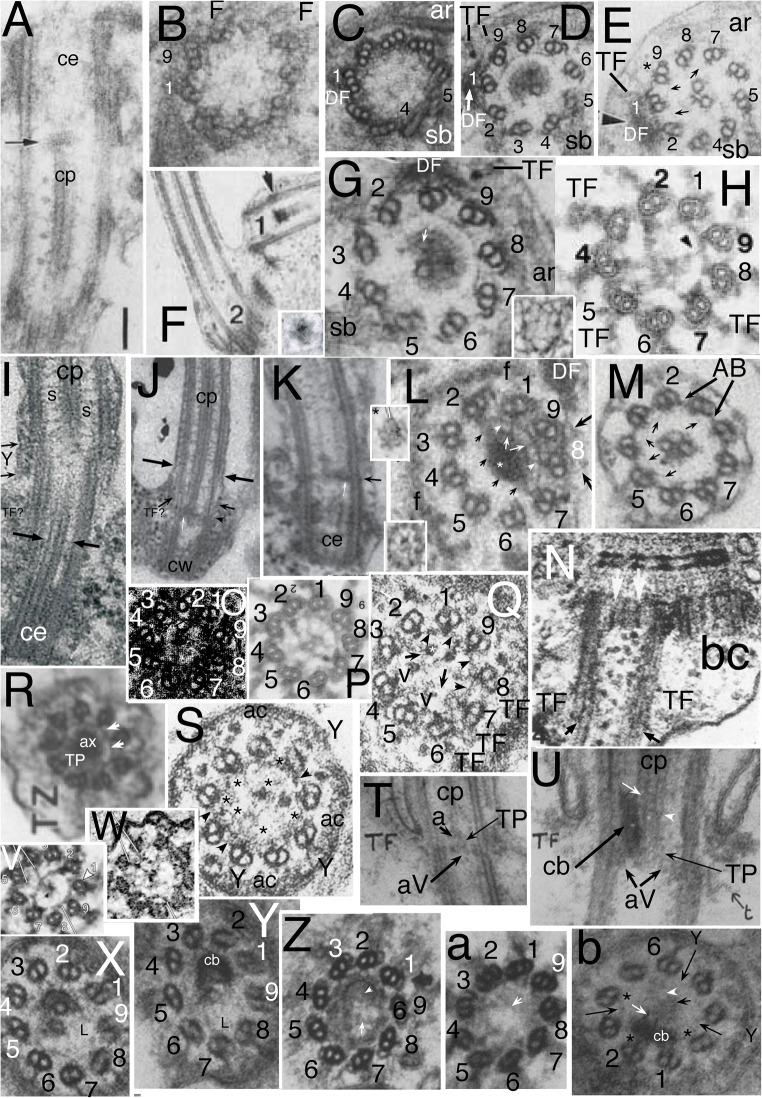
Malawimonada transition zones (A-G *Malawimonas* I-M *Gefionella*) compared with divergent outgroups (fungi, parabasalian metamonads and a rhizarian); four insets show magnified TZ central lattices from diverse natate lineages). **A-G***Malawimonas jakobiformis* from O'Kelly and Nerad (1999 figs 10, 11-14) by permission. **A.** axosome (arrow) at cp base; note absence of TP between it and centriole (**ce**)l scale bar 250 nm. **B-E** consecutive serial section though anterior centriole (**B** proximal cartwheel and anterior fan (**F**) mts, **C** distal with dense fibre **(DF)** and striated band **(sb)** connectors to posterior centriole), TZ (**D**), and 9+2 axoneme base (**E**), showing absence of a symmetric TP lattice and transitional fibres (TF) only on doublets abutting plasma membrane. **D.** (enlarged and rotated in **G** for comparison with **H;** arrow marks grazed base of second cp mt.) shows semicircular filament linking A-tubule feet of doublets 7-9, 1, 2 only (numbered assuming homology with *Chlamydomonas* acorn peripheral filament in **H**). In **E.** arrows mark faint filaments linking five doublets, 7-2) only. **F.** LS through antiparallel centrioles showing TZs (arrow) recessed below the cell surface on their inner linked sides. **Inset***Calkinsia* (Euglenozoa: Postgaardea) axosomal filamment TS magnified from Fig. 17C. **G inset.***Chlamydomonas reinhardtii* irregular coarse lattice of distal septum of proximal TZ basal cylinder; tomogram from O'Toole et al. (2003 fig. 3C) by permission**. H.***C. reinhardtii* acorn-V complex from Geimer and Melkonian (2004 fig. 2E) by permission. **I-M***Gefionella okellyi* from Heiss et al. (2018 figs 3a, 2a, g) by permission. **I.** Oblique LS medially through axoneme cp and tangentially though posterior centriole (**ce**) showing termination of C tubules (arrows) and Y-link zone (**Y**) thus TZ and centriole much longer than *Malawimonas*. **J.** Median LS shows cp starts below cell surface. A ciliary constriction (large arrows) is barely visible, putative TFs diffuse, and basal attachments of cp asymmetric (thicker/denser on one side: white arrow) and level with putative TF bases; arrowhead indicates C tubule ends; **cw** cartwheel. **K.** LS confirming asymmetry of cp basal attachment (white arrow marks thicker side), arrow marks likely C tubule end. Inset *Viridiraptor (Rhizaria) axosome lattice magnified from Fig. 17T**. L. M.** consecutive TSs from a series of five spanning posterior centriole, TZ and spoked 9+2 axoneme. **L.** Proximal TS with peripheral acorn filament and lemon-shaped axosomal plate with dense irregular lattice linked by radial connections to acorn filament; large black arrows mark triangular linkers to membrane from doublets 8, 9, shorter than typical TFs or Y-links, small ones the lumenal edge of the axosomal plate that may hide a lumenal acorn filament; asterisk marks hint of a mt base and position to which V filaments from doublets 4 and 5 converge in discarian TZs; **f** fibrillar arc to which doublets 1-5 attach by putative Y-links). **M.** distal TZ with cp, A-B links (**AB**), and tenuous circumferential filaments linking double ends of A-tubule feet (arrows); membrane linkers shorter for doublets 8,9. **Lower inset.** Lattice from *Chlamydomonas* distal basal cylinder's distal septum tomogram for comparison (magnified from Fig. 10K). **N.***Olpidium brassicae* (Fungi: Zygomycota: Zoomycetes) barren centriole (**bc**) and ciliated centriole attached to common striated rhizoplast showing TFs and end of C tubules (arrows). **O.***Phlyctochytrium irregulare* (Chytridiomycetes) acorn-like structure from McNitt (1974 fig. 3) by permission. **P.***Chlamydomonas reinhardtii* acorn-V complex slightly more proximal than **H** from Geimer and Melkonian (2004 fig. 2E) by permission. **Q.***Olpidium brassicae* TS of TZ base; acorn-V clearer than in **O**; note peripheral acorn filaments (arrowheads), lumenal acorn filament (arrows) and putative V-filaments (**V**); doublets (3, 5, 6 with faint/partial C tubules) numbered as in **P**; the granule (asterisk) absent in *Chlamydomonas* might be underlying centriolar as in **N**. **R.***Stygiella* (= *Jakoba*) *incarcerata* (Eozoa, Jakobea) from Simpson and Patterson (2001 fig 3A). Fuzzy section showing TP/axosomal plate (lower left) apparently superimposed on partial nonagonal fibre (arrowheads)**.***Olpidium brassicae* more distal TS than **Q** at Y-link (**Y**) level including ?partial/grazing coarse irregular lattice (asterisks) slung from doublets, A-B links, and A-tubule feet. **S.***Olpidium brassicae*. TS of TZ including or grazing the TP, showing its irregular lattice (asterisks) that occupies the whole area within the A-B links (arrowheads);**ac** annular connexion. **T, U, X-Z.** Metamonada: Parabasalia. **T.***Holomastigotoide*s TZ LS; the putative amorphous **TP** is immediately beneath the axosome (**a**) terminating cp and underlain by an asymmetric acorn complex (aV). **U.***Trichonympha* showing asymmetry of cp attachments; the longer mt (white arrow) is attached laterally to the crescentic body (cb) and basally to the putative TP which immediately overlies the putative acorn-complex (aV) **V, W.***Viridiraptor invadens* (Rhizaria, Cercozoa) TSs of separate TZs showing acorn-V at slightly different levels: **V** similar to *Chlamydomonas* in Fig 3F. **W.** more like slightly more proximal *Chlamydomonas* in **P**. **X-Z.***Pseudotrichonympha* serial sections through TZ showing crescentic body (**cb**) and acorn filaments (in **Z**); **L** faint lattice. **Z.** Acorn-V superimposed on extreme base of longer cp mt (arrowhead) and likely also peripheral star-like boundary of TP; arrow marks Y-system beside the granular junction of arms of putative V-filaments (compare with **V,W**). **a,***Trichomympha* TS at TZ/centriole junction showing asymmetric acorn-like complex. **b.***Trichomympha* more distal TS; **cb** crescentic body. N, Q, S from Lange and Olsen (1976 figs 4, 13, 15), T-Z, a, b from Gibbons and Grimstone (1960 Fig. 8 figs 56, 32, 23/4) by permission)

In serial TSs the most conspicuous part of the peripheral lattice of this structure (visible in four consecutive sections: Fig. 14M-P, best in M, N) is a nonagonal fibre attached to the A-tubule feet, each filament of which bears a central granule. The first two sections are TZ, one through the narrow part of the bell, the second through its rim where it contacts the A tubules, though this shows two triplets so might have part of the acorn, which probably extends partly into the third (Fig. 14O) with nine centriolar triplets which has an eccentric elliptical density like that of *Trimastix* (Fig. 14C) suggesting that a dense eccentric acorn-aligned matrix may be characteristic of ancestral Anaeromonadea distal centrioles. The second section also embraces the very base of cp suggesting cp is embedded in the centre of TP similarly to Diphylleida, but unlike them does not extend proximal to it being absent from the third one whose eccentric roughly semicircular density must closely underlie TP, and likely to be the same eccentric density as underlies (or occupies) the lumen of the *Trimastix* acorn (Fig. 14G, H). Note also that in the same section as the parabasalian *Pseudotrichonympha* acorn (Fig. 15Z) extra TP eccentric dense material overlies or underlies part of the basal lumen of its acorn; thus parts of TP and or centriolar matrix partially obscure the acorn-homologue so I cannot be sure that complete V-filaments are present, even though there are densities in all the right places; but one can see a filament in the position expected for the stem of the Y that crosses the acorn, implying that the V-filament system is complete. As Parabasalia are in the other major branch of metamonads from anaeromonads it follows that ancestrally metamonads had a really short TZ with TP in direct contact with the acorn and likely had dense matrix close to the acorn.

Thus like planomonads but unlike Diphylleida TP is directly above the acorn-V. The continuation of the nonagonal fibre structure between the very base of the TZ and top sections of the centriole means that centriole and TZ are less differentiated at their junction than is true of Corticata and many other groups. Furthermore, anaeromonad TZ is less differentiated from the 9+2 axoneme: the cp occupies the entire TZ of *Paratrimastix* and *Trimastix* distal to the acorn. This might also be true of Planomonadida if their longer cp mt penetrates to the base of the axosome; though extreme density of the relevant sections (Fig. 13P, T) makes it hard to be sure exactly where this mt ends. Section 13T, devoid of cp, is probably best interpreted as having doublets and through the axosome, which is apparently just the extremely dense central zone of TP (asterisk); if the mt simply abuts the upper face of the axosome the planomonad TZ is that much thicker than in most metamonads, but if a mt penetrates into it but is hidden within the central density it would be no thicker than in metamonads. *Carpediemonas*, a free-living fornicate metamonad (sister group to Parabasalia) has an ultrashort TZ (Simpson and Patterson 1999) immediately overlying a dense distal centriolar plate (Fig. 14E, F); its TP stains very lightly except for the central 'axosomal' zone abutting the cp (one mt appears longer; Fig. 14F) which is linked even more tenuously to the peripheral 9-fold star of its TP for which A-tubule feet form the points.

Parabasalia also have very similar extremely short TZ (Fig. 15U, X, Z, a, b) but the genera studied by Gibbons and Grimstone (1960) somewhat differed. In *Holomastigotoides* the inconspicuous amorphous TP is closely sandwiched between a small cp axosome and the asymmetric putative acorn complex (Fig. 15T). *Trichonympha* lacks an obvious axosome; its cp base is asymmetric: only one mt directly contacts the TP and is laterally connected to 2-3 outer doublets by a dense crescentic body absent in most TZs (Fig. 14U, b) but positioned similarly to one sector of a basal cylinder/TH; the shorter cp mt connects to TP via a filament roughly half the length of the crescentic body. *Pseudotrichonympha* serial sections show that the crescentic body adjoins doublets 1 and 2 at the broad end of the underlying acorn; Fig. 14Z shows that the longer cp mt is probably nucleated just inside the inner edge of the broad acorn (or just distal to it; only tomography could establish which). It appears that compared with *Chlamydomonas* whose cp is very distant from the acorn, that this corner of the acorn lumenal filament may bulge out more towards triplet 3 to enable it to encircle the mt base. Because Fig. 14Z straddles the doublet/triplet boundary it is hard to tell whether the partially obscuring amorphous dense matrix (with a star-like periphery, points at the A tubules) is TP or distal centriolar. The amorphous lattice (L) in Fig. 14Y (and more faintly in 14X) may represent TP.

## Uniquely simple malawimonad transition zones

The aerobic biciliate malawimonads (*Malawimonas*, *Gefionella*), once classified with metamonads as Loukozoa (Cavalier-Smith 2013), are sister to metamonads on 159-protein trees excluding planomonads (Heiss et al. 2018) but branch nearer planomonads on 351-protein trees with wider taxon sampling of Sulcozoa: sister to planomonads by ML and to Varisulca by PhyloBayes, though when rapidly evolving sites are removed they group with metamonads (Brown et al. 2018).

*Malawimonas jakobiformis* (O'Kelly and Nerad 1999) has the shortest and simplest TZ of any eukaryotes (Fig. 15A, F) whose structure puzzled me for decades. Four serial TSs of the anterior ciliary base (Fig. 15B-E) show that its fully triplet centriole fits into just two thin sections, the proximal showing A-tubule pinhead and cartwheel (Fig. 15B), the distal with nine A-B feet and irregular lumenal filaments (Fig. 15C), and that a cp mt plus nine A-tubule feet are present in the very next section (Fig. 15D, enlarged and rotated in G); thus on the classical definition of the TZ there is none—its TZ is best defined as the zone containing A-tubule feet instead of arms and spokes. One cp mt begins even before the triplets fully end as Fig. 15C appears to graze the very base of the longer cp mt. The next two sections (Fig. 15D, E) exhibit both nine partial C-tubules (centriolar structures) *and* nine A-tubule feet (TZ characters) thus have an overlap axially of classical centriolar and TZ characters, and lack spokes and dynein arms; as D has an asymmetric axosomal plate with one cp mt and D has two cp mts with projections but no plate there is also axial overlap of TZ and (+2 characters. There is neither a complete TP nor a complete acorn-V complex, nor complete sets of A-B links, Y-links or TFs. Instead a peripheral filament attaches to the A-tubule feet of five doublets bearing a partial C tubule (essentially half the acorn), and the axosomal 'half plate' attaches laterally to that filament by extremely tenuous filaments. The axosomal plate *might* hide a tenuous lumenal acorn filament between doublets 2 and 7; magnified in Fig. 15G; it lacks radial symmetry and consists of medium density matrix with an irregular supporting lattice that somewhat resembles the irregular lattice of the distal septum of the proximal basal cylinder of *Chlamydomonas* shown in the inset. One cp mt base is embedded at the flat edge of this 'half-plate'. Thus like the centriole the entire TZ occupies the thickness of only two sections with different substructure; here the cp is normal but doublets lack arms and spokes. In both sections the axoneme is substantially recessed from the plasma membrane. There is no adjacent ciliary membrane for attachment of Y-links or TFs except for the middle doublet of the five acorn-bearing doublets (i.e., doublet 9, which in natate eukaryotes is connected to the base of the Y of the acorn-V complex). Triplets are labelled following Geimer and Melkonian (2004, 2005), assuming the peripheral filament linking five of them to be homologous with the peripheral acorn filament of *Chlamydomonas* (Fig. 15H).

As functions associated with TFs and Y-links must be essential for ciliary development, I suggest they are present not on all *Malawimonas* doublets but *solely* on doublet 9 of each centriole. At the level of the half plate a fibre runs from the plasma membrane, between an anterior fan mt and some microfibrillar material to join the B tubule of doublet 9 (Fig 15D); I suggest this is a TF, possibly the only one for the anterior centriole. In the immediately distal section, doublet 9 has a faintly stained link directly between its A/B partition and the plasma membrane, as do Y-links (asterisk Fig. 15E); this is likely a Y-link, perhaps the only one. I designate as type IV this exceptional TZ with an acorn peripheral filament and asymmetric axosomal half plate radially linked to it at the same level, no V-filament system or symmetric TP, and Y-links only on doublet 9 and TFs only on doublet 8. There appear to be A-C links between most or all the partial triplets of the proximal TZ (Fig. 15G); but A-B links are visible only between 7 and 8 and 4 and 5. The distal TZ has only two A-C links, between 1, 2, and 9. The *Malawimonas* TZ differs radically from the largely rotationally symmetric TZs of discaria, exhibiting extreme rotational asymmetry for TFs, Y-links, A-B links, all with 9-fold symmetry in discaria. The centriole may be attached to the surface membrane additionally via more diffuse amorphous fibrillar material associated with adjacent doublets 1, 2 that may connect to the membrane between the anterior fan mts. A section in the middle of a series through the posterior cilium TZ shows essentially the same though being slightly oblique is less clear. A third series through both centrioles (O'Kelly and Nerad 1999) is also concordant for both, so this peculiar structure was present in three separate serially sectioned cells, so cannot be a developmental aberration of one cilium.

*Gefionella okellyi*, maximally distant from *Malawimonas* on multiprotein trees within the deep malawimonad clade, has longer centrioles but also with abnormal radially asymmetric TZ spanning (at least) two serial sections (Fig. 15L, M) both with nine A-tubule feet (Heiss et al. 2018). Being more strongly stained, the lattice connecting the asymmetric lemon-shaped axosomal half-plate to doublets 1, 2, 7-9 is more obvious than in *Malawimonas*, but essentially similar (Fig. 15L). These five doublets only are mutually linked by four A-C links, all absent in the distal TZ (Fig. 5M). The putative acorn peripheral filament is more obvious, clearly continuous and attached to five doublets by 11 linkers of three types, but as in *Malawimonas* shows no sign of a circumferential filament or V-filaments. In contrast to *Malawimonas*, partial C tubules are present only on four acorn-filament-linked doublets in the proximal TZ (8, 9, 1, 2; Fig. 15G) and absent in the distal TZ (Fig. 15M). All five acorn-filament-linked doublets have different links to lumenal and surrounding structures. End doublets 2 and 7 each have two closely spaced links to the peripheral acorn filament: an A-tubule foot and an AB-foot. That supports acorn peripheral filament homology with *Chlamydomonas* whose partial end triplets 2, 7 have two linkers (A-tubule foot and A-B foot for 7, both A-tubule foot and a second A-projection for 2) and doublets 8, 9 have only one (A-tubule feet) and 1 probably has two: an A-tubule and A-B foot (Fig 15H). *Gefionella* distal TZ has nine inner A-B links and nine A-tubule feet. Its proximal TZ has obvious AB-links only on both sides of doublets 4 and 9; 9 has a unique radial linker from the acorn filament to the B end of the A-B linker. *Chlamydomonas* middle doublets 1, 9, 8 have linkers to the A-tubule end of A-C links; triplets 1, 9 probably use AB-feet but not A-tubule feet.

Full length TFs were not identified, possibly because some sections may be missing from the series: triangular projections on doublets 7-9 that connect to the membrane, may be rudimentary TFs, much shorter than usual; moreover doublets 1-5 also have similar triangular projections that connect instead to an unusual dense microfibrillar arc, one end of which is connected to the centriolar dense fibrous connector (Fig. 15L). The immediately distal section (as in *Malawimonas*) lacks doublet arms and spokes (Fig. 15M, unlike the next one), but has probable Y-links on doublets 2-7 but dissimilar shorter ones on the other three. Thus *Gefionella* TZ may also be regarded as a variant type IV with lemon-shaped axosomal plate (axP) connected by a dozen or so radial linkers to the peripheral acorn filament and more TFs, Y-links, and A-B links than *Malawimonas*, but still asymmetric. In LS axP radial asymmetry is clear (thick on one side, very thin on the other: Fig. 15J, K). If no sections are missing from series e-i of Heiss et al. Fig. 3, then as in *Malawimonas* the asymmetric half-plate/acorn is restricted to one section. There is therefore no doubt that *Gefionella* and *Malawimonas* have genuinely asymmetric half-plates joined at essentially the same axial level to the acorn peripheral filament, and that there is neither a typical dense rotationally symmetric TP nor a V-filament in Malawimonada. In better-stained *Gefionella* the peripheral acorn filament is structurally homologous with that of the complete *Chlamydomonas* acorn: in both links from the end doublets (2, 7) are more prominent than those to doublets 1, 8, 9; they agree in fine details of shape and in having a granule on the mid part of each filament on either side of doublet 9. Both genera have incomplete C tubules in the TZ which may extend throughout its length in *Malawimonas*, but which peter out asymmetrically in *Gefionella* which in several other respects shows more TZ symmetry that *Malawimonas*. A-tubule feet of both malawimonads have a double terminal structure similar to the pinheads of centriolar cartwheels. Distal to the gefionella half-plate are traces of very fine filaments between the A-tubule feet (Fig. 15M arrows) that might be related to the more prominent nonagonal fibres of many discaria. Pinheads of centrioles have a similar tenuous circumferential filament visualised only by cryotomography (Greenan et al. 2018 fig. 4a). These similarities make it plausible that the TZ originated by duplicating pinheads axially (see later discussion).

A problem in interpreting *Gefionella* is that LSs (Fig. 15I-J) are hard to integrate into a single convincing explanation of how cp is attached to the half-plate. Unfortunately Fig. 15K showing an unusual central structure in the basal cp zone is fuzzy; it suggests that the cp might not be directly embedded in the half-plate as is one *Malawimonas* mt, which is consistent with not clearly seeing cp in the TS (Fig. 15L; though a half-hidden mt base might be at the asterisk), though Fig. 15J implies that one cp mt penetrates virtually as far as the top of the centriole. However a single LS can be misleading as shown by *Chlorarachnion reptans* (Hibberd and Norris 1984 figs 65, 66) where one LS shows the cp axosome directly attached to the TP central disc, but another shows it 120 nm away (their Fig. 66) separated from TP by a faint cylindrical structure, presumably matrix protein left behind when the axosome was torn from cp; though the *Gefionella* basal cylinder might be a similar artefact, that is doubtful. These micrographs also make the point that chlorarachnid axosomes are separate structures from the TP, and that TP is distinct from their acorn complex (separated from it by an 8 nm gap).

## Evolutionary implications of malawimonad TZ simplicity

I have shown that malawimonads consistently lack both the V-component of the acorn-V and a radially symmetric TP and have instead an asymmetric axosomal half plate connected to precisely the same five doublets as the acorn peripheral filament. The most basic question is whether their simplicity represents the ancestral state for eukaryotes or is a secondary simplification.

As reviewed above for the deepest lineages (and later for shallower ones), all other lineages appear to have a TP with nine fold symmetry that is at least slightly distal to the asymmetric acorn complex and supported by nine A-tubule feet (universal in eukaryotes) and A-B links (universal in eukaryotes but restricted to some doublets at some levels in malawimonads). In theory one might argue that such a TP was ancestral for all eukaryotes, as previously assumed when discussing ciliary origins (Cavalier-Smith 2014). But that would require that the ancestral malawimonad simultaneously lost the V-filament system *and* either (1) lost TP and evolved de novo an unrelated lemon-shaped axosomal plate, attached it to the peripheral acorn filament lower down, and freshly attached cp to it or else (2) detached TP from the distal A-tubule feet and A-B links and attached it lower down to just 5 doublets and converted it into the lemon-shaped axosomal plate of malawimonads by fundamentally changing its symmetry. That would be extremely complex mechanistically and of no obvious selective advantage; there is also no example of any other eukaryote lineages making such a radical change to their TS. Many have lost cilia altogether but none have certainly retained them and so drastically changed the architecture of both major components of TZ: the acorn complex which defines it base and the TP which provides an anchorage for cp nucleating machinery and in most cases also the mature cp; these structures and functions are essentially independent of each other and almost constant in all other eukaryotes, despite variations in their mutual separation axially and in additional TP-associated structures in some derived discarian lineages. Even diatoms that completely lost their cp did not lose TP or radically change the acorn homologue.

By accepting that *Gefionella* represents the ancestral TZ state and that the eukaryote tree is rooted between malawimonads and discaria we simplify the origin of cilia and the TZ, as it follows that the TP and V-filament system did not have to evolve at the same time as A-B links, TFs, Y-links and the peripheral acorn filament. By having both A-tubule feet and A-B links a *Gefionella*-like early eukaryote was preadapted for evolving a symmetric TP in its present-day axial position simply by polymerising a lattice protein into an irregular mesh at the level of its nine A-B links and supported by them and the A-tubule feet. This would also require that axosomal plate assembly be delayed until after TP assembly, which would ensure that it assembles immediately distal to TP and was attached to it, enabling cp assembly and anchoring to be unaltered by TP origin. Thus cp and acorn became axially separated at the base of the huge clade discaria shown in Fig. 11, which has a primary split into two major subclades: dorsates (ancestrally gliders) and natates (ancestrally swimmers) that differ from each other and from Malawimonada in how their two centrioles are connected. The protein may have evolved from the axosomal lattice protein following gene duplication that allowed it to attach (likely with the help of distinct adaptor proteins) to the A-B links as well as A-tubule feet and also to evolve a finer mesh. If the symmetric TP fine mesh was thus evolutionarily derived from the asymmetric, coarser axosomal mesh there would already have been a variety of proteins involved in attachment to the acorn filament and A-tubule feet that could have been modified similarly for the mechanically improved radially symmetric TP.

The selective advantage of a symmetric TP sheet strung from the 9 doublet supports by 18 possibly elastic connections (9 to A-tubules feet, 9 to the A-B links) like a trampoline is that it would have been a mechanically sounder way of attaching the cp base in a vigorously lashing cilium; a trampoline attached to the frame only on one side like the malawimonad axosomal plate would be dangerous. Thus it represents an engineering improvement not a likely harmful degradation as in the alternative idea of its loss by ancestral malawimonad, which I reject as highly unlikely. I still argue that in the ancestral biciliate eukaryote the posterior cilium was used for ciliary gliding and the anterior one originally pointed rigidly forwards for catching bacteria by adhesion and surface motility as in *Phalansterium* (Cavalier-Smith 2014). That mode of locomotion (gliding posterior, rigid anterior) still characterises planomonads, the most ancient dorsate lineage, whose cilia do not normally vigorously undulate—so a simple *Malawimonas*-like axosome without a TP would suffice. Swimming was a secondarily derived ciliary complication. As I discuss after reviewing additional groups, I found evidence that TPs and acorn complexes may both differ slightly between dorsates and natates. The low diversity of malawimonads (two genera only despite being as ancient as discaria) might stem from their primitive TZ not giving the evolutionary potential for such diverse swimming modes as did the discarian TP.

**Fig. 16.**
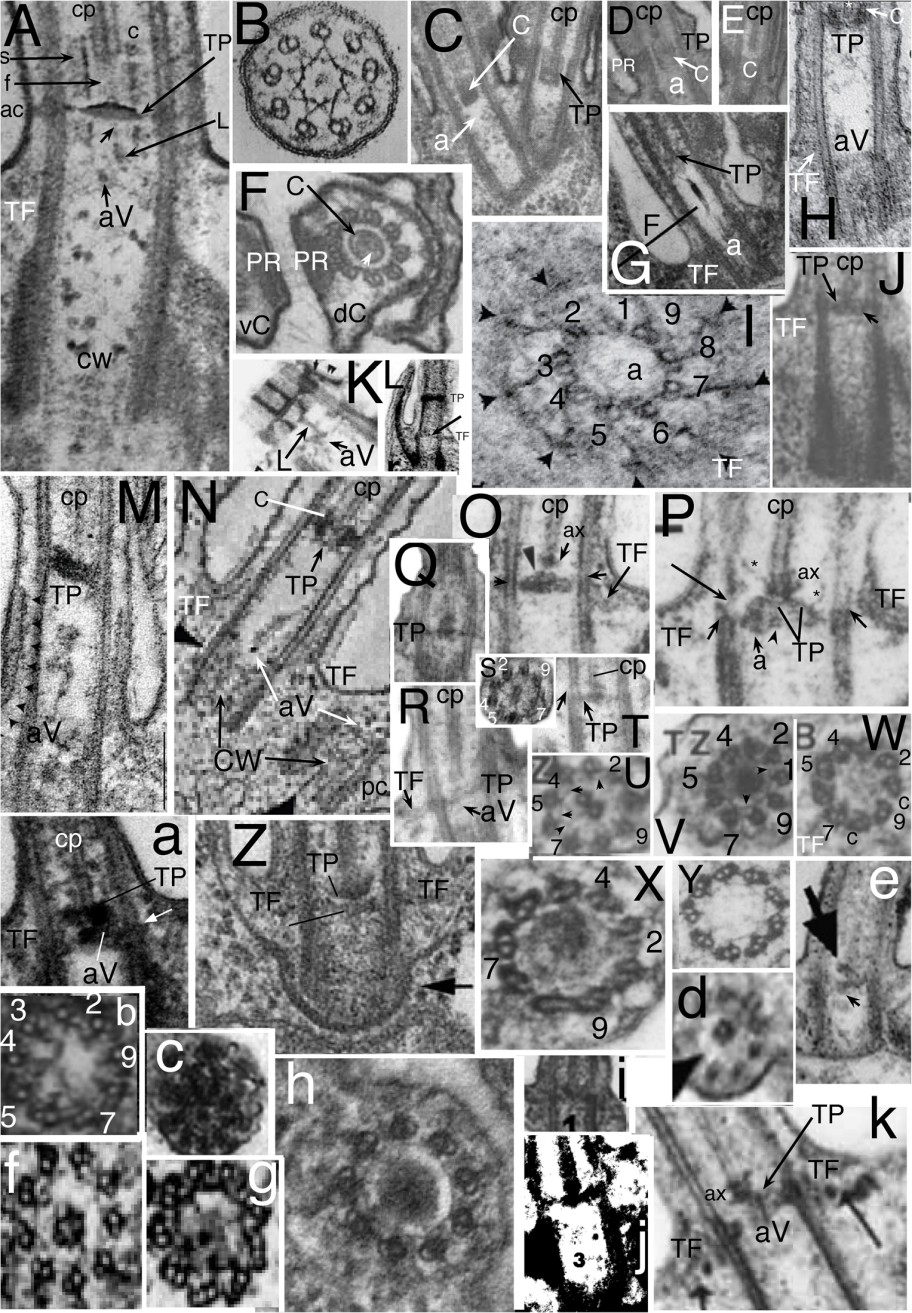
Eozoan TZs compared with green algae**. A, B.*** Stigeoclonium* sp. (Plantae: Chlorophyta, Chaetophorales). In **A** The acorn-V filament system (**aV**) is joined by slanting linker (**L**) directly to a central axial filament (**arrow**) stemming from the less dense lower part of **TP**; this proximal stellate structure and its central filament (f) are shown in TS in **B);** distal and proximal 'basal cylinders' (**c**) have a fluted wall, seen in TS as 18-gonal stars with 18 dense granules at each vertex, so are not literally cylinders**.** They are really two concentric 9-pointed stars, mutually rotated by 20°, the inner more obtuse star points being attached to the inner vertices of the outer, whose vertices join to the A-tubule feet. The central pair enters the upper cylinder and is joined to TP by a central fibre (**f**) resembling a distal hub-spoke structure, less obvious than cartwheel (**cw**) hub-spokes. **C-F.*** Rhynchomonas nasuta* (Eozoa: Euglenozoa: Kinetoplastea: Bodonida: Neobodonina) from Swale (1973 figs 2D, 3B, D, 4A). **C-E.** In LS a sleeve-like basal cylinder (**c**) surrounds **cp** and **TP** level with the base of the paraxonemal rod (**PR**); **a** putative acorn complex. **F.** The basal cylinder (**c**) surrounds both cp mts (arrow);**vC** ventral cilium; **dC** dorsal cilium. **G.*** Azumiobodo hoyamushi* (Neobodonina) from Hirose et al. (2012 fig. 2A) by permission; the proximal TZ, longer than in *Rhynchomonas*, has a central filament, **F**. **H. I.*** Trypanosoma brucei brucei* (Kinetoplastea: Trypanosomida)*.***H.** LS showing very short basal cylinder (**c**) surrounding cp (asterisk) distal to **TP** and putative acorn-V (**aV**) at **TF** level**. I.** TS of acorn (**a**) and TFs (arrowheads); numberd after Geimer and Melkonian (2004). **J.*** Jakoba libera* (**Eozoa: Jakobea: Jakobina**) anterior cilium from Patterson (1990 fig. 1E) by permission; short type I TZ with TP possibly directly overlying acorn complex (arrow); these structures are less fuzzy in *Reclinomonas* (**O,P**). **K.*** Chlamydomonas reinhardtii* detergent-extracted TZ in LS showing slanting linker between acorn and proximal stellate structure. From Geimer and Melkonian (2004) by permission. **L.*** Procryptobia sorokini* (Bodonida, Parabodonina) from Frolov et al. (2001 fig. 17) by permission; type II TZ with putative acorn complex (arrow) at TF level. **M, N.*** Trypanosoma brucei***M.** Sum of five tomographic slices (total thickness 8.5 nm) showing cp termination of cp at TP and surrounding basal cylinder. **N.** 1.6 nm thick tomographic slice through junctional complex between **cp**, basal cylinder (**c**), and **TP**, and acorn complexes (**aV**) of ciliated and barren (posterior) centriole (**pc**); **cw** cartwheels. **O, P.*** Reclinomonas americana* (**Eozoa: Jakobea; Jakobina**) consecutive LSs of posterior cilium through **cp/TP** junctional complex. **O.** section though axosome (**ax**) around putative shorter mt apparently ending just above TP (arrowhead; arrows mark position where TP joins doublets), through similar plane to **J**, but sharper). **P.** section through slightly longer cp mt joined to TP; axosomal plate with slender laminae (asterisks) to doublets; the asymmetric acorn complex (**a** and arrowhead) appears to be directly attached beneath TP; arrows mark putative end of C tubules; left doublet ? broken at long arrow. **Q-T.*** Andalucia godoyi* (**Jakobea: Andalucina**) from Lara et al. (2006 figs 6, 7, 12, 16). **Q, R** anterior cilium in oblique and LS. **S.** posterior cilium TS embracing superimposed cp base and TP lattice**. T. U-W.*** Stygiella incarcerata* (**Jakobea: Andalucina**) consecutive sections of anterior cilium from Simpson and Patterson (2001, then *Jakoba incarcerata*) fig 3h-j by permission. **U.** immediately above cp base doublets, five filaments (partial-nonagonal) link A-tubule feet of doublets 2-7 only. **V.** immediately proximal to **U** a dense semicircular sheet stretches between doublet 2-7 and four filaments (partial nonagonal) link A-tubule feet of doublets 7-9, 1, 2 **W.** Top of centriole with filled C tubules (**c**) and acorn filaments. **X, Y** consecutive sections of *Reclinomonas americana* anterior cilium TP **(X)** and top of centriole (**Y**) with nonagonal fibre linking A-tubule feet**.*** Reclinomonas americana* TS of centriole **Z.*** Stephanopogon pattersoni* (**Eozoa: Percolozoa**) arrow centriolar cup. **a.*** Creneis carolina* (**Eozoa: Percolozoa**) from Pánek et al. (2014 fig. 5E) by permission; TP overlies asymmetric dense acorn complex (aV); arrow marks end of C tubule. **b.*** Pleurostomum flabellatum* (**Eozoa: Percolozoa**) possible acorn-V structure from Park et al. (2007 fig. 3C) by permission; numbering follows Geimer and Melkonian (2004)**. c.*** Pharyngomonas kirbyi* (**Eozoa: Percolozoa**) TP in TS TP in TS from Park and Simpson (2011 fig. 4H) by permission. **d. e.*** Stephanopogon minuta* TZ from Yubuki and Leander (2008 5A, 5B) by permission. **d.** TS showing dense hub and asymmetric acorn (at level of aY in **Z**). **e.** LS showing axosomal thickening of TP (large arrow) and acorn complex (small arrow). **f.** cilium 1, **g.** cilium 2 of **'*** Percolomonas*' *sulcatus* showing putative acorn-V complex (**Eozoa: Percolozoa**) from Brugerolle and Simpson (2004 fig. 4c). **h.*** Stephanopogon pattersoni* TS showing TP with radial links to doublets and A-B links. **i.*** Pleurostomum flabellatum* TZ in LS from Park et al. (2007 fig. 3A) by permission**. j.** *Percolomonas cosmopolitus* (**Eozoa: Percolozoa**) from Fenchel and Patterson (1986 fig 6c) by permission. **k.*** Tetramitus rostratus* (**Eozoa: Percolozoa**) cilium 3 Balamuth et al. (1983 fig. 12) by permission. A, from Manton (1964 figs 1, 20); H, I, M, N from Lacomble et al. (2009 figs 1C, 3E, 4A, D); O, P, X, Y from O'Kelly (1997 figs 8, 9, 12, 13); Z, h from Lee et al. (2014 figs 4D,F) by permission.

Probably all eukaryotes have some kind of A-B link, typically close to TP. Ancestrally and in dorsates they appear to be single direct links as in *Gefionella*, but in corticates there are often two between each doublet pair that seem V-shaped (e.g., *Bigelowiella* Fig. 4A, C). They are typically single in Natozoa but inward pointing V-shape of the corticate inner linker appears to go back to metamonads (e.g., Fig. 10) and Euglenozoa (Fig. 16F, I). In *Chlamydomonas* and related green algae and haptists with stellate structures they are more complex and include a prominent radial component connecting to TP (Fig. 10 M, O) which may strengthen support for the more complex structures associated with TP. Thus the V-shape of the inner A-B link may be an ancestral natate character and additional presence at least near the TP of an outer A-B link an ancestral corticate character.

## Sequence phylogeny supports this scenario

This tripartite division of eukaryotes into three major clades with contrasting ciliary structure and function is surprisingly well supported by a phylogenetic analysis of two sets of proteins of eubacterial origin that eukaryotes probably acquired from the enslaved α-proteobacterium that became mitochondria (Derelle et al. 2015). Most cite only the authors' analyses with 37 eubacterial or 39 α-proteobacterial proteins (with 15 in common, thus 41 in all) as outgroups to root the eukaryote tree which put the root between photaria (as defined on Fig. 11) and malawimonads plus poiates on five of the six trees (using three algorithms) and between Eozoa/Discoba and all other eukaryotes on the sixth. However, these eukaryote proteins are collectively highly divergent from their eubacterial ancestors as a result of extra-rapid evolution during mitochondrial origin, raising the danger of long-branch attraction artefacts. Derelle et al. therefore also analysed 27 eubacterial proteins after excluding the 10 most divergent ones which could have biassed the results. Remarkably both PhyloBayes site-heterogeneous trees put *Malawimonas* represented by two species and Diphylleida (represented only by *Collodictyon*) as sister of all other eukaryotes, albeit with only low to moderate support for their being excluded from the rest (0.56 using CATGTR+G4; 0.85 with CAT+G4, the former (best fitting) showed *Malawimonas*, *Collodictyon* and all other eukaryotes as a collapsed trifurcation, the latter (less will fitting the data) put *Collodictyon* deepest but this could be an artefact of it not having any close relatives on the tree and thus divergent from others that it was pulled to the bottom when using a less good algorithm). However ML (known to be less accurate and more prone to long-branch artefacts) placed these two taxa one node higher as sister to obazoa. Theoretically CATGTR+G4 site-heterogeneous trees excluding the ten fastest evolving proteins should be the most accurate. Had they not regrettably allowed the software to collapse the deepest branches into an uninformative trifurcation, the deepest branch would probably have been *Malawimonas*, then *Collodictyon*, then all other eukaryotes, judged from the order shown. Given that malawimonads have shorter branches than all bikonts and most obazoa they are less likely than most lineages to be pulled to the base of the tree artefactually by the eubacterial outgroups.

It was disingenuous therefore not to mention this important result anywhere, and to consign the trees showing this novel finding solely to the electronic appendix without discussing its evolutionary significance. Derelle et al. also unwisely wrote that 'both eukaryotic datasets based on proteins of bacterial origin bear a congruent phylogenetic signal' and give the same position of the root in most analyses' and that eukaryote relationships 'are fully consistent with the results of [six] recent analyses' and saying they 'pinpoint a single eukaryotic root'. These statements hide the fact that they actually used *three* datasets not two and the theoretically best *totally contradicted* the position of the root advocated in their abstract and earlier paper (Derelle and Lang 2012) using only mitochondrial proteins; and also that all recent PhyloBayes trees using 124-351 proteins of neomuran origin robustly contradict the deep topology within their supposed 'clade' Opimoda by putting malawimonads and diphylleids more deeply than their 37/39 protein trees (Brown et al. 2013, 2018; Cavalier-Smith et al. 2014, 2015; Zhao et al. 2012). Derelle et al. thought that their α-proteobacterial dataset should be more reliable outgroups for eukaryote phylogeny than eubacterial proteins generally as they are somewhat less distant than some other eubacterial proteins. In fact comparison with those five papers and three others (Gawryluk et al. 2019; Janouškovec et al. 2017; Lax et al. 2018) that also put *Malawimonas* as the deepest branch, but like several of the nine main trees of Derelle et al. wrongly grouped *Collodictyon* with Amoebozoa show better topological agreement with the eubacterial set trees. Thus using only mitochondrial genes is objectively worse, I suggest because using proteins involved in a wider set of processes is likely to be less biassed by idiosyncratic evolution related just to mitochondria, and because (as I have often found) a wider set of outgroups often gives superior results, probably because using one taxon only (worse still one species as some do) that may by chance misleadingly attract the wrong ingroup to the base if fortuitously shared oddities systematically bias root position.

It is not clear why they ignored their ground-breaking 27-protein trees, other than that their topology fitted neither their earlier result nor unsound expectation that mitochondrial proteins should be more reliable. Dismissing results from excluding the fastest evolving genes as 'inefficient' because patristic distances were reduced by only 8-10% was irrational and misleading if, as in this case, such reduction refutes the main conclusion of the paper. However, their exclusion of outparalogues from the eubacterial data that probably biassed the trees of He et al. (2014) into incorrectly putting Discoba as the deepest branch was a valuable advance. Giving the 37/39 proteins more credence because they most agreed across methods was not sound because agreement between methods and datasets can result from a dominating consistent bias rather than true phylogenetic signal. In hard-to-resolve cases it is irrational to expect poor and good methods to agree. Better methods should give a different result from poor ones. I would argue that the long-branch bias was so great in the 37/39-protein data that all three methods were inadequate to the task, but after reducing the distance somewhat, artefacts were reduced in intensity enough for the best method to give the most accurate result and the worst the least accurate, mimicking the He et al. (2014) incorrect result.

I consider the widely ignored 27-protein trees of Derelle et al. (2015) technically the best evidence yet from sequence tree outgroup rooting of the root position of the eukaryote tree. It is all the more credible because it gives the same result as my demonstration of a uniquely primitive half-plate/acorn and TF structure for Malawimonada alone. This is the first time we have a congruent rooting based on ultrastructure and sequence tree outgroup-rooting. Because of taxonomically and genetically better sampled site-heterogeneous trees, overall topology of the eukaryote tree is also more secure than it was seven years ago; consensus has steadily grown since recognition of clade obazoa (Brown et al. 2013). If we accept the rooting of Fig. 11, group Diphoda is not a clade, thus its definition is illogical; it is now pointless (united by nothing). Discaria however is a clade, a supergroup established by the origin of a rotationally symmetric TP between acorn complex and asymmetric cp axosome and by origin of the V-filament system. These are the biggest changes in evolution of the ciliary system since cilia and centrioles evolved. Discaria immediately split into two major lineages with different modes of ciliary motion, which in Fig. 11 I call 'dorsates' and 'natates'. Though I did not find a satisfactory TS for the planomonad TP or acorn the evidence from Fig. 13 is most consistent with their being axially separate but close structures, similarly to metamonads rather than TP being absent as in malawimonads.

For years outgroup rooting using rDNA trees was plagued by long-branch attraction artefacts that put the fast evolving metamonads and microsporidia at the base of the tree, discrediting this way of rooting eukaryotes. This defect also applies to ribosomal multiprotein trees, becoming standard for prokaryote phylogeny. Cavalier-Smith and Chao (2020) found that rooted 51 or 26 ribosomal protein (RP) trees place the eukaryote root between Discoba and other eukaryotes or between Percolozoa and all other eukaryotes using PhyloBayes or ML depending on taxon sampling. We showed that both conclusions are untrustworthy as the eukaryote stem on RP trees is so long that its accelerated evolution will have overridden the majority of the phylogenetic information that might root them correctly.

The proteins used by Derelle et al. (2015) though highly diverged exhibit a substantially shorter stem, so should have more chance of correctly placing the root *provided the most divergent proteins are excluded*. It would be worth redoing their 27-protein study after adding more similarly conserved proteins and key missing taxa, notably Varisulca other than *Collodictyon*, Planomonada, and other key deeply branching missing groups, notably Hemimastigophora, Glaucophyta, and Haptista, to see if Malawimonada remain at the very base of the tree. Unfortunately, the entirely anaerobic Metamonada cannot be included because of loss of mitochondria-related proteins. However, given a root beside Malawimonada or *Malawimonas* plus *Collodictyon*, Metamonada would be sister to photaria according to the 351-protein trees of Brown et al. (2018) where Metamonada plus eozoa and corticates form a maximally supported clade by PhyloBayes and 98% supported by ML, and thus are not sisters to obazoa plus Planomonadida and/or Varisulca.

## Further phylogenetic perspectives

Phyletically dorsates correspond exactly with the podiate clade (i.e., Sulcozoa and all their descendants) as first defined (Cavalier-Smith 2013). At that time I supposed that three characters ancestrally defined dorso-ventrally flattened Sulcozoa: a dorsal sub-plasma-membrane proteinaceous 'thecal' (really cytoskeletal) layer; ventral thin, branched pseudopodia for feeding; and gliding locomotion on the posterior cilium. Multiprotein phylogeny has now reasonably strongly and consistently shown that Planomonada, which have ciliary gliding and dorsal 'theca', but no pseudopodia diverged first. Therefore podiate pseudopodia evolved one node later than dorsal theca and gliding. Therefore I now exclude the probably primitively non-peudopodial planomonads from podiates and coin the name dorsates for the major clade comprising Sulcozoa, Amoebozoa and opisthokonts; ancestrally dorsates had the dorsal proteinaceous layer (absent in their ventral groove) and posterior ciliary gliding. Podiates probably all have myosin II, the paralogue used for their amoeboid motilty (Richards and Cavalier-Smith 2005; Clark et al. 2007) whereas it has not been found, despite repeated attempts, in Planomonadida, Metamonada or Corticata (Berney and Cavalier-Smith unpublished), though a divergent version is present in percolozoan *Naegleria* amoebae. In marked contrast to dorsates, gliding evolved not ancestrally in natates but only secondarily in highly derived lineages, notably at the base of phylum Cercozoa in Rhizaria and of Euglenoida in Euglenozoa, and also in a few phagotrophic heterokonts such as *Caecitellu*s.

'Natates' emphasises that this was ancestrally a clade of swimming bikonts whose centrioles were primarily held together divergently by a single large central cross-striated connector composed largely of centrin. This is retained in bikont corticates and eozoa, but Metamonada and most Percolozoa with two extra cilia also have extra striated connectors, whilst Hemimastogophora lost it when evolving rows of unikont kinetids. Malawimonads have different, thinner connectors *on either side* of their well separated, askew centrioles: most prominent a very long amorphous right distal 'dense fibrous' connector and a cross-striated left connector that is connects the anterior centriole distally to proximal part of the posterior centriole (*Gefionella*) of to the proximal part of the right root (*Malawimonas*). Identifying doublets linked to the malawimonad half-acorn as homologues of those in corticates bearing the peripheral acorn filament enables a consistent numbering system for all eukaryotes where acorns are clearly established. Thus *Malawimonas* dense fibrous connector stems from anterior centriole triplets 9, 1, 2 and its striated band from doublets 4, 5 (Fig. 15D, E, G). In *Chlamydomonas* the distal striated connexion links triplets, 9, 1, 2, thus is homologous with the distal dense fibrous connector of malawimonads not the more obviously striated band (which links the proximal end of the anterior centriole to the proximal end of the posterior right root) presumably lost in Chlorophyta. By contrast, as I show below, fibrous centriolar connectors of fungi that vary in degree of striation link doublets 4-6 in the chytridiomycete *Polyphlyctis willoughbyi*. They are thus not homologous with the *Chlamydomonas* distal striated conexion (or with its proximal ones that link triplets 8/9 to 2/3), but probably are homologues of malawimonad striated bands.

Dissimilar connectors on opposite sides of the centriole appear to have been retained by most dorsates (even in Diphylleida when becoming swimmers), but the striated connector was apparently lost by planomonads during their evolution of flattened dorsoventrality, whereas Apusozoa ancestrally added a third median connector (but lost again by *Apusomonas* itself). By contrast, the more recent, derived opisthokont dorsates (new infrakingdom Opizoa (i.e., phyla Choanozoa and Opisthosporidia), probably lost the distal connexion when losing the posterior cilium and its roots, reducing it to a relict shortened barren centriole that lacks the distal portion (see later).

Figure 11 shows the primary divergence within natates is between the small (but structurally diverse) anaerobic clade Metamonada ancestrally with tetrakont kinetids, and huge aerobic clade that approximates to what we once called bikonts (Stechman and Cavalier-Smith 2002); but is here named 'photaria' to emphasise that it embraces all eukaryote algae, whether they arose by primary, secondary or tertiary symbiogenesis (Cavalier-Smith 2013), plus their closest non-photosynthetic relatives and descendants. Photaria does *not* refer to a synapomorphy, because the clade was ancestrally non-photosynthetic, and I have not identified any single ultrastructural synapomorphy for it. Its bikonty contrasts with the tetrakonty of its metamonad sisters, which added two extra anterolateral cilia, but is the ancestral character for all crown eukaryotes, and was lost by some sublineages: notably, most Percolozoa duplicated their kinetids to make a double bikont structure, whereas Hemimastigophora became truly unikont by losing ciliary transformation and centriolar connectors; Ciliophora evolved kineties—rows of ancestrally bikont ciliary kinetids.

**Fig. 17.**
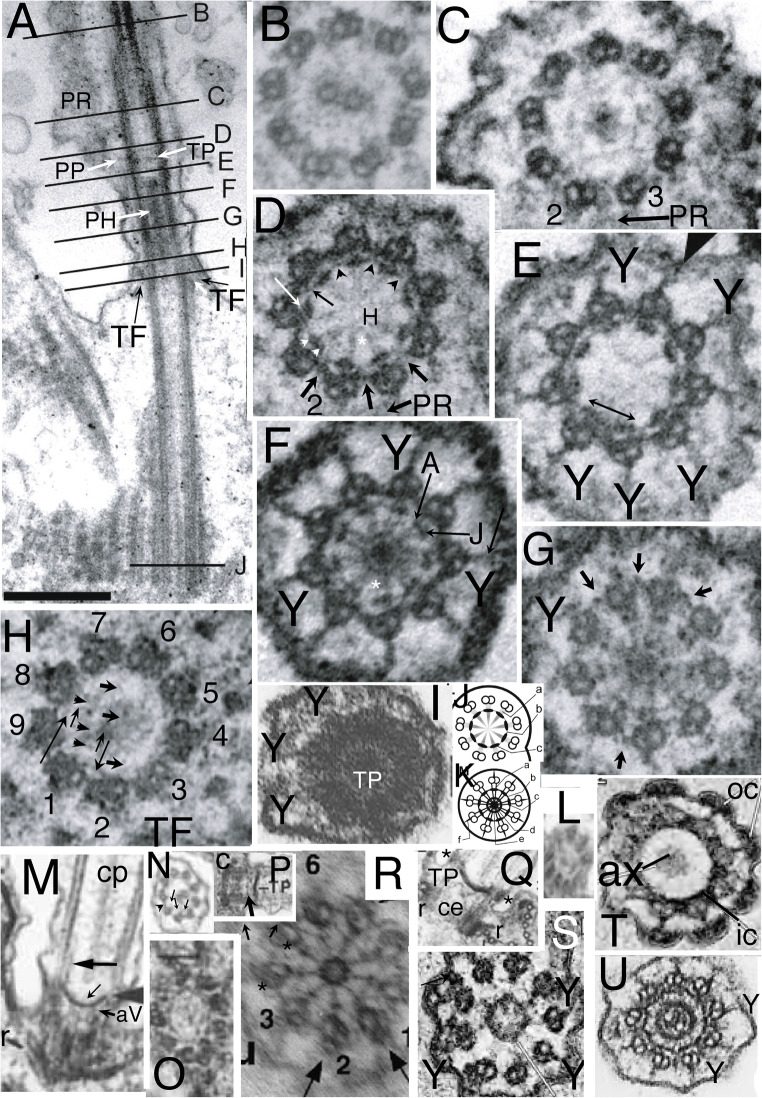
Six filamentary patterns in immensely stretched TZ of the postgaardiid *Calkinsia*(Euglenozoa)A-H; short TZ/centrioles of Hemimastigophora (L-P), and other comparisons**. A.*** Calkinsia aureus* dorsal cilium LS showing levels represented in TS by **B-H**; PR paraxonemal rod; paraxial plate **(PP)** is opposite **TP; PH** proximal hub; TF transition fibres; white arrow marks end of C tubules. **B.** 9+2 axoneme; doublets with arms and spokes. **C.** doublets unchanged**,** cp replaced by axosomal filament**. D.** distal hub-spoke structure; the diamond-shaped densities beside the doublets (long white arrow) have two distinct parts (white arrowheads) lesser densities (asterisks) between them and the hub (H) and the spoke filaments are also double; the diamonds are linked by V-shaped filaments like star points directed towards the centres of the A-B links (black arrowheads). **E-G.** successive levels of proximal hub-spoke structure, spokes more clearly double, showing Y-links, (Y, arrowhead). **H.** acorn V at the TF/triplet start level; two longest thin arrows mark peripheral acorn and fat arrows lumenal acorn filament; other arrows explained in text **I.*** Postgaardi mariagerensis***(Euglenozoa**, Postgaardea) from Simpson et al. (1996 fig. 17) by permission; Y Y-links. **J. K.*** Calkinsia* diagrams from Yubuki et al. (2009) interpreting hub-spoke structures: **J** level D, **M** level F. **L-P Hemimastigophora TZs** from Foissner and Foissner (1993 figs 44, 45, 49, 51) by permission: **L-P.*** Stereonema geiseri.***L.** Slightly oblique TS of putative acorn complex. **M.** Median LS of TP (arrowhead) with faint central projecting filament (small arrow) and thin basal cylinder (larger arrow) **r** long 2-mt root*.***N.** TS of distal TZ with slender nonagonal fibre (arrows) attached to A-tubule feet and thicker A-B links (arrowhead. **O.** TS of centriole/TZ junction embracing central part of T with irregular lattice superimposed on underlying acorn filaments. **P.** Tangential LS of **TP** and underlying acorn filaments (arrow); very short centriole with basal cup (**c**). **Q.*** Hemimastix amphikineta* LS of **TP,** separate ac (asterisks), and long and short 2-mt roots (**r**) from Foissner and Foissner (1993 fig. 58) by permission. **R.*** Sainouron acronematica* (**Cercozoa**) distal hub-spoke complex, from Cavalier-Smith et al. (2008b fig. 4e) by permission. **S, T.*** Viridiraptor invadens* (Cercozoa): **S.** TZ at level of proximal hub (='central ring') and its spokes; **Y** Y-links. **T.** TZ at level of axosome (**ax**) and inner cylinder (**ic**); the 'outer cylinder' (**ic**) is just the extra dense arms of the Y-links. **U.*** Collodictyon triciliatum*(Sulcozoa) TS of cp within sleeve and surrounding stellate structure and Y-links(Y) from Brugerolle et al. (2002) by permission. A-H, J, K from Yubuki et al. (2009 fig. 6); S, T from Hess and Melkonian (2014 fig. 5C_2_, E) by permission.

As noted above, *Hemimastix* TZ probably has a central distal filament (Fig. 17M) or hub connecting TP to the base of cp; a shorter less conspicuous one is also present in *Spironema* and *Heteronema* (Foissner and Foissner 1993). As I have not identified such a hub in metamonads, Eozoa or dorsates, yet found one widely across corticates, a distal TP hub connecting cp to TP may be a synapomorphy for the clade comprising Corticata and Hemimastigophora (Lax et al. 2018), which is confidently a clade given the strong, now compelling, evidence above rooting eukaryotes between Malawimonada and discaria. *Hemimastix* and *Heteronema* also have a thin basal cylinder above the TP (Fig. 17M, N) which TSs suggest is probably a nonagonal tube (NT). I suggest this may be a homologue of both the NTs of Plantae and Cercozoa, and also the thin basal cylinder of Myzozoa (Fig. 12J, K, R, S, T, V) as well as the thinner outer component of the TH of bigyran and pseudofungal heterokonts (Fig 10S, T, U). I name the Hemimastigophora/Corticata clade eucorta (meaning well developed (eu-) cortex = 'bark') as corticates ancestrally had a pellicle with cortical alveoli and Hemimastigophora a cortical pellicle with microtubules and proteinaceous thickening instead.

Many seem unaware that the pointless mouthful Diaphoretickes (Adl et al. 2012) was a junior synonym of corticates (Cavalier-Smith 2003a) long used as the clade name for Plantae plus Chromista referring to cortical alveoli, their likely key synapomorphy, later formalised to superkingdom Corticata (Cavalier-Smith et al. 2015). Furthermore the name Archaeplastida (Adl et al. 2005) is a pointless junior synonym for kingdom Plantae (which goes back to Haeckel 1866) as precisely redefined by Cavalier-Smith (1981). It is better to retain old simple names than to destabilise nomenclature and cause confusion by inventing unnecessary new ones or using acronyms like SAR instead of formally established subkingdom Harosa (Cavalier-Smith 2010). The reader will notice that as far as possible I have chosen short informative names for new clades that either refer to the groups' founding synapomorphy or majority phenotype (Fig. 11). One may refer to an individual organism belonging to natates as a natate and a member of dorsates as a dorsate, and of photaria as a photarian. None of these is proposed as a taxon, so all use lower case initial letters like opisthokont or a grade name like excavate; the distinctions between clades, grades, and taxa, all useful in different contexts, were explained by Cavalier-Smith (1998).

## Origin of green plant TZ stellate structures

Previously it was a mystery how the seemingly unique and complex viridiplant stellate structures evolved. This is more comprehensible now I have shown that TPs of corticates in general have a peripheral pattern with broad star points in phase with A tubules as well as narrow star points out of phase with doublets. This means that the stellate pattern did not have to evolve totally de novo in the ancestral viridiplant. It merely had to multiply the broad star subcomponent of this TP pattern axially and proximally to create both the stellate star-point pattern and filament base of these star points to make the core filament pattern of the proximal basal cylinder and its peripheral stars. Repeating that multiplication distally would make the distal basal cylinder and star, but extra material was added to thicken the distal cylinder wall, and in many but not all lineages also added on the distal face of the TP to make a thicker cross-pice to the H-profile. The two basal cylinder/star complexes then differentiated in detail. During interpolation of the two now prominent star-complexes into the TZ between cp and acorn-V structures, their assembly could have been templated directly by homologous *parts* of the preexisting TP pattern.

The slightly unusual TZ structures of chaetophoralean chlorophyte alga *Stigeoclonium* (Fig. 16A) suggests how that key step in Viridiplantae origin may have happened. Its cp base is within the distal third of the upper cylinder and apparently attached to the centre of the TP by an intervening narrow distal hub-like fibre (f) similar to that of biliphytes but about twice as long. Thus the upper cylinder/stellate pattern could have evolved *without destroying the original connection of cp*, which could have been retained by lengthening whilst extra tiers of the pattern were inserted if it was originally the glaucophyte length; but it was more likely the same length as in *Picomonas*, which is essentially the same as in *Stigeoclonium*, if as argued above Viridiplantae are sisters of Rhodaria not Glaucophyta. Thus I propose that during the transitional stage both the old narrow diameter hub and the new greater diameter basal cylinder coexisted; the latter did not evolve from the former. On my interpretation the distal hub of *Rhodelphis* is also relatively long. To explain why tomography shows a more complex star pattern lattice at the distal end of the distal cylinder as well as of proximal basal cylinder in *Chlamydomonas*, I suggest the early TP pattern was duplicated early on in the subclade including Chlamydomonadales when the ancestral distal hub was lost and cp lost its ancestral connection to TP. That may explain why the cp of *Chlamydomonas* is relatively free to rotate unlike in most eukaryotes other than *Paramecium* (Omoto and Kung 1980), whose rotation may be facilitated by only one mt being attached to TP and appropriate mode of attachment. The domed upper half of *Stigeoclonium* TP to which the distal cylinder is attached is denser than the lower half, exactly as in *Chlamydomonas*, but this extra dense layer is not attached to the dense wall of the distal basal cylinder. I suggest that attachment as in *Chlamydomonas* evolved when the distal hub was lost and functioned to stabilise the upper stellate complex mechanically.

Interpolation of the proximal star/cylinder could also in principle have occurred without destroying the preexisting connection of TP to the asymmetric acorn-V complex—exceptionally well shown in *Stigeoclonium* (Fig. 16A). As this linker is necessarily asymmetric it may have a more limited capacity to grow axially than did the distal hub. The especial shortness of the proximal cylinder of *Stigeoclonium* may be linked to the fact that the asymmetric laterally tilted linker remains attached to TP via a short central filament (Fig. 16A). In *Chlamydomonas* by contrast this tilted linker is joined not to TP but to a septum at the base of the proximal cylinder that is absent from *Stigeoclonium* (as is the distal septum of the distal cylinder). Therefore the greater separation of TP and acorn in *Chlamydomonas* than in glaucophytes like *Cyanoptyche* (Fig. 6M) was associated with disconnecting the connector to TP and evolving the proximal septum: I suggest it arose by duplication and modification of the TP lattice; its lattice pattern in the lowest tomogram of the *Chlamydomonas* proximal cylinder is not altogether clear (O'Toole et al. 2003 Fig. 3A), but is somewhat similar to but distinct from the TP lattice and that of the cylinder mid region. *Stigeoclonium* also has a finer axial linker between TP's centre and the upper end of the tilted linker, which is not normally visible in *Chlamydomonas*. However, Geimer and Melkonian (2005) discovered centrin in the acorn complex and found that a centrin-containing filament is seemingly continous though the proximal spetum thus extends from the acorn to TP (Fig. 3G). Therefore a direct filamentary connection bewteen TP and acorn may exist in all Viridiplantae despite presence of the basal transition cylinder.

Two things confirm the mechanistic plausibility of my interpretation of stellate complex evolution involving axial duplication of several existing TZ components. One is that uni-3-1 mutants of *Chlamydomonas* cause axial duplications of the basal cylinder star complex, inserting extra copies above or below the standard position, which may be subtly different from standard (e.g., losing TP whilst retaining the stellate pattern; one mutant cell that made a cilium had the TP *within* the distal cylinder, reverting to the *Stigeoclonium* condition: O'Toole et al. 2003). Both this mutation and its suppressor modify the triplet/doublet transition structure with multiple repercussions. The second evidence of an inherent tendency for TZ element duplication is the radically modified TZ structure in pseudocilia of *Glaucocystis* and *Gloeochaete* (Fig. 6G) which shows that TP duplication actually occurs naturally during evolution.

One complication to consider is that in addition to their stellate structure several *Pyramimonas* species have a basal cylinder or coiled fibre resembling a TH; this surrounds their cp distally to the stellate structure in the same position relative to the axosome as biliphyte NT (Moestrup and Thomsen 1974; Melkonian 1982). As Pyramimonadales are one of the deepest branches of viridiplant infrakingdom Chlorophyta, the *Pyramimonas* coiled fibre likely evolved directly from a biliphyte NT; if so, in early Viridiplantae the stellate structure likely coexisted with NT and was interpolated between it and the axosome in the ancestor of *Pyramimonas*. Coexistence of NT and stellate pattern shows that TH did not simply transform into the stellate basal cylinder even though both occur in the distal TZ. Fig. 10C shows that the so called spiral fibre or 'coiled fibre' of *Pyraminonas orientalis* (Moestrup and Thomsen 1974) cannot be a simple spiral as it has flat faces like an open ended polygonal prism, and is thus quite similar to NT but differs in having about 18 faces not just nine, thus about two linkers to each doublet, so may be 18-gonal. However *Pyramimonas obovata* appears to have about four links to each doublet, making it logically 36-gonal, appearing more like a circular ring (Melkonian 1982 Fig. 15). I suggest that both variants of this *Pyramimonas* distal TZ structure could have originated from the biliphyte NT by evolving extra linkers to the doublets, modifying its shape slightly. Its length also varies more than NT, having about 9 tiers of subunits in *P. obovata* but about 25 in *P. amylifera* (Melkonian 1982 Figs. 17, 16).

Therefore the distal basal cylinder of Viridiplantae probably arose not from NT but by longitudinal hypertrophy of parts of the TP of Biliphyta as proposed above. Note that in LS the *Cyanophora* thick TP exhibits two dense distal projections on each side of the central axis in Fig. 5D which are at the correct radial position to become the thick wall of a star-bearing distal basal cylinder if they were to become extended distally by multiplication. Thus the biliphyte TP was probably preadapted for evolving two star-patterns, which are not homologous to either the distal hub or the NT of ancestral Biliphyta. One extra duplication occurred only in chlorophyte algae like *Chlamydomonas* the annular connection is double, which must have occurred after the primary duplications that made the basal cylinders as many divergent green plants have only the ancestral single ac.

To test and extend my explanation of TZ stellate pattern origins we need the markedly higher resolution of cryo-electron-tomography as recently used for the centriole (Greenan et al. 2018; Li et al. 2019) to be applied to *every* axial level of the *Chlamydomonas* TZ, and also to several of the marked structural variants of viridiplant TZs (only some mentioned above), and to key biliphytes. That would establish which components are really homologous at submolecular resolution and direct attention to which need further investigation by comparative proteomics to enable a complete evolutionary picture.

## Making cryptic structures manifest: the Postgaardia test case

The underlying principle behind my explanation of stellate structure origins is that ordinary looking simple short TZs have much axially distinct structure that is normally hidden by a combination of superimposition of two or more structures within a single thin section and obscuring dense anorphous matrix. Evolutionary stretching of different axial subzones can make formerly hidden structures apparent without radical molecular innovation of their underlying lattice structure. Given that a single filament like F-actin is only 7 nm thick, one 50 nm 'thin section' could in principle contain as many as seven different superimposed filamentary arrays. To test my thesis we need to find a corticate outgroup in which some species have short type I TZ and at least one has an exceptionally long type II in which every part is axially so stretched that all components are separated so greatly that two cannot be included in a single section. Also as many as possible must ideally be reduplicated many times individually to fill a whole section, so superimposing the *same* hypertrophied substructure making it easy to identify and unambiguous to compare with distant taxa. The best example is euglenozoan infraphylum Postgaardia that is probably sister to infraphylum Euglenoida (itself with secondarily long TZ) and comprises only three genera: *Postgaardi* with typical short type I TZs (Simpson et al. 1996 fig. 17) with TP close to the ciliary base, *Bihospites* (Breglia et al. 2010) possibly with a longer TZ, but structure unknown; and *Calkinsia* with very long (1 μm) TZ (Yubuki et al. 2009, who did not identify either key TZ structure: TP and acorn-V) within which serial sections revealed six different filamentary structures, not previously compared with any non-Euglenozoa but implied to be unique to *Calkinsia*.

**Fig. 18.**
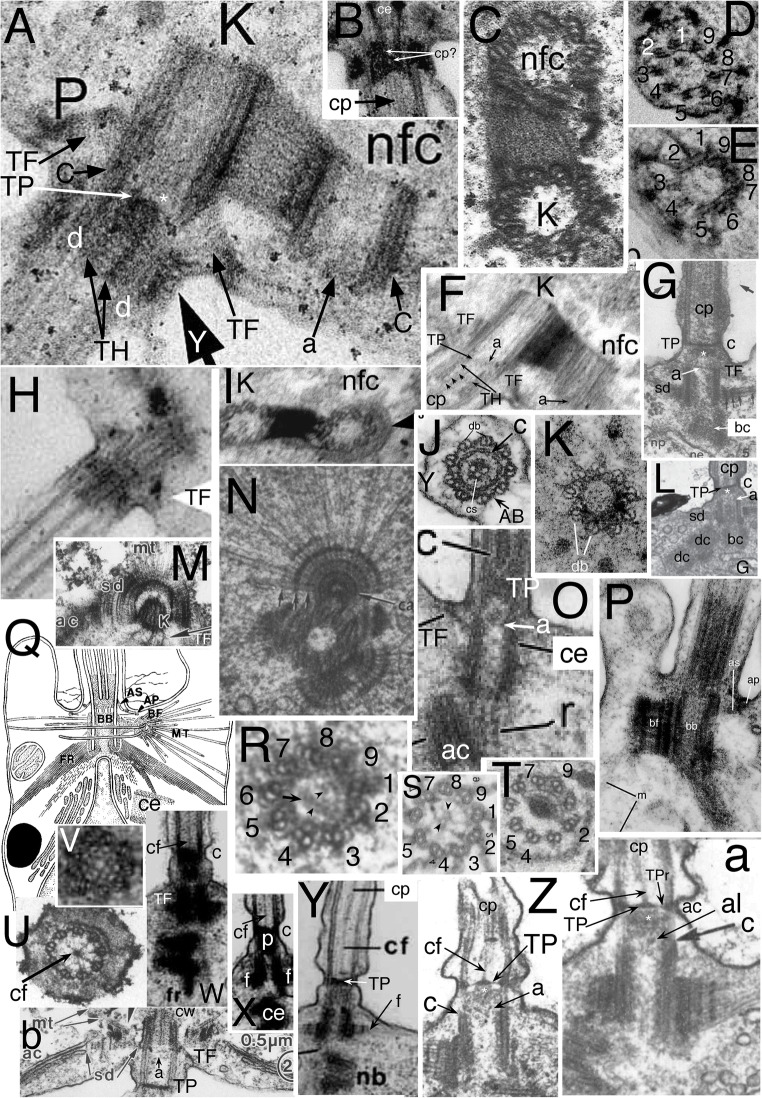
Opisthokont transition zones: comparison of Fungi (A-I, M), Choanozoa (N, R, U, X, Y) and sponges (J, K, O-Q). **A-C.*** Polychytrium aggregatum* Chytridiomycetes (Polychytriales**)** zoospores*.***A.** Tangential LS through cilium-bearing centriole (K = kinetosome) and barren centriole (nfc =non-flagellate centriole; distally capped by acorn-like structure **a**) showing endpoints of C tubules (**C**) and lattice substructure of transition cylinder/helix (**TH)**. **TF** transition fibres, **TP** transition plate, asterisk putative acorn-homologue, **Y** Y-links. **d** doublets. **B.** Median LS showing central pair mts apparently passing through the strongly stained TZ 'plug' zone **(cp?)** to top of centriole**. C.** TS through fibrous connective linking centrioles. **D, E.*** Neokarlingia chitinophila* (Polychytriales) zoospore cilium consecutive serial sections just distal to (**D**) and at (**E**) the TZ acorn-homologue (fig 4a, b). **F, I.*** Karlingiomyces asterocystis* (Polychytriales); LS (**F**) and TS through centrioles; **cp** penetrates through entire **TH** (**F**, note radial linkers from **TH** to **cp**), its base being below TH proximal end, in same section as distal end of triplets (**I;** at this level **nfc** lumen has a fuzzy acorn-homologue). **G.*** Stephanoeca diplocostata* (**Choanoflagellatea: Acanthoecida)** from Leadbeater (1987 fig. 5); **a** acorn; **bc** barren centriole; **c** constriction; **sd** striated disc mts; **asterisk** plug proximal to **TP;** arrows ciliary hairs. **H.*** Maunachytrium keaense* (Chytridiomycota: Lobulomycetales) from Simmons et al. (2009 fig. 6A) by permission. **J, K.** Demosponge *Sigmadocia caerulea* (Haplosclerida) larval epithelial cilium from Maldonado (2004 fig. 4J, G) by permission. **c** = transitional cylinder linked to A tubule feet of doublets (**db**) and by thin radial links to central sheath (**cs**) around cp; **Y** = Y-links, AB =A-B linker. **L.*** Volkanus costatus* (**Choanoflagellatea: Acanthoecida)** from Leadbeater (2010 fig. 25); **a** acorn; **bc** barren centriole; **dc** daughter centrioles; **sd** striated disc mts; **asterisk** plug proximal to TP. **M.*** Monoblepharis polymorpha* (Chytridiomycota: Monoblepharidales) semicircular striated disc (**sd**), from which microtubules (**mt**) radiate, surrounds ciliated centriole (k);**ac** annular cisterna of Golgi. **N.*** Codonosiga* (=*Codosiga*) *botrytis* (**Choanoflagellatea: Craspedida)** TS of radiating mts around anterior centriole, with circumferential dense arcs shown in LS in **Y**, **Z**. **O.** Demosponge *Halisarca dujardini* (Halisarcida) from Gonobobleva and Maldonado (2009 fig. 3C) by permission; **c** = transitional cylinder. **a** = putative acorn-homologue;**ac** accessory centriole, **ce** ciliated centriole, **r** =rootlet. **P.** Demosponge *Sigmadocia caerulea* (Haplosclerida) larval epithelial cilium from Maldonado (2004 fig. 4H); **as/ap** =TF; **bf** basal foot from which mts (m) radiate into cytoplasm. **Q.** Demosponge *Halichondria melanodocia* (Halichondrida) larval epithelial cilium from Woollacott and Pinto (1995 fig. 2) AS =alar sheet (= TF), FR = fibrous rootlet. AP = **R.** Choanoflagellate *Codonosiga* (=*Codosiga*) *botrytis* TZ/centriole junctions showing acorn homologue with peripheral filaments linking A-tubule feet of five doublets (7-9, 1, 2); arrowheads mark possible homologues of the more distinct central elements in *Chlamydomonas* in **S**; arrow marks densities absent in *Chlamydomonas* but present in *Phalansterium* (Fig. 19C,D). **S.*** Chlamydomonas reinhardtii* (Chlorophyta)acorn-V complex proximal to filaments with acorn shape; arrowheads mark similar elements at centre of standard arc-like acorn lumenal filament between doublets 2 and 7 and subsidiary filament arcing from 1 and 8) from Geimer and Melkonian (2005 fig. 4.38) by permission*.***T.** Malawimonad *Gefionella okellyi* acorn-homologue(not acorn-shaped) without V-filaments. **U.*** Codonosiga* (=*Codosiga*) *botrytis* TS through ciliary bulge showing central fibre (**cf**) and radial links to doublets fig. 30 **V.** Choanoflagellate *Monosiga ovata* (**Craspedida**) TS of barren basal body showing unique lumenal lattice**. W.*** Desmarella moniliformis* (**Choanoflagellatea: Craspedida); cf** central filament connecting cp to dense plug at and below constriction; f**r** fibrillar rootlet, **c** ciliary constriction. **X**. *Monosiga ovata* showing wide constriction with central plug (**p**) so densely stained as to obscure details visible in **Y. Y.** Choanoflagellate *Monosiga ovata* showing central filament linking cp (**c**) and a secondary plate just distal to **TP**; **nb** aciliate centriole; mt fans (**f**) surround centriolar base. **Z, a.*** Codonosiga* (=*Codosiga*) *botrytis* fig. 21 fig. 27. *Codonosiga* (=*Codosiga*) *botrytis* LS showing variable length of central fibre connecting cp axosomal plate to TP; **al** putative cross section of acorn-like lumenal filament. **b.*** Monoblepharis polymorpha* (Chytridiomycota) striated disc (**sd**) level with top of cartwheel (**cw**) (which protrudes from centriole base) associates with a smooth annular cisterna (**ac**); **a** acorn filaments, seen in TS in Fig. 20A. A-F from Longcore and Simmons (2012 fig. 3c, d, e, 4a, b, 6a, d); N, R, U, V, Z, a from Hibberd (1975 figs 15, 19, 27, 29, 30), M, b from Mollicone and Longcore (1994 figs 18, 20) by permission V, X, Y. from Karpov and Leadbeater (1998 figs 3, 5, 6) by permission

I show here that all six are structurally related to corticate TZs discussed above and that they enable the correct axial order of homologous structures in other eukaryotes to be established using their order in *Calkinsia* (Fig. 17A-H) as reference. I identified TP as the previously overlooked intradoublet plate opposite the paraxial plate below the paraxonemal rod, as this is its position in kinetoplastids (Fig. 17A); unfortunately no TS was shown for that position. The overlooked acorn-V is visible in the section where triplets begin (Fig. 17H) though slightly obscured by underling distal centriolar lumenal structures—presence of TFs shows this section must be closer to level I in Fig. 17A, not H. Doublets/triplets are labelled after Geimer and Melkonian (2005) assuming that the short fat arrows mark the position of the lumenal acorn filament and arrowheads mark the densities corresponding with the inner arc centrin filament of *Chlamydomonas* shown in Fig. 18S. An irregular lattice possibly representing distal centriolar matrix obscures the positions opposite triplets 4, 5 where *Chlamydomonas* and Cercozoa have the V, but a filament linking the putative peripheral and lumenal acorn filaments between doublet/triplets 8 and 9 (marked in Fig. 17H by upper short thin arrow) is positioned exactly as is the filament connecting the base of the V to the peripheral acorn in *Chlamydomonas* if this triplet numbering is correct. Also the central density marked by the right arrow corresponds with the central density at the base of the *Chlamydomonas* V. Thus it is likely that *Calkinsia* and Euglenozoa generally have a V-system as well as acorn filaments, as deduced above for Metamonada. Fixing those landmarks enables the following interpretation.

The most distal structure connecting *Calkinsia* cp to TP (Fig. 17A, C) is a central filament similar to those of choanoflagellates and haptophytes shown above. It differs by being enclosed by an irregularly stellate medium density area around which the doublet spoke heads (absent at the level of the central filament in other eukaryotes) cluster. This makes the central zone at C and above more rod-like than filament like in overall appearance, and in TS similar to the axosome of the cercozoan *Viridiraptor* in TS (compare Fig. 15F inset and K* inset). I therefore suggest it may have arisen by serial axial multiplication of euglenozoan axosomal structures.

Closer to TP where doublets lack arms and spokes is a faint but axially extensive distal hub-spoke system (Fig. 17D). Though less densely stained the central hub has a similar appearance and diameter to that of the cercozoan *Sainouron* (Fig. 17R). However the spokes are distinctly different, each comprising two close but distinct filaments; their outermost density close to the A-tubule feet clearly comprises two separate dense granules; a less dense set of granules about halfway along are also apparently double and distinctly further from doublets than is the major dense granule in *Sainouron*.

Proximal to TP and distal to the acorn is a similar hub-spoke structure that is accompanied by Y-links apparently absent distally (Fig. 17E-G). Just below TP hub-spokes are essentially indistinguishable from the distal hub-spokes (Fig. 7E) but more proximally the hub is filled by dense matrix (Fig. 17F, G) making it appear as a dense rod in LS (Fig. 17A). These spoke filaments are more strongly stained thus obviously double. Yubuki et al. (2009) did not notice the distal hub or that distal spokes are also double, so their diagrams (J, K) represent them as basically different. In fact a fundamentally similar hub-spoke structure extends all the way from the base of the central filament to the acorn *on both sides* of TP, differing at different levels primarily in strength of staining of different components. Might the hub-spoke structure have evolved by extensive axial duplication of parts of basic lattice structure of the postgaardian TP?

Despite no published LS or serial TS of *Postgaardi* TZ, Fig. 17I appears to be a TS of its dorsal TP, but overstaining conceals detail; but shows it is overall radially symmetric, with central denser zone with an irregular lattice connected by numerous radial links that traverse a peripheral lighter zone to link it to the doublets. There are substantially more than nine radial linkers, evenly spaced in the upper region; from their spacing I infer roughly 18 altogether, but they are clearly not arranged in pairs as in *Calkinsia* spokes, so could not be simple precursors of its spokes. The adjacent ventral cilium in the same section (Simpson et al. 1996) shows TFs and has a semicircular central structure likely representing the acorn. As the two centrioles are parallel and almost exactly side by side (Yubuki et al. 2013 Fig. 1 based on unpublished serial sections) this implies its TZ must be very short. As *Calkinsia* spokes are basically double it is unlikely that they are homologous with those of Rhizaria and other corticates where they are invariably single. However its hub might be related to that of Rhizaria. Though no hub-like structure is visible in Fig. 17I one could easily be hidden with the central dense disc, which might represent a filled hub like that proximally in *Calkinsia*, as it is essentially the same size as the hub of *Viridiraptor* proximal hub-spokes in Fig. 17N; one can scarcely even see mts in the surrounding doublets.

An attractive explanation for why hubs are present scattered across most photarian lineages, but have not been found in metamonads, malawimonads or dorsates, is that a TZ hub evolved in association with TP in the ancestral photarian. In principle hubs might be homologous across photaria even if TZ spokes are not. Thus the double spokes of *Calkinsia* may have evolved independently of the single-filament spokes of corticates despite both possibly being linked to homologous hubs. Further work is needed on postgaardian and other discicristate TZs ideally by tomography to test this. In sum, *Calkinsia*'s immensely longer TZ probably evolved by enormous axial multiplication of (a) axosomal and (b) simple hub-spoke structures.

## Non-ciliary characters shared by Rhodaria

In addition to the novel ciliary TZ explained above, apparently ancestral characters of Biliphyta and of Plantae that like unstacked thylakoids with phyobilisomes were lost in Viridiplantae, I found two other rare characters shared by *Picomonas* and *Rhodelphis*.

A large curved smooth ER cisterna that separates their cytoplasm into two regions (one devoted to prey uptake and digestion; one containing major organelles like centrioles, nucleus, Golgi, mitochondria, and microbodies); it curves around the latter organelles, closely adhering to outer membranes of the mitochondrion and microbodies (Seenivasan et al. 2013; Gawryluk et al. 2019). Possession of a giant mitochondrion-linked smooth ER cisterna (mt-linked smooth ER or mtSER) partitioning the cytoplasm into feeding and organelle-rich zones appears to be unique in eukaryotes to these two genera and thus corroborates their relationship independently of the two TZ characters and sequence trees. The only other protist I know with a similar giant smooth ER cytoplasm-partitioning cisterna is *Platysulcus* (Shiratori et al. 2015), the most divergent of all heterokonts on multiprotein trees (Thakur et al. 2019). However, though its cisterna is similarly large and partitions the same organelles from the digestive zone, it is not specifically broadly attached to the mitochondrion; thus mitochondrial linkage is uniquely shared by *Picomonas* and *Rhodelphis*, not the smooth cisterna per se. Its presence in a heterokont chromist suggests that similar cisternae either already occurred in early corticates or arose independently. One possibility is that this smooth cisterna might be evolutionarily related to cortical alveoli, which I have argued were the ancestral condition in Corticata (Cavalier-Smith 2018; Cavalier-Smith et al. 2018), and could have evolved by becoming detached from the plasma membrane. An independent example of cell partitioning by a smooth cisterna into a trophic zone for food catching (in this case by reticulopodia) and digestion and a genetic/biosynthetic organelle-rich zone is the membrane surrounding the central capsule of rhizarian Ectoreta (Cavalier-Smith et al. 2018); this also was suggested to have evolved from cortical alveoli detached from the cell surface but differs from that of *Platysulcus* by completely surrounding the nuclear/organellar zones except for large pores to allow axopodial mts to pass between the two cytoplasmic compartments. That symmetric arrangement is possible only in protists like Ectoreta with aciliate trophic phase.

*Picomonas* and *Rhodelphis* mitochondria are distinct from most Plantae in having tubular cristae rather than irregularly flattened ones that sometimes seem nearer tubular as in photosynthetic Plantae. Their mitochondria also uniquely have two major electron dense granular inclusions, first discovered in *Picomonas* (called edms1 and 2), where they appear associated with mitochondrial membranes (Seenivasan et al. 2013). *Rhodelphis* has morphologically similar dark condensations in the mitochondrial matrix, but not closely associated with envelope membranes (Gawryluk et al. 2019). If those of *Picomonas* are also in the matrix, not within the intermembrane space they can also be regarded as rare ultrastructural shared characters that could have evolved like the TZ characters in the ancestral rhodarian. Gawryluk et al. (2019) overlooked the similarity of these genera in these four respects and the close similarity of their centriolar roots to those of Glaucophyta.

## *Rhodelphis* and *Cyanophora* centriolar root homologies

Gawryluk et al. (2019) were apparently unaware of the extremely thorough 3D-reconstruction of centriolar roots of the glaucophyte *Cyanophora cuspidata* (Heiss et al. 2017), which corrected previous errors. Consequently they seriously misinterpreted their own micrographs, overlooking their remarkable near identity with *Cyanophora* roots. They noticed only three microtubular roots, two wide bands (wmb1, wmb2), assuming that one belonged to each centriole and a narrow anterior band of three mts. *Cyanophora* also has a 3 mt anterior root and three wide roots of approximately nine mts each, two posterior and one anterior. Careful examination of their micrographs and critical comparison with Heiss et al. data and reconstructions lead me to conclude that *Rhodelphis* like *Cyanophora* must also have three wide roots.

Figure 1a and m of Gawryluk et al. show that in *Rhodelphis* also a wide root runs on *each* side of the posterior centriole, which must be homologues of the left and right posterior roots of Cynanophora. Furthermore if their Fig 1n is genuinely the anterior centriole it has a curved root of 9 mt on one side and one of 2 mt plus a singlet on the other. As in *Cyanophora* this wide mt root labelled wmb1 cannot be the same root as that labelled wmb1 in Fig. 1m. The fig. 1n wide root has a putative multilayered structure on its concave face (not noted by the authors) exactly as does the 9-mt anterior wide root (AWR) of *Cyanophora*; thus it must be the *Rhodelphis* AWR homologue and the 3 mt root the homologue of the anterior narrow root (ANR) of *Cyanophora*. The wmb1 in 1m has 9 (+1 offset) mts and dense fibrillar material on both surfaces; it is probably the *Rhodelphis* posterior right root (PRR) which in *Cyanophora* has 8 mts plus the Xmt making 9 altogether, with the posterior multilayered structure on one surface and the multilayered connective on the other. The 'striated structures' on their Fig 1k, l are probably the lamellar parts of the anterior and posterior multilayered structures. I presume that the wmb2 in their Fig. 1m is the posterior left root (PLR) but without serial sections it is probably not possible to identify correctly every posterior root example in their micrographs, so I have been unable to count the mts in PLR or to determine whether it is split, or the various fibrillar roots are as complex as in *Cyanophora* as is highly likely. However the anterior mt roots are identical and on available evidence no differences have been identified between the posterior mt bands of *Rhodelphis* and *Cyanophora*. Moreover the two multilayered structures are identically positioned and at least some other equivalent fibrillar roots are the same. Gawryluk et al. (2019) implies that there is a single striated connective but their Fig. 1j, k seem to show three distinct parallel ones. Their descriptions of the wide mt roots are inaccurate as three positionally and structurally distinct ones are conflated into two. I conclude that all ciliated Biliphyta have four distinct and highly conserved mt roots.

## Revision of kingdom Plantae

I revise kingdom Plantae Haeckel 1866 em. Cavalier-Smith 1981 by grouping classes Picomonadea and Rhodelphea as new phylum Pararhoda, within subkingdom Biliphyta Cavalier-Smith 1981, and creating new infrakingdom Rhodaria to group Pararhoda with phylum Rhodophyta Wettstein, 1922:

### Diagnosis of Rhodaria infrak. n

Red algae plus biciliate phagotrophic heterotrophic phagotrophs with long ciliary transition zone having just distal to the ciliary constriction a dense, distal annular septum surrounding the central pair microtubules about 0.5 μm above the transitional plate to which the central pair (cp) microtubules are attached by a long, thin hub; cp below the constriction surrounded by a nonagonal tube linked closely to A-tubule feet. Phylogenetically comprises the most inclusive clade including classes Picomonadea Seenivasan et al. 2013, Rhodelphea (Gawryluk et al. 2019), and phylum Rhodophyta, but excluding Glaucophyta and Viridiplantae. **Comment.*** Rhodelphis* alone was previously added to Plantae (using the unnecessary junior synonym 'Archaeplastida') by Gawryluk et al. 2019 who did not realise it belongs specifically in subkingdom Biliphyta or that it is ultrastructurally closer to *Picomonas* than any other eukaryotes. As they referred only to their inaccurate phylum diagnosis when introducing the class and ordinal names, I provide more accurate diagnoses:

### Class Rhodelphea

Tikhonenkov, Gawryluk, Mylnikov, and Keeling in Gawryluk et al. 2019. **Revised diagnosis:** Phagotrophic biciliates with orthogonal centrioles linked by three striated connectors; cryptic non-photosythetic plastids with Toc/Tic import proteins but no genome. Anterior centrioles with one narrow microtubular root of three microtubules and a wide root with multilayered structure; posterior centrioles with wide left and right microtubular roots, the right with a multilayered structure; without feeding groove or cytostome. Ciliary transition zone with transition plate close to cell surface to which the central pair microtubules attach and distal to that plate a long TZ with long nonagonal tube attached to A-tubule feet; distal to that is a smaller diameter loose transition helix followed by an annular dense septum surrounding the central pair microtubules just distal to the ciliary constriction and level with the single annular connexion. Distal TZ has Y-links and A-B links but no doublet arms or spokes. Curved smooth ER cisterna separates cytoplasm into two regions (one devoted to prey uptake and digestion; one containing major centrioles, nucleus, Golgi, mitochondria, and microbodies; it curves around the latter organelles, closely adhering to outer membranes of the giant mitochondrion and microbodies.

### Order Rhodelphales

Tikhonenkov, Gawryluk, Mylnikov, and Keeling in Gawryluk et al. 2019 orth. em. **Revised Diagnosis:** as for class Rhodelphea plus cell surface covered in thick layer of glycostyles; posterior cilium with one row of simple hairs; ingest whole eukaryote cells. Currently includes only family Rhodelphidae Tikhonenkov, Gawryluk, Mylnikov, and Keeling in Gawryluk et al. 2019 (here orthographically corrected to Rhodelphidaceae with same type *Rhodelphis*).

**Comment:** class and order described under the International Code of Nomenclature for algae, fungi, and plants which for simplicity is best applied to the entire kingdom Plantae even for those members that are neither algae, fungi not Embryophyta. Thus the suffix–ales is preferred over–ida for the order, but the suffixes–phyceae–mycetes or mycota- or -phyta would not be appropriate for phagotrophic heterotrophs that are neither algae nor fungi, so this code's phenotypically biassed and inappropriate recommendation on suffixes at class and phylum a rank should not be followed. The new diagnoses for the class and order are more accurate than earlier for phylum Rhodelphidia (Gawryluk et al. 2019) which wrongly stated there were only three centriolar microtubular bands and implied a single striated connector, and did not mention TZ structure, multilayered structures, glycostyles or cytoplasmic partitioning by a smooth cisterna. As Rhodelphea has fundamentally the same body plan as class Picomonadea Seenivasan et al. 2013 I group both as a new phylum placed with Rhodophyta within Rhodaria; separate 'phyla' Picozoa, Rhodelphidia are unnecessary:

### New phylum Pararhoda. Diagnosis

Non-photosynthetic biciliates without feeding groove or cortical alveoli but with cytoplasm separated into two regions (one devoted to prey/food uptake and digestion; one containing major organelles: centrioles, nucleus, Golgi, mitochondria, and microbodies) by a large smooth ER cisterna that curves around the latter organelles, closely adhering to mitochondrial outer membranes. Giant mitochondrion with tubular cristae and matrix dense inclusions. Centrioles orthogonal or at an obtuse angle, each with two microtubular roots. Ciliary transition zone with transition plate close to cell surface to which central pair microtubules attach and distal to that plate a long TZ with long nonagonal tube attached to A-tubule feet; distal to that is an annular dense septum surrounding the central pair microtubules just distal to the ciliary constriction and level with the single annular connexion. Cryptic plastid may be present. Comprises eukaryovorous class Rhodelphea with surface glycostyles, posterior ciliary hairs, distal transition helix, and two multilayered structures; and secondarily miniaturised pinocytotic class Picomonadea without glycostyles, ciliary hairs, transition helix, or multilayered structures. Differ from Glaucophyta by predatory not photosynthetic nutrition and lacking cell walls or anterior ciliary hairs. **Etymol:*** Para* Gk beside, beyond + *rhodos* Gk rose, rose-red; emphasises that it comprises the closest outgroups to Rhodophyta. **Comment:** given the very sparse genic sampling of *Picomonas* the non-maximally supported evidence that Pararhoda may be paraphyletic rather than holophyletic is not convincing. Even if it were, it should not override the strong evidence for fundamentally similar body plans for both classes—insufficiently different ultrastructurally for separate phylum rank. Correcting the interpretation of *Rhodelphis* mt roots makes both Pararhoda and Biliphyta homogenous not only for TZ but also for ciliary root structure; non-cruciate asymmetric pattern of four different roots, two with multilayered structure except in the highly simplified picomonads, which nonetheless retain all four dissimilar roots.

## *Rhodelphis* and the origin of kingdom Chromista

Discovery that *Rhodelphis* must have a cryptic plastid with Toc/Tic envelope translocators means that red-alga-like import machinery existed earlier than the last common ancestor of crown Rhodophyta. This raises the possibility that the alga enslaved by the first chromist was not a crown red alga as often assumed but an older stem rhodophyte or rhodarian. Previous multiprotein analyses of chloroplast phylogeny have been contradictory concerning whether chromist plastids evolved from crown rhodophytes or are their sisters (e.g., Yoon et al. 2005, Kim et al. 2015), and ML trees do not even consistently resolve the basal branching order of chromists and do not always show them as holophyletic. Some separate red algae into two clades and some or all suggest that chromists might be less closely related to cyanidiophyte red algae than to the rest of the red algae (e.g., a 93-protein tree: Kim et al. 2015) but that was strongly contradicted by their 93 chloroplast *gene* tree which strongly showed red algae as holophyletic and Hacrobia and then heterokonts as their outgroups. The new cyanobacteria-rooted Toc75/Omp85-like tree including both *Rhodelphis* sequences (Gawryluk et al. 2019 fig. 9e) appears particularly robust with almost every node maximally supported including that for *Rhodelphis* being sister to Rhodophta plus Chromista. In that tree red algae are maximally holophyletic as are halvarian chromists, which implies that chromist plastids came from stem, not crown. Unfortunately that tree did not include glaucophytes or Hacrobia, but it does suggest that the origin of the chromist plastids was substantially earlier than the primary divergence of crown rhodopyte (into cyanidiophytes and the rest) so chromist plastids did not come from crown but from stem red algae rather soon after it diverged from its rhodelphid sisters, i.e., extremely early in diversification of Rhodaria.

That tree also includes an OMP85 protein from the cercozoan *Bigelowiella natans* whose ancestors enslaved a green alga to make its green chloroplast and nucleomorph. This sequence does not group within Viridiplantae as it would have done if it originated by the green secondary symbiosis, not within crown red algae as it would if it were a relatively recent (post chromist origin) lateral gene transfer from a rhodophyte, but is sister to halvarian clade (92% bootstrap support) in conformity with multiprotein trees of chromist subkingdom Harosa. This important result is the first direct and strong sequence tree evidence that Rhizaria ancestrally had a red algal plastid. The most reasonable interpretation is that ancestral Rhizaria had a plastid stemming from the same red algal enslavement that generated photosynthetic harosan chromists. This does not mean that it was photosynthetic or still had a red algal plastid genome. It may simply have had a non-photosynthetic plastid of red algal origin, either one retaining a genome or one that had already lost its genome but still retained a nuclear encoded plastid-import machinery like *Rhodelphis*. This chlorarachnid (former red algal) plastid would inevitably have been lost after its Toc75 and at least some other proteins were transferred into the newly enslaved green alga; thus chlorarachnid green algal enslavement was probably a *plastid replacement* closely similar to that generating green dinoflagellate chromists (*Lepidodinium*; see Cavalier-Smith 2017a, 2017b, 2017c. Plastid replacement (an obvious possibility raised by Archibald 2012) is a simpler explanation of the origin of many of the other eight *Bigelowiella* plastid-targeted proteins previously suggested to have come from red algae or heterokonts by multiple lateral gene transfers (Archibald et al. 2003), e.g., rpl1, rpS22, acyl carrier protein, glutamate-1-semialdehyde 2,1-aminomutase, fructose-1,6 bisphosphatase, geranylgeranyl reductase.

The cryptic former-red-algal plastid would have preadapted the stem chlorarachnid lineage to the secondary enslavement of a green alga by making both chloroplast-to-nuclear gene transfer and retargeting their proteins to the enslaved green alga unnecessary for the protein-import machinery. Instead they could simply have added a signal sequence to preexisting nuclear-coded plastid proteins targeting former red algal Toc75 and lost the green algal Toc75, thus functionally replacing it. This evidence for plastid replacement in the ancestral chlorarachnid provides strong support for the idea that the encestral chromist had a red-algal plastid that was multiply lost in all major chromist lineages (Cavalier-Smith et al. 2015; Cavalier-Smith 2018). Together with the evidence that a hacrobial ancestor had such a plastid that acquired a novel bacterial gene this virtually proves that both chromist subkingdoms Harosa and Hacrobia ancestrally had a red algal plastid. It is important to study the source of Toc75 and other putatively plastid-replacement-derived proteins from other chlorarachnids and from Hacrobia to test this conclusion rigorously. If Toc75 trees including Hacrobia confirm that all chlorarachnid Toc75s are more closely related to Toc75 of other Harosa and none nest within Hacrobia, that would rule out the unlikely theoretical possibility that it came by lateral gene transfer from a hacrobian, making it entirely unreasonable to argue any longer that the first chromist had no plastid. Further corroborative evidence could come from taxon-rich phylogenies of the other 6 proteins listed above. Ancestral photophagotrophy of Chromista is close to being firmly established.

## Revision of kingdom Protozoa

Kingdom Protozoa originally included unicellar algae and bacteria as well as heterotrophic unicellular eukaryotes (Owen 1858) and was always recognised as ancestral to kingdoms Plantae and Animalia. When establishing kingdom Chromista (Cavalier-Smith 1981), I refined kingdom Protozoa by excluding prokaryotes, chromists, and unicellular Plantae, and made a preliminary classification of the core Protozoa into phyla, of which only Ciliophora and Euglenozoa retain that rank. Nearly two decades later, ultrastructural advances, organismal discovery, and rDNA trees led to a major revision (Cavalier-Smith 1998) in which subkingdoms Archezoa (with phyla Metamomada and Parabasalia) and Neozoa of 11 phyla were recognised, including new phylum Cercozoa and greatly refined Amoebozoa; phylogenetic demarcation from Animalia was improved by transferring Myxozoa to Animalia despite secondary amoeboid vegetative unicellularity of most species, but Microsporidia were erroneously transferred to Fungi. This error persisted in the simplification to nine phyla (Parabasalia being transferred to Metamonada: Cavalier-Smith 2003b) and improvement of their monophyly by Cavalier-Smith (2003a) when corticates were first recognised as a major supergroup. Kingdom Protozoa took essentially its present circumscription when I transferred Alveolata, Rhizaria, and Heliozoa to new subkingdom Harosa of Chromista (Cavalier-Smith 2010). That left seven phyla in Protozoa, four in subkingdom Eozoa, three in subkingdom Sarcomastigota, the latter later revised by including Apusozoa within broader new phylum Sulcozoa (Cavalier-Smith 2013).

During that period ideas about the root of the eukaryote tree repeatedly changed in light of new evidence. Since Cavalier-Smith (1978), I repeatedly sought to understand ciliary origins and to find ciliary characters that might securely indicate the root of the eukaryote tree (e.g., Cavalier-Smith 1981, 1982a, 1982b, 1991c, 2000, 2002, 2014). I remained unsatisfied with these attempts and tried to find molecular evidence for the root (Stechmann and Cavalier-Smith 2002; Stechmann and Cavalier-Smith 2003; Cavalier-Smith 2003a, 2003b; Cavalier-Smith and Chao 2020) as well as centriolar root patterns (Cavalier-Smith 2013, 2017a Fig. 8, 2018 Fig. 2) but these also proved unconvincing. Now however, placing the root between Malawimonada and all other eukaryotes is strongly supported by the congruence of evidence from malawimonad's unique ciliary TZ and TF simplicity and from 27-protein trees rooted on eubacteria. This provides a firmer basis for distinguishing between paraphyletic and holophyletic protozoan groups (Fig. 11). The fundamental contrast in TZ between malawimonads and discaria (a clade, not treated as a taxon) leads me to establish new phylum and subkingdom Malawimonada to stress their primary divergence from all other eukaryotes. I divide protozoan discaria into two further subkingdoms according to whether they belong to the dorsate or the natate clade of Fig. 11. Placing malawimonads within a phylum Neolouka (Cavalier-Smith et al. 2015) is no longer meaningful as they are not a derived type of excavate as suggested earlier (Cavalier-Smith 2018) but the earliest eukaryote subclade.

Since Apusozoa (Apusomonads and Breviatea) and Varisulca were first treated as subphyla of a single phylum (Sulcozoa) with dorsal pellicle, ventral groove, and usually pseudopods (Cavalier-Smith 2013) there have been great advances in their ultrastructure and multiprotein phylogeny. In their light I consider the cellular differences between them sufficient to merit phylum rank and therefore remove Apusozoa from Sulcozoa as a separate phylum (thus restoring its original rank). In the light of multigene phylogeny (Lax et al. 2018) I now accept the original phylum rank for Hemimastigophora (Foissner et al. 1988) that was previously controversial, and transfer this group from Chromista to Protozoa. I accept Opisthosporidia as a major taxon embracing microsporidia, aphelids, and rozellids distinct from phylum Choanozoa (Karpov et al. 2014) but their ranking as a superphylum is superfluous taxonomic inflation, so I reduce its rank to phylum since its subgroups differ too little in phenotype to merit phylum rank; as explained in Ruggiero et al. (2015), I strongly agree with Karpov et al. in treating these phagotrophs as Protozoa not Fungi. These changes increase to 11 the protozoan phyla I now recognise, of which seven are clades. These phyla include 42 classes, all apparently clades. Table 1 summarises the thus revised higher classification of kingdom Protozoa. This revision splits natate and dorsate protozoa into subkingdoms Natozoa (ancestors of chromists and plants) and Sarcomastigota (ancestors of animals and fungi). I here briefly explain these and other improvements at intermediate higher ranks.

Given the tree rooting beside Malawimonada, earlier subkingdom names Eozoa (now only one of three natozoan clades) and Neozoa (Cavalier-Smith 1997) seem inappropriate as the former is not ancestral to the latter as assumed when they were established; instead natate and dorsate Protozoa are sister groups of equal age. Therefore instead of retaining Eozoa with substantially modified circumscription, I propose new subkingdom Natozoa (meaning swimming life) for natate Protozoa to emphasise that they were ancestrally swimmers not gliders as were dorsate protozoa, and that they are predominently planktonic (main exceptions heterotrophic euglenoids and amoeba phase of Percolozoa) not benthic as are almost all Sarcomastigota except diphylleids and acanthoecids which feed whilst actively swimming. Dorsate Protozoa approximate to Sarcomastigota (Cavalier-Smith 1983) as revised by Cavalier-Smith (2010) with the inclusion of all, not just some, Sulcozoa. I therefore revise Sarcomastigota in this way as its name remains phenotypically nicely descriptive of the organisms included. This introduces not only two new subkingdoms, each with five phyla but also new infrakingdoms. Within Sarcomastigota I group opisthokont phyla Choanozoa and Opisthosporidia together as Opizoa, a useful name for those who wish to refer to 'unicellular opisthokonts other than Fungi and Myxozoa'—more precise than the clumsy and inaccurate term 'unicellular opisthokont' in many paper titles. I also create infrakingdom Diacentrida to group phyla Apusozoa and Amoebozoa, whose biciliate ciliated members mostly have two full strongly divergent centrioles, in contrast to Sulcozoa where they are orthogonal. Opizoa, which evolved from them by posterior ciliary suppression retained its centriole in reduced form. By contrast when *Phalansterium* and Archamoebae in Amoebozoa independently lost the posterior cilium they also lost is centrioles, so suppressed ciliary transformation and became truly unikont. The new subdivision of Sarcomastigota into three infrakingdoms represents three successive grades of bikont ciliary organisation: ancestrally with orthogonal centrioles with the posterior one attached to the side of the anterior one (Sulcozoa); then stronger divergence of the posterior centriole giving an obtuse-angled kinetid (Diacentrida); then suppression of the posterior cilium followed by divergent adjustments to the position and angle of the shorter (older) barren centriole in opisthokonts.

In Natozoa I downrank Eozoa as circumscribed since Cavalier-Smith et al. (2015, i.e., excluding Metamonada, which Ruggiero et al. 2015 included) to infraphylum. The name Eozoa is older than unranked Discoba (Hampl et al. 2009) for this clade and was proposed for discicistates plus the hydrogenosome-containing Parabasalia and Anaeromonada (Cavalier-Smith 1987a, 1987b); it was originally intended to embrace the most primitive mitochondrial eukaryotes considered more advanced than the amitochondrial Archezoa. Given the new rooting of eukaryotes, it seems appropriate to divide ancestral Natozoa into three infrakingdoms, each a clade: (1) deepest branching Archezoa containing only the anaerobic tetrakont Metamonada; (2) next branching ancestrally aerobic Eozoa containing bikont (Eolouka and Euglenozoa) or double bikont (Percolozoa, mostly with amoeboid phases) lineages, with some secondary anaerobes; and (3) third deepest branching aerobic Hemimastigophora with rows of unikont kinetids. These three infraphyla constitute the three phenotypically most divergent natozoan groups, so merit this high rank.

One basal kingdom Protozoa suffices for all eukaryotes outside Animalia, Fungi, Plantae and Chromista. It is best to keep the number of highest taxa as low as we reasonably can, reserving kingdoms for the greatest phenotypic disparities only (Cavalier-Smith and Chao 2020). An attempt at Linnean ranking in Table 1 of Adl et al. (2019) was very incomplete, poorly judged, self-contradictory (higher ranks within lower ones!) and not a balanced Linnean classification, unsurprising given extreme past prejudice against traditional ranking. That table is substantially discordant with ranks denoted by suffixes of the names used and with standard practise of most taxonomists (e.g., Ruggiero et al. 2015, who were harshly and incorrectly criticised by Adl et al. (2019) for things they did not do). Adl et al. (2019) though a useful compilation failed to appreciate the principles of sound ranking. Proper use of sub-, infra-, and super- taxa enable us to restrict the number of higher-rank taxa so as to get the maximum simplifying benefit from hierarchical classification. Contrary to Lax et al. (2018) there is no merit in treating Hemimastigophora as a superkingdom. Nor would it be taxonomically beneficial to increase the number of protozoan phyla to 16 by splitting the four paraphyletic phyla into extra phyla; their major subclades have insufficient disparity in body plans to merit that. But we must avoid collapsing great disparity into one low-ranked group.

Ignoring the longstanding phylum Percolozoa (Cavalier-Smith 1991b, 1993c) and the fact that Percolozoa always embraced *several* classes differing greatly in ultrastructure, especially ciliary roots, Adl et al. (2019) confusingly lumped all under the original class name Heterolobosea, following the lead of those averse to ranking, who criticised the concept of phylum Percolozoa embracing several classes of which Heterolobosea was only one, basing their erroneous criticisms on studying two flagellates then misidentified as *Percolomonas* (Brugerolle and Simpson 2004), leading them to misjudge the distinctiveness of Percolomonadida and percolozoan classes. Those flagellates misnamed '*Percolomonas*', now assigned to new genera *Harpagon* and *Pseudoharpago*n, are here placed in class Lyromonadea, *not* Percolomonadea. An ill-considered sliding of the meaning of Heterolobosea (originally just eruptively amoeboid Acrasida and Schizopyrenida, the latter amoeboflagellates usually with rostrum, cytopharynx, but neither feeding groove nor ciliary root R1: Page and Blanton 1985) by those dogmatically against ranking to embrace all extremely different Percolozoa that fall well outside the original heterolobosean phenotypes seriously confused percolozoan classification for years. Table 1 therefore includes partial revision of phylum Percolozoa to make it evolutionarily sounder and up-to-date by establishing new subphylum Orthozoa, with orthogonal centrioles, not parallel ones, whose members all differ radically from classical class Heterolobosea, and explicitly listing key features of all six percolozoan classes (two new) needed to do justice to their remarkable cytoskeletal and ciliary diversity. Thus there are now five percolozoan classes of substantially different phenotype from classical Heterolobosea.

## Opisthokont transition zone evolution

When naming the opisthokont clade (Cavalier-Smith 1987b) I pointed out similarities between the microtubular/striated centriolar roots of choanoflagellates like *Codonosiga* (Hibberd 1975) and monoblepharid and certain spizellomycete fungi as shown in Fig. 18, also stressed by Barr (1992). I argued that Fungi and animals independently evolved from phagotrophic choanoflagellates, later explaining both events in more detail (Cavalier-Smith 2001, 2017b, 2017c). TZs also give independent, unexpectedly strong, ultrastructural evidence for opisthokont phylogenetic unity congruent with sequence trees, as shown below.

Figure 18 reveals an overlooked fundamental unity in centriolar root and TZ ultrastructure between choanoflagellates, chytridiomycete fungi, *and* sponges. Many chytridiomycete fungal TZs have a dense plug at the ciliary base, which obscures much ultrastructure and has prevented their TZs from being properly understood developmentally and evolutionarily. It has been overlooked that many choanoflagellates and virtually all sponges have a similar dense TZ plug. I give the first comparative evolutionary ultrastructural interpretation of the fundamental nature of the chytridiomycete TZ plug based on overlooked substructures in exceptionally clear micrographs by Longcore and Simmons (2012, Figs. 3, 4, 6, 8, 10) of order Polychytriales, which have longer barren procentrioles than other Chytridiomycota, often nearly mature length, and more strongly developed centriolar connectors, likely retained ancestral characters in this early diverging lineage. As for TZs generally, the dense plug has two fundamentally distinct components. Between the doublets and ciliary membrane of *Polychytrium aggregatum* are about six dense serrations that represent Y-links more strongly stained than usual (Fig. 18A). Inside the doublets is a prominent TH that extends almost to the base of the TZ doublets; its proximal part distal to the putative TP is more medially sectioned, but its distal part is more peripheral and shows the peripheral lattice well (Fig. 18A). Immediately proximal to the putative TP the asterisk marks the putative acorn complex, visible in TS in Fig. 18E of *Neokarlingia chitinophila* (also Polychytriales). The next (more distal) section of the series (Fig. 18D) shows three superimposed structures: a fine lattice with circumferential and radial components that I consider the TP lattice; and grazes two others— the base of a cp mt and three sides of a nonagonal fibre. The juxtaposition of cp and TP within a single section implies that cp passes through the centre of the plug to its very base, which a more median *Polychytrium* section supports (Fig. 18B).

A cp passing through the TH centre is clearly seen in another polychytrid, *Karlingiomyces asterocystis* (Fig. 18F); as in *Polychytrium*, TH is differentiated into two parts—the longer distal zone that encloses cp to its base midway between cp and doublets is a zigzag in LS similar to the heterokont TH, connected to the doublets by more linkers and a peripheral lattice; the shorter proximal zone extends from the base of cp to the putative TP, seemingly not zig-zag and a little denser, corresponds positionally with the proximal basal zone in *Polychytrium* where dense matrix almost hides the lattice. A TS of this species embracing the doublet/triplet junction includes the base of both cp mts, a TP star-like lattice and hints of an acorn filament showing how thin the TP must be and proving the cp goes right through the TH (Fig. 18I left); even the barren centriole has a distal cap (Fig. 18F) with perhaps an acorn/TP-like substructure (in TS in Fig. 18I right). *Maunachytrium* (order Lobulomycetales; Fig. 18H) and *Catenochytridium* (which I place in Chytridiales: later section) also clearly have cp within a basal TH just above TP.

Demosponge embryo cilia from at least five orders of both major subclasses (Maldonado 2004; Gonobobleva and Maldonado 2009; Woollacott and Pinto 1995) invariably show in LS an obscuring dense plug distal to the TP (Fig. 18P, Q), which in TS is seen as a transitional cylinder (Fig. 18J) at the level of Y-links. Just proximal (level with TFs) is an ill-defined TP with at least a peripheral lattice with doublet- and interdoublet-facing radial elements. As in fungi the cylinder is probably a TH, present at least in the common ancestor of animals and fungi, and thus opisthokonts; if it is homologous with the basal cylinder of Sulcozoa, it arose earlier still. Three demosponge orders have retained mts stemming from a striated basal foot beside the ciliated centriole that radiate asymmetrically parallel to the apical cell membrane similarly to choanoflagellates, monoblepharids and a minority of Chytridiomycetes; *Halichondria* and *Halisarca* also retain direct attachment to a pointed nucleus similarly to Blastocladiales and many Chytridiomycetes. Calcareous sponge choanocytes have a longer dense plug further from the ciliary base whose structure is unclear (Eerkes-Medrano and Leys 2006 Fig. 5J). Thus a dense plug including a TH is general in sponges. It might have evolved in the common ancestor of all opisthokonts and was lost in other animals and in some fungal lineages.

Choanoflagellates (with type II TZ) are consistent with an opisthokont propensity to lose plugs, some showing clear dense plugs (e.g., *Desmarella* (Fig 18W)) and some seemingly lack them (e.g., *Codonosiga* Fig. 18Z,a). That presence or absence of a dense plug is a phylogenetically trivial aspect of differential staining is shown by *Monosiga ovata* where some cells show a densely stained plug that obscures TZ structure (Fig. 18X) on either side of TP, which cannot be seen, and another in the same fixation shows a discrete TP at the ciliary constriction at the same position as the more normal thin TP with thicker central disc in *Codonosiga* (Fig. 18Y). However choanoflagellate plugs are apparently proximal to the TP and thus probably not homologous with those of sponges and fungi. Choanoflagellates of order Craspedida all have a central filament (of variable length, often with radial spokes to doublets) linking the central dimple on TP to a small axosome at the base of cp (Fig. 18U, w-Z , a), which is absent in order Acanthoecida (Fig. 18G, L; at least in all the tectiform subclade) contrary to Karpov and Frolov (1995) who said it was found in all choanoflagellates and Karpov et al. (2018) who incorrectly wrote it is present in *Stephanoeca diplocostata* (Fig. 18G shows one of its cp mts only extending to TP, a situation perhaps confused with the craspedid filament). As the central filament is also absent in it is clearly absent across tectiform acanthoecids and likely restricted to Craspedida, which therefore have a longer TH than Acanthoecida, presumably a difference correlated with their different feeding modes. Most choanoflagellates have a plug just proximal to TP (similar in size to the centriole) which may stain moderately allowing some substructure to be seen if densely. Fig. 18Z, a of *Codonosiga* hint that this plug has peripheral densities like a TH and also tilted spiral densities. In choanoflagellates this TH-like variably staining plug is definitely proximal to TP. In fungal zoospores TP position has been hard to establish. Often it is thought to be absent; sometimes it has been labelled proximal to the plug just above, but I argue below that most such cases are probably really the acorn-homologue, not TP). If my TP label in Fig. 18A is correct then *Polychytrium* would have a type I TZ which could be converted into a type II as in choanoflagellates by moving TP relative to TFs, but maintaining the position of the plug relative to TFs. A few micrographs suggest that some choanoflagellates may have a faint, thin TH around the basal part of the distal TZ, e.g., *Monosiga* (Fig. 18Y), but this is never as obvious as in sponges or many fungi.

In sponges also the TP is probably just below the plasma membrane, not above it as in choanoflagellates, thus a type I TZ (Maldonado 2004; Gonobobleva and Maldonado 2009). LSs of the demosponge *Halisarca* (Fig. 18O) and haplosclerid demosponge *Stigmadocia* (Fig. 18J, K) show a TH inside the doublets whose dense staining is mainly responsible for the plug appearance. In *Stigmadocia* the TH is longer than the centriole and cp clearly extends distally from the TP and TH surrounds the cp as seen in TS (Fig. 18J); Fig. 18K is a grazing section of the junction of TH and cp at the level of TFs. The *Halisarca* section shows TH clearly distally but it is unclear whether the dense plug below the apparent end of cp is an artefact of section obliquity or if the proximal part of TH is actually packed with dense material.

Opisthosporidia, the protozoan sisters of Fungi, have uniciliate zoospores in aphelids (TZ medium) and rozellids (TZ very long) that do not exhibit dense plugs (Letcher et al. 2017a, b; Karpov et al. 2019) or unambiguously position the TP in aphelids. *Paraphelidium tribonematis* has a very short centriole and Fig. 8G and J of Karpov et al. (2019) appear to be of acorns in TS and slightly more distal 7F level with TFs and Y-links may graze a weakly stained TP lattice; but no TH. *Aphelidium tribonematis* has a plug of medium density material positioned proximally to cp much as in *Halisarca* which resembles a basal cylinder/TH in LS *(*Karpov et al. 2019 Fig. 6D-I, but the LS looks odd and might be freakish). In *Aphelidium chlorococcalium* the filament at the triplet doublet junction linking five A-tubule feet labelled 'coil fibre' (Karpov et al. 2019 Fig. 2F) is more likely the peripheral acorn filament, whereas the slanting filaments distal to SFs called proximal diaphragm in their Fig. 2H are more likely part of a TH (just visible also in 3L). Letcher et al. (2017b) labelled (probably correctly) a 'spiral fibre' in the same position proximal to the base of cp in *Aphelidium desmodesmi*. *Rozella rhizoclosmatii* has very short centrioles and immensely long TZ (1 μm) apparently with a clear thin TP just below TFs. I conclude that *Aphelidium* has a TH (likely distal to TP) probably homologous with those of Chytridiomycetes and sponges, though micrographs are not very informative. This has almost certainly been lost by *Paraphelidium* and probably also by *Rozella*.

## Transition zones in opisthokont outgroups: diacentrids

The nearest relatives of opisthokonts (closest apusomonads, then breviates, then Amoebozoa) all ancestrally had two cilia whose centrioles point almost in opposite directions. I therefore now group them collectively as new protozoan infrakingdom Diacentrida (Gk *dia* apart emphasises this unusual kinetid arrangement, contrasting with the ancestral condition in discaria, where centrioles were orthogonal—and still are in deepest branching dorsates Varisulca and Planomonada and most natates except for a few derived groups where they secondarily became parallel, notably Euglenozoa, and heterokont Synurales). All three groups ancestrally had pointed cells with a long forward-pointing anterior cilium, its centriole joined basally by fibres to an anterior nucleus, and posterolateral cilium pointing backwards ventrally to the nucleus.

**Fig. 19.**
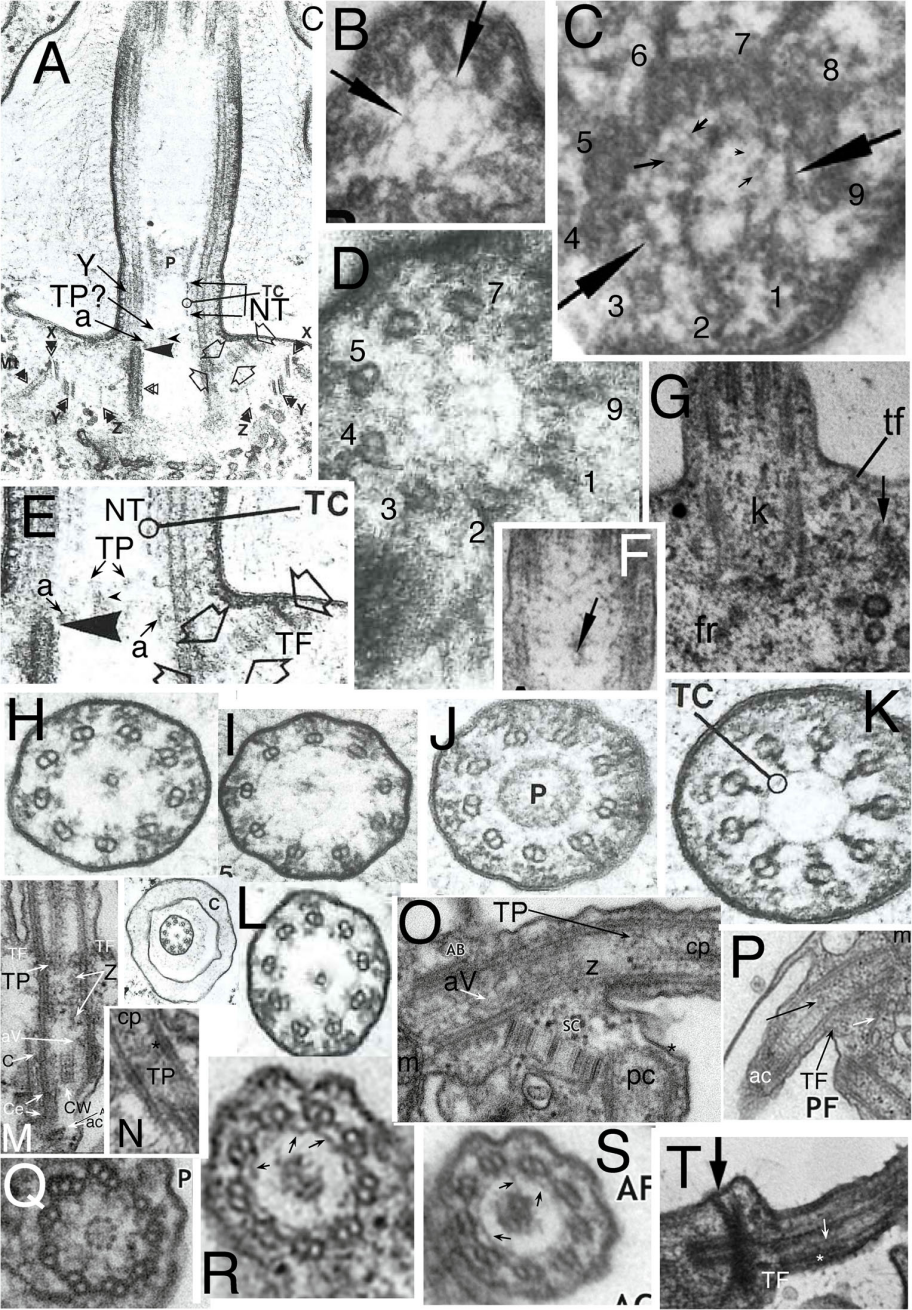
Early torcid transition zones: Amoebozoa and Apusozoa. **A, D, E, H-L.*** Phalansterium digitatum* (Amoebozoa:) from Hibberd (1983 figs 9, 10, 13-17) by permission. **B, C, F, G.*** Phalansterium arcticum* from Shmakova et al. (2018 figs 19A-C, E) by permission. **M, O, P, Q, R, S.*** Thecamonas trahens***(Apusozoa: Thecomonadea)** from Heiss et al. 2013a figs 2A 3D, F, C, 4A, 7B by permission. **T.*** Mastigella rubiformis* (Amoebozoa: Archamoebae) from Zadrobílková et al. (2015) fig. 7F by permission. **A.*** P. digitatum* swollen proximal TZ region without cp, showing plug (**P**), nonagonal tube NT (=TC), putative TP and acorn (**a**) plus linker between them (small arrowhead); open arrows indicate striated TF, double arrowheads concentric dense bands linking radiating mts. **B.*** P. arcticum* TS above TZ base showing 18-gonal/nonagonal tube (arrows)**C.*** P. arcticum* acorn complex at TZ base (proximal to **B**, in TF zone). **D.*** P. digiataum*acorn-V complex at doublet/ytiplet junction*.***C, D** doublet numbering after Geimer and Melkonian 2004). **E.** enlargement of **A** at centriole/TZ junction. **F.*** P. arcticum* TZ in LS showing central structure (originally identified as a mt), which might be a mt as in **H**, **L** or instead the central filament as in **I**. **G.*** P. arcticum* TZ LS. **H, L.** P. *digitatum* TSs in distal Y-link zone showing single central mt. **I-K** successively more proximal TSs of *P. digitatum* TZ showing central filament (**I**), plug (**P** in **J**) and nonagonal tube/'transition cylinder' (**TC** in **K**). **M.*** Thecamonas trahens* posterior cilium LS near base of highly asymmetric centriole and TZ. Zig-zagnonagonal/18-gonal tube (**Z**); **ce** centriole elongation on inner side; **cw** cartwheel. **N.*** Pygsuia biforma* (**Apusozoa: Breviatea**) oblique LS of TZ showing TP and axosomal plate (asterisk); from Brown et al. (2013 fig. 1h). **O.*** Thecamonas trahens* LS of anterior ciliary base linked directly to mitochondrion (**m**) and to posterior centriole (**pc**) by striated connector (**sc**); posterior centriole closely linked to cell surface (asterisk);**z** zigzag nonagonal/18-gonal tube. **P.*** T. trahens* LS of both centrioles showing large offset and thin connector (arrow); base of anterior centriole is linked to a mitochondrion (**m**); base of posterior (**AF**) beveled, at cell surface; **a** putative acorn. **Q.*** Thecamonas trahens* posterior centriole TS near base showing triplet, doublet and singlet mts. **R.*** Thecamonas trahens* anterior cilium at cp base showing peripheral 18-gonal star (arrows), central axosomal densities and faint radial linkers between them. **S.*** Thecamonas trahens* anterior cilium with nonagonal tube (arrows) around cp*.***T.** Tangential LS of *Mastigella rubiformis* (**Amoebozoa: Archamoebea**) ciliary base showing two-tiered circumferential centriolar root radiating from distal centriole with upper fibrillar sheet and lower radiating mts (Black arrow). Asterisk is dense cylinder outside the doublets similar to that of *Viridiraptor*. White arrow marks possible TZ basal cylinder.

Apusomonads are benthic, invariably glide on surfaces by their long posterior cilium, have a pronounced dorsal 'theca', and ventral pseudopods for feeding. The anterior cilium is surrounded by a cytoplasmic sleeve or collar, supported on both surfaces by a flexible extension of the submembrane proteinaceous 'thecal' (better, pellicular) layer, which together with the enclosed cilium forms a left-pointing flexible proboscis able to explore the environment by wiggling as cells glide. The posterior centriole ends at the cell's tip, contacting the plasma membrane at its base on one side (Heiss et al. 2013a) which might even be the ancestral condition for eukaryotes as it occurs in all Malawimonada (O'Kelly and Nerad 1999; Heiss et al. 2018) and all apusomonads, both with particularly short centrioles. The base of the posterior centriole is extended posteriorly on the side closest the cell apex, making it exceptionally acutely bevelled, implying that its mts can grow differentially proximally from the cartwheel (Fig.19M,P)—as can those of *Chlamydomonas* (O'Toole and Dutcher 2014). In both cilia the basal TZ is occupied by medium density material essentially indistinguishable from that of choanoflagellates, which in the posterior cilium has a zigzag appearance (Fig. 19M) and might therefore be related to the similarly zigzag lattice of opisthokont TH. Because the centriole has a mixture of triplets and doublets even in the cartwheel zone (Fig. 19Q) the boundary between it and the TZ is hard to define in *Thecamonas*. The peripheral zig zag is mainly proximal to the putative TP but apparently extends slightly distal to it (Fig. 19M); at the cp base in TS it appears as a fluted 18-gonal tube (Fig. 19R), or sometimes more nonagonal (Fig. 19S).

Breviate amoebae have pseudopodia but locomote mainly by ciliary gliding or swimming. Deep-branching breviate *Pygsuia* glides on its posterior cilium, but *Breviata* (without dynein inner arms) and *Subulatomonas* (sisters by rDNA trees) lost the posterior cilium and glide with their anterior one; their posterior centrioles are short. All three are pyriform, retaining this shape despite lacking the thick pellicle dorsal layer of apusomonads, though *Subulatomonas* apparently has a very thin one; centrioles are at the narrow tip of the cell and nucleus nearby. *Pygsuia* centrioles are rather long, the ciliated one of *Breviata* somewhat shorter; mature ones lack cartwheels but procentrioles in *Breviata* have them. TZ is type I with TP in both genera close to the plasma membrane a little distal to TFs; *Breviata* cp abuts TP and *Pygsuia* flat cp axosome is very close to TP. There is no obvious distal TH/cylinder but *Breviata* shows hints of transitional structures (not standard spokes) between doublets and cp in the TS immediately above TP. *Breviata* has a short proximal TZ 'cylinder' inside the doublets (Heiss et al. 2013b Fig. 6A), which in TS (their Fig. 4D) is nonagonal; its TP in Heiss et al. (2013b Fig. 3F) is indistinguishable from that of *Thecamonas* in Heiss et al. (2013a Fig. 4C) both are largely amorphous with hints of peripheral star-like densities. *Thecamonas* has an unusual combination of TZ structures: a nonagonal or 18-gonal fibre/tube distal to TP (Fig. 19R,S) and a more amorphous TH-like zig-zag walled structure proximal to TP (Fig 19M,O)

TZ structure of both apusozoan groups (breviates, apusomomads) may be more similar than they currently appear: a nonagonal or 18-gonal tube appears predominantly proximal to the TP in breviates and distal in apusomonads, but each has more amorphous structures in the other position. High resolution studies of a diversity of apusozoan lineages would be valuable to test whether they have basically homologous structures differing sightly in relative position to TP and degree of clarity and to establish more firmly the ancestral TZ condition before the origin of the opisthokont structures. The previous section showed that sponges and fungi both have distal TH-like structures, whereas in choanoflagellates there is a mainly proximal TH-like structure that in some without an obscuring dense plug extends slightly distally to TP as in Apusozoa, the ancestors of opisthokonts. On present evidence it appears therefore that the ancestral apusozoan condition of a largely proximal nonagonal tube may have been inherited by choanoflagellates and that animals and fungi may have independently extended it distally to TP and reduced it proximally. By contrast choanoflagellates apparently extended the TZ distally by (a) moving the TP distally (thus evolving type II TZ) and (b) evolving the central filament to move the cp base even further from the cell surface.

Most Amoebozoa lost cilia after pseudopodial locomotion evolved, but the main subclades of infraphylum Conosa (Mycetozoa/Archamoebae; Variosea) kept them. Myxogastrid and exosporean Mycetozoa (e.g., *Physarum, Protosporangium*) and a few Variosea (e.g., *Ceratiomyxella*) retained the posterior cilium but unlike in Apusozoa it is not used for gliding and was lost by infraphylum Archamoebae and most Variosea which evolved a more cone-like microtubular cytoskeleton, probably from the dorsal fan of mts present at the cell apex of ancestral crown eukaryotes. Like *Breviata*, archamoebae lack outer dynein arms. I often saw small, uncultured soil putative *Mastigella* (Archamoebae) without obvious pseudopods gliding on their anterior straight cilium; Zadrobilková et al. (2015) also contrast gliding and crawling in *M. eilhardi*, *erinacea*, *ineffigiata*, and *rubiformis* without making clear its motive force. *Tricholimax* and most *Pelomyxa* have immotile pseudocilia without dynein arms or cp mts and aberrant axoneme patterns. *Pelomyxa* evolved from *Mastigella* by ciliary multiplication (Cavalier-Smith 1991a, 1991b, 1991c; Zadrobilková et al. 2015): *P. palustris* axonemes vary in different strains (likely different species as other nominal *P. palustris* strains differ genetically—one has nine doublets with A-tubule feet rather than spokes plus a central mt without projections so may represent an extended TZ; another has 8 doublets plus two peripheral and one central singlet and an irregular concentric ring of granules that might represent a loose TH or spoke heads; the third has about six doublets, 2-4 triplets and about two peripheral and 3-4 central singlets plus irregular densities (Griffin 1988 Figs. 13, 12 and 14-15 respectively). *Pelomyxa binucleata* is the only known species with motile cilia—normal simple undulation in young uninucleate cells, odder forms in older binucleate ones; its cp base is surounded by a basal cylinder (Frolov et al. 2005), likely the ancestral condition for *Pelomyxa* TZ. Other species lack cilia (*P. corona*: Frolov et al. 2004) or are immotile with aberrant axonemes. *P. stagnalis* has 9+2, 9+0, 9+1, 10+2 axonemes without distinctive TZ (Chistyakova and Frolov 2011). *P. flava* has nine doublets and irregular central material (Frolov et al. 2011 Fig. 4b) which in LS (their Fig. 4A) resembles an extended multigyred TH. *P. schiedti* (related to *Mastigella* on actin trees: Zadrobilková et al. 2015) has a central mt (or filament? unclear which) and 9 doublets probably with A-tubule feet not spokes, but TZ unclear (Zadrobilková et al. 2015). *P. gruberi* has a TZ basal cylinder and axonemes vary in pattern even on different cilia of the same cell; one had nine doublets, but one was in the centre with the solitary central mt. Thus *Pelomyxa* pseudocilia are multiform and most cannot be interpreted simply as hypertrophied TZs as in other protist pseudocilia. *Mastigella rubiformis* (Archamoebea) probably has a long TH surrounding cp (Zadrobilková et al. 2015) essentially indistinguishable from that of Chytridiomycetes (Fig. 19T) and also an outer dense cylinder in its similar length Y-link zone. But no micrographs of pelomxid TZ are at all clear. Clearer but previously misinterpreted, *Mastigella commutans* is the only member of Pelomyxidae where the TP position is clear—just above the TFs—but was misidentified as a 'transitional cylinder' (Walker et al. 2001 Fig. 7b.g). Immediately distal to TP is a slender ring attached to doublets by A-tubule-feet (their Fig. 7f) below the start of cp, which unusually are not attached to the TP, these feet extending distally for a substantial distance past the start of cp but without an associated cylinder (their Fig. 7b, e). Logically their Fig. 7e TS should include the acorn structure also but it is dense and has too much superimposition to see it. Appearance of the so-called transition cylinder in LS (their Fig. 7b) is consistent with it being a composite of the TP immediately overlying the acorn.

Mastigamoebidae TZs have two or three different TZ patterns. Most distinctive are *Mastigamoeba schizophrenia* and *punctachora* with a long dense plug (earlier called the dense cylinder or column: Simpson et al. 1997; Walker et al. 2001) extending proximally from just below the cp. In *M. punctachora* the plug's distal end does not extend fully across the intra-doublet lumen and the cp and part of the TP are seen asymmetrically beside it, showing that the plug is proximal to the TP, likely true also in *P. schizophrenia* though no section included this zone. Unusually, the *P. schizophrenia* centriole has doublets not triplets; nonetheless Simpson et al. (1997) correctly identified a thin-walled cylinder (actually nonagonal) level with the TFs as distal centriolar not TZ. However the diagrams in Walker et al. of these and two other species misrepresented it as the same structure as the TZ TP/acorn complex of *M. commutans* under the common name 'transition cylinder' (TC). The triplet centriole of *M. punctochora* has a nonagonal tube (NT) over most of its distal length (their Fig. 5hj), which is presumably homologous with the NT, but both are a different structure from those labelled TC in their figs (Fig. 5a, g, h) at the level of the TFs, which almost certainly represent extra dense material associated with the acorn complex. In fact there is a prominent lumenal acorn filament at the expected level of the TFs in the TS in their Fig. 5g. This means that in comparison with *Mastigella commutans* whose TZ is short because TP immediately abuts the acorn, that of these two mastigamoebae is very long, partly because of the plug inserted between the TP and acorn. However, both species have a cp-free zone between the plug and acorn (short in *M. schizophrenia*, almost as long as the plug in *M. punctachora*. In this zone doublets have A-tubule feet with unusually dense periodic staining, but the lumen is largely empty in *punctachora* but with medium density material in *schizophrenia*. In *punctochora* at least the A-tubule feet extend throughout the sub-TP zone both beside and below the plug. *M. punctachora* belongs to Mastigamoebidae clade A (Ptáčková et al. 2013). In both species Y-links to the membrane (conflated with TFs by the authors) extend throughout the long A-tubule foot/plug zone. By contrast, *Mastigamoeba simplex* from clade B has a short TZ without a plug (Walker et al. 2001). Like *M. commutans*, *M. simplex* has a TP acorn complex mislabelled as TC, in which one cp mt is slightly longer than the other and attached directly to TP (their Fig. 5f; a putative acorn is in TS 5g). Distal to TP, the Y-link/A-tubule-foot zone is probably short without TH or other structures linked to the A-tubule feet (their Fig. 5e); thus *simplex* is also appropriate for this ultrasimple TZ. Unidentified *Mastigamoeba* sp. of Brugerolle (1991a, 1991b) exhibits a third pattern: there is no plug but dense A-tubule feet extend about as far as in *M. schizophrenia*, as do unnoted Y-links; at least one cp mt apparently extends through the feet zone to the TP. Thus the TZ is long, so I suggest this species and *M. schizophrenia* likely belong in Mastigamoebidae A like *punctachora*. Brugerolle called the feet a spiral or helical structure, but presented no TS or other evidence for that interpretation. As they appear axially in register (not staggered) on opposite sides of the axoneme I do not consider them spiral or helical, just extra-strongly stained A-tubule feet, like the less strongly stained ones in *M. commutans* (Walker et al. 2001 Figs. 7b arrowhead, e).

*Phreatamoeba* sp. (Brugerolle 1991a Figs 4a, b) has a fourth TZ type: apparently distal to the TP is a diffuse TH supported by A-tubule feet (his Fig. 4b). Though unclear how far it and Y-links extend distally and where the cp ends, it has a long TZ but differs from the *Mastigamoeba* species in having neither a plug nor simply the feet, but a TH. If it is related to *Phreatamoeba balamuthi* which is in Mastigamoebidae clade A, that raises the possibility that all clade A have long TZs. I suspect that the cylinder marked by an arrow in his Fig. 4A is a distal centriolar structure (perhaps related to the clearly centriolar thick cylinder of *M. commutans*: Walker et al 2001 Fig. h-j) not TZ as he assumed. There are hints that *Phreatamoeba* (=*Mastigamoeba*) *balamuthi* (Chavez et al. 1986 Fig. 19) has a similar distal centriolar cylinder. As noted previously (Cavalier-Smith et al. 2016) it was premature to suppress the name *Phreatamoeba* and lump it with *Mastigamoeba*, as we do not know whether the type species of *Mastigamoeba* is in clade A or B or neither! We shall eventually need at least two ciliated genera in Mastigamoebidae given their genetic and TZ diversity. Micrographs of *Rhizomastix* TZ are too poor to fully support previous interpretations (Ptáčková et al. 2013; Zadrobilková et al. 2016). Given that no uniform TC exists in the four species diagrammed by Walker et al. 2001) it is unhelpful to say that Zadrobilková et al., 2015 Fig. 9B shows a 'transition zone cylinder ... similar to that seen previously in *Mastigamoeba* and *Mastigella* (Walker et al. 2001)'. That LS is so fuzzy and unclear that the TZ parts of their Fig. 12 diagram are largely guesswork. None of these archamoeba papers clearly identified the position of the TP (though I have now for some), which remains a mystery for both studied *Rhizomastix*. Their Fig. 9B is reminiscent of many pictures of fungal TZ plugs, so it might have a short TZ plug similar to them (i.e., unlike other archamoebae); the periodic dense periphery of this dense zone is also similar to the periodic densities of the outer parts of *Mastigamoeba* sp. Y-links (Brugerolle 1991a Fig.5A), probably also in *Mastigella rubiformis* (Fig. 19T) and to the outer cylinder of *Viridiraptor* (Hess and Melkonian 2014); as this LS is tangential it may simply represent dense material associated with both Y-links and A-tubule feet and need not have a definite cylinder or inner plug (but might have either—most likely a cylinder as apparently in *M. rubiformis*). Their problematic Fig. 9A of *R. elongata* is said to show a TZ spiral like that of *Mastigamoeba* sp. (which I argue is not a spiral but dense A-tubule feet). I think the periodic dense blobs labelled by arrows probably are indeed dense A-tubule feet like those of *Mastigamoeba* sp. If so they suggest that *Rhizomastix* has a long TZ in contradiction to Fig. 9B suggesting a shorter one. The slanting diagonal lines that extend over the whole cilium even outside the doublets appear to be an artefact (sectioning?) being absent from the other five LSs. We do not know where *M. commutans* is on the tree (the GenBank entry of that name is thought to be cross contamination from *M. punctachora*: Ptáčková et al. 2013). TZ structure is uncertain in *Mastigella rubiformis* which more closely related *Rhizomastix* on sequence trees than to Mastigamoebidae; it appears to have a medium length TZ with rather dense Y-link zone consistent with my interpretation of *Rhizomastix*, and perhaps a basal cylinder (Fig. 19T).

Variosean subclade Holomastigida (*Multicilia*, *Artodiscus*) multiplied its unikont cilia—now that the close relationship of *Artodiscus* to *Multicilia* is confirmed (Ntakou et al. 2019) we need not retain order Artodiscida (Cavalier-Smith 2013) so I here formally transfer Artodiscidae to order Holomastigida alongside Multiciliidae, thereby reducing the number of orders in class Variosea (Cavalier-Smith et al. 2016) to five. Within Variosea in the light of multiprotein phylogeny (Kang et al. 2017) I also transfer Filamoebidae from Varipodida to Protostelida, and Schizoplasmodiidae from Phalansterida to Protostelida; their earlier positions (Cavalier-Smith et al. 2016) were based on insufficiently resolved rDNA trees. Aerobic *Phalansterium*, representing the earliest diverging branch in Variosea (Kang et al. 2017) probably lost its posterior cilium and centriole early to become truly unikont, convergently with Archamoebae; two have a clear TZ acorn.

*Phalansterium* unusually has a large cytoplasmic collar around its cilium (Cavalier-Smith et al. 2004; Ekelund 2002; Hibberd 1983) which is not subdivided into filodigits (microvilli) as in choanoflagellates (Fig. 19L). In freshwater *P. digitatum* the proximal 15 μm of the cilium is thicker, stiff and immotile, and surrounded basally by the 4-6 μm long collar within which axoneme structure is entirely TZ with Y-links and no doublet arms or spokes. This exceedingly long TZ is far longer than that of *Calkinsia* discussed above and likewise axially differentiated in an evolutionarily illuminating manner; distally only one cp mt enters the collar zone (Fig. 19H, L) probably true of all *Phalansterium* though information on soil species is scrappy (Fig. 19F, G); more proximally this mt is connected by a central filament (Fig. 19I) to a near-basal tapering plug (Fig. 19A, J), which I consider an axially hypertrophied axosomal plate and/or distal hub spoke structure. Below the plug is a discontinuous 'transitional cylinder', really a stack of discrete short nonagonal tubes (NT) connected by short radial linkers to the A-tubule feet (Fig. 19A, K). In *P. digitatum* NT is about 100 nm long and begins a little distal to a very thin and weakly stained putative TP (Fig. 19A); present also in soil *P. arcticum* (Fig. 19B). At the TZ base immediately distal to triplet ends is the acorn-homologue in both species (Fig. 19A-D). *P. digitatum* acorn is connected to the tenuous TP by an eccentric tilted linker ~25 nm long (Fig. 19A), similar to that of *Chlamydomonas*. Unicellular soil *Phalansterium* feed by catching bacteria on the stiffly beating cilium, moving them down to its base by ciliary surface motility (presumably by the same surface motility machinery Breviatidae use to glide) and ingest them inside the collar base (Smirnov et al. 2011; Shmakova et al. 2018). They lose the collar when transforming into swimming flagellates or aciliate amoebae. Ribosomes but not mitochondria are in the proximal collar cytoplasm; distally it resembles a pure actin gel like a pseudopod.

Acorn-like filaments at the triplet/doublet junction are clearer in *Phalansterium digitatum* and *arcticum* (Hibberd 1983 Figs. 10,13; Shmakova et al. 2018 Fig. 4C) than in most other dorsate eukaryotes (see Fig. 19A, C, D) but in *P. arcticum* were misidentified as 'concentric rings, or as a transition cylinder' (Shmakova et al. 2018). In both species an extra beaded filament absent in *Chlamydomonas* connects the granule on the lumenal filament opposite doublet 6 to the A-tubule foot of triplet 5. I was unable to find a well contrasted acorn in other Conosa to cheque the generality of this extra filament, but low-contrast Fig. 5.1 section 3 of Wright et al. (1979) suggests myxogastrids have an acorn too. Above the *P. digitatum* plug (not clearly homologous with opisthokont plugs, but possibly so) the long distal TZ is greatly swollen, with nexin-like links between the doublets and Y-links but nothing obvious in the lumen except the slender central filament which may reasonably be supposed to link cp to the plug analogously to the central filament of choanoflagellates.

In sum, *Breviata*, *Thecamomas*, and *Phalansterium* have short TZ nonagonal tubes likely distal to TP in *Phalansterium* (unless the plug were really a TP-homologue) and *Thecamonas*, but at least largely proximal to it in *Breviata*; they lack a clearly identifiable TH but the *Phalansterium* plug might be related. Apusomonads have TZ lattice structures suggestive of a TH but more amorphous and positionally different (below TP), whereas archamoebae are distinctly variable, some having a very short TZ without special structures and others have a long zone distal to TP with or without a TH or circular fibre or proximal to TP with or without a dense plug. The detailed section on fungi below shows that in fungi also one can identify either NTs or TH in different species, but not both in the same species. I will argue that this probably means that the NT is a general structure but so slender and inconspicuous that it is visible only when the more extensive dense staining TH associated structures are removed by secondary loss and suggest that this differential visibility of potentially coexisting structures is probably true of both opisthokonts and their diacentrid ancestors and thus a feature of their joint clade, which I call torcids to signify my conclusion that the whole clade ancestrally probably had a non-microvillar collar surrounding its anterior cilium (torc is English for a metal collar—worn round the neck by richer Anglo-Saxons in prehistory). On the whole, diacentrids do not have densely staining plugs that obscure fundamental TZ architecture, but are seen in some Conosa, e.g., protostelid varioseans *Ceratomyxiella tahitiensis* and *Planoprotostelium aurantium* (Spiegel 1981 Figs. 1, 3); for the latter the position of TP is unknown so it is unclear whether their plugs are proximal as in *Mastigamoeba* or distal as in fungi. If protostelid dense plugs are hiding THs as in opisthokonts, it would suggest that a TH was present in ancestral Conosa, but was lost by Mycetozoa, which apparently have neither a TH nor a dense plug (Karpov et al., 2003a, b).

The fundamentally similar TZ structure in opisthokonts and diacentrids probably goes back at least to the base of the dorsate clade if the basal cylinder/TH of *Ancyromonas* is homologous with that of opisthokonts and diacentrids, which there is no compelling reason to question. As a rather similar basal cylinder is present in Diphylleida, for the first time I have shown its presence in *all* major clades of ciliated dorsates on Fig. 11 except Ichthyosporea and Filasterea (only one micrograph clearly showing a 9+2 cilium: Torruella et al. 2015), both almost devoid of relevant data. Therefore a TH pervades dorsate TZs, but is not found in basal natates (protozoan subkingdom Natozoa) yet more widespread than previously realised in Corticata.

The greater ciliary similarity than previously recognised between *Phalansterium* and craspedid choanoflagellates, both of which seem to have a long thin central filament joing cp to the lower TZ, unlike any other dorsate eukaryotes (or any natates except Haptista) raises again the question whether the collars of choanoflagellates and *Phalansterium* could be more related and not totally convergent as Hibberd (1983) argued and Cavalier-Smith (2013) accepted until this review caused a fundamental rethink.

## Diacentrid origin of opisthokonts: cellular unity of torcids

Hibberd argued that the tubular mitochondrial cristae of *Phalansterium* made it unlikely to be closely related to choanoflagellates, which like most opisthokonts have flat cristae. That is correct, but as diacentrids essentially all have tubular cristae and include all three closest outgroups to opisthokonts it follows that opisthokonts evolved from a tubulicristate ancestor by changing cristal form. He also saw no sign of the entire collars of *Phalansterium digitatum* tending to become microvillar during culture and therefore supposed such a transformation to be unlikely. But one does not expect to see such radical changes occurring within a modern species, so not seeing them does not make it impossible to have happened once over 500 million years ago. Choanoflagellate microvillar collars had to evolve from something. Why not a *Phalansterium* type collar? The fact that two of the three immediate outgroups to opisthokonts have continuous collars around the anterior cilium (in all species in apusomonads the closest outgroup, just in *Phalansterium* in the third closest) makes it more likely than not that choanoflagellate and sponge collars evolved from the collared cells of a diacentrid flagellate by (a) losing the posterior cilium and (b) converting a continuous periciliary collar supported by an actin mesh into a discontinuous collar supported by discrete bundles of microfilaments attached to its radially pseudosymmetric mt apical skeleton to allow filter feeding for the first time. On this view the choanoflagellate cilium is homologous with the anterior cilium of dicentrids, not the posterior ones used for gliding in Apusozoa and Sulcozoa.

Previously I assumed that the opisthokont cilium (posterior during cell swimming in fungal spores, animal sperm and choanoflagellate dispersive cells) was equivalent to the older posterior cilium used for gliding by most Sulcozoa and Apusozoa. I now consider this mistaken, and think a much smoother and evolutionarily simpler transition from diacentrid to ancestral stem choanoflagellate-like opisthokont would have occurred if the anterior cilium and its roots were retained and the gliding posterior cilium and its roots were lost, as clearly happened within Amoebozoa in ancestral Archamoebea, and in *Phalansterium* and all other distinct uniciliate clades of Variosea. In the ancestor the anterior ciliumum beat asymmetrically so pulled the cell forward; during the origin of the microvilli for filtering its beat switched to a base-to-tip symmetric undulation, which pushed the cell forward when swimming (thus making the originally anterior cilium point posteriorly, thus physiologically opisthokont) or pulled water through the microvilli when feeding. In other words, the opisthokont cilium is the younger anterior cilium and its barren centriole (when present) all that is left of the older apusozoan posterior gliding cilium. It would be mechanistically easier to evolve the apical mt fan from the dorsal fan of Apusozoa or *Malawimonas* than from the much more complex and geometrically very different posterior roots as earlier postulated (Cavalier-Smith 2013). But it would be even easier to evolve them from the subcollar mt fan of a *Phalansterium*-like flagellate than from the dorsal fan of an apusomonad or breviate. I therefore suggest that the last common ancestor of all diacentrids and the last common ancestor of apusozoa had both a collar (like *Phalansterium*) and a posterior cilium that it used for gliding on surfaces, and that the immediate ancestor of opisthokonts was such a generalised, collared apusozon cell, less specialised than either breviates or apusomonads (or any specific amoebozoan lineage).

**Fig. 20.**
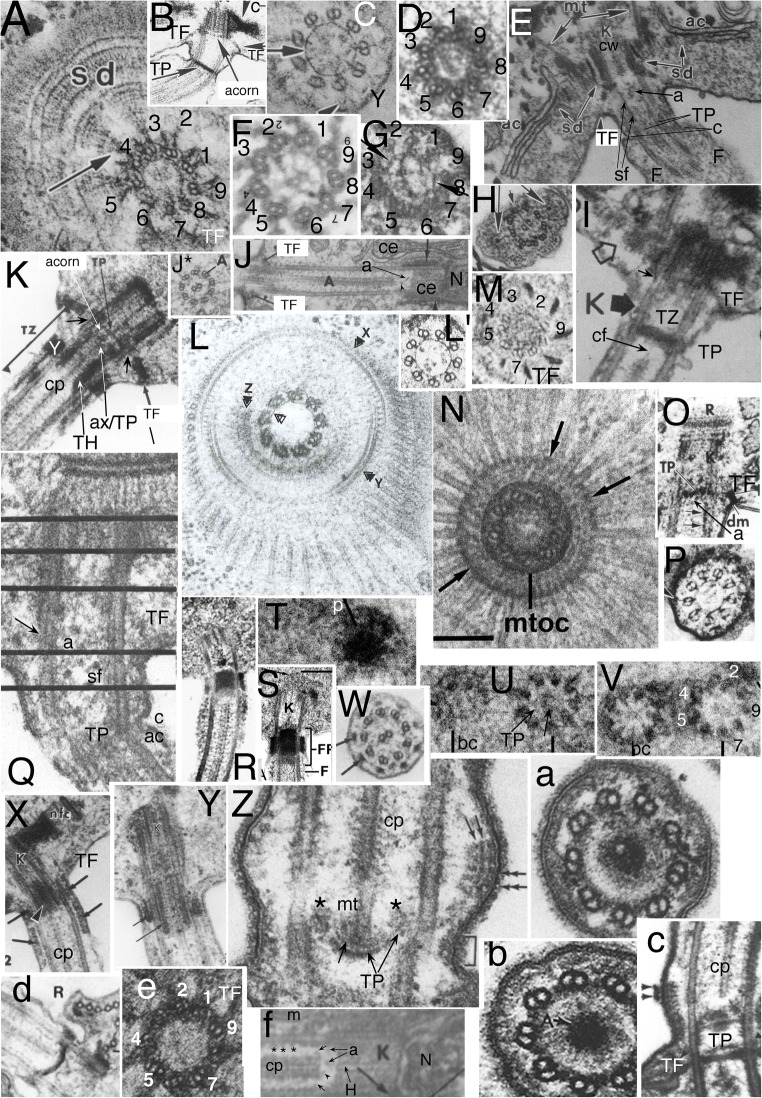
Fungal transition zones. **A-C.*** Monoblepharis polymorpha* (Chytridiomycota: Parachytriomycetes). **A.** TS of TZ/centriole junction showing central acorn complex (doublets numbered after Geimer and Melkonian 2004) and the striated disc (**sd**) of radiating mts linked by circumferential filaments attached (arrow) distally to triplets. **B.** LS showing **TP** at constriction well separated from acorn complex at TZ base (level with TF bases); **c** centriolar connector. **C.*** Monoblepharis polymorpha* spiral fibre (arrow) in proximal TZ; **Y** Y-links. **D.*** Codonosiga* (=*Codosiga*) *botrytis* (Choanoflagellatea). Distal TS of non-ciliate centriole with putative acorn complex (triplets numbered after Geimer and Melkonian 2004, often with faint C tubules implying they end within this section) from Hibberd (1975 fig 29) by permission. **E.*** Gonapodya polymorpha* (Parachytriomycetes); LS showing annular cisternae linked to striated disc, putative acorn complex (**a**) and spiral fibre (**sf**) proximal to **TP; F** lateral flanges. **F.*** Chlamydomonas reinhardtii* (Chlorophyta)acorn-V complex proximal to filaments with acorn shape from Geimer and Melkonian (2005) by permission. **G.*** Phalansterium arcticum* (Amoebozoa) from Shmakova et al. (2018) by permission. **H.*** Gonapodya polymorpha* LS showing central filament (arrows) supporting ciliary flanges*.***I.*** Harpochytrium hedynii* (Parachytriomycetes: Monoblepharidales) from Travland and Whisler (1971 fig. 2) by permission; **K** TZ proximal to **TP;** arrow marks top of centriole; **cf** central filament linking cp to **TP**; open arrow striated disc. **J.*** Coelomomyces punctatus* (Allomycetes: Blastocladiales) from Martin (1971 fig. 11); arrow dense sleeve around centriole (**ce**); centriole attached basally to nucleus (N) nestles in indentation of mitochondrion m); central pair mts (**A)** with projections extend greatly below TFs; a putative acorn lumenal filament; arrowhead putative link between cp and acorn. J*.* Coelomomyces* intractyoplasmic axoneme (**A**) doublets with arms and spokes and cp. **K.*** Catenochytridium hemicysti* (Chytridiomycetes: Cladochytriales) from Barr et al. (1987 fig. 24). Type Ia **TZ** with TP/axosome (**ax/TP**) close to acorn) and distal **TH** with central pair (**cp**) within it; **Y** dense Y-link zone extended as distal sleeve around doublets; if arrows mark ends of C-tubules old TP label marks a distal centriolar plate. **L.*** Phalansterium digitatum* (Amoebozoa) from Hibberd (1983 Fig. 12) by permission; concentric rings X, Y, Z link pericentriolar fan mts. **L'*** Olpidium brassicae* slender ring linked to A-tubule feet proximal to TP; from Lange and Olson (1976 fig. 14)*.***M.*** Caulochytrium protostelioides* (Chytridiomycetes) TS through dense distal part of TFs including acorn-V and probably also upper part of underlying centriolar (not TZ) 'terminal plate'; from Powell (1981 fig. 13) by permission. **N.*** Monosiga ovata* (Choanoflagellatea) TS of proximal end of anterior ciliated centriole with radiating mts; arrows show circumferential linkers; from Karpov (2016 fig. 5a) by permission. **O. P.*** Caulochytrium protostelioides* (Chytridiomycetes) from Powell (1981 figs 11, 14) by permission. **O.** LS of centriole and proximal type II TZ showing spiral fibre (arrows) and acorn-complex overlying dense centriolar 'terminal' plate ('TP'; the more distal TZ TP is not shown). **P.** TS of proximal TZ with spiral fibre and dense A-tubule feet. **Q.*** Olpidium brassicae* ciliated centriole LS from Lange and Olson (1976 fig. 1) by permission; **sf** level of spiral fibre/circular filament in **L'; a** level of acorn TS in Fig. 15Q; arrow distal end of C tubule. **R, S, U-V.*** Polyphlyctis willoughbyi* (Chytridiomycetes) from Letcher and Powell (2018 figs 5A, L-N, 6A). **R, S.** Type Ib TZ; LSs through ciliary plug (**FP**) and centriole (**K**). **T-V.** consecutive sections through TZ base (right) and barren centriole (left). **T.** base of plug (**p**). **U.** right centriole **TP** with dense central zone (arrow) and top of barren centriole (**bc**). **V.** bc (cartwheel zone); right centriole with acorn lumenal filament between doublets 2, 7. **W**, **X.*** Allochytridium luteum* (Chytridiomycetes) from Barr and Désaulniers (1987 figs 22, 23) by permission. **W.** arrows mark rods supporting sleeve around doublets distal to TZ. **X.** arrows mark basal sleeve around doublets, arrowhead TH. **Y.*** Allochytridium expandens* from Barr (1986 fig. 33) by permission; short arrow marks Y-link zone sleeve around doublets, long arrow basal cylinder/TH. **Z-c.*** Paramecium tetraurelia* (Alveolata: Ciliophora) from Dute and Kung (1978 figs 2, 3, 10, by permission. **Z.** TZ fixed directly in glutaraldehyde, then OsO_4_, in LS shows one mt of **cp** attached to curved thin axosomal plate (thin arrow) connected by granules to thick central zone of curved **TP**. Paired arrows mark plaque and bracket necklace zones; asterisks mark loose ring linked to doublets distal to TP. **a.** TS at level of single mt attached to axosome. **b.** TS at TP level showing its outer ring attached to doublets, dense central thickening (A), and thinner intermediate lattice. **c.** incubation in polycationic ferritin solution before fixation (to label surface anionic sites: arrowheads) breaks cp from axosome, allowing TP to flatten in LS. **d**. *Hemimastix amphikineta* (Hemimastigophora) from Foissner and Foissner (1993 fig. 58) by permission. **e.** Putative acorn lumenal filament in Rhesus monkey oviduct centriole from Anderson (1972 fig. 1c) by permission. **f.*** Coelomomyces punctatus* (Allomycetes: Blastocladiales) from Martin (1971 fig. 12) by permission; zoospore centriole (**K**) attached basally to nucleus (**N**) and laterally to mitochondrial envelope (**m**); cartwheel hub (**H**) extends throughout centriole to putative acorn (**a**); arrowhead marks granule at base of one cp mt that may be part of connector to acorn complex; short arrow marks possible tenuous relics of TP linking cp base to doublets; asterisks mark A-tubule feet on doublets opposite cp base

The apical mt fan of the fungus *Monoblepharis* (Figs. 18B, 20A) and the major apical fan of choanoflagellates *Codonosiga* (Fig. 18N) are orthogonal to the long axis of the cell, partially surround the ciliated centriole and have 5-6 concentric semicircular dense filaments holding the mts in a fan shape. *Phalansterium*'s apical mt fan almost completely surrounds the centriole at about 45° to the cells long axis, and also has six concentric dense filaments which are fully circular even though the fan is incomplete on one side and not fully radially symmetric. This full circularity is possible because the second centriole that must have been present in the last common ancestor of Conosa has been lost so the filaments are not prevented from circularising. Before that ancestor lost the posterior cilium the fan would probably have been more asymmetric, effectively indistinguishable from the opisthokont fan. In *Monoblepharis* retention of the second centriole seems indirectly to have constrained an earlier more asymmetric development, though *Codonosiga* created a kind of pseudosymmetry by adding four accessory foci for mt nucleation of four smaller fans. The chytridiomycete *Nowakowskiella* expanded the fan beyond a semicircle to about 280°and has three major concentric fibres over that angle. As dorsal fans of crown Apusozoa are in almost the same plane as the anterior centriole (probably antiparallel not orthogonal to it) and lack the semicircular fibres causing the banding pattern, convert them into the opisthokont pattern evolving the semicircular fibres de novo and more radically changing the root angle would involve greater changes than simply converting *Phalanterium*-like fan prior to loss of the older posterior centriole into the opisthokont pattern.

The 'basal feet' of animal ciliated centrioles and 'subdistal appendages' of their aciliate centrioles are homologous centriolar structures that radiate from the distal end of centrioles parallel to the cell surface. I suggest they are simplified homologues of the more elaborate apical fans of choanozoan and fungal opisthokonts and diacentrids as they are striated and associated with numerous apical mts radiating from the amorphous pericentriolar material. If this is correct it should be possible to use the diverse and increasingly well characterised animal pericentriolar proteins (Nigg and Holland 2018) to help characterise homologues in torcids generally and thus place simplified animal structures in evolutionary context. It should be possible to identify apical fan proteins shared by torcids (or a cladistically coherent subset of them) that are absent from more distant groups such as Sulcozoa, natates, and malawimonads. One example of a pericentriolar material protein already traced to the base of torcids (but no more distantly) is SPD-2/Cep192 which has been retained by *Dictyostelium* acentriolar centrosome (despite loss of centrioles and cilia) (Azimzadeh 2014). This provides molecular support for my thesis of torcid cell apical skeletal conservation. The fact that ODF2 protein localises both to TFs and basal feet and is essential for development of both implies that it originally served for TF assembly in the ancestral eukaryote but was recruited in the ancestral torcid for morphogenesis also of the torcid apical fan. It may be an example of an originally TZ protein secondarily recruited for centriolar appendage positioning.

I further suggest that the ancestor of opisthokonts not only had a collar and cytoskeleton like that of a pre-*Phalansterium* that still retained the posterior gliding cilium, but actually fed like a *Phalansterium* with its asymmetrically beating anterior cilium sticking out ahead of the cell, catching bacteria as it glided onwards on its posterior cilium and engulfing them inside its collar. If the cilium was much longer than the collar and beat from base to tip it would draw bacteria towards collar and cilium. Some would stick to the cilium and be moved downwards and engulfed inside the collar at its base, whereas those that hit and stuck to the outside of the collar would either bounce off or have to be engulfed by pseudopodia growing upwards from the cell body outside of the collar. Such an apusozoan ancestor could have evolved in three directions: (1) by losing the collar and fishing mode (sticking bacteria to the ciliary membrane), and reducing the subpellicular layer so it could phagocytose anywhere on its surface, thereby yielding breviates; (2) by keeping the collar but making it a narrower sleeve that excluded bacteria, and developing its ventral pseudopodia into long branches so as to use them to catch and ingest bacteria whilst stationary, retaining and emphasising its dorsal pellicle, thus making ancestral apusomonads; (3) by converting the continuous collar to a discontinuous collar of filodigits to enable filter feeding of large volumes of water whilst stationary and attached orthogonally to substrata—perfected by enhancing radial symmetry by losing the posterior gliding cilium, and using the formerly anterior cilium as a posterior pulsellum for dispersion instead of gliding. The latter would have created a stem choanoflagellate ancestor of all opisthokonts as follows.

Altering the nucleation pattern of collar actin (originally a 3D mesh with actin-like proteins 2/3 mediating branching in a *Phalansterium* type collar) to make unbranched actin bundles supporting discrete filodigits rather than a continuous collar would improve prey-carrying current flow and allow more bacteria to be caught (by filtering a volume of water not just passing it over a surface). Making the apical fan orthogonal to the centriole, not 45°, would allow collar widening by separating filodigit attachment points (their actin bundles are cross-linked to radiating fan microtubules: Karpov and Leadbeater 1998) allowing it to filter a greater volume of water; adding subsidiary fans on the other side of the centrioles probably contributed substantially to collar widening and thus filtering capacity, allowing faster growth. Thus improving filtering allowed choanoflagellates to become successful benthic and planktonic water filterers in major waters, including oceans, so the ancestral continuously collared diacentrids died out, explaining why *Phalansterium* has so little adaptive radiation compared with choanoflagellates. No *Phalansterium* inhabit the sea. Unicellular phagotrophic *Phalansterium* have been successful only in soil where the potential of large scale water filtering must be less; all species are morphologically very similar and retained an ability to feed alternatively as amoebae, which may give them a competitive advantage over choanoflagellates which also exist in soil but in low abundance. In fresh water, only multicellular *P. digitatum* from oligotrophic pools seems to have survived; neither Hibberd (1983) nor others found any evidence for phagotrophy. I conjecture it is just a saprotroph as its cilia are largely embedded in jelly so it could not feed as soil unicells do. On this theory a stem apusozoan evolved into a stem choanozoan, the first opisthokont.

I suggest this happened in fresh water and opisthokonts colonised the sea only after their primary divergence into 'holozoa' and 'holomycota'. The latter include no lineages still with filodigits or collars for predation; they may have lost collars in their common ancestor or separately in each of the three sublineages. Of these, deepest clade Cristidiscoidea ancestrally lost cilia altogether and thus use their branching filopodia, which apusozoa use just for feeding, for both feeding and locomotion, therefore almost inevitably lost filodigits with cilia. Cristidiscoidea remain largely freshwater with two sublineages (Galindo et al. 2019): order Fonticulida (Cavalier-Smith 1993d; I here formally add *Parvularia* to this order as new family Parvulariidae) with small cells is entirely from freshwater or soil; order Nucleariida (Cavalier-Smith 1993d; here formally augmented by adding *Lithocolla* and Pomphyolyxophryidae Page 1987) includes large-celled freshwater *Nuclearia*, *Pompholyxophrys* and marine and freshwater *Lithocolla*, Schulze 1874, all with a glycocalyx or perle-like cell covering, and unidentified marine and freshwater lineages. It is confusing to apply vernacular nucleariid to the whole class (Galindo et al. 2019; López-Escardó et al. 2017) as it should be kept specifically for order Nucleariida, and Discicristoidea used for the whole clade (as Torruella et al. 2015 correctly do).

Fungi and Opisthosporidia are largely and ancestrally freshwater and terrestrial (Vossbrinck and Debrunner-Vossbrinck 2005 for microsporidia) with only scattered small marine sublineages. Both ancestrally retained a large mt fan connected to the ciliated centriole that is cross striated by 5-6 semicircular filaments similarly to both choanoflagellates and *Phalansterium*, but (like choanoflagellates only) is orthogonal to the centriole. This supports my original view that fungi evolved from choanoflagellates with filodigit (microvillar) collars but lost them when they evolved cell walls and necessarily abandoned phagotrophy (Cavalier-Smith 1987b, 2001) in preference to my later idea that their immediate ancestors may not have had filodigits (Cavalier-Smith 2013). I now argue that orthogonality of fungal and rozellid mt fans is retained by developmental inertia when the former lost phagotrophy and the latter became parasites but retained vegetative nakedness and phagotrophy, and went through a functionally choanoflagellate ancestry. There was no functional reason to change their engrained morphogenetic programme (in marked contrast to the major changes during opisthokont origins), though I suggest the choanoflagellate secondary small fans were lost as unnecessary when the collar was lost. It remains more likely that chytridiomycete rhizoids evolved by extending their new walls around branching filopodia like those of apusomonads and cristidiscoids (Cavalier-Smith 2013) than around filodigits (Cavalier-Smith 2001). This further supports my continued inclusion of Cristidiscoidea in the protozoan phylum Choanozoa as their flagellate ancestors must also have had a choanoflagellate-like collar if those of fungi and opisthosporidia did so, not a more primitive *Phalansterium*-like continuous collar.

Just as holomycota probably secondarily lost collars but retained their supporting cortical mt fan, the most divergent holozoan groups (Ichthyosporea and Filasterea) also probably evolved thus from stem choanoflagellates. Ichthyosporea (to which I now formally add Corallochytrida as a third order as it is robustly sister to the other two on particularly thorough multiprotein trees using 24,021 amino acid positions (Grau-Bové et al. 2017; this is taxonomically more parsimonious than retaining two classes and inventing the unnecessary unranked clade name Teretosporea: Torruella et al. 2015) ancestrally evolved a vegetative cell wall independently of fungi and like them retained uniciliate zoospores just for dispersal, not feeding, so similarly lost collar and filodigits, but also lost the mt fan. Ichthyosporean *Dermocystidium* has a type I TZ with TP-proximal amorphous material near the doublets but no other special features (e.g., Lotman et al. 2000); it has an orthogonal barren centriole and a striated rhizoplast connecting centrioles to the nucleus like some fungi. Filasterea (endoparasitic *Capsaspora* and free-living *Ministeria* Shalchian-Tabrizi et al. 2008) lost collars but retained filodigits. *Ministeria vibrans* has a much reduced cilium (Cavalier-Smith and Chao 2003; Mylnikov et al. 2019; Torruella et al. 2015), which it uses as a vibratile stalk (Cavalier-Smith and Chao 2003) and its radiating non-collar cell body filodigits (present also in choanoflagellates) to catch bacterial prey. Neither the scale of innovations during the reductive origins of these groups and Cristidiscoidea nor their internal disparity in morphology is sufficient to merit treatment as separate phyla or removal from Choanozoa, so class rank suffices as for choanoflagellates.

A remaining question on this scenario is whether the TZ central filament was present in the last common ancestor of choanoflagellates and *Phalansterium* (the ancestral diacentrid) and lost by animals, fungi and opisthosporidia. It may have been present in the common ancestor and evolved to allow TZ lengthening because the continuous collar was originally rather narrow and long so it was best for the cilium not to undulate basally, achieved by excluding the dynein arms and spokes from an extra long TZ, and holomycota lost it when collars were lost. It may have been lost in animals when sponge embryos lost collars from their epithelia (Cavalier-Smith 2017b).

Table 2 summarises the major TZ variants for which details were given above. Any such table is oversimplified and cannot represent the full complexity of variation within such variable groups as Amoebozoa or Haptista, nor the complex and subtle homology patterns amongst related structures in a group like Halvaria where different parts of an ancestral character may be emphasised in different subgroups. It is also uncertain whether some structures given common labels are really homologous across group (notably BC and TH, as well as the boundary between these two being unclear). This table should not be read on its own but in conjunction with the text that explains complexities and caveats in detail. It ignores scattered secondary losses within a group (e.g., of TH within heterokonts), but emphasises major conserved patterns within related groups and key differences amongst them.

**Table 2:** Distribution of variable transition zone structures across eukaryote lineages

**Taxon/clade**	PROXIMAL TZ			TP	TZ type		DISTAL TZ
Malawimonada							
*Malawimonas*				**-**	IV		ax
*Gefionella*				**-**	IV		ax
Discaria							
Dorsates							
Planomonada				**+**	III		BC
Podiates							
Varisulca: Diphyllatea	H	star	sleeve	**+**	II		BC star
Torcids							
Amoebozoa				+	I (II)		NT (CF)
Obazoa							
Breviatea			NT	+	I		
Apusomonadida		az		**+**	I		NT*
Opisthokonts							
Opisthosporidia				+	I?II		(?TH)
FUNGI			(NT)	+	I /II		(TH/BC) (SF)
Holozoa							
Ichthyosporea				+	I		
Filasterea				+	?		
Choanoflagellatea		az		+	II		(CF)
ANIMALIA							
Porifera				+	I		TH/BC
Others				+	II		TH/BC
Natates							
Metamonada				+	I		
Photaria							
Eozoa							
Eolouka: Jakobea				+	I		ax
Discicristata							
Percolozoa				+	I		
Euglenozoa			(HS)	+	II		(HS) BC
Eucorta							
Hemimastigophora				+	I		H BC/NT
Corticata							
CHROMISTA							
Harosa							
Halvaria							
Heterokonta			H	+	I		TH
Alveolata							
Ciliophora				+	I		ax (loose TH)
Miozoa							
Protalveolata				+	I		BC/sleeve/TH
Myzozoa				+	I		BC
Rhizaria		H		+	I (II)		HS
Telonemia				+	I		ax
Hacrobia							
Haptista			NT	+	II		CF (BC dp)
Cryptista			NT	+	II		ax (TH)
PLANTAE							
Biliphyta							
Glaucophyta				+	I		NT das TH
Rhodaria				+	I	H	NT das TH
Viridiplantae		BC star		+	II		BC star (TH)

ax axosomal plate. az amophous/zigzag material. BC basal cylinder. CF central filament. das distal annular septum. dp distal plate. H hub. HS hub-spoke structure. NT nonagonal tube (those with * can also appear 18-gonal). SF spiral filament. Structures in brackets are only found in one or a few members of a group

All eukaryote TZs have A-tubule feet, A-B links, and TFs, though malawimonads probably have just one or two (not nine) TFs; Y-links are present in all except *Giardia* and Sporozoa (lost independently). Probably all have proximal peripheral acorn filaments, and all discaria also lumenal acorn filaments and perhaps some kind of V-filament

## Early podiate origin of the torcid continuous collar

In planomonads, the most divergent dorsates, the anterior cilium is in a deep pocket, not at the cell apex. Nonetheless, because the pocket's pellicle is lined by a submembrane protein layer potentially able to support a rigid shape, it might become able to grow outwards to form a collar around the anterior cilium only. I suggest that probably first happened in a stem podiate when ventral branching pseudopods (emanating from the ventral groove) first evolved, i.e., before torcids diverged from their varisulcan sisters.

In favour of this is the ventral 'apertural rim' of the aciliate filopodial varisulcan *Rigifila ramosa* (Yabuki et al. 2013b), which is morphologically a radially symmetric collar similar in thickess to those of soil *Phalansterium*, and might better be called a collar as it protrudes about 1.6 μm from the ventral surface and surrounds the filopodial base like a collar, instead of the cilium as in *Phalansterium*. In a 351-protein tree (Brown et al. 2018) *Rigifila* is sister to Diphylleida, and this freshwater clade sister to the marine flagellate *Mantamonas* that glides on its posterior cilium whilst its also non-undulating anterior cilium sticks out straight ahead of the cell stiffly vibrating (Glücksman et al. 2011). Given that planomonads are all posterior ciliary gliders (Cavalier-Smith et al. 2008b; Glücksman et al. 2013), as ancestrally were torcids, it follows that the ancestral varisulcan also was a posterior ciliary glider. Unfortunately *Mantamona*s has been refractory to good EM fixation, so we still lack ultrastructure for the only known gliding varisulcan, which hampers reconstruction of their ancestral state. It would be especially interesting to know its kinetid organisation and what kind of pellicle it has as diphylleids have a restricted, probably evolutionarily reduced single-layer pellicle whose margins tend to inroll, somewhat like the more robust 2-layer apusomonad pellicle. By contrast *Rigifila* has a thinner 2-layer pellicle that extends over the flared rim of its collar.

However, I suggest the ancestal varisulcan (last common ancestor of *Rigifila* and *Mantomonas*) was a gliding flagellate similar to *Mantamonas* but with much longer anterior cilium surrounded at its base by a collar similar to that of *Rigifila*, and was able to emit *Rigifila*-like ventral pseudopods when not gliding. Whether pseudopods came only from the ventral groove outside the collar as in apusomonds, or from inside the collar also as in *Phalansterium* is less important than the idea that it had all four of anterior ciliary collar, ventral groove, ventral pseudopodia, and posterior gliding. By adaptive radiation it could have generated other Varisulca by differential losses: *Mantamonas* by losing the collar; diphylleids by losing gliding and collar; *Rigifila* by losing both cilia and the ventral groove, retaining the collar and directing filopodia through it whilst radially symmetrising the whole pellicle; and torcids by keeping all four characters and evolving anterior ciliary bacterial entrapment (if it did not already do that). A stem podiate with these characters could have generated Amoebozoa also simply by evolving pseudopodial amoeboid locomotion and losing posterior ciliary gliding, followed later by numerous independent losses of cilia or just of the posterior cilium.

At some stage the orthogonal sulcozoan centrioles must have become more divergent and antiparallel. The dorsal mt fan as in malawimonad ancestors was apparently retained by apusozoa (called fan in breviates, ribbon in apusomonads) and became much broader in conosan Amoebozoa and more cone-like in the secondary unikonts, e.g., *Phalansterium*, Holomastigida, Archamoebea. Unless concentric filaments causing fan striation are convergent between opisthokonts and *Phalansterium*, which seems implausible, they must have been present in the torcid ancestor and have been lost in Conosa other than *Phalansterium* whenever the collar was lost, at which times the orientation of the former dorsal fan reverted to a more longitudinal quasi-ancestral state. Ultrastructure of *Mantamonas* is especially important to seek dorsal fan homologues [none clear in diphylleids, which may have lost them and the X microtubule band of *Ancyromonas*, though a likely fan derivative—Heiss et al. 2011 has lost close association with the centriole] and possible precursors of these concentric filaments. In absence of such evidence, one possibility is that proteins of curved double strips X, Y, Z (Fig. 20L) might be related to and derived from the curved double pellicular layers of *Rigifila*, which eventually could be tested proteomically. During the origin of Amoebozoa pseudopodial locomotion ancestral sulcozoan pellicular layers must have been lost, but relatives could have been recruited beforehand for making a circular filamentous skeleton for the pericollar fan.

On this view of dorsate evolution a ciliary collar is virtually as ancient as pseudopodia and differential losses of major morphological characters coupled with adaptive modifications of feeding habits (Cavalier-Smith 2013) was as important in producing their cell structural diversity, as were differential losses of protein genes during the molecular evolution of opisthokonts (Torruella et al. 2015). In contrast there was no tendency to evolve a ciliary collar in natate eukaryotes. Instead those that entrap prey by cell projections nearly all use axopodia with mts not actin as their skeleton (for multiple origins of axopodia see Cavalier-Smith and Chao 2012 and Cavalier-Smith et al. 2018). It would not have been possible to reach such a precise explanation of opisthokont origin without taxonomically rich multiprotein trees securely establishing phylogenetic relationships as in Fig. 11, discovery of ecologically minor but phylogenetically crucial genera (e.g., *Rigifila*, *Mantamonas*, *Gefionella*, *Parvularia*, *Pygsuia*, *Ministeria*), good ultrastructure for all major lineages except *Mantamonas*, and rooting arguments put forward in this synthesis.

## Fungal transition zones

Fungal TZs should interest not only mycologists but also cilia specialists as examples of the extreme specialization possible in cells (zoospores) that make cilia for terminally differentiating cells that (like animal sperm) do not divide until after cilia developmentally regress. The allomycete *Coelomomyces punctatus* parasitic on the malaria mosquito *Aedes quadrimaculatus* is the only eukaryote where I found a reasonably clear case of complete secondary loss of TP and Y-links, yet TFs, A-tubule feet and (probably) the acorn are retained, implying they are the most fundamental parts of the TZ.

Above I compared polychytrid chytridiomycete cilia especially with other opisthokonts to demonstrate the fundamental similarity of TZ across all major opisthokont groups despite some variation within each. As fungal TZs are extremely diverse, I conclude my phylogenetic survey by showing that all are probably variants of that basic pattern. I follow the fungal higher classification of Cavalier-Smith (2013 Table 8) except that I exclude Microsporidia as being protozoan Opisthosporidia and now rank Chytridiomycota (3 ciliated classes: Parachytriomycetes, Chytridiomycetes, Allomycetes) and Zygomycota (3 classes: Zoomycetes—ancestrally uniciliate, e.g., *Olpidium*; anestrally pseudocciliate Glomomycetes, aciliate Mucoromycetes) as phyla within subkingdom Eomycota, not sub- or infraphyla as before. I recognise only four fungal phyla, two ancestrally ciliate, two not; and consider 16 (Tedersoo et al. 2018) unwarranted excess. Barren centrioles, connectors have been lost in anaerobic Neocallimastigales (gut symbionts) which have 1, 2, 4 or many cilia; Barr (2001) said they also lost props—misleading as *Caecomyces* has normal TFs (Gold et al. 1988 Fig. 13c-f) albeit less prominent than versions in aerobic fungi. In Blastocladiales the barren centriole (when present) is orthogonal to the ciliated one, possibly the ancestral fungal condition; in Spizellomycetales and Polychytriales at an acute angle (or subparallel); in Chytridiales they became secondarily parallel (Barr 2001); though depicted as parallel in Monoblepharidales (Barr 2001), as they are in *Monoblepharis* (Mollicone and Longcore 1994)—in *Harpochytrium* (Travland and Whisler 1971) they are at an acute angle.

Fungal TZ nomenclature is idiosyncratic compared with that for most protists adopted here; in particular Y-links are confusingly called transitional fibres (Mollicone and Longcore 1994; Barr 2001) or TZ fibrils (Gold et al. 1988), classical transitional fibres are called props (Lange and Olson 1978; Barr 2001), perhaps because longer and more obvious than in many eukaryotes, and the name transitional plate as used by Barr (2001) does not mean what I and most protistologists call transitional plate. He used the term 'electron dense region' to embrace what I differentiate as TP and ac, and others (e.g., Mollicone and Longcore 1994) lump these under the fungal-specific term 'flagellar plug', and the abbreviation TP or tp can be applied to different structures (Mollicone and Longcore 1994). However, no previous attempt has been made to differentiate between TP and acorn-filaments. Here I apply the same general names used above for other eukaryotes to homologous structures in fungi, which often entails using different names from original authors. I start with non-Chytridiomycetes, all of which lack dense TZ plugs, which often allows a TP to be more clearly identified. Arguments above from outgroup comparisons indicate that this absence of dense plugs in so many small outlying groups must be polyphyletic simplification of the fungal TZ.

Though I formerly included Neocallimastigales in Chytridiomycetes (Cavalier-Smith 2013), a multiprotein tree based on 29,255 amino acids maximally supports their being sisters of Monoblepharidales (Ahrendt et al. 2018), so I here group both orders in new class Parachytriomycetes. *Caecomyces* has a type I TZ with slightly longer centriole than monoblepharids and clear TP and distally directly attached cp that is surrounded by a fairly short, thin basal cylinder or nonagonal tube just above TP (Gold et al. 1988).

*Monoblepharis polymorpha* has a dense transverse plate level with the prominent ciliary constriction and serving as attachment for the cp. That is positionally equivalent to and structurally indistinguishable from what this paper calls the transition plate (TP), but Mollicone and Longcore (1994) label it 'TZ plug'. They label as terminal plate (tp) a less dense, possibly radially asymmetric 'plate' level with the TF bases (called props), which I equate with the acorn structure; they used the old confusing terminology for ciliates criticised above. Much of the 0.23 μm long zone between TP and centriole exhibits a slender ring in TS attached to the A-tubule feet (Fig. 20C) that is present in many fungi and called either a concentric ring or spiral fibre (Karpov and Fokin 1995, who note that the number of gyres is usually 8-11, rarely as few as three); I use spiral fibre as it probably better describes its established structure. This spiral fibre proximal to TP is positionally different from most fungal and dorsate THs discussed above which are distal to TP and much thicker and denser, though both structures have been conflated previously: Karpov and Fokin (1995) were confused by idiosyncratic fungal nomenclature and their Fig. 8 misleadingly depicts the position of the spiral thread as above TP (like the unrelated heterokont TH) not below TP as is certainly true of *Monoblepharis* and I think most Fungi, and also misrepresents the relative positions of cp and TP.

Figure 20A shows the doublet/triplet transition plane of *Monoblepharis* with about five doublets and four triplets as in *Chlamydomonas* and a probable acorn filament system; if numbering is correct the V arms appear on inferred triplets 3 and 4, not 4 and 5 as in natates. In choanoflagellates (Figs. 18R, 20D), the amoebozoan *Phalansterium arcticum* (Fig. 20G), and possibly the varisulcan *Collodictyon* (Fig. 13C), V arms also appear opposite 3/4 not 4/5, so I suggest many podiates may have the V opposite 3 and 4, not 4 and 5 as in natates. V densities are less obvious in the chytridiomycete *Neokarlingia* acorn, but Fig. 18E does not contradict this conclusion. *Phalansterium digitatum* is more equivocal as densities appear in all three positions (Fig. 19D) as also in the zygomycote *Olpidium* (Fig. 15Q); that zone has too much material in *Caulochytrium* (Fig. 20D) to see any simple pattern.

Other Monoblepharidales have a similar type II TZ with especially short centrioles except for *Gonapodya* (with longer ones and peculiar lateral flanges on the cilium starting just above the constriction: Fig. 20E, H) whose TP is very faint and thin (previously overlooked), and acorn-homologue has a hollow central hub with radiating filaments. *Harpochytrium hedinii* may lack a spiral fibre (Travland and Whisler 1971) and like many, if not all Chytridiomycota has much fibrillar material filling in between the Y-links making them hard to see (Fig. 20I); its TZ was wrongly thought centriolar. Centrioles of Monoblepharidales and Chytridiomycetes are ultrashort. Thus Parachytriomycetes have rather short centrioles with either a NT above TP (Neocallimastigales) or a spiral fibre below TP (Monoblepharidales), but not both, and no TH or dense plug.

Allomycetes by contrast probably have normal length or longish ciliated centrioles attached to the tip of the cone-shaped nucleus, but the position of the centriole/TZ boundary or of the true TP is not clearly established in most genera. I suspect that the so-called terminal plate level with TFs of *Blastocladiella* is the acorn-homologue as it lacks radial symmetry. Somewhat more distally *Blastocladiella* clearly has a thin nonagonal fibre with nine flat sides (Reichle and Fuller 1967 Fig. 10), but I found no evidence for a subTP spiral filament. *Cateneria* has normal length centrioles during late zoospore differentiation with an apparently symmetric thin transverse plate at the TF level, but free zoospores appear to have a longer centriole/TZ complex (Manier 1977). *Coelomomyces* has a radically peculiar zoospore with apparently no obvious TZ structures except TFs and A-tubule feet (no sign of Y-links or TP: Martin 1971). The cp seems to contact the top of the centriole almost directly and has a long (~1.25 μm) 9+2 region with standard doublet arms and spokes *inside the cytoplasm* proximal to the TFs: Fig. 20J. The centriole lumen is almost entirely filled with the cartwheel structure except for an extremely short distal zone immediately below what I suggest is an acorn-V system (but only LSs are available: Fig. 20J, b); the variable separation of cp and the putative acorn (compare Fig. 20J, b; both have an asymmetrically positioned 'granule' that might be the lumenal acorn filament in TS) is consistent with loss (or perhaps more likely extreme reduction to tenuous easily broken filaments: arrows in Fig. 20J) of the TP. The eccentric granule between one cp mt only and the putative acorn in Fig. 20J may be homologous with the centrin filament linking acorn and V in *Chlamydomonas*. If so, its likely contractility may account for the smaller separation of cp and acorn in Fig. 20f than in Fig. 20J. Surprisingly, the intracytoplasmic 9+2 axoneme is normal with cp projections and doublet spokes and arms for most of its length (Figs 20J, J*), replaced by A-tubule feet only at its extreme base (Fig. 20J*); possibly that stabilises the cp base sufficiently without needing an obvious dense TP. *Coelomyces* is the only discarian eukaryote known showing this degree of secondary TP reduction. Its C tubules star to peter out even before the top of the cartwheel, i.e., proximal to the putative acorn (Martin 1971 Fig. 7).

Thus Allomycetes and Parachytriomycetes both fit my generalisation above that in torcids, when present, NTs are typically distal to TP and spiral fibres proximal to it. Neither has a clear TH or dense plug. Zygomycetes also fit this pattern.

The zoomycete zygomycote *Olpidium* (order Olpidiales) TZ also lacks an obscuring dense plug (Lange and Olson 1976) and has a more distal TP (thus type II) whose lattice is only partly seen in Fig. 15R, but peripherally has radial filaments both connecting to A-tubule feet and out of phase with them. *Olpidium* TZ has elongated a little, separating TM and acorn more than in Polychytriales (Lange and Olsen 1976), thus shows some things more clearly. The zone between TP and centriole has a slender ring attached to the A-tubule feet (Fig. 20L'), also present in some Chytridiomycota, and usually considered a spiral or coiled or concentric fibre (Karpov and Fokin 1995), much slenderer than the heterokont TH; but some of its segments resemble a thin corticate nonagonal fibre more than a circular fibre, raising the possibility that such a fibre may even have been present in the ancestral discarian eukaryote. I suggest it might have evolved from the asymmetric acorn filament of malawimonads by axial duplication and then radial duplication of each segment. An acorn-V filament system is visible in Fig. 15Q (immediately distal to the triplets) and is much more convincing than the example from *Phlyctochytrium irregulare* (Chytridiomycetes) that Geimer and Melkonian (2004) accepted as acorn-like (Fig. 15O). As in *Chlamydomonas*, the four doublets (3-6) without a peripheral acorn filament even show a fainter C tubule, and there appears to be a somewhat irregular Y-shaped filament associated with 4, 5 and 9. This is the first clear evidence for distal extension of triplets 3-6 causing centriole distal chamfering in Fungi or any dorsates. It implies that V-filaments and this distal chamfering evolved together in the last common ancestor of discaria after it diverged from malawimonads at the same time as the TP evolved. Thus *Olpidium*, *Monoblepharis*, and *Phalansterium* together prove that a complete acorn-V system exists in dorsates as well as natates, in marked contrast to Malawimonada. However all three of these, likely also *Codonosiga*, have a filament linking doublet 3 to the lumenal acorn filament not seen in *Chlamydomonas*. Moreover in *Monoblepharis* the V-arm linking to doublet 5 is not obvious; it is as if the *Chlamydomonas* V is shifted in torcids from doublets 4, 5 to doublets 3, 4. Though its putative acorn-like complex is less clear in *Collodictyon* as Fig. 13C also includes a nonagonal fibre if numbering is correct the doublet 5 V arm seems missing and much material at 3 and 5, suggesting varisulcan acorn homologues are more like those of its torcid sisters than *Chlamydomonas*.

Now consider the highly divergent zygomycote *Amoeboradix gromovi* with an amoeboid zoospore and pseudocilium with no cp (Karpov et al. 2018). The pseudocilium has a ring of singlet microtubules only without spokes or dynein arms and its centriole is exceptionally long (1.8-2.2 μm) with doublets instead of triplets, with short cartwheel and longer non-cartwheel distal zone. It would be especially interesting to know if it has acorn filaments. Ultrashort barren centrioles (antiparallel to and basally abutting ciliated ones) have a distal dense plate. TZ structure of mature zoospores is unclear but their Fig. 4C of a sporangial long centriole (not yet grown its distal shaft) shows a TP and TFs level with the plasma membrane and the doublets starting almost immediately below it and structures that could represent an acorn system. If confirmed this would tell us that neither an acorn nor TFs intrinsically require C tubules for assembly. One section series shows the centriolar B tubule ending before the plasma membrane; though at precisely what level is unclear. This apparent contradiction might mean this series represents not a developing pseudocilium but a partially retracted one; even released zoospores can completely retract their axoneme. It is appropriate to consider the doublet-singlet transition as representing the top of the centriole; if so it may not be correct to say that its 'kinetosome consists of doublets and singlets'. A second *Amoeboradix* (strain X-44, unsequenced) had singlets throughout the pseudociliary shaft and centriole including its cartwheel base. This should be a separate species; lumping it with *A. gromovi* even temporarily was unwise. Even X-44 has a definite dense zone (similar in size to chytridiomycete plugs) at the pseudocilium base and a long 'centriole' (1.5 μm). This raises important questions of how the cell decides where to make this boundary without B and C tubules.

By rDNA operon phylogeny *Amoeboradix* is closely related to *Sanchytrium* (Karpov et al. 2018) that was originally placed in Monoblepharidales (Karpov et al. 2017) and now constitutes family Sanchytriaceae. However the new better sampled site-heterogeneous trees robustly exclude Sanchytriaceae from Monoblepharidales and Chytridiomycota and group it with moderate support with Glomomycetes. I therefore establish a new order Sanchytriales for Sanchytriaceae, which I place as a fourth order in class Glomomycetes within phylum Zygomycota.

Whether Zygomycota should be treated as a single phylum (Kirk et al. 2008; Moreau 1954; or subphylum (Cavalier-Smith 1998, 2013), or subdivided into two (Ruggiero et al. 2015; Spatafora et al. 2016), three (James et al. 2006; Naranjo-Ortiz and Gabaldón 2019) or even more (e.g., an excessive 9 in Tedersoo et al. 2018) has been controversial. Trees bases on a few genes only are often too inaccurate for resolving deep phylogeny and used uncritically cause oversplitting, which has been rife. An ML analysis using 29,255 amino acid positions from complete genomes (but of only 11 zygomycetes excluding Glomomycetes) shows only two large zygomycote clades (Ahrendt et al. 2018) as in an earlier consensus analysis of 192 individual protein trees for 25 species including a glomomycete (Spatafora et al. 2016). Together they imply that to maintain holophyly phylogeny does not require more phyla than Mucoromycota (including Glomomycetes) and Zoopagomycota. But without similar, better sampled site-heterogeneous trees we cannot rule out the possibility that these two clades might merge into one. But even if they do not (as I suspect will be the case), one could accept a paraphyletic Zygomycota if, as I do, we judge that phenotypic differences between the two apparent clades to be insufficient to merit phylum rank. I retain a conservative preference for single phylum Zygomycota Moreau 1954 divided into subphyla Mucoromycotina Benny, 2007 (two classes; Mucoromycetes Doweld, 2001, including orders Mucorales, Endogonales, and Mortierellales; Glomomycetes Cavalier-Smith 1998 em. to include Sanchytriales) and Zoopagomycotina Benny, 2007 (class Zoomycetes Cavalier-Smith 1998 with 3 subclasses: Bolomycetidae Cavalier-Smith 2013, including Olpidiales and Basidiobolales; Pedomycetidae Cavalier-Smith 2013, including Zoopagales and 5 other orders; Entomomycetidae Cavalier-Smith 2013, Entomophorales). There is no justification for making Mortierellales a subphylum or splitting Glomomycetes into three classes; those based largely on rDNA trees can better be just subclasses. Both subphyla show varying degrees of independent ciliary reductions, by shortening or losing one or both centrioles and ancillary structures.

Likewise should Chytridiomycota be one phylum or subdivided into two or more? Ciliary divergence does not justify more than one. Chytridiomycetes have very varied shortish TZ, parts often obscured by variants of the dense TZ plug, which often exaggerates their differences; almost all seem to be variants of a basic common pattern, type I with a distal TH as noted above.

One aberrant chytridiomycete, *Caulochytrium*, whose barren centriole is orthogonal or at 45° to the ciliated one (suggesting early divergence), lacks these confusing densities (Powell 1981). Unusually, its ciliated centriole is linked to the nucleus via a long striated rhizoplast (Fig. 20O). Its TZ is type II with a spiral fibre linked to wide dense A-tubule feet (Fig. 20P) extending about 0.25 μm distally to the acorn-V complex (Fig. 20M) which overlies a dense plate at the top of the centriole, and morphologically like monoblepharid proximal spiral fibres (Fig. 20O, P). The cp begins near the distal end of the spiral fibre; the true TP must be at this level as in *Monoblepharis* (Fig. 20B) but no micrograph showed the cp/TP junction. A 2-gene rDNA tree showed *Caulochytrium* as a deep isolated branch (Karpov et al. 2010) and a multiprotein tree using 29,255 amino acid positions (Ahrendt et al. 2018) firmly placed it within Chytridiomycota as sister to *Rhizoclosmatium*, so is closer to Chytridiales than Spizellomycetales [Naranjo-Ortiz and Gabaldón 2019 wrongly said its phylogeny is unstudied; Powell in Adl et al. 2019 correctly put it within Chytridomycetes (as order Caulochytriales) but Wijayawardene et al. 2018 unwisely treated it as a separate phylum].

Serial sections reveal an acorn structure (showing acorn filaments fuzzily, not resolving V) at the triplet/doublet junction of *Polyphlyctis willoughbyi* (Fig. 20V right), deep-branching within Chytridiales sensu stricto (Letcher and Powell 2018 Fig. 5N), and in the immediately distal section an also overlooked TP lattice (Fig. 20U right) that must also be in contact with the very base of the dense plug that begins in the next section (Fig. 20T). These close juxtapositions mean than neither structure will be seen unless specifically sought in serial thin sections. I suggest that in all Chytridiomycetes with a similarly long dense plug it is a distal TZ structure with TP at its very base which is also directly attached to the underlying acorn complex (as the opisthokont section argued for Polychytriales). *Polyphylyctis* differs from Polychytriales discussed above in that the cp starts distally to the plug and does not pass through its centre. A reasonable interpretation of *Polyphlyctis* is that its plug arose by serial duplication of TP (so is essentially a stack of TP lattices) and cp is nucleated at its distal-most duplicate just as in fungi with a single thin TP (e.g., *Caulochytrium*). In Polychytriales (e.g., *Polychytrium*, *Neokarlingia*, *Karlingiomyces*) and in *Catenochytrium* and *Maunachytrium*, by contrast, TP as a whole cannot have hypertrophied to make the central part of the plug; they instead evolved a TH around cp, absent in *Polyphylyctis*, so its central plug is not homologous with that of the other five genera, though the core structure of peripheral dense collar embracing the Y-links is homologous throughout (though its dense staining might be convergent in different groups). Thus there are at least two different classes of type I TZs in Chytridiomycetes: (a) those e.g., *Catenochytrium* whose cp nucleates below the plug (b) others where it nucleates distally, e.g., *Polyphlyctis*. I call them type Ia, and Ib.

*Catenochytridium*, which rRNA operon trees (James et al. 2006) put in the sister clade to Polychytriales (i.e., order Cladochytriales or suborder Cladochytriineae: Cavalier-Smith 2013), the novelty of whose TZ structure was previously overlooked (Barr et al. 1987), has a cp passing through the dense cylinder to its base where it ends at what might be a small axosome just distal to the so-called terminal plate (Fig. 20K). The distal offset of the rim of the dense basal cylinder shows it is a helix not a stack of rings. Its density decreases just below the 'axosome'; distally the matrix in its lumen is also low density, enabling radial connectors between cp and the TH to be seen easily. The Y-link zone matrix opposite the distal end of the TH is so dense that it obscures individual links, unlike Fig.18A; a dense sleeve (or possibly nine separate dense rods as in close relative *Allochytridium luteum*) outside the doublets extend distally from this Y-link zone for ~0.25 μm. The dense TH/basal cylinder of Polychytriales is closely similar to the heterokont TH and basal cylinder of Biliphyta, but unlike most other dorsates except *Planomonas*. I suggest that *Catenochytridium* 'tp' is an acorn-V and that the apparent 'axosome' is really the thicker central disc of a slender TP distally abutting the acorn, whereas the acorn (located at the base of TFs) is separated from it by a larger space (accounting for the different angle of TFs) and these centriole linkers evolved to maintain a connection with TP at the very base of the plug.

*Allochytridium luteum* also has a long centriole and a fundamentally similar type Ia TZ, but a dense plug completely fills the lumen within the TH entirely concealing it, 'axosome' and cp whose existence can only be inferred indirectly from the shape and position of the plug (Barr and Désaulniers 1987) so far into the TZ lumen that one cannot say if a spiral fibre is present; a distal NT appears absent. However the Y-link zone matrix is less dense so one can see individual links. A TS through the base of the 9+2 axoneme distal to the Y-links shows 9 discrete flattened rods between doublets and membrane alternating with slenderer rods opposite the interdoublet spaces (Fig. 20W), these collectively corresponding with the 'sleeve' of *Catenochytridium*. Apart from differences in relative positions of dense obscuring matrix (probably fundamentally relatively trivial) the main difference in its TZ is in the angle TFs make with the axoneme, less in *A. luteum*, whose dense plug has an asymmetric arrangement of linkers to the top of the centriole, one slanting one of which is quite dense like that of *Chlamydomonas* that links the acorn-V to the basal cylinder lower septum. In *Allochytridium expandens* highly structured dense doublet-associated material similar to TH is present on both sides of the putative TP (Fig. 20Y), but without serial sections and TSs identifying its exact nature is impossible, though there may be distinct dense cylinders above and below TP: calling them collectively a spiral fibre (Karpov and Fokin 1995) was unjustified: they have no obvious similarity to the spiral fibre of *Monoblepharis*.

The confusing review of fungal 'concentric ring structures' in Karpov and Fokin (1995) appears to conflate two distinct circumferential structures, here distinguished as (1) the transitional helix (distal to TP and surrounding cp) and noted only in type Ia chytridiomycete TZ; and (2) the spiral fibre proximal to TP (thus unassociated with cp) noted only in the aberrant type II chytridiomycete TZ of *Caulochytrium*, in the zygomycote *Olpidium*, and in *Monoblepharis*. As the spiral fibre is present in at least three classes of fungi, it may have been an ancestral character, and lost several times. Though one might think the presence of TH only in a subset of Chytridiomycetes implies it is derived, the TH of most choanoflagellates is so similar that it could be related and thus lost several times within fungi (the heterokont TH was lost several times within the group, so why not multiple losses in opisthokonts and in eukaryotes generally if heterokont/planomonad and opisthokont TH/basal cylinders are all related?). A third concentric structure, the distal NT positionally resembles the TH, but is morphologically more similar to the spiral fibre in its slenderness. The *Blastocladiella* nonagonal fibre (Reichle and Fuller 1967 fig. 10) might be a variant of the spiral fibre if it is a type II TZ as suggested above, or else related to *Caecomyces* NT (if TP is really proximal to it contrary to my interpretation).

*Catenochyridium* and *Allochytridium* are closely related by rDNA (James et al. 2006; Powell et al. 2018) as well as TZ and are part of a deep branching clade, currently an order Cladochytriales (Mozley-Standridge et al. 2009) or equivalent suborder (Cavalier-Smith 2013), but this name is inappropriate as the type species *Cladochytrium tenue* branches elsewhere with *Zopfochytrium* within Chytridiaceae by 28S rDNA trees (Powell et al. 2018). Using the whole rDNA operon the *Allochytridium* clade groups weakly as sister of Polychytriales, so it could be more appropriate to group it with them as a suborder not with Chytridiales. *Zopfochytrium polystomum* TZ is only marginally less obscured by the dense plug than most Chytridiales, but I can see Y-links and the distal part of a distal TH in Fig. 5I of Powell et al. (1981). Slightly proximal to the plug base and just distal to the base of TFs (i.e., at inferred acorn position in allochytrids) are lumenal densities (their Fig. 5E and I) that might be part of the acorn complex. Chytridiomycetacae (*Chytriomyces* and *Pseudorhizidium* (Letcher and Powell 2014 Fig. 4 C, D)) have a seemingly discontinuous transverse plate just above the base of TFs (interpreted here as the acorn complex), clearly separate from the plug base that should represent TP. In *Pseudorhizidium* the distal half of TH is clearly visible, less dense than the proximal plug and one can see cp at its centre (their Fig 4D; this cannot be seen in their 6C supposedly the same species ignoring the lettering error in the legend). In *Dendrochytrium*, sister to core Chytridriaceae (Letcher and Powell 2018), putative TP at the plug base and the lateral zone where TH is expected stain more strongly than the central lumen (their Fig. 6D). In *Asterophlyctis* (*Chytriomyce*s clade 2K; sister to Chytriomycetaceae: Letcher et al. 2018) Y-links are clearly visible and the central plug staining is consistent with its embracing both TH and cp. *Podochytrium dentatum* (Chytriomycetaceae clade) has strong staining in inferred TP, TH, and Y-link region but not in the main central lumen around the cp; it also has an asymmetric discontinuous plate half way between the TP position and TF bases, likely acorn-V (Longcore 1992).

It is beyond the scope of this paper to reinterpret all fungal TZs, but having shown how an acorn structure can be identified at their base and that (with the possible exception of *Coelomomyces*) all fungi have a TP either immediately overlying the acorn (short type I) or separated from it by a doublet zone typically with a spiral fibre (long type II), and that type Ia have a distal TH, but Ib do not but in some chytrids can undergo TP hypertrophy to make one type of plug, I have set out the basic structural patterns, which in many chytridiomycetes are secondarily confused by superimposition of dense matrix. If the spiral fibre is the ancestral condition, which its presence in some Opisthosporidia (fungal sisters) supports, and this fibre acts as a structural spacer generating the type II pattern one can argue that ancestrally fungi probably had a type II TZ as in Choanozoa and those chytridiomycetes with type I lost it and secondarily became type I. That is the only reasonably clear example of a likely secondary transition from type II to type I by structural loss I found in eukaryotes. The more general pattern seems to be that early diverging eukaryotes have short TZs and numerous independent lengthenings produced in different ways to suppress ciliary undulation at their base for various reasons such as ciliary inclusion in deep pits. Presence of traces of a TH in some opisthosporidia also supports my suggestion that ancestral opisthokonts had a TH and most fungal lineages except type IIa chytrids lost it. Fungi may be a special case involving multiple losses of spiral fibres and TH for TZ shortening because they lack such feeding specialisations, using their typically terminally differentiated cilia only for dispersal so retention of such TZ-lengthening features ceased to have any strong selective advantage. Their losses could also have been favoured because selection for maintaining any ciliary characters would be restricted to a brief dispersal stage, not sustained throughout the life cycle as in phagotrophic flagellates.

## Acorn filament diversity

A classical acorn-V filament as in *Chlamydomonas* is clearly present not only in the rhizarian *Viridiraptor* (Fig. 4A'', A''') but in the eustigmatophyte heterokont *Vischeria* (Santos and Leedale 1991 Fig. 6, and as noted above in the hyphochytrid heterokont *Rhizidiomyces*) as well as in the rhizarian *Katabia*. Thus it was present in the ancestral corticate. Acorns are present also in Natozoa: in several metamonads (trimastigids Fig. 14C; Parabasalia Fig. 15Z,a); discicristaes (Percolozoa Fig. 18b; Euglenozoa: *Calkinsia* Fig. 16I, *Trypanosoma* 5C, 16H,I); Jakobea Fig. 16W); and in Hemimastigophora probable acorns can be seen in less convincing oblique TS of the TF zone of *Spironema* and *Stereonema* (Foissner and Foissner 1993 Figs. 25 and 45). In most Natozoa presence of V-filaments also is unclear, mainly because of extra dense material in the 4/5 doublet zone (though not evident in *Trypanosoma* despite absence of such densities), but the arguments above for a Y-like V-system opposite doublets 4 and 5 in the acorn zone of *Pseudotrichonympha* (Fig. 15Z) are reasonably convincing (but presence of an additional filament opposite 3 cannot be excluded), whilst the percolozoan *Pleurostomum* apparently has filaments opposite doublets 3, 4, and 5 (Fig. 16b). Thus all major natate lineages apparently possess V-filaments, in marked contrast to Malawimonada which lack them.

I also found evidence for an acorn-like structure and V-filament system in all major lineages of podiates, though in none did I find clear evidence for a V specifically restricted to doublets 4-5. As summarised in the preceding section I found one fungus and one amoebozoan seemingly with radial filaments opposite doublets 3, 4, 5 and another fungus, another amoebozoan, a choanoflagellate and a sulcozoan seemingly with filaments to doublets 3 and 4 only. This seriously suggests that the V-filament pattern may differ in some or all natates from the canonical pattern in *Chlamydomonas*, which so far I have convincingly found *only* in corticates; however Fig. 20e of a putative lumenal acorn filament in a monkey centriole might be interpreted as showing also a very faint V opposite 4/5. Though caution is necessary because of possible confusion by superimposition of other structures and because even in *Chlamydomonas* the V filament-complex varies in appearance a little with level, e.g., in visibility of the V and/or the Y-like stem connecting its apex to the peripheral filament near doublet 9 or presence of an additional arc inside and parallel to the lumenal filament or a peripheral filament between 4 and 5 (Geimer and Melkonian 2004, 2005).

Even 'acorn' shape varies: distally it has a wide end in *Chlamydomonas* opposite doublets 1 and 2 the other being pointed, but more proximally the peripheral filament appear to curve more smoothly near doublet 1 so both ends appear pointed. In fact in all acorn-like images outside corticates I found none clearly with a 1-associated broader end, which always appears pointed. Thus in most non-corticates the acorn does not look like an acorn but like a segment defined by the intersection of two arcs of different diameter, thus with two pointed ends. *Phalansterium arcticum* was the only podiate found with an obviously broad-ended acorn shape, which was at the other end opposite doublets 7 and 8 (caused by a tangential filament between doublets 8 and 9 and a lumenal filament granule being denser than the end of the lumenal filament between that same granule and doublet 7. Thus changes in relative staining of components can change the perceived shape of the acorn without fundamentally altering its overall architecture. Thus the 'acorn' shape of corticates and of *P. articum* can have evolved secondarily from the apparently ancestrally double-pointed segment. In natozoa, *Pseudotrichonympha* has a broader 1/2 end like corticates but it is possible that this is caused by the base of the crescentic body not the acorn filament, as other natozoan acorn profiles without crescentic bodies appear to be double pointed.

My survey emphasises the likelihood that the V-filament system differs somewhat in natates from that of corticates, and hints that it *may* also differ a little in some or all natozoa (perhaps in the same way). Only for planomonads did I find some evidence from LS for an acorn-system sandwiched between the centriole and TP without finding any TS directly portraying the 'acorn'; thus we do not know what type of V, if any, they have. However, I predict that planomonds as the sisters of podiates will turn out to have a similar acorn-V complex; likely all dorsates have basically the same pattern. Though the axosomal density of *Gefionella* has the double-pointed segment shape it is unclear whether it or *Malawimonas* also has a discrete lumenal acorn filament; if both have only the peripheral acorn filament, the lumenal filament (other half of the acorn) evolved at the same time as the V in the ancestor of discaria.

Given a eukaryotic root between malawimonads and discaria and almost immediate divergence of dorsates and natates after TP evolved and was inserted between the acorn and cp, it is not surprising that the acorn V may differ systematically between dorsates and the derived corticate subclade of natates. It would also not be surprising if some natate lineages retain a more dorsate-like pattern, as hinted above. Unfortunately, though electron cryo-tomography has revealed centrioles and procentriole triplet and cartwheel structures in immense molecular detail in at least two natates and two dorsates (Li et al. 2019), this has not yet been done for the acorn-V in any organism. More thorough study (including tomography of phylogenetically key species) of natozoan and dorsate acorn-Vs, and of the malawimonad TZ are needed to test my inference of systematic differences between these three clades. Till then a reasonable working hypothesis would be that the V-system evolved after the acorn, and when it did so in the ancestral discarian it may have been more like a W with three arms going to doublets 3, 4, and 5, and later the 3 filament **or** the 5 filament (but not both) was secondarily lost to make two different V patterns in different lineages. Evolution often duplicates structures multiply and identically before differential specialisation and/or simplification by loss as in arthropod limbs or mouthparts, the vertebrate spine or gills or floral parts. Even presence of the acorn structure at the base of animal TZs is scarcely ever mentioned. Oddly the acorn was not explicitly even mentioned in an otherwise excellent review of the role of *Chlamydomonas* centrioles in ciliary organisation that focused mainly on IFT involvement (Wingfield and Lechtreck 2018).

## TP lattice architecture and diversity

Are there systematic differences also in the TP lattice of corticates, natozoa and dorsates? In most dorsates other than Diphylleida there is no evidence for superimposed peripheral stars associated with TPs such as occur in corticates, but good TSs of simple thin TPs are few and far between (do microscopists consider them too mundane to publish?), and can be hard even to identify with confidence or discern unambiguous substructure; the few I found often seem like simple amorphous sheets slung between the supporting doublets with a slightly denser centre, e.g., the chytridiomycete fungus *Polyphlyctis* (Fig. 20U). In the zygomycote fungus *Olpidium* the putative TP (Fig. 15S) consists of an irregular but roughly hexagonal lattice. These fungal TPs might not fundamentally differ, but simply reflect more or less staining of an amorphous matrix supported by the lattice. *Ancyromonas* (Planomonada) putative TP has an irregular lattice, easily seen in its lightly stained periphery but also just visible in its thicker denser central axosomal zone (Fig. 13T). As planomonads are maximally divergent from fungi, I suggest dorsate TPs generally consist of a simple sheet with an irregular supporting lattice that may be variably filled with dense matrix especially in its thicker central zones. Filaments of the lattice probably attach both to the A-tubule feet and to the middle granule of A-B links, so there are likely generally 18 attachment points.

In natozoan protozoa finding non-superimposed TSs of TP is also hard. In the deepest branching natates (metamonads) the putative TP is so close to the underlying acorn that a 9-fold symmetric TP is normally superimposed on the acorn within the same section. Matrix density across this complex is sometimes uneven; in the parabasalia *Pseudotrichonympha* it is greatest outside the lumenal filament and near the longer cp mt base (Fig. 15) whereas in trimastigids though TP density may be even, asymmetric dense centriolar matrix underlying the acorn gives a different type of asymmetry (Fig 14C, O). Allowing for these complications, metamonad TP substructure is not obviously greatly different from that of dorsates. Jakobids have similar superimposition problems, and sometimes fuzziness and low resolution (Fig. 15R, 16J, U-W) but the TP consists essentially of a rather amorphous sheet stretching from A-tubule feet (like a trampoline membrane radially supported from its frame) thus appearing as a very obtusely pointed star peripherally. The *Jakoba* TZ is very short but TP seems rather thick (Fig. 16J) suggesting it may directly overlie an acorn complex, but good TSs are wanting. *Reclinomonas* is basically similar; three sharper axially distinct structures clearly abut each other (Fig. 16O, P): a dense axosomal flange surrounds the cp mts, one slightly longer than the other directly contacting the centrally domed TP, which directly overlies an asymmetric putative acorn filament system; in TS the TP lattice slung from A-tubule feet and A-B links exhibits a central star-like pattern of greater density (Fig. 16X) probably corresponding with the overlying axosomal plate (less axially distinct than that of *Viridiraptor*, Fig. 17L); the C tubules with dense lumen terminate in the same section whose central density shows radial asymmetry implying that the acorn is within this section also; Fig. 16Y TS showing only A-B links and A-tubule feet with a nonagonal fibre is probably immediately below the TP/acorn complex at the very top of the centriole. *Andalucia* (Fig. 16Q-T) and *Stygiella* (Fig. 16U-W) belonging to the other suborder (Andalucina) are essentially like Jakobina, *Stygiella* more convincingly showing the acorn (Fig. 16V superimposed on the TP; Fig. 16W grazing the acorn but not TP); thus TZs are uniform across clade Jakobea. In Percolozoa, *Stephanopogon* shows a similar range of structures including an apparent nonagonal fibre (Fig. 16h, apparently representing the periphery of the TP), which may show up so clearly because of the low density just inside it, reminiscent of the lower density on the periphery of *Ancyromonas* (Planomonada) and *Postgaardi* (Euglenozoa) putative TPs (Figs 13T, 17I); this appearance probably reflects the cup-shape of the central thickened zone of the TP (see Fig. 16z), so the lucent 'periphery' is actually *below* the thinner outer edge of TP. Most Percolozoa seem to have a rather dense acorn complex immediately proximal to TP (*Stephanopogon*, *Creneis*, *Pleurostomum, Percolomonas* in Fig. 16Z-k; plus *Dactylomonas venusta* (Hanousková et al. 2019 Fig. 9H) with an even TP lattice and *Psalteriomonas lanterna* with a densely staining acorn complex (Broers et al. 1990 Fig. 15); a central hub is often prominent in the putative acorn, e.g., Fig. 16d. Hemimastigophora micrographs have relatively low resolution, but all have extremely short centrioles (Fig. 20d) overlain by a putative acorn immediately below an amorphous thin TP, thus a very short TZ (Foissner and Foissner 1993). Overall, natozoan TPs are similar to those of dorsates; so I conclude that the ancestral discarian TP was probably a circular thin sheet immediately overlying the acorn, slung between the A-tubule feet, and with a fine-grained irregular supporting web-like lattice (with branching radial and circumferential components) that may have been filled with dense matrix and likely also had a thicker/denser centre or immediately overlying axosomal plate.

In corticates complex extras such as stellate structures, TH, and hubs in longer TZs often distract from the simpler duller TPs. Ciliates being well studied, with numerous TPs per cell and simple type I TZs, are clearer than most. *Paramecium tetraurelia* TP is not overstained, but being dish shaped (Fig. 5A, 20Z), the central part only is shown in Fig. 5H which slices tangentially across the dish base, revealing a honeycomb-like lattice with cells ~10 nm or less that is essentially the same in the thinner better contrasted outer parts as the dense almost obscured thicker centre. The rim of the dish (Fig. 20a,b) has an essentially similar lattice that extends all the way to the A-B links but no further, but the centre of these TSs is confused by superimposition of the axosomal cup and the cp mt fixed within it; Fig. 20Z shows that the base of this mt lacks the characteristic projections found throughout the zone where cp has two mts. Fig. 20c that was washed and stained with ferritin before fixation shows that TP ceases to be curved when such treatment detaches the mt, implying that when fixed without pretreatment the cp is actively deforming it, indicative of the forces TP must withstand during ciliary beating; in this undeformed state the central thickening is clearly distinct from the overlying axosome; it is a 2-ply structure with denser seeming more homogeneous upper layer and less dense lower layer. TPs of green algae *Stigeoclonium* (Fig. 16A) and *Chlamydomonas* show exactly the same 2-ply contrast in the central zone in line with the distal and proximal basal cylinders. Therefore this greater central thickness with visibly different upper and lower laminas is an ancestral feature of all corticates that can be seen in many micrographs shown above.

I now interpret the peripheral TZ lattice in the cercozoan *Bigelowiella* which includes star-point like substructures linked to the inner V-shaped A-B links (Fig. 4A) as its TP rim, not a lattice separate from TP. In *Chlamydomonas* the tomogram showing the TP (Fig. 10M) has a central denser zone, amorphous at low resolution but resolving at high magnification into a fine lattice indistinguishable from that of *Paramecium* and from dorsates described above, but also includes a slightly denser periphery—the base of the upper basal cylinder. This confirms the generality of a fine-meshed central TP in all discaria. This denser zone is surrounded by a paler zone across which slender radial linkers pass to the dense ring on the inner face of the doublets, This outer TP ring was not previously noted in normal thin section TSs suggesting its axial extent may be only a few nm. But a single TP ring is clearly present at the periphery of the TP ring in the LS of the green alga *Nephroselmis* (Fig. 10A). Radial linkers in Fig. 10M are apparently not uniform; some resemble star-points opposite the A tubule, others seem opposite the complex structures in the A-B link positions, which in places resemble normal diamond-shaped paired links of *Bigelowiella* and other corticates, but more often seem Y-shaped or wedge-shaped; I suggest these more elaborate interdoublet structures serve as strong supports for TP and ancillary structures.

In sum, discarian TP core structure is a thin lattice that completely fills the circular space within the doublet A-B links and is likely supported by these links and specialised A-tubule feet that appear in LS as a small dense triangle inside each doublet and in TS as a peripheral ring (TP ring). TP lattice structure universally is an irregular mesh of fine honeycomb with <10 nm cells that in simplest cases pervades the whole TP but is usually thicker and likely at least a two-ply structure with internal axial differentiation. The pattern of a thicker central zone, thin rim, attached peripherally to a TP rim (a tiny dense triangle in LS) is seen across the whole discarian tree from heterokont natates (Fig. 1C) to choanoflagellate dorsates (Fig. 18Z, a) but can be obscured by extra dense matrix or supplemented by extra structures distally or proximally. In many lineages an axosomal plate is a separate distal plate of smaller diameter and with a coarser lattice to which cp is attached that may be close to or more distant from TP. In some this plate becomes more globular or cup-shaped, in others it appears to be integrated so closely with the distal face as to be regarded as part of its structure, the 'axosomal thickening'. In dorsates the axosomal plate is usually circular but in corticates it is commonly somewhat star-like in shape resembling a starfish stretched in one direction, thus not rotationally symmetric. I suspect that this stretching plane may correspond with the plane through the two cp mts, but two mts are attached to the axosomal plate or thickening only in some lineages. In many others one mt only is attached so one might expect a more radially symmetric central structure, perhaps the ancestral condition as it is found in malawimonads whose half plate is best considered a homologue of the axosomal plate not the TP.

Most dorsates and natozoa exhibit no clear radial differentiation of TP beyond the simple circular central thickening and have only inner A-B links, which appear linear in dorsates and V-shaped in some natozoa. Corticates however generally have both outer and inner A-B links and peripheral differentiation of radial linkers that stand out from the often less dense background matrix—in many cases as 18 filaments, sometimes converging on A-tubule feet as 9 broad starpoints. Many corticates at or close to the TP level show more complex interdoublet structures than simple A-B links, sometimes resembling Ys or narrow star points. But without much more study especially tomographic it is not possible to be precise about their axial extent and homologies. A widespread feature of corticate TZs are distal or proximal hub-spoke structures, which are also present in Hemimastigophora, thus are scattered across clade eucorta but are virtually absent from dorsates, and very rare in eozoa—known only in the euglenozoan *Calkinsia* whose double spoke structures likely evolved independently (see above). Some corticates of diverse lineages appear to have a circumferential star-like filament of diameter intermediate between the axosomal boundary and peripheral star elements. Whether this is an integral part of TP or a distal attachment is unclear; it might be related to positioning of the distal TH or basal cylinders (demarcation of these categories is fuzzy) often at or near this position in eucorta, but essentially absent in eozoa and metamonads. As noted above basal cylinder/TH are much more widespread in dorsates that previously recognised, and it is an open question whether these are related to or convergent with those of corticates.

## Key steps in TP evolution

Thus on present evidence corticate TPs are markedly more radially differentiated than those of dorsates or natozoa. Therefore an earlier assumption that ancestral TPs had a rotationally symmetric lattice with a hub-spoke-star substructure is likely incorrect; my depiction (Cavalier-Smith 2014 fig. 4) of the inferred ancestral TP lattice may be quite accurate for corticates, but not for discaria as a whole (which probably had a poorly differentiated irregular lattice) and certainly not for eukaryotes as a whole which as argued here likely had an asymmetrically attached axosomal plate but no TP at all. Thus TP/axosomal evolution progressed in four distinct phases: (1) an axosomal plate evolved attached asymmetrically to the peripheral acorn filament for the basic function of supporting cp nucleation machinery; this converted an ancestral 9+0 cilium to a 9+2 axoneme in stem eukaryotes; (2) inner A-B linkers evolved, which probably diverged in structure between dorsates (simple linear links) and natates; (3) a discoid random fine lattice was slung between them and specially strengthened A-tubule feet linked by the TP ring; and axosomal assembly was blocked immediately distal to the acorn, which made it happen instead by default immediately distal to TP; this generated the ancestral discarian. (4) in the ancestor of corticates TP underwent greater radial differentiation generating (a) star-like peripheral supports and (b) at an intermediate diameter filamentary supports for TH/basal cylinders. (If dorsate TH/cylinders/sleeves are related to those of corticates, at least a facilitating precursor step to 4b must have happened earlier.)

## A general principle of TZ development and evolution

TZ substructures may be assembled sequentially from proximal to distal (just as for centriole, TZ, and 9+2 motile axoneme: Cavalier-Smith 1974). Thus in typical simple type I discaria I suggest that acorn-V assembly precedes TP core assembly, that TP core assembly precedes axosomal plate/thickening assembly and this precedes assembly of the cp nucleation machinery. These four events must happen in sequence before doublets can assemble arms and spokes and cp and its projections can assemble. Correct spatial assembly axially would be ensured if completion of earlier steps are biochemical triggers for later ones. The TP is a two-phase structure like fibreglass or vertebrate collagenous connecting tissue—protein fibres embedded in a jelly-like matrix. Many variations come about through changing the density, thickness or staining density of the matrix (and similar variations in matrix around Y-links), without fundamental changes in lattice architecture, as highlighted in the fungal sections.

Certain mutations would be inherently disruptive and could lead to radical changes in one step. One has only to look at some of the multiply complex phenotypes generated by a variety of single gene mutations in *Chlamydomonas*, e.g., deletion of the δ-tubulin gene in the *uni3-1* mutant may interfere with, but does not totally block, triplet formation and in different cells can block formation of the distal stellate structure and thus the 9+2 axoneme, or allow normal TZ and 9+2 development, or cause assembly of an extra H-like stellate complex in the centriole region (O'Toole et al. 2003). *Uni2* mutations of a phosphoprotein located in the acorn zone drastically upset TZ development causing distortion or multiplication of cylinder-stellate structures, often blocking assembly on the younger cilium (Piasecki et al. 2008; Piasecki and Silflow 2009). Non-lethal mutations of such drastic polymorphic effects could be followed by secondary mutations stabilising one mutant phenotype in a useful way, then a suite of others causing minor improvements in function and viability. In general preexisting TZ structures could be deleted or extra ones inserted or by axial repetition made longer or by partial deletion shorter.

If the TP evolved at the same time as V-filaments, as I argue, their origin may be causally related. It is hard to see how a TP could be inserted between acorn and axosomal plate in one step unless it happened by rescue mutations at least partially correcting a necessarily harmful block to malawimonad-like axosomal assembly. Substantially correcting a really harmful mutation provides a much stronger selective advantage driving suppressor mutant spread than a succession of purely beneficial changes of small effect—I doubt whether purely beneficial, incremental minor mutational changes, could have caused this radical change.

In principle mutations in acorn filament protein(s) might have made them assemble on different doublets in such a way as to create V-filaments, which in turn blocked assembly of the axosomal plate directly at the acorn. For example, the apparent peripheral linker between doublets 4 and 5 could have evolved from a modified peripheral acorn filament, and then serve as substratum for adding radial filaments pointing towards the centre of the lumen, perhaps originating by duplicating components of the radial/lumenal acorn filament. If these converged on the centre to form the characteristic discarian central granule at the V apex, this may have projected distally enough to block normal assembly of the axosomal plate at the acorn axial level, either mechanically or by covering the normal triggering site. Cp assembly would consequently be blocked unless the defect was by chance 'corrected' indirectly by a suppressor mutation in a duplicate of another peripheral filamentary protein (or set of proteins) enabling it/them to polymerise from all nine A-tubule feet at the slightly distal level of the A-B links rather than on just the five acorn doublets, thereby making a simple TP lattice able to serve as a base for immediately-distal assembly of the original axosome, which in consequence likely modified its shape to improve adhesion to and compatibility with TP. Unlike many evolutionary events it seems impossible to imagine a smooth transition in which a hypothetical intermediate could assemble cp at both the acorn and AB-link levels; there had to be a relatively sudden blockage released by instantaneous novelty.

One can envisage more gradualistic changes in most other TP or TZ characters, e.g., the addition of a second (outer) set of AB linkers or radial differentiations of star pints or their duplication above or below TP, or interconversion of separate axosomal plate and closely adherent axosomal thickening of TP.

As noted earlier, many innovations increasing TZ length, either proximal or distal to TP involved inserting novel structures that directly prevented assembly of spokes and dynein arms on doublet. This came about by attaching such structures directly to doublets via A-tubule feet (a previously insufficiently emphasised core TZ component) or indirectly to the TP periphery. By leaving the axosomal plate free this need not interfere with cp assembly as must have occurred during the transition from malawimonads to discaria. These TZ-extending structures include distal TH, nonagonal fibres, basal cylinders, stellate structures, and sleeves of many groups, and proximal spiral filaments (mainly fungi), and hub-spoke structures. As noted above, different phylogenetic lineages are more prone to use different physical devices for TZ lengthening, these depending on predispositions of TP substructure variants that differ amongst lineages. Thus TH/basal cylinders are very widespread across discaria, but stellate structures and hub-spokes are largely restricted to corticates (Table 2). If the primary selective advantage is as argued inhibiting undulation near the ciliary base it may not matter which of them is adopted, so the precise geometry is determined mainly by phylogenetic preadaptation, not the specific TZ substructure, which is why these structures are so phylogenetically informative (often remarkably conserved within major groups but substantially differing between groups), less confused by local ecological adaptations than most characters.

Phylogenetic preadaptation of the TZ may also influence the details of certain ancillary structures, e.g., centriolar alveolar plates (AP) of ciliates. Thus the peripheral zone of *Paramecium* AP (Fig. 5D, L) is rather similar to that surrounding the TP of *Chlamydomonas* (Fig. 10M). Thus both have interdoublet dense Y-like structures (putatively peripheral anchors for the plates) and also separate radial links to the A tubules; thus the basic architecture of the AP periphery might have evolved by duplication and divergence of similar components of the ancestral corticate TP because they serve similar functions and are available as precursors. By contrast the central zone of AP differs fundamentally in function as it need not (and must not) interact with axosomal structures, so it must be very different; it must offer mechanical support but its detailed architecture does not matter greatly; economy and ease of assembly led to a lattice (major ring with 18 radial struts and central filler an amorphous sheet, collectively entirely different from that of TP.

## Origin of TZ/centriole differentiation

The TZ and centriole are morphogenetically more closely related than either are to the 9+2 axoneme in that both cp-facing spokes are replaced by shorter A-tubule projections: A-tubule feet in the TS; A-B feet in the distal centriole; pinheads in the proximal (cartwheel) centriole. I conjecture that the TZ A-tubule feet and centriolar pinheads are distantly related and might have diverged from a common ancestor when cilia evolved. Their divergence may even have preceded the origin of axonemal spokes and arms. Other structures also may have been repurposed between centriole and TZ by modifying their axial assembly position. For example the nonagonal fibres or tubes of distal TZ appear very similar to the nonagonal fibres in the distal centriolar zone of numerous discarian lineages. The centriole is subdivided axially into distinct distal and proximal zones, as is the axoneme into 9+2 and TZ; both may use fundamentally similar axial labelling principles to specify axial positional information. Functionally and structurally the TZ acorn-V is closely associated with the underlying centriole so must interact during development.

As suggested above, the original function of the roughly biradial acorn-associated axosomal plate and associated non-rotational symmetry of the acorn filament system may have been to provide a quasi-biradial nucleation site for cp mts, which might have originated before centrioles evolved C-tubules and were still doublets, i.e., at stage c (possibly even at stage b, the 9-singlet stage) of the ciliary origin scenario of Cavalier-Smith (2014 fig. 4); I now argue that TP originated later than in that scenario significantly *after* establishment of a malawimonad-like quasi-biradial axosome. Axosomes cannot be strictly biradial as cp 1 and 2 differ in attached proteins and are not bilaterally symmetric. Though pinheads and A-tubule feet could have evolved at the 9-singlet stage, as Y-links may also have done, I suggest that centriolar A-B feet and centriolar axial differentiation probably evolved only after doublets, and that TFs (predominantly attached to B tubules) probably did so also. A-C links presumably immediately followed C-tubules, likely when arms and active bending arose. A secondary consequence of acorn filament origin may have been distinct radial labelling of all nine doublets/triplets, necessary for attachment specificity of the chiral centriolar roots as Geimer and Melkonian (2004) noted.

## Centriole growth and the acorn

When I discovered the nine singlet/cartwheel intermediate in centriolar assembly in *Chlamydomonas* (Cavalier-Smith 1967, 1974; Randall et al. 1967) I assumed that centriolar growth proceded unidirectionally from base to tip starting with the cartwheel zone, much as argued here for sequential TZ assembly. However as the acorn structure is at the apex of much shorter interphase procentrioles as well as longer mature centrioles (Geimer and Melkonian 2004), interphase procentrioles must grow into centrioles at their base (or less likely by inserting tubulin interstitially between the acorn and distal triplet zone). A near-full length probably 9-singlet centriole during de novo centriolar assembly after *C. reinhardtii* meiosis (Cavalier-Smith 1974 Fig. 29) showed that centriole formation must be more complex that simple basal to distal growth. I therefore raised the possibility that 'developing 9-triplet basal bodies are longer than the cartwheel-containing region of mature ones and contain a cartwheel throughout their length, but that this is subsequently broken down in the distal region (possibly also to some extent in the proximal region ...' (Cavalier-Smith 1967 p. 198) and 'that during development the cartwheel is present as an internal scaffold throughout the length of the developing basal body and is subsequently removed from its distal end.' (Cavalier-Smith 1967 p. 208). A purely temporary scaffold function was also indicated by cartwheel loss in some vertebrate centrioles when mature (Kalnins and Porter 1969). The ability of cartwheels to self assemble in vitro (Guichard et al. 2018) is consistent with this being the first step in centriole formation and the 9-fold-symetry of its hub determining the overall 9-fold symmetry of the centriole as proposed (Cavalier Smith 1967; Cavalier-Smith 1974).

Beech et al. (1991) found that in four other related green algae nascent centrioles often had either cartwheels or cartwheels plus single mts projecting from their base and that cartwheels can be longer than in mature centrioles, proving that *partial basal disassembly* of cartwheels and cartwheel-singlets must occur during centriole growth. In cercomonad clade A1 Karpov et al. (2006) found that the anterior (younger) centriole has a basal cartwheel but the mature posterior one did not, proving that cartwheels can be entirely disassembled during centriole maturation, thus are essential for early growth but not mature functions, consistent with being fundamentally developmental scaffolds. Tomography confirmed that in the mitotic cycle new *C. reinhardtii* centrioles at metaphase have singlet mts with elongated cartwheel, which add doublets/triplets distally and then disassemble the basally protruding singlet-cartwheel zone (O'Toole and Dutcher 2014) as inferred by Beech et al. (1991). Thus distal assembly and basal disassembly underlie procentriole to centriole conversion in green algae.

*Chlamydomonas* centriole maturation apparently differs in the successive duplications in a single multiple fission cell cycle. If daughter cells exceed a set size threshold they divide again almost immediately without further cell growth, this being repeated till size falls below that threshold (Craigie and Cavalier-Smith 1982; Umen and Goodenough 2001). Thus in second or later centriole maturations within one multiple fission cycle there is no long delay between the formation of a short procentriole capped by an acorn and its growth to full length as there is for the first. Instead the basally singlet, distally triplet intermediate is thought to be converted to a full length triplet centriole by growing B and C tubules towards its base, thus omitting basal trimming of A singlets (O'Toole and Dutcher 2014). Presumably this full-length centriole would then grow TZ doublets at its tip, adding acorn and TFs (details unclear), in which case such a centriole would not have gone through a short procentriole stage already with added acorn. Thus in multiple fission cell cycles the first centriole duplication in growing cells makes a 'dormant' centriole (Beech et al. 1991) only activated into growth later, whereas subsequent duplications in the same cycle yield non-dormant centrioles able to grow at once without reactivation.

Cryoelectron tomography shows that in humans the long 9-singlet stage matures differently: B tubules, then Cs, are added to its *middle* (not distally) and grow at both ends till triplets extend its full length (Guichard et al. 2010). Thus basal trimming of singlets may be absent. Many molecular details are known of procentriole assembly, procentriole to centriole conversion, and differences in ciliated centrioles in animals and that processes may differ amongst cell types (Nigg and Holland 2018) and species (*Caenorhabditis* even has mature singlet centrioles), but we are largely ignorant about when and how acorns are added and their role in duplication or ciliation. Evolution of multiciliate animal epithelial cells inevitably involved processes not involved in ancestral opisthokont vegetative cell cycles with a single pair of centrioles, as also necessarily did the special terminal differentiation of sperm.

In fungi, unlike choanoflagellates and most animals normal bicentriolar vegetative cell cycles are also absent. Their short barren centriole is not comparable to the short procentriole of *Chlamydomonas* if it represents the mature posterior centriole as argued above, as it will never need to grow to full length and become ciliated. In chytridiomycete fungi *Polychytrium* and *Karlingiomyces* the barren centriole is somewhat shorter than the ciliated one yet still has at its tip a short doublet TZ region with structure most simply interpretable as an acorn-complex (Fig. 18A, F). The barren centriole of the zoomycete *Olpidium* is even shorter yet has a very short distal zone without a cartwheel at its apex (Fig. 15N), which I suggest is likely also an acorn complex that serves to cap its barren centriole and prevent its growth. I suggest that the short barren centrioles of fungal zoospores are permanently prevented from growth to full length and ciliation, like the barren centriole of the uniciliate green alga *Monomastix* (Heimann et al. 1989) and some uniciliate heterokont algae (*Mallomonas splendens*, pedinellids: see Beech et al. 1991). Fungi probably differ in that the barren centriole may not have borne a cilium in previous cell cycles. It is like the dormant procentriole of *Chlamydomonas* but differs in growth being permantly blocked. The likely retention of an acorn complex by the fungal barren centrioles even though they never need to grow cilia makes it likely that cell-cycle controlled centriolar dormancy of a procentriole until after cell division is a universal property of all eukaryotes with cilia, irrespective of whether they have binary or multiple fission cell cycles (there is much ultrastructural evidence across eukaryotes that procentrioles are formed hours before cell division in binary fission cycles). I suggest imposition of centriolar dormancy was a common-ancestral discarian process dependent on prior assembly of an acorn complex, and likely evolved earlier at the malawimonad half acorn stage. If so it would have been easier for ancestral fungi to lose the ability to reactivate a dormant centriole after division than to evolve a completely novel mechanism of imposing a block to growth that did not require prior assembly of the acorn complex. Thus likely acorn retention in the fungal barren centriole is an example of developmental/phylogenetic inertia—evolution following the line of least resistance, not simply making a uniciliate cell with no barren centriole in the first place as would an intelligent designer. Calling the short barren centriole a procentriole would be developmentally misleading as it does not develop into a centriole or cilium in the next cell cycle as do procentrioles of *Chlamydomonas* or trypanosomes, but must either be destroyed or diluted out by generations of vegetative growth; it is really a postcentriole. Its retention is itself evidence that opisthokont cilia evolved from the anterior younger cilium of diacentrids. The uniciliate 9+0 sperm of diatoms certainly retained the heterokont anterior tinsel cilium with no trace of a barren centriole, showing that they did lose the posterior centriole by losing ciliary transformation.

## Appendix

### Diagnoses of New Taxa

Diagnoses of new taxa not included in Table 1 or the preceding text are given here:

### Kingdom Protozoa: phylum Choanozoa

**New family Parvulariidae** (within order Fonticulida of class Cristidiscoidea): **Diagnosis:** spherical to elongate aerobic, filose amoebae with cell bodies not over 6 μm (unlike larger *Nuclearia*) with one or two nuclei; cristae flat to discoid; walled cysts may contain one, two or at least three cells. Phylogenetically closer to *Fonticula* than to *Nuclearia* or *Lithocolla* (Galindo et al. 2019). Type genus *Parvularia* López-Escardó and Torruella in López-Escardó et al. 2017 p. 177.

### Kingdom Fungi: phylum Chytridiomycota

**New class Parachytriomycetes Diagnosis:** Zoospore with one to many cilia; uniciliate species have (a) a cone of microtubules radiating from its base towards the nucleus and (b) a distal fan of microtubules radiating in the plane orthogonal to the centriolar axis from an arc of dense fibrillar material attached distally to the ciliated centriole. Aerobic free living species (Monoblepharidales) have additional concentric dense filaments near fan basal arc giving a striated appearance and a second much shorter, barren centriole (parallel or at acute angle to main one, never orthogonal), both absent in anaerobic gut parasites (Neocallimastigales, sometime multiciliate). Centrioles not directly attached to nucleus (unlike Allomycetes) or to mitochondrion, without striated rhizoplast connecting to nucleus (unlike zoomycete Olpidiales). Transition zone type I or short type II, with either nonagonal tube distal or spiral fibre proximal to transition plate; transition helix or distal dense plug absent (unlike most Chytridiomycetes). Phylogenetically defined as the last common ancestor of *Monoblepharis* and *Neocallimastix*, plus all its descendants. Sole orders Monoblepharidales and Neocallimastigales.

### Phylum Zygomycota: Class Glomomycetes Cavalier-Smith

**New order Sanchytriales Diagnosis:** monocentric fungal parasites of the filamentous xanthophyte alga *Tribonema* that form epibiotic sporangia. Uniciliate zoospores can be amoeboid with a pseudocilium with long doublet centrioles and singlet pseudociliary shaft without a central pair microtubule; penetrate cell wall and form intracellular rhizoids. Phylogenetically closer to *Gigaspora*, *Diversispora* and *Rhizophagus* than to *Mucor*, *Basidiobolus* or *Olpidium*. Sole family Sanchytriaceae Karpov & Aleoshin 2017 (*Sanchytrium*, *Amoeboradix*). **Comment.** Glomomycetes (arbuscular-vesicular fungi plus a few algal symbionts; there was no sound reason to change the name to Glomeromycetes) was too highly ranked as a phylum (Oehl et al. 2011); its three previously known clades need no higher rank than orders: Glomales Morton & Benny 1990 (merits two suborders); Geosiphonales Cavalier-Smith 1998 (Archaeosporales Walker & Schüssler in Schüssler et al. 2001 is a junior synonym); Paraglomerales Walker & Schüssler in Schüssler et al. 2001.

### Kingdom Chromista: subkingdom Harosa

**New infrakingdom Telonemia. Diagnosis:** Pyriform phagoheterotrophic uninucleate biciliates with tubular mitochondrial cristae and acronematic cilia with strongly divergent centrioles inserted below a prominent rostrum. Rostrum and side of cell supported by two dissimilar multilayered cortical sheets of microtubules with associated microfibrillar layers, which are probably the laterally hypertrophied posterior centriolar roots (A2 the I-fibre linked right root) and P layer the left root with C-fibre (see Cavalier-Smith et al. 2015); eukaryotic algal prey cells ingested at broad end of cell after cortical sheets terminate and may separate. Ciliary transition zone type I with transitional plate close to the cell surface; transition helix or secondary distal plate absent, unlike most Hacrobia or Heterokonta; transitional hubs absent unlike most rhizaria. One cilium with tripartite tubular hairs points forward (*Lateronema*) or both cilia (probably without hairs) point backwards (*Telonema*) during swimming. Extrusomes present or absent. Filogranular material near centrioles. Microbodies and Golgi dicytosomes present. Comprises only phylum Telonemia (Shalchian-Tabrizi et al. 2006) with single class Telonemea Cavalier-Smith 1993. Phylogenetically sister to infrakingdoms Rhizaria and Halvaria (Strassert et al. 2019).

### Kingdom Chromista: subkingdom Hacrobia

**Phylum Cryptista**

**New subphylum Endohelia: Diagnosis:** Axopodial phagoheterotrophs with simple flattened extrusomes and transnuclear cytoplasmic channels traversed by axopodial axonemes nucleated by globular centrosomes with distinct shell and core. Two pairs of cilia with parallel centrioles (*Tetrahelia*) or lacking cilia and centrioles (*Microheliella*). Tubular mitochondrial cristae. Non-kinetocyst flattened extrusomes.

Sole class Endohelea Cavalier-Smith in Yabuki et al. (2012) em. Revised diagnosis as for Endohelia.

Comprises orders Microhelida Cavalier-Smith in Yabuki et al. (2012) and **Axomonadida** Cavalier-Smith in Yabuki et al. (2012) as emended here by excluding *Tetradimorpha radiata* and *tetramastix*. **Diagnosis revised** to correspond with that of sole included family: **Tetraheliidae fam. n. Diagnosis** Tetraciliates with four standard length centrioles (not extra long as in *Heliomorpha* and *Tetradimorpha*) and axopodia nucleated by a globular centrosome with distinct granular shell and microfibrillar core; centrioles arranged as two pairs (parallel within a pair; 30° between pairs) linked basally by amorphous material connecting them to the centrosome. Lateral Golgi dictyosomes on either side of nucleus. Axopodia with numerous irregularly arranged microtubules and irregularly flattened extrusomes, not kinetocysts as in *Heliomorpha* and *Tetradimorpha radiata*. Large cell size (> 60 μm), with centrosome 18-20 μm and thick pseudopellicle layer beneath plasma membrane. Has lazily swimming purely flagellate stage with fully retracted axopodia. Type genus ***Tetrahelia*****gen. n**. **Diagnosis:** as for family Tetraheliidae. **Etymol:*** tetra-* L. 4 (combining form) refers to four cilia; *helio-* L. sun to the radiating axopodia. Type species *Tetrahelia pterbica* comb. n. Basionym *Tetradimorpha pterbica* Mikrjukov and Patterson in Mikrjukov 2000 p. 109*.*

### 28 Major conclusions

1. Phylum Malawimonada has a shorter and simpler ciliary transition zone (TZ) than other eukaryotes, which is fundamentally asymmetric, a segment-like axosomal plate being linked by the peripheral acorn filament to only five doublet mts. A transition plate (TP) with 9-fold symmetry is absent as are V-filaments linked to any of the other four doublets.
2. All other ciliated eukaryotes and their non-ciliate descendants constitute clade discaria characterised ancestrally by (1) a circular disc-like TP with a filamentous skeleton having a peripheral rotationally symmetric star-pattern surrounding an irregular lattice, often thicker in its central zone; and more proximally (2) a complete acorn-V filament complex, which forms the proximal boundary of their TZ. Even diatoms which have lost central pair (cp) mts and centriolar triplet C tubules retain both structures as well as Y-links and A-B links in their TZ.
3. In many discarian groups TP and the acorn-V system have been conflated and the fundamentally different type of TZ 'transverse plates' they represent not fundamentally understood. I correct these misattributions in several groups, notably ciliates, fungi, and trypanosomes.
4. Critical reevaluation of outgroup-rooted multiprotein trees for 26 proteins of eubacterial origin places the root of the eukaryote phylogenetic tree between protozoan subkingdom Malawimonada and clade discaria.
5. I divide discaria into dorsate and natate subclades or 'supergroups', both ancestrally aerobic and non-amoeboid. Dorsates comprise opisthokonts, Apusozoa, Amoebozoa, and Sulcozoa. Natates comprise superkingdom Corticata (Plantae, Chromista) plus new protozoan subkingdom Natozoa [i.e., Metamonada, Eozoa/Discoba, Hemimastigophora]; a second set of (outer) A-B links likely evolved in or close to the ancestral natate which may have given their somewhat more complex TP extra support.
6. Ancestrally dorsates were dorsoventrally flattened benthic non-pseudopodial biciliates gliding on their ventral surface by their posterior cilium, with rigid dorsal pellicle, TZ transition helix/basal cylinder, and orthogonal centrioles linked by lateral connectors, one amorphous and one striated.
7. Ancestrally natates were non-amoeboid swimming, planktonic non-gliding biciliates feeding by catching prey drawn into their ventral feeding groove by ciliary water currents, with orthogonal centrioles usually linked by a single central distal striated connexion, without ultrastructurally distinct dorsal pellicular layer.
8. Dorsates split primarily into non-pseudopodial planomonads with a single pellicular layer and podiates with branching myosin-II-based pseudopodia emitted from the ventral groove for feeding. Dorsate TZs usually have inner A-B links only; lack hub-spoke structures; but may ancestrally have had a distal basal cylinder/TH; their V-filament system probably differs somewhat from that of corticate natates.
9. Podiates comprise Varisulca and the torcid clade. Subphylum Varisulca of Sulcozoa comprises three relict groups of sharply different feeding modes: *Mantamonas* retaining ancestral gliding; diphylleids—planktonic swimmers with pseudopodial feeding groove; and Rigifilida that lost cilia and use branching filopodia emerging though a radially symmetric ventral collar for feeding.
10. Torcids were ancestrally posterior ciliary gliders able to emit pseudopodia but with longer active anterior cilium than *Mantomonas*, hypothetically feeding by trapping bacteria using their anterior cilium and a continuous basal collar as in the unikont amoebozoan *Phalansterium*. 'Torc' refers to this continuous collar arguably retained by *Phalansterium* and Apusomonadida, but lost by other Amoebozoa and Breviatea—a likely synapomorphy for the clade (sometimes misleadingly called amorphea), i.e., obazoa plus Amoebozoa.
11. The ancestral opisthokont was probably a stem choanoflagellate that evolved from a stem apusozoan by losing the posterior gliding cilium (and associated roots) and converting its apical continuous collar into a discontinuous collar of microvilli around the anterior cilium to allow filter feeding and more efficient trapping of suspended bacteria. That entailed evolving actin bundles not a 3D mesh for collar support and reorienting the former dorsal fan microtubules orthogonally to the ciliated centriole to allow their attachment more widely to increase collar width and enlarge filtering capacity.
12. The six concentric filaments that support the pericentiolar apical fan of choanoflagellates, some Chytridiomycota, and *Phalansterium* are probably homologous and go back at least to stem torcids. Retention of an orthogonal striated fan in fungi after their loss of phagotrophy is evidence that they went though a stem choanoflagellate ancestry with microvillar collar not just a torcid ancestry with continuous collar and more longitudinal fan. This supports retention of protozoan phylum Choanozoa for all stem opisthokonts (i.e., those other than animals, fungi, and protozoan phylum Opisthosporidia whose novel infection apparatus justifies its phylum rank).
13. Only four phyla are needed for kingdom Fungi: Chytridiomycota all have ciliate zoospores, but only a few Zygomycota, whose subphyla Mucoromycotina and Zoomycotina lost cilia independently of each other and of Neomycota (=Dikarya). Chytridiomycete TZ dense plug may be an ancestral opisthokont character, retained even by sponges, radically reinterpreted as dense matrix hiding TP/TH and Y-links. This matrix was multiply lost in fungi and other opisthokonts some of which retained distal nonagonal fibres or proximal spiral fibres likely ancestrally hidden by dense TH and plug matrix.
14. I divide kingdom Protozoa into three subkingdoms: Malawimonada with only the purely flagellate phylum of that name; Sarcomastigota with a mix of amoebae and ancestrally benthic flagellates with five phyla (ancestral Sulcozoa, Apusozoa, and Choanozoa; derived Opisthosporidia and Amoebozoa, clades which respectively became parasites and pseudopodial movers); and Natozoa ancestrally swimmers, also with five phyla [ancestral biciliate 'excavate' phylum Eolouka (Jakobea, Tsukubamonadea), anaerobic tetrakont Metamonada, double bikont amoeboflagellate Percolozoa, biciliate cytopharyngeal Euglenozoa, and 'multikinetid unikont' Hemimastigophora].
15. Distal TZ hubs, more widespread in corticates than previously recognised, probably arose in the last common ancestor of corticates and Hemimastigophora, a clade I call eucorta.
16. The TZ hub-lattice structure of Cercozoa is here reinterpreted as a composite of two thin structures superimposed within a single thin section: a distal central hub widespread across eucorta, and a peripheral lattice that forms a previously unrecognised part of the TP lattice of discaria generally.
17. The so called 'terminal plate' of ciliates is not a TZ plate (thus neither TP, not acorn-V) but a centriolar triplet-associated structure unique to ciliates with a third lattice pattern, which likely functions to attach their centrioles laterally to cortical alveloli. Use of this same name in other groups like trypanosomes or fungi is therefore misleading (where it usually unwittingly designates the acorn-V lattice), so in ciliates I rename it alveolar plate.
18. I group classes Picomonadea, Rhodelphea as new phylum Pararhoda, in turn grouped with Rhodophyta as new infrakingdom Rhodaria of Plantae within subkingdom Biliphyta, which also includes Glaucophyta with fundamentally similar TZ.
19. The biliphyte long TZ distal region is unique in eukaryotes, with a constriction-associated annular septum well distal to TP and a long basal nonagonal tube (NT) close to doublets between TP and annular septum plus often a shorter somewhat loose TH distal to the annular septum. Biliphytes also share dissimilar simple central hubs both proximal and distal to TP.
20. During the origin of Viridiplantae multiplication of one peripheral component of the corticate peripheral TP lattice above and below TP formed multi-tier proximal and distal stellate structures and basal cylinders. Separate duplication of the central component of TP probably formed the proximal septum of the proximal cylinder, enabling connection to the acorn-V complex to be maintained by an asymmetric connector that replaced the biliphyte proximal hub. Initially the biliphyte distal hub connecting to the central pair and TH were retained but the distal hub was lost in most Viridiplantae and TH lost by all except Pyramimonadales. The biliphyte NT was lost, allowing its annular connexion to move down to the ancestral position just above TP. Thus origin of stellate structures required less fundamental innovation than previously supposed.
21. Glaucophyte pseudocilia (in *Glaucocystis* and *Gloeochaete* only) are highly modified hypertrophied TZs that retain Y-links, TP and NT, but largely or entirely lost cps.
22. Cryptista may have one, two or three TZ plates but only the lowermost is TP. Rollomonad Cryptista have a long TZ proximal to TP with a nonagonal tube. I transfer *Tetradimorpha pterbica* to new genus *Tetrahelia*, here grouped with *Microheliella* in existing cryptist class Endohelea, now the sole member of subphylum Endohelia; *Heliomorpha* is transferred to Cercozoa and Telonemia to Harosa, and *Picomonas* to Rhodaria, which makes cryptists more homogeneous in TZ.
23. Haptista have one or two TZ plates but only the lower one is TP. All corticate TPs have a peripheral lattice composed of wider star points in phase with A tubules and narrower ones out of phase. I explain how the apparently distinct TPs of prymnesiophytes (including their great diversity) and pavlovophytes diverged from a common ancestor.
24. Plantae ancestrally had a TH likely to be homologous with that of Heterokonta and even a few haptophytes. As several heterokont lineages and several opisthokont lineages independently lost TH we must take seriously the likelihood that at least one or two gyres of a TH were ancestrally present in Corticata, and the possibility that TH were also present in the ancestral natate and discarian (which would require only three extra losses in Natozoa).
25. Halvaria (heterokonts, alveolates) ancestrally probably had a bell-shaped TZ resembling that of heterokont Labyrinthulea, comprising a bell-shaped distal plate embracing a single cp mt, and associated with the axosome and ciliary constriction and immediately underlying flat TP. Differential loss of different parts of the bell likely generated the axosomal cup of ciliates, the axosomal sleeve of Colponemea, the thin basal cylinder of Myzozoa, and TH of non-labyrinthulean heterokonts. I explain how polyphyletic widening of the distal structures to accommodate two mts led to many TZ variants.
26. The function of polyphyletic evolutionary TZ lengthening may be basal bend suppression; conxcopy /y "\\10.82.5.60\springer\Prod\jwf\template\Standard\\Contentchecker\*.txt" "C:\Program Files\Arbortext9.1\APP-D Unicode\"copy "\\10.82.5.60\springer\Prod\jwf\template\Standard\\Contentchecker\*.dtd" "C:\Program Files\Arbortext9.1\APP-D Unicode\"copy "\\10.82.5.60\springer\Prod\jwf\template\Standard\\Contentchecker\*.jar" "C:\Program Files\Arbortext9.1\APP-D Unicode\"copy "\\10.82.5.60\springer\Prod\jwf\template\Standard\\Contentchecker\Validation*.*" "C:\Program Files\Arbortext9.1\APP-D Unicode\"del "D:/Programs/ProductionJournal/Temp/ccc.bat"vergent changes in TZ length in many lineages may be associated with modified ciliary beating patterns, which are extremely diverse across lineages.
27. Two dramatic cases of TZ lengthening [*Calkinsia* within euglenozoan class Postgaardea (in natates) and *Phalansterium* within amoebozoan class Variosea (in dorsates)] show far greater axial ultrastructural diversity than in short TZ relatives and illuminate the contrasting evolutionary potential of natate and dorsate TZs.
28. Diatoms, despite losing the cp and spokes ~110 My ago, retain dynein arms, undulatory motility, and a very standard type I TZ with TP, Y-links, A-B links, and acorn complex, fundamentally similar to other heterokonts that lost the TH, illustrating TZ structural conservatism in the absence of TZ length changes.

## Declarations

Thomas Cavalier-Smith: Deceased
